# An integrated console for capsule-based, automated organic synthesis†

**DOI:** 10.1039/d1sc01048d

**Published:** 2021-04-13

**Authors:** Tuo Jiang, Samuele Bordi, Angus E. McMillan, Kuang-Yen Chen, Fumito Saito, Paula L. Nichols, Benedikt M. Wanner, Jeffrey W. Bode

**Affiliations:** Laboratory for Organic Chemistry, Department of Chemistry and Applied Biosciences, ETH Zürich 8093 Zürich Switzerland bode@org.chem.ethz.ch; Synple Chem AG 8093 Zürich Switzerland wanner@synplechem.com

## Abstract

The current laboratory practices of organic synthesis are labor intensive, impose safety and environmental hazards, and hamper the implementation of artificial intelligence guided drug discovery. Using a combination of reagent design, hardware engineering, and a simple operating system we provide an instrument capable of executing complex organic reactions with prepacked capsules. The machine conducts coupling reactions and delivers the purified products with minimal user involvement. Two desirable reaction classes – the synthesis of saturated N-heterocycles and reductive amination – were implemented, along with multi-step sequences that provide drug-like organic molecules in a fully automated manner. We envision that this system will serve as a console for developers to provide synthetic methods as integrated, user-friendly packages for conducting organic synthesis in a safe and convenient fashion.

## Abstract 2

Using a combination of reagent design, hardware engineering, and a simple operating system we provide an instrument capable of executing complex organic reactions using prepacked capsules with minimal user involvement.

## Graphical abstract

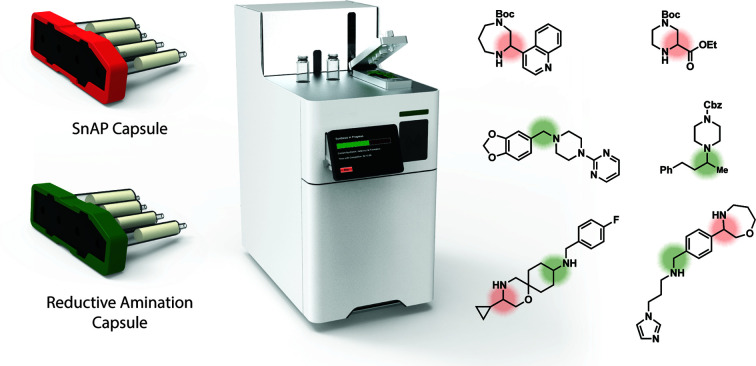

## Introduction

Synthetic organic chemistry has long been practiced with an assortment of standard – but not standardized – equipment including stir plates, heating devices, cooling baths, glass flasks, and a menagerie of funnels, filters, extractors, and traps. While this flexibility has enabled synthetic chemistry to be executed with minimal equipment at relatively low cost, the lack of standards for reagents and apparatus contributes to poor reproducibility and a reputation of capriciousness. More significantly, traditional practices impose inherent safety hazards and stymie efforts at automation of basic techniques. As noted by Cronin,^1^ IBM,^2,3^ Jensen, Jamison,^4^ and others the deficiency of standard equipment precludes the “digitalization” of organic synthesis, which is necessary for mechanizing not only the production of valuable organic molecules but also collecting and annotating data that will make complex molecule synthesis more predictable and reliable.

**Fig. 1:**
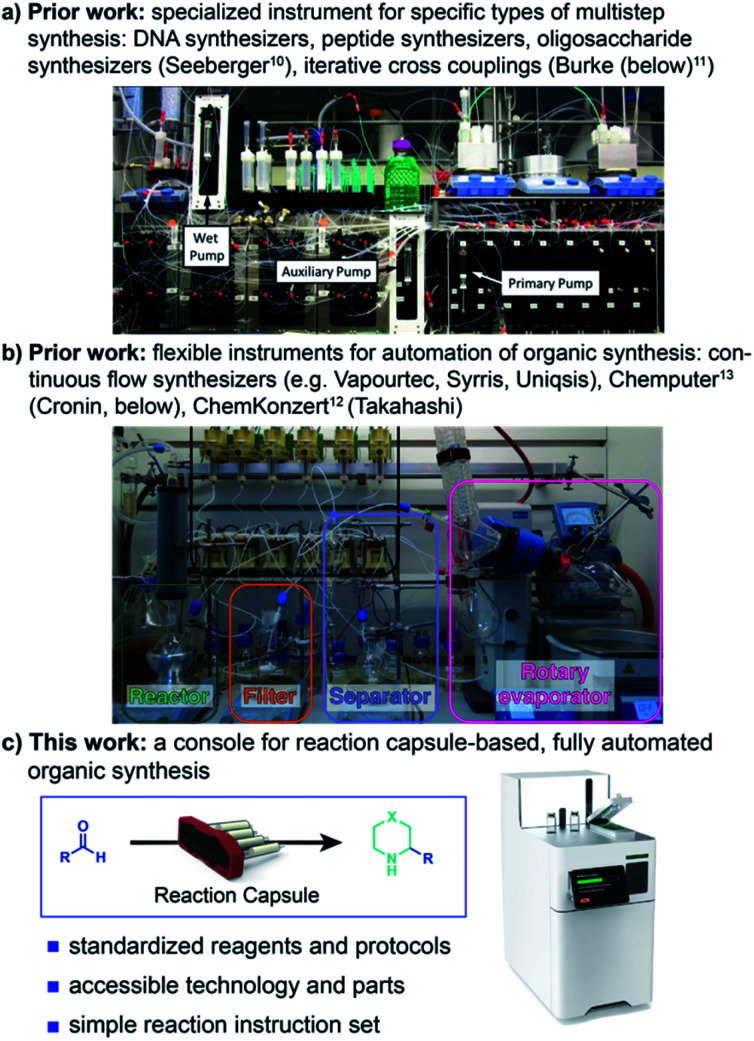
Approaches towards automatic organic synthesis with specially developed instruments.

The widely recognized desire to automate synthesis is manifested in a delightful array of devices and contraptions for organic synthesis.^5,6^ Innovative and robust chemical operating systems for the iterative synthesis of oligomeric molecules, such as peptides^7,8^ and DNA,^9^ have led to widespread deployment of automated synthesizers for access to these molecular classes. Researchers have also made outstanding progress on extending these concepts to more demanding oligomers, including carbohydrates^10^ and polyolefins^11^ (Fig. 1).

More specialized targets that require diverse – and often operationally demanding – experimental conditions have proven more challenging to access in an automated fashion, especially for *de novo* synthesis without an established route. Current approaches include inspiring endeavours to add elements of automation to traditional organic synthesis equipment, as exemplified by the ChemKonzert^12^ and the Chemputer,^13^ as well as grander implementations such as Eli Lilly's Life Science Studio.^14^ At the current stage of development, however, these approaches require considerable manual intervention for setup, cleaning, and programming, precluding their operation by non-specialists.

As an alternative to automating classical techniques for organic synthesis, numerous practitioners have advocated new modalities. For example, flow chemistry takes a fundamentally different approach to organic reactions, which is particularly well-suited for reliable scale up and production of optimized routes.^15,16^ Highly specialised, self-contained systems are used to prepare radioactive tracers in tiny quantities.^17^ Others have promoted alternatives to traditional techniques for workup, separation, and purification. This is perhaps best exemplified by the use solid supported reagents and scavengers for complex molecule synthesis, as champanioned by Ley and co-workers.^18,19^ These solid-supported reagents and scavengers have evolved into disposable kits for reaction screening/workup that are widely used in the discovery phase of the pharmaceutical industry. Despite the welcome step away from some of the unsavory and time-consuming rituals of organic chemistry, the lack of a standard platform and equipment for implementing these concepts has hampered their widespread adoption.

In this report, we document a compact, capsule-based, fully automated console for executing organic synthesis, including the formation of saturated N-heterocycles, reductive amination, and multi-step combinations. By recognizing that preparative organic synthesis can be reduced to a few basic operations such as mixing, heating, and transfers coupled with pre-packaged reagents and resins, reaction protocols can be scripted and executed with high reproducibility. The resulting union of hardware engineering, software development, reaction innovation, and purification technologies provides a hands-free approach to preparing organic compounds prized in drug discovery. This ‘operating system’ for automating organic synthesis obviates the need to directly handle most organic reagents and waste, resulting in a safe, user friendly platform suitable for unsupervised operation. We envision that future organic methodologies will be implemented using the combination of optimized reagent capsules and short software scripts, thereby both improving safety and enabling their direct adoption by end users, regardless of their technical knowledge of preparative organic chemistry.

## Results and discussion

**Fig. 2:**
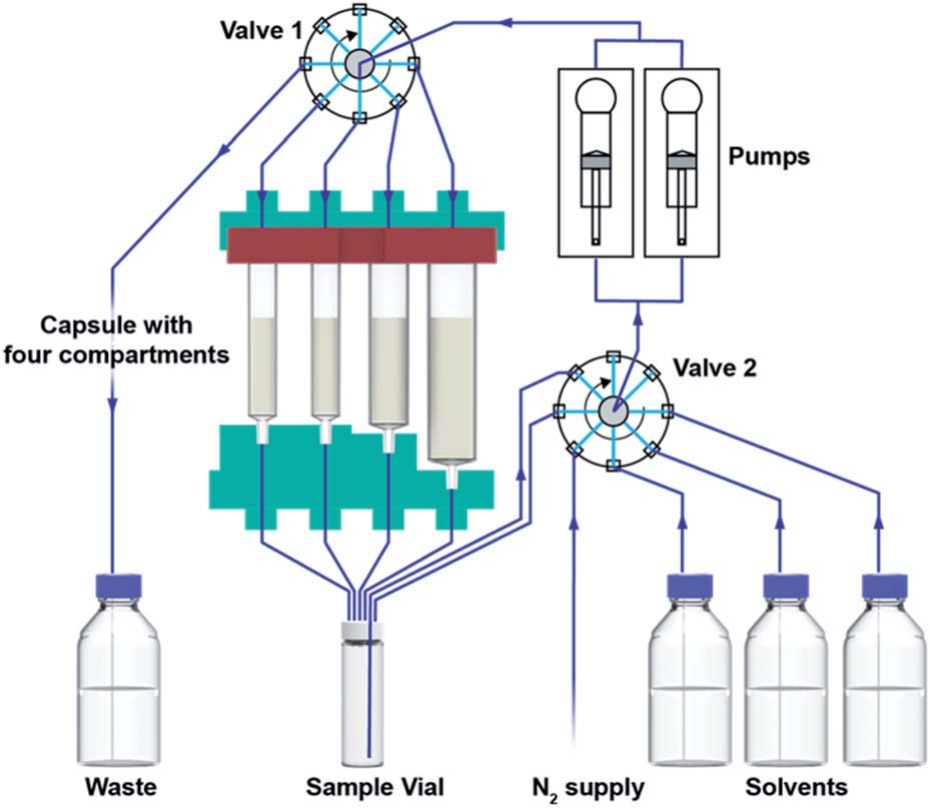
Fluidics setup of the automated console. The setup is composed of two valves, two syringe pumps and the heatable capsule holder. Through different valve settings solvents can be pumped from the solvent reservoirs or sample vial into the individual compartments of the capsule or into the waste. The connected nitrogen supply facilitates preserving an inert environment.

**Fig. 3:**
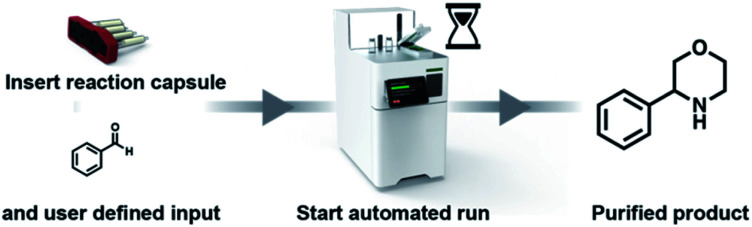
The user adds the starting material to the reaction vial, which is loaded together with the cartridge into the console. The automated reaction is started on the touchscreen. After completion the user can pick up the product in the reaction vial.

In order to facilitate consistent assembly and operation of a modular console for automated organic synthesis, we sought to construct it from off-the-shelf components. Accordingly, our design began by combining commercial rotary valves and syringe pumps with Teflon or glass components (Fig. 2) that were compatible with organic solvents. With this straightforward setup we can use up to five solvents or mixtures, while maintaining a container for waste collection. Although this modest and somewhat inflexible configuration will preclude some reaction types, the majority of useful methodologies are operationally compatible with this design. These choices also facilitate construction within defined specifications and reduce set-up parameters that might limit operation by non-specialist users. The key liquid handling elements were supplemented by an electromagnetic stirrer, heating elements in the vial and capsule holders, a touch screen interface, and an RFID scanner (Fig. 3).

**Fig. 4:**
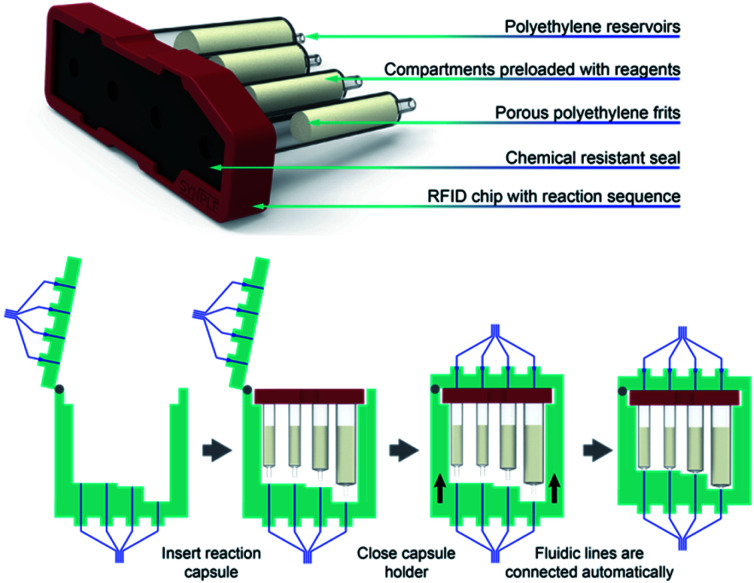
The capsule consists of four compartments that are prefilled with the necessary reagents. A chemically resistant seal on top and luer connections at the bottom interface with the console. After the capsule is inserted into the capsule holder, the fluidic lines connected automatically *via* motorized sledge.

An automated reaction sequence is comprised of a series of sequentially executed fundamental instructions (Table 1). Only these seven instructions were sufficient to operate all the components of the device – pumps, valves, heaters, stirrers and capsule holder – to constitute a synthetic sequence. Additional parameters are used to define a specific operation. The microprocessor is capable of storing a multitude of reaction sequences including a self-cleaning program, which enables rapid consecutive runs of the console, much like modern, capsule-based automated chromatography systems or coffee makers. With an eye towards preventing operational mistakes, the capsules include an RFID chip read by the console, which ensures the correct program is loaded and that the console is properly configured before the automated sequence begins (Fig. 4).

**Table 1:** Set of instructions for the script that comprise a reaction sequence

Instruction	Parameters	Description
cmd_start	—	Run start sequence (*e.g.* close capsule holder and prime pump)
cmd_pump	(Pump-speed, pump-time, pump-volume)	Run the pump for a certain time with the defined pump speed and syringe volume
cmd_valve	(Valve-number, port-number)	Turn valve 1 or 2 to the desired port number
cmd_stir	(Stir-on/off, stir-speed)	Turn stirrer off or on with defined speed
cmd_heat	(Heater-number, temperature)	Set one of the heaters to defined temperature
cmd_delay	(Time)	Wait for defined period of time
cmd_end	—	Run end sequence (*e.g.* open capsule holder and reset instrument for next reaction)

After several prototypes, we developed a custom engineered capsule-holder assembly and a housing unit that allows the capsule to be easily exchanged without manual attachment of fluidic lines (Fig. 4). This key element ensures that reactions can be setup in seconds from prepackaged capsules, guaranteeing safe operation and cleaning of the console.

For designing the capsule, we first addressed the selection of materials considering existing supply chains. Polyethylene reservoirs were attractive as capsule compartments due to their low cost, commercial availability and resistance against commonly used solvents and reagents. Packing solid reagents in these reservoirs between two porous polyethylene frits facilitates storage and allows for the immediate deployment on the console. The intended chemistry applications required four separate manipulations, each of which was assigned to a compartment. Different compartment sizes were selected depending on the quantity of reagent required. A case was designed to combine the four compartments into a single capsule. On top of the capsule assembly a Viton seal ensures the tightness of the connections, after the insertion and automatic connection of the capsule inside the console.

**Scheme 1:**
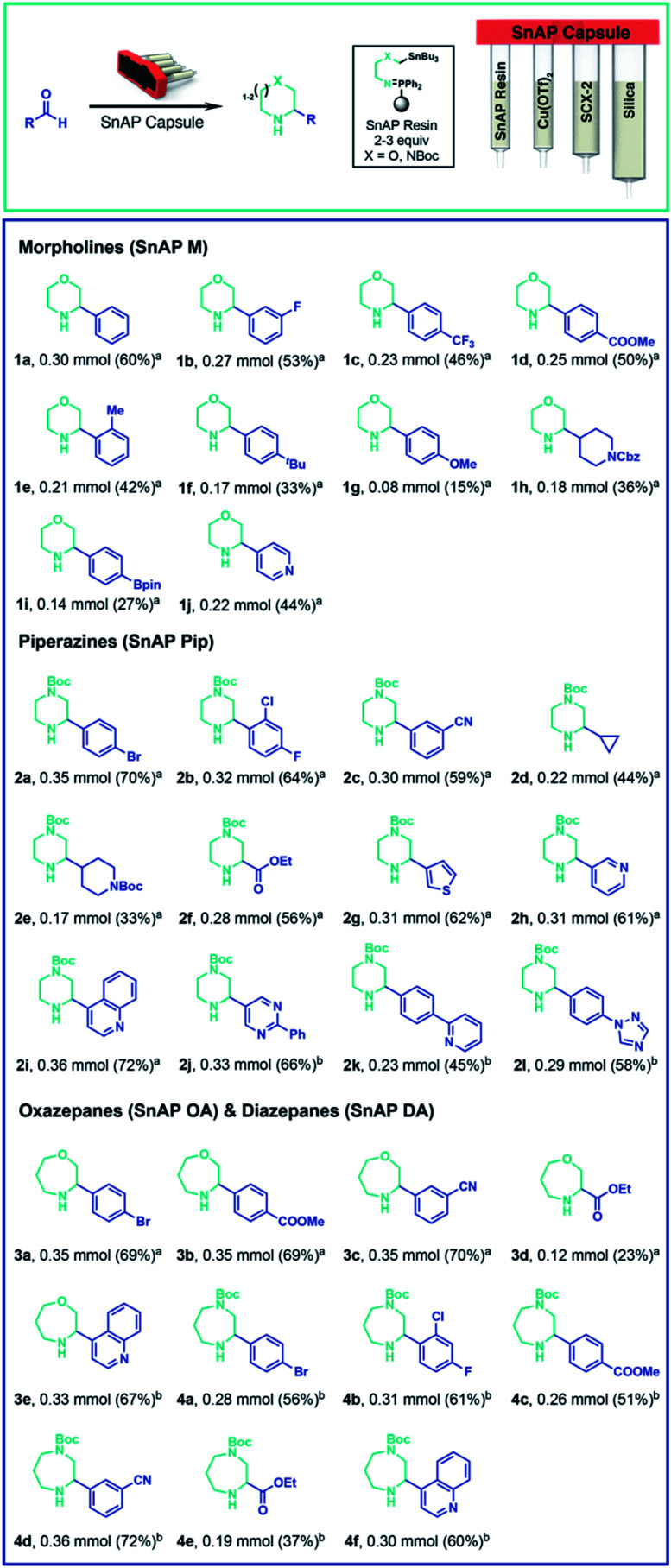
Automated formation of saturated N-heterocycles. A broad scope of aldehydes are transformed to various saturated N-heterocycle using SnAP reagent capsules. In most cases a purity of >90% was obtained. See ESI† for detailed evaluation of the reaction product after automated sequence (a) quantity of the desired product directly after the automated sequence, calculated from^1^ H-NMR using 0.25 mmol (0.5 equiv.) of mesitylene as internal standard is given (b) quantity of isolated product after column chromatography is given.

As a candidate reaction for complete automation, we selected the synthesis of substituted, saturated N-heterocycles from aldehydes using stannyl amine protocol (SnAP) chemistry,^20–22^ which is recognized as a versatile approach to substituted morpholines, piperazines, oxazepanes, and other attractive scaffolds. The chemistry proceeds with a broad substrate scope under consistent conditions (Cu(OTf)_2_, 2,6-lutidine, CH_2_Cl_2_/HFIP (1,1,1,3,3,3-hexafluoro-2-propanol)), but utilizes potentially toxic tin reagents and requires labor intensive steps, including weighing of the reagents, complexation of the copper and ligand, preparation of an imine intermediate, an intricate aqueous workup, and column chromatography. In providing a fully automated implementation of SnAP chemistry, we considered all the steps and dedicated one compartment of the capsule to a specific task: (1) imine formation of the SnAP reagent with the aldehyde, (2) copper-mediated cyclization using Cu(OTf)_2_/2,6-lutidine complex in HFIP, (3) workup to remove copper salts, and (4) purification using silica-based chromatography (Scheme 1). We planned a system for research scale (up to 0.5 mmol) operation, in which users need only to add the aldehyde starting material; all other reagents would be encapsulated into a sealed capsule; no reagents would need to be weighed, and the console would deliver the purified N-heterocyclic product with no further user involvement.

For imine formation, we generated solid-supported iminophosphoranes from polymeric triphenylphosphine and the corresponding SnAP azides^23^ and packed this material into the first compartment. Extensive testing identified the optimal conditions to be continuous circulation of a solution of the aldehyde in CH_2_Cl_2_ over 2–3 equivalents of the iminophosphorane while heating at 50 °C. The standard conditions for cyclization to the saturated N-heterocycles requires the addition of the imine to preformed Cu(OTf)_2_/2,6-lutidine complex in HFIP. This was best achieved by loading solid 2,6-lutidinium triflate and Cu(OTf)_2_ into the second compartment, separated by a polypropylene frit to ensure stability upon storage. Given the low solubility of Cu(OTf)_2_ in the reaction media, the imine solution was recirculated through the Cu(OTf)_2_/2,6-lutidinium triflate compartment to effect the cyclization and generate the N-heterocycles. The cyclization was complete within 3 hours when the compartment and the reaction vial were heated to 40 °C.

For the workup and purification of the N-heterocycles, we implemented a “catch & release” strategy. After cyclization, the console loaded the reaction mixture onto silica-gel to remove most of the copper salts and the filtrate was transferred onto silica-supported propylsulfonic acid (SCX-2, 4 equivalents, compartments 3 and 4) which protonated the basic N-heterocycle and retained it on the support. This material also acted as a metal scavenger and successfully removed the remaining copper salts. Following washing of the resin with MeOH to expel the non-basic byproducts, including any unreacted aldehyde and most of the tin species, the desired product was released in high purity (>90%) using a 2.5 M *N*,*N*-diisopropylamine solution in THF in the majority of examples. Typically, only a minimal amount of tin residue (<5%) was observed (^1^H NMR analysis).

Using this instrument and the appropriate capsules, we performed the automated synthesis of morpholines, oxazepanes, piperazines and diazepanes using aryl, heteroaryl and alkyl aldehydes (Scheme 1). The corresponding substituted N-heterocycles were obtained as THF solutions, which were evaporated to afford the products in good yield and high purity (≥90%). In general, aryl aldehydes bearing electron-withdrawing groups, such as halides, fluorinated alkyls, esters and nitriles, gave good yields. Electron rich aldehydes – which are slower to form the imine intermediate – or alkyl aldehydes – which are prone to enamine formation – gave the cyclized products with lower yield, but still in high purity.

**Scheme 2:**
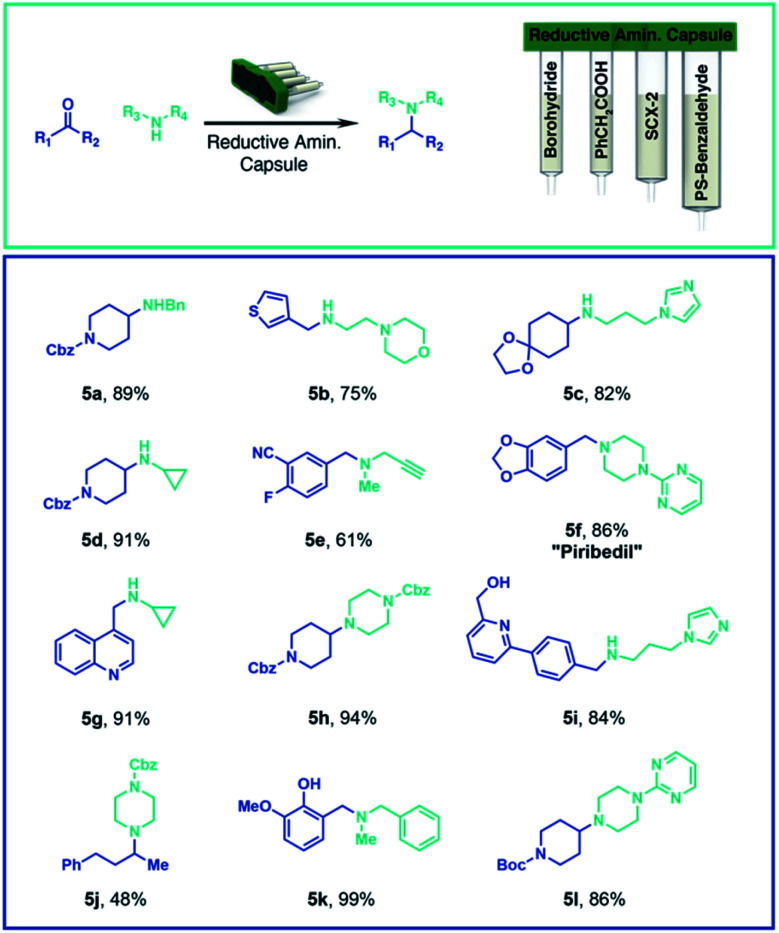
Reductive amination process and products. Aldehydes and ketones are coupled with primary or secondary amines with a capsule for reductive amination. Yields of the reaction product (purity >95%) directly after the automated synthesis are given.

By changing only the capsule contents and software instructions, the same console can be used for other common organic reactions. For example, we implemented a capsule-based approach to reductive amination by loading one compartment with solid supported cyanoborohydride, and a second with SCX-2, to effect the purification using the same solid-phase extraction method described for the SnAP chemistry. In this case, the user selects both coupling partners (up to a maximum of 0.5 mmol) and places them in the reaction vial together with the reaction solvent. Optimization of the reaction conditions identified CH_2_Cl_2_/HFIP (4 : 1) as the preferred solvent system for the broadest range of test substrates. For reductive aminations between secondary amines and ketones, toluene at 40 °C proved to be the preferred solvent and phenylacetic acid – loaded into a third compartment – was used as an activator. A fourth compartment was charged with solid supported benzaldehyde, used as a scavenger for any unreacted primary amine. Depending on the choice of carbonyl (aldehyde *vs.* ketone) and amine (1° or 2°) compounds different preprogrammed reaction sequences where implemented; the user selects the appropriate program on the touch display. A variety of compounds were synthesized with the optimized conditions, leading in most of the cases to high yields and purity within 3 to 5 hours (Scheme 2).

**Scheme 3:**
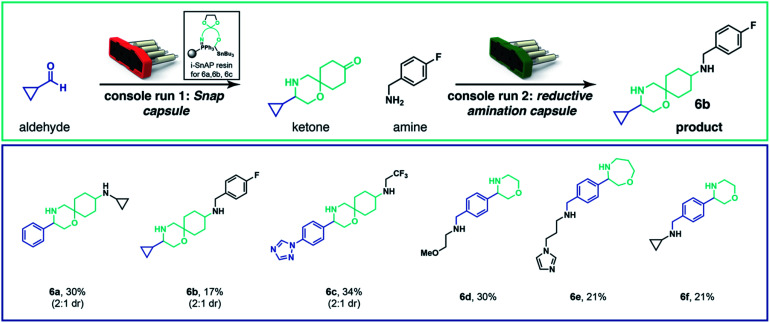
Automated, multistep, capsule-based organic synthesis. The SnAP capsule and reductive amination capsule can be combined in two consecutive machine runs to generate more complex structures. Yields were calculated from isolated material after purification by chromatography; diastereomers -where present-were resolved in a later step by preparative HPLC for characterization; see ESI† for details.

To execute fully automated, multistep organic synthesis, we employed reagents containing a protected carbonyl and loaded them into capsules.^24^ By simply adapting the script and SnAP capsules to include a deprotection step on a polystyrene supported acid we could telescope the N-heterocycle formation and carbonyl deprotection. After the cyclization and deprotection were complete, the solvent was removed, an amine was added, the vial placed on the same console, and the script and capsule for the reductive amination were loaded. The fully automated sequence gave **6a** in 30% overall yield as a mixture of two diastereomers. Several other attractive products were prepared (**6b** and **6c**) by this automated sequence (Scheme 3). The protected carbonyl can be included in either the SnAP reagent (**6a**, **6b**, **6c**) or the aldehyde substrate (**6d**, **6e** and **6f**).

Capsule designs and scripts for a number of other organic transformations have been developed^25^ and console upgrades, including the ability to employ low temperature and photochemical conditions, will make this approach suitable for many other reaction classes. Ongoing innovations in facilitating the execution of organic synthesis, such as reagents coated onto glass beads^26^ and immobilized catalysts and reagents,^27^ will facilitate the implementation of other important reactions onto this automated, consoled-based format.

We are aware that not every type or reaction encountered in organic synthesis can be implemented on such an instrument. The aim of this tool is to increase efficiency in the laboratory by automating routine steps, allowing more time to spend on challenging reactions that are harder or impossible to automate. The console also greatly facilitates standardization of reaction conditions and reagents, enhancing reproducibility and reliability. With some innovation, we believe that capsules for reaction classes can be developed and innovations in organic methods will obviate some of the more dangerous and unpleasant practices that cannot be easily implemented.

## Conclusions

The desire for automation in medicinal chemistry laboratories is easily observed with the widespread adoption of automated purification instruments, which use pre-packed silica-gel cartridges for the purification of organic compounds and require little user involvement. Our console/capsule/script approach brings the same level of convenience, reliability, and automation to the setup, execution, workup, and purification of organic reactions, using a compact, user-friendly setup. We envision instruments such as the ones described here will serve as standardized platforms upon which automated chemistry will be developed. To make an analogy, the console serves as the computer and operating system while the capsules are the applications. Organic chemists will become the developers who package their methods into integrated, user-friendly packages that can be used immediately in a safe and convenient fashion.

## Author contributions

J. W. B., B. M. W., and P. L. N. conceived of the idea. B. M. W. built the console and designed the software. T. J., K.-Y. C. implemented and developed the SnAP capsules. S. B. developed the reductive amination. A. E. M. optimized and performed multi-step reactions. F. S. designed and synthesized the iSnAP reagent. All authors contributed to data acquisition and analysis. J. W. B, B. M. W., A. E. M and P. L. N. wrote the manuscript.

## Conflicts of interest

This technology was developed with the support of funding from the Swiss Commission for Technology Innovation, the ETH Pioneer Fellowships, and the European Research Council and licensed to the ETH spinoff company Synple Chem AG. B. M. W., P. L. N., K.-Y. C. and J. W. B. are listed as inventors of a patent (WO Pat., WO2017121724A1, 2016) related to this technology. B. M. W., P. L. N., T. J. and J. W. B. are co-founders of Synple Chem AG.

## Supplementary Material

SC-012-D1SC01048D-s001

SC-012-D1SC01048D-s002
