# Supplementary material for: An integrated console for capsule-based, automated organic synthesis
Source: Chem Sci. 2021 Apr 13;12(20):6977–82. doi: 10.1039/d1sc01048d (PMC8153237; doi:10.1039/d1sc01048d)
Supplement: SC-012-D1SC01048D-s001 [file SC-012-D1SC01048D-s001.pdf]

Electronic Supplementary Material (ESI) for Chemical Science.
This journal is © The Royal Society of Chemistry 2021

Supporting Information

An Integrated Console for Capsule-Based, Fully Automated Organic Synthesis

Tuo Jiang,[ab] Samuele Bordi,[a] Angus E. McMillan,[a] Kuang-Yen Chen,[a] Fumito Saito,[a] Paula L. Nichols,[ab] Benedikt M. Wanner,*[ab] and Jeffrey W. Bode*[a]

[a] Laboratory for Organic Chemistry, Department of Chemistry and Applied Biosciences, ETH Zürich, 8093 Zürich, Switzerland

[b] Synple Chem AG, 8093 Zürich, Switzerland

wanner@synplechem.com

bode@org.chem.ethz.ch

This PDF file includes:

Materials and Methods
Supplementary Text
References

Table of Contents:

1. General Remarks 3

2. Console Operation and Script 4

2.1 Console commands 4

3. Synthesis of Azides and Resins 7

3.1 Synthetic Route towards SnAP Morpholine Azide (M4) 7

3.2 Synthetic Route towards SnAP Piperazine Azide (P4) 8

3.3 Synthetic Route towards SnAP 1,4-Oxazepane Azide (OA3) 9

3.4 Synthetic Route towards SnAP Diazepane Azide (DA4) 10

3.5 Synthetic Route towards SnAP (2-spiro-(4-EG-cy)-M) Azide (SM3) 11

3.6 Loading of the Resin 12

4. Automated Heterocycle Formation on the Console 12

4.1 General Procedure A for the Automated N-Heterocycle Synthesis on the Console 12

4.2 Characterization of Reaction Products 14

4.2.1 Morpholine products 14

4.2.2 Piperazine products 17

4.2.3 Oxazepane products 22

4.2.4 Diazepane products 23

5. Automated Reductive Amination on the Console 26

5.1 General Procedure B for the Automated Reductive Amination on the Console 26

5.2 Characterization of Reaction Products 27

6. Combination of Heterocycle Formation and Reductive Amination on the Console 31

6.1 General Procedure C 31

5.2 Characterization of Reaction Products 33

7. NMR Spetra of product compounds 39

8. Additional References 122

1. General Remarks

Unless otherwise noted, all reactions were performed under N_2 with anhydrous conditions. All reagents were used as received from commercial suppliers. Anhydrous THF and DMF were dried by passing through activated alumina under nitrogen. Reactions were monitored by thin layer chromatography (TLC) on Merck precoated aluminum-backed silica gel 60 F_{254} plates with UV at 254 nm, or by subjecting to NMR analysis.

TLC plates were stained using iodine, $KMnO_4$ or ninhydrin solutions. Flash chromatography purification was performed on Silicycle Silica Flash F60 (230–400 Mesh) silica gel using a forced flow of eluent at 0.2-0.3 bar.

NMR spectra were recorded on *Bruker* Avance III 400 MHz at 400 or 500 MHz (H) and at 100, 125 MHz (C), respectively, using $CDCl_3$ as the solvent unless indicated otherwise. Chemical shifts (δ) are reported in ppm, using the residual solvent peak in $CDCl_3$ (H: δ = 7.26 ppm and C: δ = 77.16 ppm) as the internal standard. All ^{13}C spectra were measured with complete proton decoupling. NMR coupling constants (J) are reported in Hertz (Hz), and splitting patterns are indicated as follows: br, broad; s, singlet; d, doublet; dd, doublet of doublet; ddd, doublet of doublet of doublet; dt, doublet of triplet; t, triplet; q, quartet; quint; quintet; sext, sextet; m, multiplet.

High resolution mass spectra were measured by the Mass Spectrometry Service Facility of Molecular and Biomolecular Analysis Service MoBiAS, Laboratory of Organic Chemistry at ETH Zurich on a *Bruker* Daltonics maXis for ESI-Q-TOF spectrometer (ESI-MS) or on a *Bruker* solariX (9.4T magnet) equipped with a dual ESI/MALDI-FT-ICR source using *trans*-2-[3-(4-*tert*-butylphenyl)-2-methyl-2-propenylidene]malononitrile (DCTB) as matrix (MALDI-MS).

Console used reagents and solvents

Polystyrene-supported PPh_3 (1.7 mmol/g, 100-200 mesh, 2% cross-linked with divinylbenzene) was used as received. 4Å molecular sieves (powder) were dried at 110 °C under high vacuum for 16 hours (ca. 0.1 mbar) and stored in a desiccator. $Cu(OTf)_2$ was dried at 110 °C under high vacuum for 2 hours (ca. 0.1 mbar) and stored in a desiccator. 2,6-Lutidinium triflate was prepared following the literature[1] and stored in a desiccator. Anhydrous CH_2Cl_2 was purchased over 4Å molecular sieves (> 99.8%, stabilized with ca. 50 ppm amylene) and used as received. HFIP (1,1,1,3,3,3-hexafluoroisopropanol) was distilled over activated 3Å molecular sieves (beads) and stored over activated 3Å molecular sieves (beads). The purity of other console-used chemicals is listed as follows: MeOH (> 99.9%), DIPA (*N*,*N*-diisopropylamine, ≥ 99.5%) and THF (≥ 99.5%, stabilized with ca. 250 ppm BHT).

1) Curley, J. J.; Bergman, R. G.; Tilley, T. D.: Preparation and physical properties of early-late heterobimetallic compounds featuring Ir–M bonds (M = Ti, Zr, Hf). *Dalton Trans.* **2012**, *41*, 192–200.

2. Console Operation and Script

2.1 Console commands

To carry out a reaction the console can operate several modules that include: two 8-port rotary valves, two syringe pumps, a magnetic stirrer, a capsule heater and a sample vial heater. These instructions to operate the modules and in which order and time are listed in a table. The console will read each line step by step and executes the operations of the modules. One set of instructions consist of a command and optionally several parameters:

Command set for automated console:

Command	Parameters	Description
Cmd_start	–	Run start sequence (e.g. close cartridge holder and prime pump)
Cmd_pump	(pump-speed, pump-time, pump-volume)	Runs the pump for a certain time with the defined pump speed and syringe volume. If no pump-time is given (NOP) the console will only dose the define volume.
Cmd_valve	(valve-number, port-number)	Turn valve 1 or 2 to the desired port number
Cmd_stir	(stir-on/off, stir-speed)	Turn stirrer off or on with defined speed
Cmd_heat	(heater-number, temperature	Set one of the heaters to defined temperature
Cmd_delay	(time)	Wait for defined period of time
Cmd_end	–	Run end sequence (e.g. open cartridge holder and reset instrument for next reaction

The script example below shows the script the machine reads (Command and parameter) for the generation of the N-Heterocycle products. For better readability the sequence is split into different section and comments where added:

Command	Parameter	Comment
Start sequence		
Cmd_start		Initiate sequence and initialize console
Initiate reaction		
Cmd_valve	0,DCM	Turn valve 1 to CH2Cl2 port
Cmd_valve	1,CART1	Turn valve 2 to port with cartridge compartment 1
Cmd_pump	200,NOP,2700	Draw 4.5mL solvent
Cmd_pump	20,NOP,2700	Dispense 4.5mL solvent
Cmd_stir	1,15	Turn on stirrer with speed “15”
Cmd_heat	0,35	Heat sample holder to 35°C
Cmd_delay	60	Wait for 1 min
Imine Formation		
Cmd_valve	0,CIRC	Turn valve 1 to sample vial tubing
Cmd_valve	1,CART1	Turn valve 2 to port with cartridge compartment 1
Cmd_heat	1,50	Heat cartridge holder to 50°C
Cmd_pump	10,5400,300	Pump continuously with 0.1mL/min for 90min
Cmd_heat	1,40	Heat cartridge holder to 40°C
Cmd_pump	10,900,300	Pump continuously with 0.1mL/min for 15min
Prepare next step		
Cmd_valve	0,BALANCE	Turn valve 1 to sample vial pressure balance tubing
Cmd_valve	1,WASTE	Turn valve 2 to waste port
Cmd_pump	100,NOP,3000	Draw 5.0mL
Cmd_pump	100,NOP,0	Dispense 5.0mL
Cmd_valve	0,DCM	Turn valve 1 to DCM port

Cmd_valve	1,WASTE	Turn valve 2 to waste port
Cmd_pump	200,NOP,1800	Draw 3.0mL
Cmd_pump	300,NOP,0	Dispense 3.0mL
Cmd_valve	1,CART1	Turn valve 2 to port with cartridge compartment 1
Cmd_pump	200,NOP,2100	Draw 3.5mL
Cmd_pump	50,NOP,0	Dispense 3.5mL
Cmd_valve	0,BALANCE	Turn valve 1 to sample vial pressure balance tubing
Cmd_pump	200,NOP,2100	Draw 3.5mL
Cmd_pump	50,NOP,0	Dispense 3.5mL
Cmd_valve	0,HFIP	Turn valve 1 to HFIP port
Cmd_valve	1,WASTE	Turn valve 2 to waste port
Cmd_pump	200,NOP,1800	Draw 3.0mL
Cmd_pump	300,NOP,0	Dispense 3.0mL
Cmd_valve	1,CART2	Turn valve 2 to port with cartridge compartment 2
Cmd_pump	200,NOP,1200	Draw 2.0mL
Cmd_pump	20,NOP,0	Dispense 2.0mL
Cmd_heat	0,37	Heat sample holder to 37°C
Cyclization reaction		
Cmd_valve	0,CIRC	Turn valve 1 to sample vial tubing
Cmd_pump	50,300,600	Pump continuously with 0.5mL/min for 5min
Cmd_pump	10,4200,600	Pump continuously with 0.1mL/min for 70min
Prepare next step		
Cmd_heat	0,0	Turn off sample holder heater
Cmd_heat	1,0	Turn off cartridge holder heater
Cmd_valve	0,DCM	Turn valve 1 to DCM port
Cmd_valve	1,WASTE	Turn valve 2 to waste port
Cmd_pump	200,NOP,1800	Draw 3.0mL
Cmd_pump	300,NOP,0	Dispense 3.0mL
Cmd_valve	1,CART2	Turn valve 2 to port with cartridge compartment 2
Cmd_pump	200,NOP,1200	Draw 2.0mL
Cmd_pump	50,NOP,0	Dispense 2.0mL
Cmd_valve	0,BALANCE	Turn valve 1 to sample vial pressure balance tubing
Cmd_pump	200,NOP,2100	Draw 3.5mL
Cmd_pump	50,NOP,0	Dispense 3.5mL
Silica purification		
Cmd_valve	0,CIRC	Turn valve 1 to sample vial tubing
Cmd_valve	1,CART3	Turn valve 2 to port with cartridge compartment 3
Cmd_pump	100,NOP,8400	Draw 14.0mL
Cmd_pump	20,NOP,0	Dispense 14.0mL
Cmd_valve	0,DCM	Turn valve 1 to DCM port
Cmd_pump	200,NOP,2400	Draw 4.0mL
Cmd_pump	20,NOP,0	Dispense 4.0mL
Cmd_valve	0,BALANCE	Turn valve 1 to sample vial pressure balance tubing
Cmd_pump	100,NOP,9000	Draw 15.0mL
Cmd_pump	100,NOP,0	Dispense 15.0mL
SCX purification		
Cmd_valve	0,CIRC	Turn valve 1 to sample vial tubing
Cmd_valve	1,CART4	Turn valve 2 to port with cartridge compartment 4
Cmd_pump	100,NOP,9600	Draw 16.0mL
Cmd_pump	20,NOP,0	Dispense 16.0mL
Cmd_valve	0,DCM	Turn valve 1 to DCM port
Cmd_pump	200,NOP,2400	Draw 4.0mL
Cmd_pump	50,NOP,0	Dispense 4.0mL

Cmd_valve	0,BALANCE	Turn valve 1 to sample vial pressure balance tubing
Cmd_pump	100,NOP,9000	Draw 15.0mL
Cmd_pump	100,NOP,0	Dispense 15.0mL
Cmd_valve	0,CIRC	Turn valve 1 to sample vial tubing
Cmd_valve	1,WASTE	Turn valve 2 to waste port
Cmd_pump	100,NOP,7200	Draw 12.0mL
Cmd_pump	300,NOP,0	Dispense 12.0mL
Cmd_pump	100,NOP,7200	Draw 12.0mL
Cmd_pump	300,NOP,0	Dispense 12.0mL
Cmd_valve	0,MEOH	Turn valve 1 to MeOH port
Cmd_pump	200,NOP,4200	Draw 7.0mL
Cmd_pump	300,NOP,0	Dispense 7.0mL
Cmd_valve	1,CART4	Turn valve 2 to port with cartridge compartment 4
Cmd_pump	200,NOP,7200	Draw 12.0mL
Cmd_pump	50,NOP,0	Dispense 12.0mL
Cmd_valve	0,BALANCE	Turn valve 1 to sample vial pressure balance tubing
Cmd_pump	100,NOP,9000	Draw 15.0mL
Cmd_pump	100,NOP,0	Dispense 15.0mL
Cmd_valve	0,CIRC	Turn valve 1 to sample vial tubing
Cmd_valve	1,WASTE	Turn valve 2 to waste port
Cmd_pump	100,NOP,9000	Draw 15.0mL
Cmd_pump	300,NOP,0	Dispense 15.0mL
Cmd_valve	0,DCM	Turn valve 1 to DCM port
Cmd_pump	200,NOP,2400	Draw 4.0mL
Cmd_pump	300,NOP,0	Dispense 4.0mL
Cmd_valve	0,DIPA	Turn valve 1 to THF/DIPA port
Cmd_pump	200,NOP,3600	Draw 6.0mL
Cmd_pump	300,NOP,0	Dispense 6.0mL
Cmd_valve	1,CART4	Turn valve 2 to port with cartridge compartment 4
Cmd_pump	200,NOP,6000	Draw 10.0mL
Cmd_pump	50,NOP,0	Dispense 10.0mL
Cmd_pump	200,NOP,6000	Draw 10.0mL
Cmd_pump	50,NOP,0	Dispense 10.0mL
Finalize and wash pump		
Cmd_stir	0,0	Turn of stirrer
Cmd_valve	0,MEOH	Turn valve 1 to MeOH port
Cmd_valve	1,WASTE	Turn valve 2 to waste port
Cmd_pump	200,60,600	Pump continuously with 2.0mL/min for 1min
Cmd_valve	0,DCM	Turn valve 1 to DCM port
Cmd_pump	200,60,300	Pump continuously with 2.0mL/min for 1min
Cmd_end		The end of the script terminates the process and the consoles returns to idle.

3. Synthesis of Azides and Resins

3.1 Synthetic Route towards SnAP Morpholine Azide (M4)

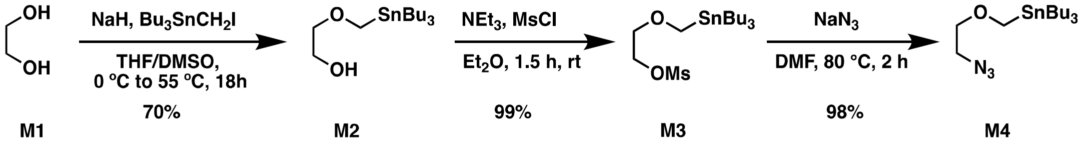

2-((Tributylstannyl)methoxy)ethan-1-ol (M2)

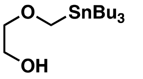

A suspension of NaH (2.12 g, 52.9 mmol, 1.20 equiv, 60% in mineral oil) in THF/DMSO (10:1, 150 mL) was cooled to 0 °C followed by the dropwise addition of ethylene glycol (7.40 mL, 132 mmol, 3.00 equiv). The suspension was allowed to warm to room temperature. After 1 h, the mixture was cooled to 0 °C followed by the dropwise addition of tributyl(iodomethyl)stannane (19.0 g, 44.1 mmol, 1.00 equiv). The reaction mixture was allowed to warm to room temperature and heated at 55 °C for 18 h. The reaction was quenched carefully with deionized H_2O (30 mL) and phase-separated. The aqueous phase was extracted with MTBE (100 mL × 2). The combined organic phase was washed with deionized H_2O (50 mL × 2), brine (50 mL), dried over Na_2SO_4 and concentrated to dryness. The residue was purified by flash column chromatography (hexanes/EtOAc; v/v = 10/1) to afford 2-((tributylstannyl)methoxy)ethan-1-ol (**M2**) as a colorless liquid (11.2 g, 70%).

^{1}H NMR ($CDCl_3$, 400 MHz): δ 3.76 (s, $J(^{117/119}\text{Sn-}^1\text{H})$ = 7.3 Hz, 2H), 3.71–3.66 (m, 2H), 3.46–3.43 (m, 2H), 1.91 (t, J = 6.2 Hz, 1H), 1.57–1.48 (m, 6H), 1.34–1.26 (m, 6H), 0.94–0.87 (m, 15H).

These spectral characteristics were identical to those previously reported.[2]

2-((Tributylstannyl)methoxy)ethyl methanesulfonate (M3)

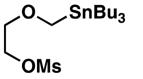

To a solution of 2-((tributylstannyl)methoxy)ethan-1-ol (**M2**) (11.8 g, 32.3 mmol, 1.00 equiv) and Et_3N (9.00 mL, 64.6 mmol, 2.00 equiv) in Et_2O (100 mL) was added MsCl (2.75 mL, 35.5 mmol, 1.10 equiv) dropwise. The mixture was stirred at room temperature for 1.5 h. The reaction mixture was quenched with deionized H_2O (50 mL) and phase-separated. The aqueous phase was extracted with MTBE (100 mL × 2). The combined organic phase was washed with deionized H_2O (50 mL × 2), brine (50 mL), dried over Na_2SO_4 and concentrated to dryness. The crude 2-((Tributylstannyl)methoxy)ethyl methanesulfonate (**M3**) was obtained as light yellow liquid (14.3 g, 99%) and used directly in the next step.

^{1}H NMR ($CDCl_3$, 400 MHz): δ 4.35–4.32 (m, 2H), 3.74 (s, $J(^{117/119}\text{Sn-}^1\text{H})$ = 7.8 Hz, 2H), 3.62–2.59 (m, 2H), 3.03 (s, 3H), 1.55–1.45 (m, 6H), 1.36–1.24 (m, 6H), 0.93–0.87 (m, 15H).

These spectral characteristics were identical to those previously reported.[2]

((2-Azidoethoxy)methyl)tributylstannane (M4)

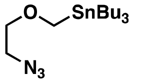

To a solution of 2-((tributylstannyl)methoxy)ethyl methanesulfonate (**M3**) (4.67 g, 10.5 mmol, 1.00 equiv) in DMF (21 mL) was added NaN_3 (1.02 g, 15.8 mmol, 1.50 equiv) in one-portion. The suspension was stirred vigorously at 80 °C for 2 h. After cooling to room temperature, the crude mixture was quenched with deionized H_2O (60 mL) and extracted with MTBE (80 mL × 3). The combined organic phase was washed with deionized H_2O (20 mL × 5), brine (10 mL), dried over Na_2SO_4 and concentrated to dryness. The residue was purified by flash column

2) Luescher, M. U.; Vo, C.-V T.; Bode, J. W.: SnAP Reagents for the Synthesis of Piperazines and Morpholines. *Org. Lett.* **2014,** *16*, 1236–1239.

chromatography (hexanes/EtOAc; v/v = 50/1) to afford ((2-azidoethoxy)methyl)tributylstannane (**M4**) as a colorless liquid (4.01 g, 98%).

^{1}H NMR ($CDCl_3$, 400 MHz): *δ* 3.74 (s, *J*($^{117/119}$Sn-^{1}H) = 7.8 Hz, 2H), 3.54–3.51 (m, 2H), 3.32–3.30 (m, 2H), 1.51–1.47 (m, 6H), 1.35–1.26 (m, 6H), 0.94–0.88 (m, 15H).

^{13}C NMR ($CDCl_3$, 100 MHz): *δ* 74.5, 62.7, 50.8, 29.3, 27.4, 13.9, 9.1.

These spectral characteristics were identical to those previously reported.[3]

3.2 Synthetic Route towards SnAP Piperazine Azide (P4)

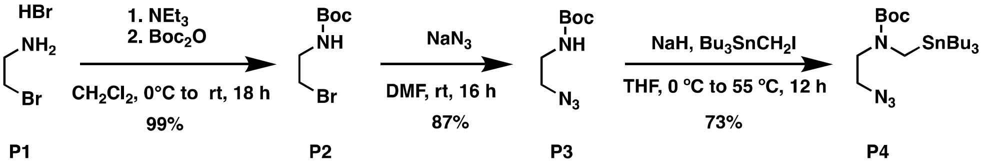

***tert*-Butyl (2-bromoethyl)carbamate (P2)**

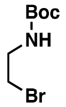

A flame dried 25 mL flask was charged with Boc_2O (533 mg, 2.44 mmol, 1.00 equiv) in CH_2Cl_2 (13 mL) at 0 °C. Commercially available 2-bromoethylamin hydrobromide (556 mg, 2.71 mmol, 1.11 equiv) was added in one portion at 0 °C, followed by the dropwise addition of NEt_3 (508 µL, 3.66 mmol, 1.50 equiv) over 10 min. The reaction mixture was allowed to warm to rt and stirred for 18 h. The colorless reaction mixture was diluted with CH_2Cl_2 (100 mL) and washed with sat aq NH_4Cl (2 × 10 mL), sat aq $NaHCO_3$ (2 × 10 mL) and brine (2 × 10 mL). The combined organic layers were dried over anhydrous Na_2SO_4, filtered and concentrated under reduced pressure to obtain pure **P1** in quantitative yield (531 mg).

^{1}H NMR ($CDCl_3$, 400 MHz): *δ* 4.94 (br s, NH), 3.58–3.49 (m, 2H), 3.45 (t, *J* = 5.5 Hz, 2H), 1.45 (s, 9H).

^{13}C NMR ($CDCl_3$, 100 MHz): *δ* 155.7, 79.9, 42.5, 33.0, 28.5.

These spectral characteristics were identical to those previously reported.[4]

***tert*-Butyl (2-azidoethyl)carbamate (P3)**

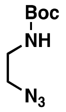

To a DMF (62 mL) solution of *tert*-butyl (2-bromoethyl)carbamate (**P2**) (3.50 g, 15.6 mmol, 1.00 equiv) was added NaN_3 (2.03 g, 31.2 mmol, 2.00 equiv) in one-portion. The mixture was stirred for 16 h at room temperature before being quenched by H_2O (100 mL). The mixture was extracted with MTBE (100 mL × 2) and the combined organic phase was washed with H_2O (60 mL × 4) and brine (60 mL), dried over Na_2SO_4 and concentrated. The crude product (2.68 g, 87%) was pure by NMR and used directly for the next step.

^{1}H NMR ($CDCl_3$, 400 MHz): *δ* 1.45 (s, 9H, (CH3)3), 3.31 (m, 2H, CH2), 3.42 (m, 2H, CH2), 4.98 (broad s, 1H, NH).

^{13}C NMR ($CDCl_3$, 100 MHz): *δ* 28.2, 40.0, 51.1, 79.6, 155.7.

These spectral characteristics were identical to those previously reported.[5]

---

3) Siau, W.-Y.; Bode, J. W.: One Step Synthesis of Saturated Spirocyclic N-Heterocycles with SnAP Reagents and Ketones. *J. Am. Chem. Soc.* **2014**, *136*, 17726–17729.

4) van Dijk, M.; van Nostrum, C. F.; Hennink, W. E.; Rijkers, D. T. S.; Liskamp, R. M. J.: Synthesis and characterization of enzymatically biodegradable PEG and peptide-based hydrogels prepared by click chemistry. *Biomacromolecules* **2010**,*11*, 1608.

5) Hyungmi, K.; Mi Kyoung, K.; Hyunah, C.; Youhoon, C.: Novel JAK1-selective benzimidazole inhibitors with enhanced membrane permeability. *Bioorganic Med. Chem. Lett.* **2016**, *26*, 3213–3215.

tert-Butyl (2-azidoethyl)((tributylstannyl)methyl)carbamate (P4)

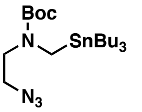

Sodium hydride (60% suspension in mineral oil, 552 mg 13.8 mmol, 1.20 equiv) was washed with n-pentane and suspended in THF. The suspension was cooled to 0 °C and azido compound **P3** (4.29 g, 23.0 mmol, 2.00 equiv) in THF (0.5 M) was added dropwise over 10 min. The resulting suspension was warmed to 23 °C and allowed to stir for 1 h. After re-immersion of the reaction mixture in the ice-bath, tributyl(iodomethyl)stannane (5.00 g, 11.5 mmol, 1.00 equiv) was added dropwise over 10 min. The resulting mixture was allowed to stir at 55 °C for overnight before it was cooled and quenched carefully by H_2O (15 mL). The layers were separated the aqueous layer was extracted with EtOAc (3 × 15 mL). The combined organic layer was washed twice with H_2O (15 mL each), brine (20 mL), dried over Na_2SO_4, filtered and concentrated under reduced pressure. Purification by flash column chromatography (hexanes/EtOAc; v/v = 1/0 → 4/1) afforded the desired product as a colorless liquid (4.12 g, 73%).

^{1}H NMR ($CDCl_3$, 400 MHz): δ 3.49–3.25 (m, 4H), 3.12 (s, 0.64H), 2.83 (s, 1.24H), 1.54–1.39 (m, 15H), 1.29 (h, *J* = 7.2 Hz, 6H), 0.87 (dt, *J* = 14.4, 7.6 Hz, 15H).

^{13}C NMR ($CDCl_3$, 100 MHz): δ 155.1, 79.9, 79.5, 49.5, 49.5, 49.4, 48.6, 34.3, 34.2, 29.1, 28.5, 28.4, 27.4, 13.7, 10.5, 9.6.

HRMS (ESI) m/z for $C_{20}H_{42}N_4O_2Sn$ $[M+H]^+$ calcd 491.2406, found 491.2402.

IR (v/cm^{-1}, thin film) 2978, 1696, 1366, 1251.

3.3 Synthetic Route towards SnAP 1,4-Oxazepane Azide (OA3)

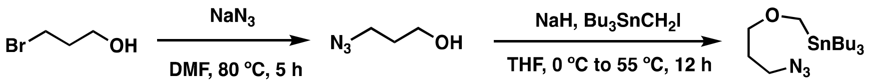

OA1 57% OA2 70% OA3

3-Azido-1-propanol (OA2)

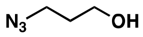

3-bromo-1-propanol (40.0 g, 288 mmol, 1.00 equiv) was allowed to stir in DMF (72 mL). Sodium azide (26.7 g, 410 mmol, 1.40 equiv) was added in portions and the resulting suspension was allowed to stir for 30 min. The reaction mixture was heated to 80 °C for 5 h. Et_2O (100 mL) and water (75 mL) were added to the mixture. The aqueous layer was extracted with Et_2O (2 × 100 mL) and the combined ethereal layers were washed with water (3 × 75 mL), dried over Na_2SO_4 and filtered. The solution was concentrated *in vacuo* to afford the desired azide **OA2** as colorless liquid (16.6 g, 57%).

^{1}H NMR ($CDCl_3$, 400 MHz): δ 3.73 (t, *J* = 6.2 Hz, 2H), 3.48 (t, *J* = 6.7 Hz, 2H), 1.82 (m, 2H).

These spectral characteristics were identical to those previously reported.[6]

((3-Azidopropoxy)methyl)tributylstannane (OA3)

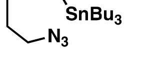

Sodium hydride (2.10 g of a 60% suspension in mineral oil, 51.9 mmol, 0.750 equiv) was washed with n-hexane and suspended in THF (45 mL). The suspension was cooled to 0 °C and azide **OA2** (6.90 g, 69.2 mmol, 1.00 equiv) was added dropwise over 10 min. The resulting suspension was allowed to stir at rt until no more gas formation was observed. The suspension was cooled to 0 °C and tributyl(iodomethyl)stannane (14.9 g, 34.6 mmol, 0.500 equiv) was added dropwise over 10 min. The mixture was allowed to stir at 55 °C for 16 h. The reaction mixture was allowed to cool to rt and treated with water (15 mL). The aqueous layer was extracted with Et_2O (3 × 15 mL) and the combined organic layers were washed with water (2 × 15 mL), brine (20 mL), dried over Na_2SO_4, filtered and

6) Jin, J.; Wu, D.; Sun, P.; Liu, L.; Zhao, H.: Amphiphilic triblock copolymer bioconjugates with biotin groups at the junction points: synthesis, self-assembly, and bioactivity. *Macromolecules,* **2011**, *44*, 2016–2024.

concentrated *in vacuo*. The crude product was purified by column chromatography on silica gel (hexanes/EtOAc; v/v = 1/0 → 6/1) to afford the desired the SnAP reagent **OA3** (9.8 g, 70%).

^{1}H NMR ($CDCl_3$, 400 MHz): δ 3.70 (s, J($^{117/119}$Sn-^{1}H) = 14.8 Hz, 2H), 3.39 (d, J = 5.9 Hz, 2H), 3.35 (d, J = 6.8 Hz, 2H), 1.81 (tt, J = 6.8, 5.9 Hz, 2H), 1.56–1.45 (m, 6H), 1.36–1.25 (m, 6H), 0.99–0.81 (m, 15H).

^{13}C NMR ($CDCl_3$, 100 MHz): δ 72.13, 62.28, 48.75, 29.39 (3C), 29.28, 27.44 (3C), 13.85 (3C), 9.15 (3C).

HRMS (ESI) m/z for $C_{16}H_{36}N_3OSn$ $[M+H]^+$ calcd 406.1875, found 406.1880.

IR (ν/cm^{-1}, neat) 2956, 2925, 2871, 2854, 2096, 1463, 1377, 1340, 1293, 1260, 1094, 1021, 874, 725.

3.4 Synthetic Route towards SnAP Diazepane Azide (DA4)

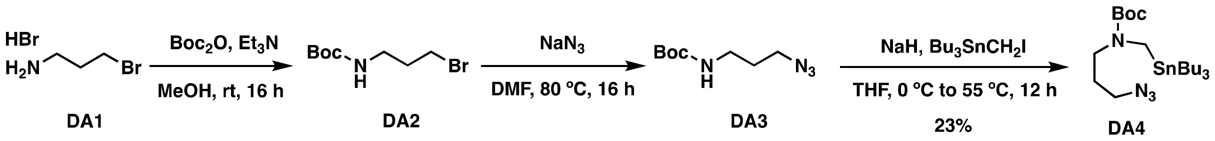

***tert*-Butyl (3-bromopropyl)carbamate (DA2)**

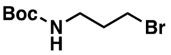

3-Bromopropylamine hydrobromide (**DA1**) (24.8 g, 113 mmol, 1.00 equiv) in MeOH (95 mL) was treated with NEt_3 (14.0 mL, 100 mmol) and stirred for 5 min. The mixture was cooled to 0 °C and Boc_2O (46.6 g, 213 mmol, 1.90 equiv) was added. The mixture was allowed to stir for 16 h at rt. CH_2Cl_2 (100 mL) was added and the resulting solution was washed with 1 N $KHSO_4$ (3 × 100 mL) and brine (200 mL), dried over $MgSO_4$, filtered and concentrated *in vacuo*. The resulting crude dark red liquid was used without isolation.

^{1}H NMR ($CDCl_3$, 400 MHz): δ 4.75 (br, 1H), 3.40 (t, J = 6.5 Hz, 2H), 3.22 (q, J = 6.4 Hz, 2H), 2.01 (quint, J = 6.6 Hz, 2H), 1.40 (s, 9H).

These spectral characteristics were identical to those previously reported.[7]

***tert*-Butyl (3-azidopropyl)carbamate (DA3)**

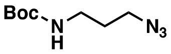

tert-Butyl-(3-bromopropyl)carbamate **DA2** (27.0 g, 113 mmol, 1.00 equiv) and NaN_3 (14.7 g, 227 mmol, 2.00 equiv) were dissolved in DMF (300 mL) and stirred at 80 °C for 16 h. DMF was removed *in vacuo* and the residue was dissolved with CH_2Cl_2 (330 mL). The resulting solution was washed with 1 N $KHSO_4$ (3 × 100 mL) and brine (3 × 100ml), dried over $MgSO_4$, filtered and concentrated *in vacuo*. The resulting residue was co-evaporated with toluene (2 × 100 mL). The resulting dark red liquid was used without isolation.

^{1}H NMR ($CDCl_3$, 400 MHz): δ 4.63 (br, 1H), 3.36 (t, J = 6.7 Hz, 2H), 3.21 (q, J = 6.4 Hz, 2H), 1.77 (quint, J = 6.7 Hz, 2H), 1.44 (s, 9H).

These spectral characteristics were identical to those previously reported.[7]

7) Barnard, A.; Long, K.; Yeo, D. J.; Miles, J. A.; Azzarito, V.; Burslem, G. M.; Prabhakaran, P.; Edwards, T. A.; Wilson, A. J.: Orthogonal functionalisation of α-helix mimetics. *Org. Biomol. Chem.*, **2014**, *12*, 6794–6799.

tert-Butyl (3-azidopropyl)((tributylstannyl)methyl)carbamate (DA4)

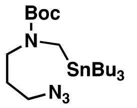

Sodium hydride (2.10 g of a 60% suspension in mineral oil, 52.2 mmol, 1.50 equiv) was washed with n-hexane and suspended in THF (45 mL). The suspension was cooled to 0 °C and azide **DA3** (13.9 g, 69.6 mmol, 2.00 equiv) was added dropwise over 10 min. The resulting suspension was allowed to stir at rt until no further gas formation was observed. The suspension was cooled to 0 °C and tributyl(iodomethyl)stannane (15.0 g, 34.8 mmol, 1.00 equiv) was added dropwise over 3 min. The mixture was allowed to stir at 55 °C for 16 h. The reaction mixture was allowed to cool to rt and treated with water (15 mL). The aqueous layer was extracted with Et_2O (3 × 15 mL). The combined organic layers were washed with water (2 × 15 mL), brine (20 mL), dried over Na_2SO_4, filtered and concentrated *in vacuo*. The crude product was purified by column chromatography on silica gel (hexanes/EtOAc = 1:0 → 8:1) to afford the desired the reagent **DA4** (4.1 g, 23%).

^{1}H NMR ($CDCl_3$, 400 MHz): δ 3.32–3.21 (m, 4H), 3.03 (m, 0.67H), 2.78 (m, 1.33H), 1.87–1.74 (m, 2H), 1.52–1.43 (m, 15H), 1.33–1.24 (m, 6H), 0.90–0.82 (m, 15H).

^{13}C NMR ($CDCl_3$, 100 MHz): δ (155.52, 155.39), (79.61, 79.31), (49.33, 49.09), (47.63, 46.46), (33.68, 33.21), 29.25 (3C), (28.62, 28.57), 27.58 (3C), 13.83 (3C), 10.62 (3C).

HRMS (ESI) m/z for $C_{21}H_{44}N_4NaO_2Sn$ $[M+H]^+$ calcd 527.2384, found 527.2386.

IR (ν/cm^{-1}, neat) 2956, 2924, 2871, 2853, 2097, 1681, 1481, 1464, 1405, 1365, 1253, 1163.

3.5 Synthetic Route towards SnAP (2-spiro-(4-EG-cy)-M) Azide (SM3)

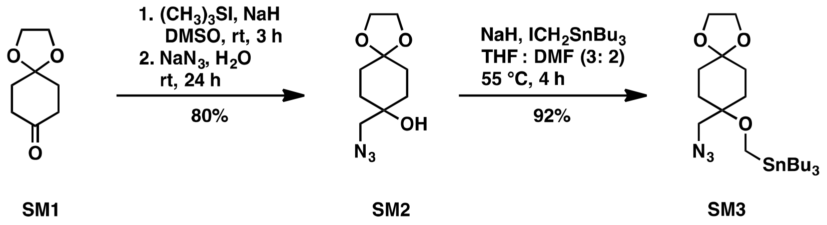

8-(azidomethyl)-1,4-dioxaspiro[4.5]decan-8-ol (SM2)

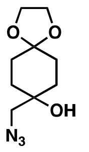

Trimethylsulfonium iodide (50.7 g, 230 mmol) was dissolved in anhydrous DMSO (600 mL) and cooled with an ice bath. Sodium hydride (60 mass%, 14.7 g, 231 mmol) was added portionwise then 1,4-dioxaspiro[4.5]decan-8-one (30.0 g, 192 mmol) in DMSO (300 mL) was added dropwise. The reaction was allowed to return to room temperature over 3 hours. The reaction was diluted with water (600 mL) and the product was extracted in MTBE (5 x 500 mL). The organic phase was washed with water (300 mL) then concentrated to afford a clear oil. Water (600 mL) and sodium azide (62.4 g, 960 mmol) were added. After stirring at room temperature for 24 h the product was extracted in ethyl acetate (5 x 200 mL). The organic phase was dried over anhydrous sodium sulfate then concentrated to dryness to afford a crystalline solid. The product was recrystallized in 400 mL of a 1:1 diethyl ether : *n*-hexane solution to afford the title compound as a crystalline solid (32.9 g, 154 mmol, 80% yield, m.p. 45 °C).

^{1}H NMR ($CDCl_3$, 300 MHz): δ 4.03–3.91 (m, 4H), 3.32 (s, 2H), 2.00–1.84 (m, 2H), 1.81–1.57 (m, 7H).

^{13}C NMR ($CDCl_3$, 100 MHz): δ 108.4, 70.6, 64.4, 64.2, 61.7, 32.5 (2C), 30.0 (2C).

HRMS (ESI) m/z for $C_{19}H_{15}N_3O_3[M+Na]^+$ calcd 236.1006, found 236.1011

(((8-(azidomethyl)-1,4-dioxaspiro[4.5]decan-8-yl)oxy)methyl)tributylstannane (SM3)

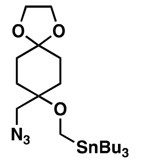

Sodium hydride (60 mass%, 3.38 g, 84.4 mmol) and anhydrous THF (110 mL) were added to a dry 500 mL r.b. flask. 8-(Azidomethyl)-1,4-dioxaspiro[4.5]decan-8-ol (15.0 g, 70.3 mmol) was dissolved in DMF (75 mL) and added dropwise to the reaction. After stirring at 55 °C for 30 minutes the slurry became a light orange solution. Tributyl(iodomethyl)stannane (36.4 g, 84.4 mmol) was added dropwise and a white precipitate began to form. After 3 hours at 55 °C the reaction was cooled to room temperature and diluted with water (200 mL). The product was extracted in MTBE (4 x 100 mL), dried over anhydrous sodium sulfate and concentrated under reduced pressure. The product was then purified by flash silica chromatography (1 : 19 ethyl acetate : *n*-hexane) to afford the title compound as a clear oil (33.4 g, 64.7 mmol, 92% yield).

^{1}H NMR ($CDCl_3$, 400 MHz): δ 4.02–3.89 (m, 4H), 3.44 (t, J = 12.0 Hz, 2H), 3.23 (s, 2H), 1.97–1.87 (m, 2H), 1.81–1.70 (m, 2H), 1.58–1.48 (m, 10H), 1.39–1.25 (m, 6H), 0.97–0.88 (m, 15H).

^{13}C NMR ($CDCl_3$, 100 MHz): δ 108.7, 75.6, 64.3, 64.2, 54.8, 49.9, 29.9 (2C), 29.2 (3C), 28.9 (2C), 27.4 (3C), 13.8 (3C), 9.0 (3C).

HRMS (ESI) m/z for $C_{22}H_{43}N_3O_3Sn$ $[M+Na]^+$ calcd 540.2223, found 540.2224.

3.6 Loading of the Resin

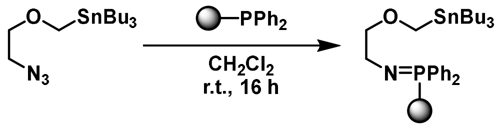

To a suspension of polystyrene-supported PPh_3 (10 g, 17 mmol, 1.7 mmol/g) in CH_2Cl_2 (60 mL) was added SnAP M azide (7.96 g, 20.4 mmol, 1.20 equiv) dropwise. The mixture was stirred at room temperature for 16 h and filtered. The crude resin was further washed with CH_2Cl_2 (50 mL × 6) and dried *in vacuo* to give SnAP M resin.

SnAP Pip, OA, DA and SM resins were prepared following the same procedure.

The loading of the SnAP resin (1.00 equiv) was determined by reacting with excess amounts of benzaldehyde (1.50 equiv) in CH_2Cl_2 and determine the unreacted aldehyde after 24 hours. The loading of each resin is shown as below:

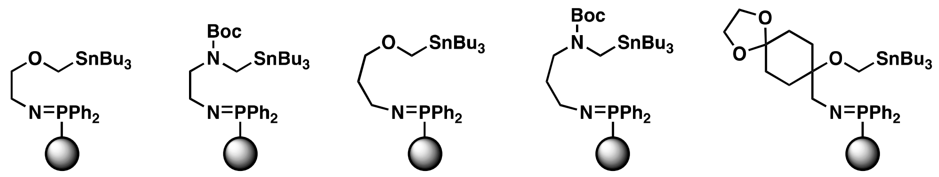

4. Automated Heterocycle Formation on the Console

4.1 General Procedure A for the Automated N-Heterocycle Synthesis on the Console

A 40 mL vial was charged with the aldehyde (0.5 mmol). The vial was placed on the console and connected to the console via a screw cap. The capsule with the desired SnAP reagent (four compartments, containing also the Cu catalyst, silica and Si-SCX-2) was inserted into the console and the capsule holder was closed. The reaction program was selected, either by scanning the RFID chip on the capsule or by manual selection. The reaction started automatically upon initiation of the sequence. After completion of the reaction sequence after 11 hours, the solution in the vial was concentrated *in vacuo* to afford the crude product. The yield and purity of the product was determined by ^{1}H NMR analysis with an internal standard or by isolation.

Heterocycle forming capsule contents:

Compartment 1: Top: 4 Å molecular sieves powder (100 mg). Bottom: SnAP resin (2.00 equiv)
Compartment 2: Top: 2,6-Lutidinium triflate (1.00 equiv). Bottom: Copper(II) triflate (1.00 equiv).
Compartment 3: SCX-2 (4.0 equiv)
Compartment 4: Silica (5 g)

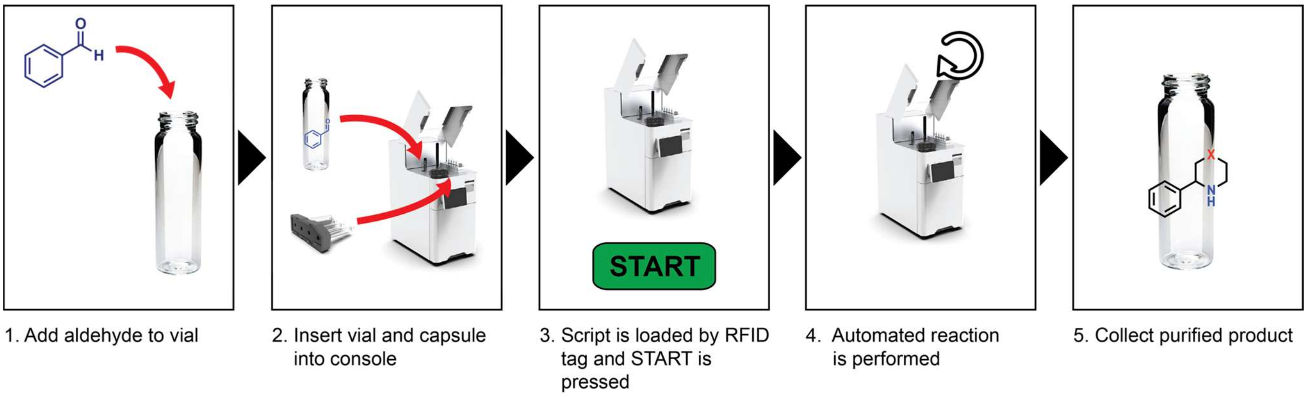

Fig. S1: 5 Steps to use the automated synthesizer for N-heterocycle synthesis.

A video of the reaction setup is available at: https://youtu.be/a01CglvgUVQ

Description of console process

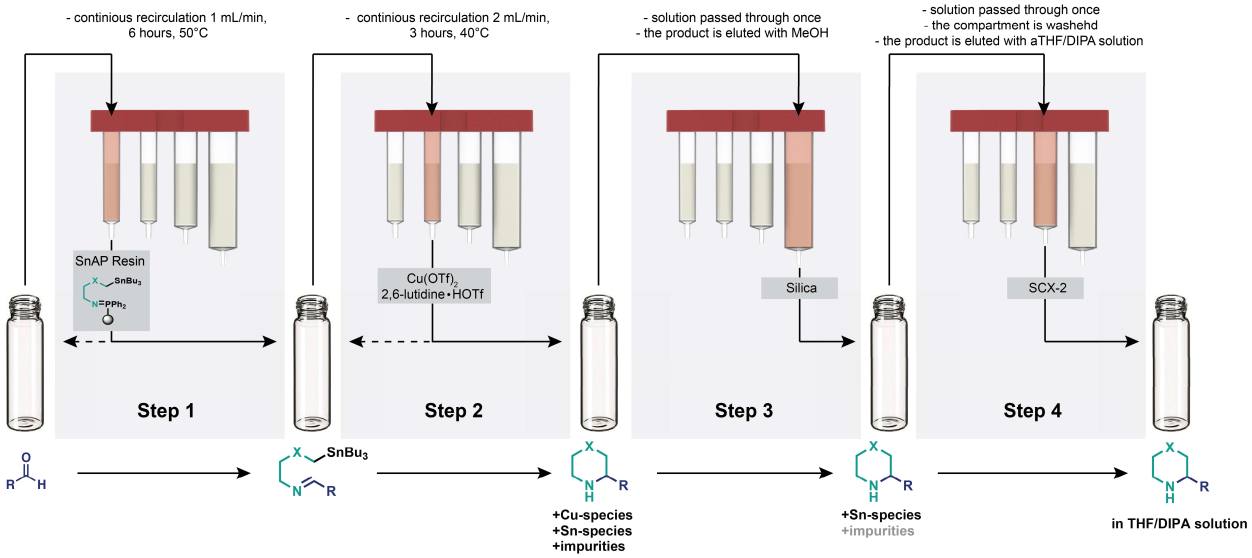

Step 1: Imine Formation

In the first step the neat aldehyde is dissolved in anhydrous CH_2Cl_2 (4.5 mL) from the solvent reservoirs. The solution is then circulated through cartridge compartment 1 (SnAP reagent) at 1 mL/min for 6 hours. Compartment 1 is heated at 50 °C and the reaction vial is heated at 35 °C. After the imine formation is complete, compartment 1 is rinsed into the vial with anhydrous CH_2Cl_2 (3.5 mL) from the solvent reservoirs.

Step 2: Cyclization

Anhydrous HFIP (2.0 mL) is added via compartment 2 (Cu-Catalyst) into the reaction vial. The solution of imine in anhydrous CH_2Cl_2 and HFIP is circulated through compartment 2 at 2 mL/min for 3 hours. Compartment 2 is heated at 40 °C and the reaction vial is heated at 37 °C. After the reaction time, compartment 2 is rinsed with anhydrous CH_2Cl_2 (2 mL).

Step 3: Purification A

The reaction mixture is loaded into compartment 4 (Silica).
Compartment 4 is washed with MeOH (12 mL) from the solvent reservoirs. This step removed most of the Cu-species. The product is washed back into the reaction vial.

Step 4: Purification B and Product release

The filtrate from the previous step in the reaction vial is then loaded into compartment 3 (SCX) and the filtrate is discarded to waste.
Compartment 3 is washed with MeOH (12 mL) and the filtrate is discarded to waste. This step is repeated once more.
Compartment 3 is washed with CH_2Cl_2 (10 mL) and the filtrate is discarded to waste. This step is repeated once more.
Compartment 3 is washed into the vial with 2.5 M DIPA/THF (20 mL). The filtrate contains the N-heterocycle product.

4.2 Characterization of Reaction Products

4.2.1 Morpholine products

3-Phenylmorpholine (1a)

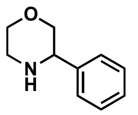

3-Phenylmorpholine was prepared according to the General Procedure A from benzaldehyde (48.9 mg, 0.500 mmol). The product was obtained in 60% yield (calculated from ^{1}H-NMR using 0.25 mmol (0.5 equiv) of mesitylene as internal standard) (77.1 mg, 0.300 mmol).

^{1}H NMR (400 MHz, CDCl3): *δ* 7.42–7.36 (m, 2H), 7.36–7.30 (m, 2H), 7.30–7.24 (m, 1H), 3.92 (dd, *J* = 10.1, 3.2 Hz, 1H), 3.91–3.85 (m, 1H), 3.82 (dd, *J* = 11.1, 3.2 Hz, 1H), 3.66 (ddd, *J* = 11.3, 11.1, 2.7 Hz, 1H), 3.40 (dd, *J* = 11.1, 10.1 Hz, 1H), 3.13 (ddd, *J* = 11.7, 11.3, 3.2 Hz, 1H), 3.03–2.97 (m, 1H), 1.83 (br s, NH).

^{13}C NMR (100 MHz, CDCl3): *δ* 140.66, 128.61 (2C), 127.87, 127.26 (2C), 73.8, 67.4, 60.7, 46.8.

These spectral characteristics were identical to those previously reported.[8]

3-(2-Fluorophenyl)morpholine (1b)

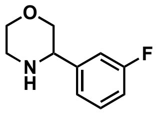

3-(2-Fluorophenyl)morpholine was prepared according to the General Procedure A from 2-fluorobenzaldehyde (62.0 mg, 0.500 mmol). The product was obtained in 53% yield (calculated from ^{1}H-NMR using 0.25 mmol (0.5 equiv) of mesitylene as internal standard) (48.0 mg, 0.265 mmol).

Column conditions: hexanes/EtOAc (v/v = 2/1, + 0.25% Et_3N).

^{1}H NMR ($CDCl_3$, 400 MHz): *δ* 7.31–7.25 (m, 1H), 7.15–7.12 (m, 1H), 6.98–6.93 (m, 1H), 3.92 (dd, *J* = 10.1, 3.2 Hz, 1H), 3.87 (m, 1H), 3.81 (dd, *J* = 11.0, 3.2 Hz, 1H), 3.63 (ddd, *J* = 11.3, 11.3, 2.7 Hz, 1H), 3.34 (dd, *J* = 11.0, 10.1 Hz, 1H), 3.12 (ddd, *J* = 11.6, 11.3, 3.3 Hz, 1H), 2.99 (m, 1H), 1.94 (br s, 1H).

^{13}C NMR ($CDCl_3$, 100 MHz): *δ* 163.08 (d, *J* = 245.9 Hz, 1C), 143.35 (d, *J* = 7.1 Hz, 1C), 130.05 (d, *J* = 8.2 Hz, 1C), 122.88 (d, *J* = 2.9 Hz, 1C), 114.71 (d, *J* = 21.2 Hz, 1C), 114.16 (d, *J* = 21.9 Hz, 1C), 73.61, 67.33, 60.13 (d, *J* = 1.7 Hz, 1C), 46.53.

HRMS (ESI) m/z for $C_{10}H_{13}FNO$ $[M+H]^+$ calcd 182.0976, found 182.0976.

8) Hu, P.; Hu, J.; Jiao, J.; Tong, X.: Amine-Promoted Asymmetric (4+2) Annulations for the Enantioselective Synthesis of Tetrahydropyridines: A Traceless and Recoverable Auxiliary Strategy. *Angew. Chem. Int. Ed.* **2013**, *52*, 5319.

3-(4-(Trifluoromethyl)phenyl)morpholine (1c)

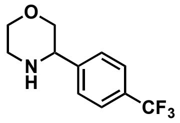

3-(4-(Trifluoromethyl)phenyl)morpholine was prepared according to the General Procedure A from 4-(trifluoromethyl) benzaldehyde (87.0 mg, 0.500 mmol). The product was obtained in 46% yield (calculated from ^{1}H-NMR using 0.25 mmol (0.5 equiv) of mesitylene as internal standard) (53.1 mg, 0.230 mmol).

Column conditions: hexanes/EtOAc (v/v = 2/1, + 0.25% Et_3N).

^{1}H NMR ($CDCl_3$, 400 MHz): δ 7.60–7.58 (m, 2H), 7.53–7.51 (m, 2H), 3.99 (dd, J = 10.0, 3.2 Hz, 1H), 3.89 (m, 1H), 3.81 (dd, J = 11.1, 3.2 Hz, 1H), 3.65 (ddd, J = 11.3, 11.2, 2.3 Hz, 1H), 3.35 (dd, J = 11.1, 10.0, 1H), 3.14 (ddd, J = 11.7, 11.3, 3.3 Hz, 1H), 3.01 (m, 1H), 1.90 (br s, 1H).

^{13}C NMR ($CDCl_3$, 100 MHz): δ 144.72 (q, J = 1.2 Hz, 1C), 130.15 (q, J = 32.4 Hz, 1C), 127.66 (2C), 125.57 (q, J = 3.8 Hz, 2C), 124.23 (q, J = 271.9 Hz, 1C), 73.56, 67.35, 60.28, 46.49.

HRMS (ESI) m/z for $C_{11}H_{13}F_3NO$ $[M+H]^+$ calcd 232.0944, found 232.0944.

Methyl 4-(morpholin-3-yl)benzoate (1d)

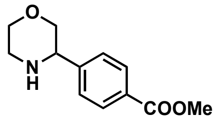

Methyl 4-(morpholin-3-yl)benzoate was prepared according to the General Procedure A from methyl 4-formylbenzoate (82.0 mg, 0.500 mmol). The product was obtained as in 50% yield (calculated from ^{1}H-NMR using 0.25 mmol (0.5 equiv) of mesitylene as internal standard) (52.8 mg, 0.250 mmol).

^{1}H NMR (400 MHz, $CDCl_3$): δ 8.03–7.90 (m, 2H), 7.51–7.38 (m, 2H), 3.97 (dd, J = 10.0, 3.2 Hz, 1H), 3.90 (s, 3H), 3.88 (m, 1H), 3.80 (dd, J = 11.2, 3.2 Hz, 1H), 3.63 (ddd, J = 11.3, 11,2, 2.7 Hz, 1H), 3.34 (dd, J = 11.0, 10.0 Hz, 1H), 3.11 (ddd, J = 11.6, 11,3, 3.2 Hz, 1H), 2.99 (m, 1H), 1.94 (br s, NH).

^{13}C NMR (100 MHz, $CDCl_3$): δ 166.97, 145.82, 129.92 (2C), 129.73, 127.26 (2C), 73.5, 67.3, 60.4, 52.2, 46.4.

These spectral characteristics were identical to those previously reported.[2]

3-(*o*-Tolyl)morpholine (1e)

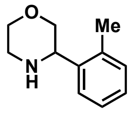

3-(*o*-Tolyl)morpholine was prepared according to the General Procedure A from 2-methylbenzaldehyde (60.0 mg, 0.500 mmol). The product was obtained as in 42% yield (calculated from ^{1}H-NMR using 0.25 mmol (0.5 equiv) of mesitylene as internal standard) (37.2 mg, 0.210 mmol).

^{1}H NMR (400 MHz, $CDCl_3$): δ 7.58 (dd, J = 7.3, 1.6 Hz, 1H), 7.24–7.11 (m, 3H), 4.15 (dd, J = 9.9, 3.0 Hz, 1H), 3.89 (m, 1H), 3.81 (dd, J = 11.1, 3.0 Hz, 1H), 3.67 (ddd, J = 11.3, 11.1, 2.6 Hz, 1H), 3.34 (dd, J = 11.1, 9.9 Hz, 1H), 3.16 (ddd, J = 11.6, 11.3, 3.3 Hz,1H), 3.03 (m, 1H), 2.39 (s, 3H), 1.70 (br s, NH).

^{13}C NMR (100 MHz, $CDCl_3$): δ 138.61, 135.62, 130.49, 127.43, 126.70, 126.43, 72.63, 67.49, 56.85, 47.11, 19.37.

These spectral characteristics were identical to those previously reported.[9]

9) Luescher, M. U.; Bode, J. W.: Catalytic Synthesis of N-Unprotected Piperazines, Morpholines, and Thiomorpholines from Aldehydes and SnAP Reagents. *Angew. Chem. Int. Ed.* **2015**, *54*, 10884–10888.

3-(4-(*tert*-Butyl)phenyl)morpholine (1f)

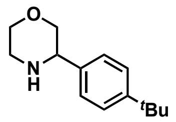

3-(4-(*tert*-Butyl)phenyl)morpholine was prepared according to the General Procedure A from 4-(*tert*-butyl)benzaldehyde (81.0 mg, 0.500 mmol). The product was obtained in 33% yield (calculated from ^{1}H-NMR using 0.25 mmol (0.5 equiv) of mesitylene as internal standard) (36.2 mg, 0.165 mmol).

Column conditions: hexanes/EtOAc (v/v = 2/1, + 0.25% Et_3N).

^{1}H NMR ($CDCl_3$, 400 MHz): δ 7.37–7.35 (m, 2H), 7.32–7.30 (m, 2H), 3.90 (dd, J = 10.2, 3.1 Hz, 1H), 3.87 (m, 1H), 3.82 (dd, J = 11.0, 3.1 Hz, 1H), 3.65 (ddd, J = 11.5, 11.3, 2.7 Hz, 1H), 3.40 (dd, J = 11.0, 10.2 Hz, 1H), 3.13 (ddd, J = 11.7, 11.5, 3.3 Hz, 1H), 3.00 (m, 1H), 1.94 (br s, 1H), 1.31 (s, 9H).

^{13}C NMR ($CDCl_3$, 100 MHz): δ 150.83, 137.67, 126.96 (2C), 125.51 (2C), 73.82, 67.35, 60.37, 46.84, 34.65, 31.48 (3C).

HRMS (ESI) m/z for $C_{14}H_{22}NO$ $[M+H]^+$ calcd 220.1696, found 220.1692.

3-(4-Methoxyphenyl)morpholine (1g)

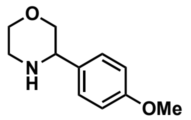

3-(4-Methoxyphenyl)morpholine was prepared according to the General Procedure A from 4-methoxybenzaldehyde (68.0 mg, 0.500 mmol). The product was obtained in 15% yield (calculated from ^{1}H-NMR using 0.25 mmol (0.5 equiv) of mesitylene as internal standard) (14.5 mg, 0.0750 mmol).

^{1}H NMR (400 MHz, $CDCl_3$): δ 7.34–7.28 (m, 2H), 6.90–6.84 (m, 2H), 3.88–3.84 (m, 2H), 3.79 (s, 3H), 3.78 (m, 1H), 3.64 (ddd, J = 11.3, 11.1, 2.6 Hz, 1H), 3.37 (dd, J = 11.1, 10.2 Hz, 1H), 3.12 (ddd, J = 11.6, 11.3, 3.2 Hz, 1H), 2.99 (m, 1H), 1.69 (br s, 1H).

^{13}C NMR (100 MHz, $CDCl_3$): δ 159.29, 132.87, 128.36 (2C), 114.00 (2C), 73.89, 67.32, 60.07, 55.42, 46.85.

These spectral characteristics were identical to those previously reported.[2]

Benzyl 4-(morpholin-3-yl)piperidine-1-carboxylate (1h)

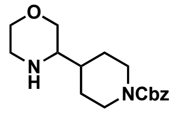

Benzyl 4-(morpholin-3-yl)piperidine-1-carboxylate was prepared according to the General Procedure A from 4-formyl-N-Cbz-piperidine. The product was obtained in 36% yield (calculated from ^{1}H-NMR using 0.25 mmol (0.5 equiv) of mesitylene as internal standard) (54.8 mg, 0.180 mmol).

Column conditions: EtOAc/MeOH (v/v = 5/1, + 0.25% Et_3N).

^{1}H NMR (400 MHz, $CDCl_3$): δ 7.36–7.30 (m, 5H), 5.11 (s, 2H), 4.22 (m, 2H), 3.87 (dd, J = 11.2, 2.9 Hz, 1H), 3.80 (ddd, J = 11.2, 2.7, 2.7 Hz, 1H), 3.52 (m, 1H), 3.29 (dd, J = 11.2, 10.1 Hz, 1H), 2.96−2.93 (m, 2H), 2.76–2.72 (m, 2H), 2.62 (ddd, J = 10.1, 7.4, 3.0 Hz, 1H), 1.80–1.77 (m, 1H), 1.66–1.62 (m, 1H), 1.53–1.44 (m, 1H), 1.27–1.18 (m, 2H).

^{13}C NMR (100 MHz, $CDCl_3$): δ 155.26, 136.93, 128.61 (2C), 128.10, 127.99 (2C), 70.33, 67.28, 67.18, 58.96, 46.04, 44.10, 43.99, 38.22, 28.23 (2C).

HRMS (ESI) m/z for $C_{17}H_{25}N_2O_3$ $[M+H]^+$ calcd 305.1860, found 305.1860.

3-(4-(4,4,5,5-Tetramethyl-1,3,2-dioxaborolan-2-yl)phenyl)morpholine (1i)

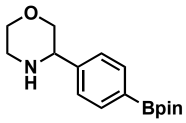

3-(4-(4,4,5,5-Tetramethyl-1,3,2-dioxaborolan-2-yl)phenyl)morpholine was prepared according to the General Procedure A from 4-(4,4,5,5-tetramethyl-1,3,2-dioxaborolan-2-yl)benzaldehyde (116 mg, 0.500 mmol). The product was obtained in 27% yield (calculated from ^{1}H-NMR using 0.25 mmol (0.5 equiv) of mesitylene as internal standard) (39.0 mg, 0.135 mmol).

Column conditions: hexanes/EtOAc (v/v = 2/1, + 0.25% Et_3N).

^{1}H NMR ($CDCl_3$, 400 MHz): δ 7.79–7.77 (m, 2H), 7.41–7.39 (m, 2H), 3.94 (dd, J = 10.1, 3.2 Hz, 1H), 3.87 (m, 1H), 3.81 (dd, J = 11.1, 3.2 Hz, 1H), 3.65 (ddd, J = 11.5, 11.0, 2.7 Hz, 1H), 3.38 (dd, J = 11.1, 10.1 Hz, 1H), 3.13 (ddd, J = 11.9, 11.5, 3.3 Hz, 1H), 3.00 (m, 1H), 1.90 (br s, 1H), 1.33 (s, 12H).

^{13}C NMR ($CDCl_3$, 100 MHz): δ 143.74, 135.13 (2C), 128.51, 126.64 (2C), 83.94 (2C), 73.68, 67.33, 60.79, 46.65, 24.98 (4C).

HRMS (ESI) m/z for $C_{16}H_{25}BNO_3$ $[M+H]^+$ calcd 290.1925, found 290.1904.

3-(Pyridin-4-yl)morpholine (1j)

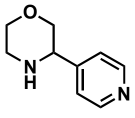

3-(Pyridin-4-yl)morpholine was prepared according to the General Procedure A from isonicotinaldehyde (53.5 mg, 0.500 mmol). The product was obtained in 44% yield (calculated from ^{1}H-NMR using 0.25 mmol (0.5 equiv) of mesitylene as internal standard) (36.1 mg, 0.220 mmol).

Column conditions: EtOAc/MeOH (v/v = 5/1, + 0.25% Et_3N).

^{1}H NMR ($CDCl_3$, 400 MHz): δ 8.55–8.54 (m, 2H), 7.32–7.31 (m, 2H), 3.94 (dd, J = 9.9, 3.2 Hz, 1H), 3.89–3.86 (m, 1H), 3.82 (dd, J = 11.2, 3.2 Hz, 1H), 3.65 (ddd, J = 11.3, 11.2, 2.8 Hz, 1H), 3.35 (dd, J = 11.2, 9.9 Hz, 1H), 3.11 (ddd, J = 11.8, 11.2, 3.3 Hz, 1H), 3.02–2.98 (m, 1H), 2.67 (br. s, 1H).

^{13}C NMR ($CDCl_3$, 100 MHz): δ 150.16 (2C), 148.90, 122.31 (2C), 72.85, 67.14, 59.41, 46.04.

HRMS (ESI) m/z for $C_9H_{13}N_2O$ $[M+H]^+$ calcd 165.1022, found 165.1026.

4.2.2 Piperazine products

***tert*-Butyl 3-(4-bromophenyl)piperazine-1-carboxylate (2a)**

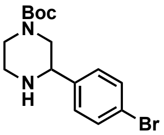

tert-Butyl 3-(4-bromophenyl)piperazine-1-carboxylate was prepared according to the General Procedure A from 4-bromobenzaldehyde (92.0 mg, 0.500 mmol). The product was obtained in 70% yield (calculated from ^{1}H-NMR using 0.25 mmol (0.5 equiv) of mesitylene as internal standard) (119 mg, 0.350 mmol).

Column conditions: hexanes/EtOAc (v/v = 2/1, + 0.25% Et_3N).

^{1}H NMR ($CDCl_3$, 400 MHz): δ 7.45–7.43 (m, 2H), 7.28–7.26 (m, 2H), 4.01 (m, 2H), 3.65 (dd, J = 10.6, 3.1 Hz, 1H), 3.05–3.02 (m, 1H), 2.87–2.81 (m, 2H), 2.65 (m, 1H), 1.86 (br. s, 1H), 1.45 (s, 9H).

^{13}C NMR ($CDCl_3$, 100 MHz): δ 154.75, 140.63, 131.70 (2C), 128.82 (2C), 121.61, 79.92, 59.71, (51.56, 50.41, 1C), 46.04, (44.38, 43.31, 1C), 28.53 (3C).

HRMS (ESI) m/z for $C_{15}H_{22}BrN_2O_2$ $[M+H]^+$ calcd 341.0859, found 341.0858.

***tert*-Butyl 3-(2-chloro-4-fluorophenyl)piperazine-1-carboxylate (2b)**

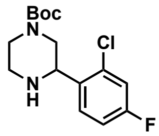

tert-Butyl 3-(2-chloro-4-fluorophenyl)piperazine-1-carboxylate was prepared according to the General Procedure A from 2-chloro-4-fluorobenzaldehyde (79.0 mg, 0.500 mmol). The product was obtained in 64% yield (calculated from ^{1}H-NMR using 0.25 mmol (0.5 equiv) of mesitylene as internal standard) (101 mg, 0.320 mmol).

^{1}H NMR (400 MHz, $CDCl_3$): δ 7.70–7.54 (m, 1H), 7.11 (dd, J = 8.5, 2.6 Hz, 1H), 6.99 (dd, J = 8.5, 2.6 Hz, 1H), 4.32–3.92 (m, 3H), 3.13–3.02 (m, 1H), 2.99–2.82 (m, 2H), 2.58 (dd, J = 12.7, 10.2 Hz, 1H), 1.73 (br s, NH), 1.47 (s, 9H).

^{13}C NMR (100 MHz, $CDCl_3$): δ 161.8 (d, J_{CF} = 249.7 Hz), 154.8, 134.9 (d, J_{CF} = 3.5 Hz), 133.7 (d, J_{CF} = 11.7 Hz), 129.2 (d, J_{CF} = 8.7 Hz), 117.0 (d, J_{CF} = 24.6 Hz), 114.4 (d, J_{CF} = 20.7 Hz), 80.0, 56.0, 50.2, 46.2, 43.5, 28.6.

These spectral characteristics were identical to those previously reported.[2]

***tert*-Butyl 3-(3-cyanophenyl)piperazine-1-carboxylate (2c)**

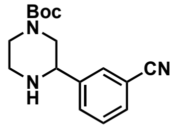

tert-Butyl 3-(3-cyanophenyl)piperazine-1-carboxylate was prepared according to the General Procedure A from 3-cyanobenzaldehyde (65.5 mg, 0.500 mmol). The product was obtained in 59% yield (calculated from ^{1}H-NMR using 0.25 mmol (0.5 equiv) of mesitylene as internal standard) (84.7 mg, 0.295 mmol).

Column conditions: hexanes/EtOAc (v/v = 2/1, + 0.25% Et_3N).

^{1}H NMR ($CDCl_3$, 400 MHz): δ 7.74–7.73 (m, 1H), 7.65–7.63 (m, 1H), 7.58–7.55 (m, 1H), 7.45–7.41 (m, 1H), 4.02 (m, 2H), 3.74 (dd, J = 10.5, 3.1 Hz, 1H), 3.07–3.04 (m, 1H), 2.92–2.83 (m, 2H), 2.67 (m, 1H), 1.88 (br. s, 1H), 1.46 (s, 9H).

^{13}C NMR ($CDCl_3$, 100 MHz): δ 154.69, 143.18, 131.75, 131.58, 130.81, 129.43, 118.80, 112.73, 80.13, 59.49, (51.38, 50.39, 1C), 45.88, (44.29, 43.32, 1C), 28.52 (3C).

HRMS (ESI) m/z for $C_{16}H_{22}N_3O_2$ $[M+H]^+$ calcd 288.1707, found 288.1711.

***tert*-Butyl 3-cyclopropylpiperazine-1-carboxylate (2d)**

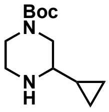

tert-Butyl 3-cyclopropylpiperazine-1-carboxylate was prepared according to the General Procedure A from cyclopropanecarbaldehyde (35.0 mg, 0.500 mmol). The product was obtained in 44% yield (calculated from ^{1}H-NMR using 0.25 mmol (0.5 equiv) of mesitylene as internal standard) (49.8 mg, 0.220 mmol).

^{1}H NMR (400 MHz, CDCl3): δ 4.16–3.80 (m, 2H), 2.96 (br d, J = 11.3 Hz, 1H), 2.91–2.78 (m, 1H), 2.67 (td, J = 11.4, 3.1 Hz, 2H), 1.74 (td, J = 10.2, 2.8 Hz, 1H), 1.66 (br s, NH), 1.46 (s, 9H), 0.77–0.67 (m, 1H), 0.58–0.41 (m, 2H), 0.29–0.11 (m, 2H).

^{13}C NMR (100 MHz, $CDCl_3$): δ 155.0, 79.7, 60.8, 45.9, 28.6, 14.5, 2.8, 2.2.

These spectral characteristics were identical to those previously reported.[2]

***tert*-Butyl 3-(1-(tert-butoxycarbonyl)piperidin-4-yl)piperazine-1-carboxylate (2e)**

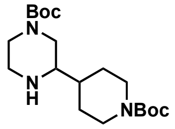

tert-Butyl 3-(1-(tert-butoxycarbonyl)piperidin-4-yl)piperazine-1-carboxylate was prepared according to the General Procedure A from *tert*-butyl 4-formylpiperidine-1-carboxylate (107 mg, 0.500 mmol). The product was obtained in 33% yield (calculated from ^{1}H-NMR using 0.25 mmol (0.5 equiv) of mesitylene as internal standard) (60.9 mg, 0.165 mmol).

^{1}H NMR (400 MHz, $CDCl_3$): δ 4.30–3.97 (m, 3H), 3.95–3.76 (m, 1H), 3.00–2.93 (m, 1H), 2.86–2.78 (m, 1H), 2.74–2.67 (m, 1H), 2.74–2.41 (m, 3H), 2.40–2.32 (m, 1H), 1.73 (br d, J = 12.7 Hz, 1H), 1.70 (br d, J = 12.7 Hz, 1H), 1.59 (br s, NH), 1.48 (s, 9H), 1.47 (s, 9H), 1.32–1.27 (m, 1H), 1.25– 1.16 (m, 2H).

^{13}C NMR (100 MHz, $CDCl_3$): δ 155.0, 154.9, 79.9, 79.6, 59.1, 48.0, 47.1, 45.7, 45.1, 44.2, 43.7, 39.4, 28.6, 28.6, 28.4, 28.4.

These spectral characteristics were identical to those previously reported. [9]

1-(*tert*-Butyl) 3-ethyl piperazine-1,3-dicarboxylate (2f)

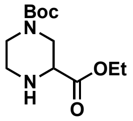

1-(*tert*-Butyl) 3-ethyl piperazine-1,3-dicarboxylate was prepared according to the General Procedure A from ethyl 2-oxoacetate (51.0 mg, 0.500 mmol). The product was obtained in 56% yield (calculated from ^{1}H-NMR using 0.25 mmol (0.5 equiv) of mesitylene as internal standard) (72.3 mg, 0.280 mmol).

^{1}H NMR (400 MHz, $CDCl_3$): δ 4.16 (q, J = 7.1 Hz, 3H), 3.66 (br d, J = 12.4 Hz, 1H), 3.38 (dd, J = 8.6, 3.5 Hz, 1H), 3.30–2.83 (m, 3H), 2.75–2.64 (m, 1H), 2.23 (br s, NH), 1.42 (s, 9H), 1.24 (t, J = 7.1 Hz, 3H).

^{13}C NMR (100 MHz, $CDCl_3$): δ 171.2, 154.6, 80.0, 61.2, 56.9, 45.8, 44.3, 43.5, 28.4, 14.2.

These spectral characteristics were identical to those previously reported.[2]

***tert*-Butyl 3-(thiophen-3-yl)piperazine-1-carboxylate (2g)**

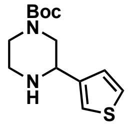

tert-Butyl 3-(thiophen-3-yl)piperazine-1-carboxylate was prepared according to the General Procedure A from thiophene-3-carbaldehyde (56.0 mg, 0.500 mmol). The product was obtained in 62% yield (calculated from ^{1}H-NMR using 0.25 mmol (0.5 equiv) of mesitylene as internal standard) (88.1 mg, 0.310 mmol).

Column conditions: hexanes/EtOAc (v/v = 3/2, + 0.25% Et_3N).

^{1}H NMR ($CDCl_3$, 400 MHz): δ 7.28 (dd, J = 5.0, 3.0 Hz, 1H), 7.23–7.21 (m, 1H), 7.09 (dd, J = 5.0, 1.3 Hz, 1H), 4.12–4.00 (m, 2H), 3.82 (dd, J = 10.3, 3.2 Hz, 1H), 3.05–3.01 (m, 1H), 2.94–2.77 (m, 3H), 1.93 (br. s, 1H), 1.46 (s, 9H).

^{13}C NMR ($CDCl_3$, 100 MHz): δ 154.81, 142.75, 126.39, 125.94, 121.26, 79.89, 55.91, (50.93, 50.24, 1C), 45.94, (44.63, 43.63, 1C), 28.56 (3C).

HRMS (ESI) m/z for $C_{13}H_{21}N_2O_2S$ $[M+H]^+$ calcd 269.1318, found 269.1319.

***tert*-Butyl 3-(pyridin-3-yl)piperazine-1-carboxylate (2h)**

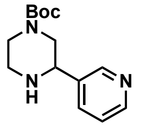

tert-Butyl 3-(pyridin-4-yl)piperazine-1-carboxylate was prepared according to the General Procedure A from nicotinaldehyde (53.5 mg, 0.500 mmol). Si-Trisamine (5 equiv) was packed underneath SCX-2 in Compartment 4. The product was obtained in 61% yield (calculated from ^{1}H-NMR using 0.25 mmol (0.5 equiv) of mesitylene as internal standard) (80.2 mg, 0.305 mmol).

Column conditions: EtOAc/MeOH (v/v = 15/1, + 0.25% Et_3N).

^{1}H NMR ($CDCl_3$, 400 MHz): δ 8.62 (d, J = 1.8 Hz, 1H), 8.51 (dd, J = 4.7, 1.5 Hz, 1H), 7.72 (ddd, J = 7.8, 1.8, 1.5 Hz, 1H), 7.31–7.16 (dd, J = 7.8, 4.7 Hz, 1H), 4.02 (m, 2H), 3.73 (dd, J = 10.6, 3.2 Hz, 1H), 3.07–3.03 (m, 1H), 2.95–2.83 (m, 2H). 2.72 (m, 1H), 1.94 (br. s, 1H), 1.45 (s, 9H).

^{13}C NMR ($CDCl_3$, 100 MHz): δ 154.73, 149.42, 149.02, 137.01, 134.69, 123.59, 80.05, 57.97, (51.33, 50.43, 1C), 46.01, (44.31, 43.38, 1C), 28.53 (3C).

HRMS (ESI) m/z for $C_{14}H_{22}N_3O_2$ $[M+H]^+$ calcd 264.1707, found 264.1706.

***tert*-Butyl 3-(quinolin-4-yl)piperazine-1-carboxylate (2i)**

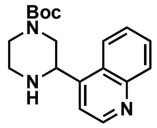

tert-Butyl 3-(quinolin-4-yl)piperazine-1-carboxylate was prepared according to the General Procedure A from quinoline-4-carbaldehyde (78.5 mg, 0.500 mmol). Si-Trisamine (5 equiv) was packed underneath SCX-2 in Compartment 4. The product was obtained in 72% yield (calculated from ^{1}H-NMR using 0.25 mmol (0.5 equiv) of mesitylene as internal standard) (113 mg, 0.360 mmol).

^{1}H NMR (400 MHz, $CDCl_3$): δ 8.88 (d, J = 4.5 Hz, 1H), 8.19 (d, J = 8.5 Hz, 1H), 8.13 (d, J = 8.3 Hz, 1H), 7.71 (ddd, J = 8.4, 6.9, 1.3 Hz, 1H), 7.65 (dd, J = 4.5, 0.8 Hz, 1H), 7.59 (ddd, J = 8.4, 6.8, 1.4 Hz, 1H), 4.48 (dd, J = 10.3, 2.8 Hz, 1H), 4.35 (br s, 1H), 4.14 (br s, 1H), 3.18 (d, J = 8.4 Hz, 1H), 3.07–2.94 (m, 2H), 2.74 (br s, 1H), 2.18 (br s, 1H), 1.50 (s, 9H).

^{13}C NMR (100 MHz, $CDCl_3$): δ 154.7, 150.6, 148.4, 146.7, 130.6, 129.30, 126.9, 126.2, 122.7, 118.2, 80.2, 77.5, 55.8, 51.4, 46.3, 44.0, 28.6, 28.5.

These spectral characteristics were identical to those previously reported.[10]

***tert*-Butyl 3-(2-phenylpyrimidin-5-yl)piperazine-1-carboxylate (2j)**

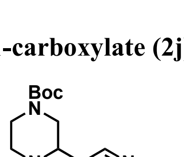

tert-Butyl 3-(2-phenylpyrimidin-5-yl)piperazine-1-carboxylate was prepared according to the General Procedure A from 2-phenylpyrimidine-5-carbaldehyde (92.0 mg, 0.500 mmol). Crude product was passed through Si-Trisamine (2 equiv) before NMR analysis.

Column conditions: CH_2Cl_2/EtOAc (v/v = 1/1, + 0.25% Et_3N). Isolated: 113 mg (66%).

10) Jindakun, C.; Hsieh, S.-Y.; Bode, J. W.: Iridium-catalyzed Synthesis of Saturated N-Heterocycles from Aldehydes and SnAP Reagents with Continuous Flow Photochemistry. *Org. Lett.* **2018**, *20*, 2071–2075.

^{1}**H NMR** ($CDCl_3$, 400 MHz): δ 8.83 (s, 2H), 8.44–8.41 (m, 2H), 7.49–7.47 (m, 3H), 4.40 (m, 2H), 3.78 (dd, J = 10.5, 3.2 Hz, 1H), 3.10–3.07 (m, 1H), 2.97–2.79 (m, 3H), 1.86 (br. s, 1H), 1.48 (s, 9H).

^{13}**C NMR** ($CDCl_3$, 100 MHz): δ 164.59, 156.42 (2C), 154.69, 137.46, 132.13, 130.88, 128.75 (2C), 128.25 (2C), 80.27, 55.97, (51.22, 50.21, 1C), 45.92, (44.30, 43.42, 1C), 28.55 (3C).

HRMS (ESI) m/z for $C_{19}H_{25}N_4O_2$ $[M+H]^+$ calcd 341.1972, found 341.1972.

***tert*-Butyl 3-(4-(pyridin-2-yl)phenyl)piperazine-1-carboxylate (2k)**

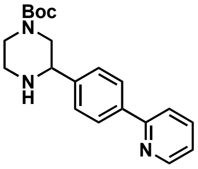

tert-Butyl 3-(4-(pyridin-2-yl)phenyl)piperazine-1-carboxylate was prepared according to the General Procedure A from 4-(pyridin-2-yl)benzaldehyde (91.5 mg, 0.500 mmol). Crude product was passed through Si-Trisamine (2 equiv) before NMR analysis.

Column conditions: hexanes/EtOAc (v/v = 1/1, + 0.25% Et_3N). Isolated: 77 mg (45%).

^{1}**H NMR** ($CDCl_3$, 400 MHz): δ 8.67–8.65 (m, 1H), 7.96–7.94 (m, 2H), 7.72–7.70 (m, 2H), 7.51–7.48 (m, 2H), 7.21–7.18 (m, 1H), 4.04 (m, 2H), 3.74 (dd, J = 10.7, 3.1 Hz, 1H), 3.08–3.04 (m, 1H), 2.93–2.84 (m, 2H), 2.75 (m, 1H), 1.99 (br. s, 1H), 1.46 (9H).

^{13}**C NMR** ($CDCl_3$, 100 MHz): δ 157.11, 154.81, 149.75, 142.39, 138.98, 136.81, 127.46 (2C), 127.10 (2C), 122.19, 120.51, 79.83, 60.09, (51.57, 50.55, 1C), 46.12, (44.43, 43.54, 1C), 28.53 (3C).

HRMS (ESI) m/z for $C_{20}H_{26}N_3O_2$ $[M+H]^+$ calcd 340.2020, found 340.2017.

***tert*-Butyl 3-(4-(1*H*-1,2,4-triazol-1-yl)phenyl)piperazine-1-carboxylate (2l)**

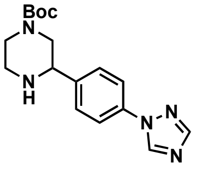

tert-Butyl 3-(4-(1H-1,2,4-triazol-1-yl)phenyl)piperazine-1-carboxylate was prepared according to the General Procedure A from 4-(pyridin-2-yl)benzaldehyde (86.5 mg, 0.500 mmol). Crude product was passed through Si-Trisamine (2 equiv) before NMR analysis.

Column conditions: EtOAc/MeOH (v/v = 25/1, + 0.25% Et_3N). Isolated: 95 mg (58%).

^{1}**H NMR** ($CDCl_3$, 400 MHz): δ 8.52 (s, 1H), 8.04 (s, 1H), 7.62–7.60 (m, 2H), 7.52–7.50 (m, 2H), 4.00 (m, 2H), 3.73 (dd, J = 10.6, 3.1 Hz, 1H), 3.06–3.02 (m, 1H), 2.93–2.81 (m, 2H), 2.70–2.69 (m, 1H), 2.09 (br. s, 1H), 1.43 (s, 9H).

^{13}**C NMR** ($CDCl_3$, 100 MHz): δ 154.70, 152.60, 141.82, 140.87, 136.44, 128.43 (2C), 120.13 (2C), 79.94, 59.62, (51.46, 50.51, 1C), 45.97, (44.32, 43.37, 1C), 28.47 (3C).

HRMS (ESI) m/z for $C_{17}H_{24}N_5O_2$ $[M+H]^+$ calcd 330.1925, found 330.1922.

4.2.3 Oxazepane products

3-(4-Bromophenyl)-1,4-oxazepane (3a)

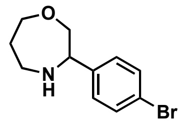

3-(4-Bromophenyl)-1,4-oxazepane was prepared according to the General Procedure A from 4-bromobenzaldehyde (92.0 mg, 0.500 mmol). The product was obtained in 69% yield (calculated from ^{1}H-NMR using 0.25 mmol (0.5 equiv) of mesitylene as internal standard) (88.0 mg, 0.345 mmol).

Column conditions: EtOAc (+ 0.25% Et_3N).

^{1}H NMR ($CDCl_3$, 400 MHz): δ 7.45–7.41 (m, 2H), 7.24–7.21 (m, 2H), 3.97 (ddd, J = 12.3, 6.3, 6.3 Hz, 1H), 3.92–3.86 (m, 2H), 3.81 (ddd, J = 12.9, 12.3, 6.5 Hz, 1H), 3.43 (m, 1H), 3.19 (ddd, J = 13.4, 4.9, 4.9 Hz, 1H), 2.99 (ddd, J = 13.9, 13.4, 6.9 Hz, 1H), 2.00–1.94 (m, 2H), 1.79 (br. s, 1H).

^{13}C NMR ($CDCl_3$, 100 MHz): δ 140.57, 131.73 (2C), 128.95 (2C), 121.33, 78.45, 70.09, 66.02, 46.36, 32.76.

HRMS (ESI) m/z for $C_{11}H_{15}BrNO$ $[M+H]^+$ calcd 256.0332, found 256.0338.

Methyl 4-(1,4-oxazepan-3-yl)benzoate (3b)

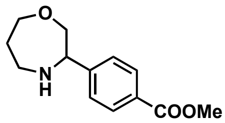

Methyl 4-(1,4-oxazepan-3-yl)benzoate was prepared according to the General Procedure A from methyl 4-formylbenzoate (82.0 mg, 0.500 mmol). The product was obtained in 69% yield (calculated from ^{1}H-NMR using 0.25 mmol (0.5 equiv) of mesitylene as internal standard) (81.1 mg, 0.345 mmol).

Column conditions: EtOAc (+ 0.25% Et_3N).

^{1}H NMR ($CDCl_3$, 400 MHz): δ 7.98–7.96 (m, 2H), 7.42–7.40 (m, 2H), 4.01–3.94 (m, 2H), 3.92–3.88 (m, 1H), 3.88 (s, 3H), 3.81 (ddd, J = 12.2, 6.5, 6.5 Hz, 1H), 3.46 (dd, J = 12.4, 9.5 Hz, 1H), 3.20 (ddd, J = 13.4, 4.9, 4.9 Hz, 1H), 3.00 (ddd, J = 13.5, 7.0, 7.0 Hz, 1H), 2.00–1.94 (m, 2H), 1.86 (br. s, 1H)

^{13}C NMR ($CDCl_3$, 100 MHz): δ 166.96, 146.68, 129.94 (2C), 129.40, 127.19 (2C), 78.32, 70.14, 66.32, 52.17, 46.36, 32.83.

HRMS (ESI) m/z for $C_{13}H_{18}NO_3$ $[M+H]^+$ calcd 236.1281, found 236.1286.

3-(3-Cyanophenyl)-1,4-oxazepane (3c)

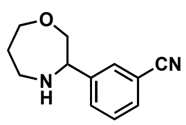

3-(3-Cyanophenyl)-1,4-oxazepanewas prepared according to the General Procedure A from 3-cyanobenzaldehyde (65.5 mg, 0.500 mmol). The product was obtained in 70% yield (calculated from ^{1}H-NMR using 0.25 mmol (0.5 equiv) of mesitylene as internal standard) (89.3 mg, 0.35 mmol).

^{1}H NMR (400 MHz, $CDCl_3$): δ 7.70 (t, J = 1.7 Hz, 1 H), 7.62–7.49 (m, 2 H), 7.42 (td, J = 7.7, 0.6 Hz, 1 H), 4,04–3.87 (m, 3 H), 3.82 (dt, J = 12.3, 6.4 Hz, 1 H), 3.47 (dd, J = 12.3, 9.2 Hz, 1 H), 3.19 (dt, J = 13.5, 4.9 Hz, 1 H), 3.09–2.95 (m, 1 H), 2.12–1.87 (m, 2 H), 1.76 (br s, NH).

^{13}C NMR (100 MHz, $CDCl_3$): δ 143.2, 131.7, 131.1, 130.9, 129.3, 118.8, 112.6, 77.8, 70.3, 65.3, 45.9, 32.9.

These spectral characteristics were identical to those previously reported.[11]

Ethyl 1,4-oxazepane-3-carboxylate (3d)

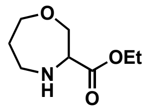

Ethyl 1,4-oxazepane-3-carboxylate was prepared according to the General Procedure A from ethyl 2-oxoacetate (512.0 mg, 0.500 mmol). The product was obtained in 23% yield (calculated from ^{1}H-NMR using 0.25 mmol (0.5 equiv) of mesitylene as internal standard) (19.8 mg, 0.115 mmol).

^{1}H NMR (400 MHz, $CDCl_3$): δ 4.19 (q, J = 7.1 Hz, 2H), 4.03 (dd, J = 12.6, 3.6 Hz, 1H), 3.92-3.59 (m, 4H), 3.28-3.12 (m, 1H), 2.94-2.80 (m, 1H), 2.26 (br s, NH), 1.93-1.78 (m, 2H), 1.27 (t, J = 7.1 Hz, 3H).

^{13}C NMR (100 MHz, $CDCl_3$): δ 172.5, 72.8, 70.8, 62.7, 61.3, 45.0, 33.4, 14.3.

These spectral characteristics were identical to those previously reported.[12]

3-(Quinolin-4-yl)-1,4-oxazepane (3e)

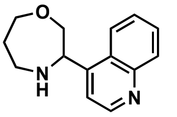

3-(Quinolin-4-yl)-1,4-oxazepane was prepared according to the General Procedure A from quinoline-4-carbaldehyde (78.5 mg, 0.500 mmol). Crude product was passed through Si-Trisamine (2 equiv) before NMR analysis.

Column conditions: EtOAc/MeOH (v/v = 20/1, + 0.25% Et_3N). Isolated: 79 mg (69%).

^{1}H NMR ($CDCl_3$, 400 MHz): δ 8.86 (d, J = 4.6 Hz, 1H), 8.16 (dd, J = 8.5, 1.3 Hz, 1H), 8.11 (dd, J = 8.4, 1.3 Hz, 1H), 7.69 (ddd, J = 8.4, 6.9, 1.3 Hz, 1H), 7.58 (d, J = 4.6 Hz, 1H), 7.56 (ddd, J = 8.5, 6.9, 1.3 Hz, 1H), 4.74 (dd, J = 9.1, 3.2 Hz, 1H), 4.10 (dd, J = 12.5, 3.2 Hz, 1H), 4.03 (ddd, J = 12.1, 6.3, 6.0 Hz, 1H), 3.87 (ddd, J = 12.1, 6.4, 6.0 Hz, 1H), 3.55 (dd, J = 12.5, 9.1 Hz, 1H), 3.30 (ddd, J = 13.5, 5.0, 5.0 Hz, 1H), 3.12 (ddd, J = 13.5, 6.8, 6.8 Hz, 1H), 2.08–2.02 (m, 3H).

^{13}C NMR ($CDCl_3$, 100 MHz): δ 150.51, 148.46, 147.00, 130.54, 129.24, 126.79, 126.21, 123.06, 118.87, 76.84, 70.38, 61.53, 46.63, 32.75.

HRMS (ESI) m/z for $C_{14}H_{17}N_2O$ $[M+H]^+$ calcd 229.1335, found 229.1329.

4.2.4 Diazepane products

***tert*-Butyl 3-(4-bromophenyl)-1,4-diazepane-1-carboxylate (4a)**

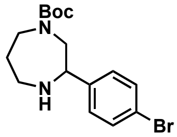

tert-Butyl 3-(4-bromophenyl)-1,4-diazepane-1-carboxylate was prepared according to the General Procedure A from 4-bromobenzaldehyde (92.0 mg, 0.500 mmol).

Column conditions: hexanes/EtOAc (v/v = 1/1, + 0.25% Et_3N). Isolated: 100 mg (56%, as a 50:50 mixture of rotamers).

11) Jackl, M. K..; Legnani, L.; Morandi, B.; Bode, J. W.: Continuous Flow Synthesis of Morpholines and Oxazepanes with Silicon Amine Protocol (SLAP) Reagents and Lewis Acid Facilitated Photoredox Catalysis. *Org. Lett.* **2017**, *19*, 4696–4699.

12) Vo, C.-V. T.; Luescher, M. U.; Bode, J. W.: SnAP Reagents for the One-Step Synthesis of Medium Ring Saturated N-Heterocycles from Aldehydes. *Nat. Chem.* **2014**, *6*, 311–314.

^{1}H NMR ($CDCl_3$, 400 MHz): δ 7.46–7.42 (m, 2H), 7.28–7.22 (m, 2H), 4.03 (dd, J = 13.9, 3.1 Hz, 1H × 0.50), 3.93 (ddd, J = 13.9, 6.7, 6.7 Hz, 1H × 0.50), 3.86–3.79 (m, 2H), 3.24–3.13 (m, 2H), 2.88 (dd, J = 13.6, 9.4 Hz, 1H × 0.50), 2.79–2.66 (m, 1H + 1H × 0.50), 2.08–1.90 (m, 1H), 1.86–1.79 (m, 2H), 1.49–1.48 (m, 9H).

^{13}C NMR ($CDCl_3$, 100 MHz): δ 155.71, 155.47, 141.63, 141.36, 131.80 (2C), 131.70 (2C), 128.74 (2C), 128.55 (2C), 121.28, 121.26, 79.69, 79.59, 64.65, 64.45, 56.78, 56.69, 47.13, 46.85, 46.25, 45.55, 30.03, 29.96, 28.69 (3C), 28.64 (3C).

HRMS (ESI) m/z for $C_{16}H_{24}BrN_2O_2$ $[M+H]^+$ calcd 355.1016, found 355.1015.

***tert*-Butyl 3-(2-chloro-4-fluorophenyl)-1,4-diazepane-1-carboxylate (4b)**

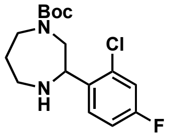

tert-Butyl 3-(2-chloro-4-fluorophenyl)-1,4-diazepane-1-carboxylate was prepared according to the General Procedure A from 2-chloro-4-fluorobenzaldehyde (79.0 mg, 0.500 mmol).

Column conditions: hexanes/EtOAc (v/v = 1/1, + 0.25% Et_3N). Isolated: 101 mg (61%, as a 63:37 mixture of rotamers).

^{1}H NMR ($CDCl_3$, 400 MHz): δ 7.54–7.46 (m, 1H), 7.09–7.06 (m, 1H), 6.99–6.92 (m, 1H), 4.25 (dd, J = 9.8, 3.2 Hz, 1H), 4.05 (dd, J = 13.7, 3.5 Hz, 1H × 0.37), 3.96 (dd, J = 14.4, 3.2 Hz, 1H × 0.63), 3.93–3.87 (m, 1H × 0.63), 3.72 (ddd, J = 14.1, 5.6, 5.6 Hz, 1H × 0.37), 3.34–3.15 (m, 2H), 2.88–2.70 (m, 2H), 2.05–1.87 (m, 1H), 1.84–1.76 (m, 1H), 1.71 (br. s, 1H), 1.47–1.46 (m, 9H).

^{13}C NMR ($CDCl_3$, 100 MHz): δ 162.76, 160.28, 155.51, 155.39, 136.06 (d, J = 3.5 Hz, 1C), 135.98 (d, J = 3.5 Hz, 1C), 133.58 (d, J = 10.0 Hz, 1C), 133.17 (d, J = 10.0 Hz, 1C), 129.23 (d, J = 8.7 Hz, 1C), 128.99 (d, J = 8.7 Hz, 1C), 116.87 (d, J = 24.4 Hz, 1C), 116.78 (d, J = 24.4 Hz, 1C), 114.51 (d, J = 20.7 Hz, 1C), 114.38 (d, J = 20.7 Hz, 1C), 79.76, 79.47, 60.54, 60.33, 55.40, 54.64, 47.65, 47.05, 46.26, 46.05, 30.21, 29.74, 28.60 (3C + 3C).

HRMS (ESI) m/z for $C_{16}H_{23}ClFN_2O_2$ $[M+H]^+$ calcd 329.1427, found 329.1427.

***tert*-Butyl 3-(4-(methoxycarbonyl)phenyl)-1,4-diazepane-1-carboxylate (4c)**

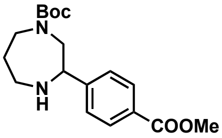

tert-Butyl 3-(4-(methoxycarbonyl)phenyl)-1,4-diazepane-1-carboxylate was prepared according to the General Procedure A from methyl 4-formylbenzoate (82.0 mg, 0.500 mmol).

Column conditions: hexanes/EtOAc (v/v = 1/1, + 0.25% Et_3N). Isolated: 86 mg (51%, as a 50:50 mixture of rotamers).

^{1}H NMR ($CDCl_3$, 400 MHz): δ 8.00–7.96 (m, 2H), 7.47–7.41 (m, 2H), 4.06 (dd, J = 13.7, 2.7 Hz, 1H × 0.50), 3.96–3.79 (m, 5H), 3.25–3.13 (m, 2H), 2.93–2.87 (m, 1H × 0.50), 2.82–2.67 (m, 1H + 1H × 0.50), 2.08–1.91 (m, 1H), 1.86–1.80 (m, 2H), 1.49–1.48 (m, 9H).

^{13}C NMR ($CDCl_3$, 100 MHz): δ 167.02, 166.97, 155.70, 155.44, 147.77, 147.50, 130.02 (2C), 129.94 (2C), 129.40, 129.33, 126.97 (2C), 126.80 (2C), 79.72, 79.60, 64.96, 64.76, 56.73, 56.63, 52.20, 52.16, 47.12, 46.82, 46.28, 45.60, 30.14, 30.06, 28.66 (3C), 28.62 (3C).

HRMS (ESI) m/z for $C_{18}H_{27}N_2O_4$ $[M+H]^+$ calcd 335.1965, found 335.1971.

***tert*-Butyl 3-(3-cyanophenyl)-1,4-diazepane-1-carboxylate (4d)**

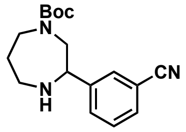

tert-Butyl 3-(3-cyanophenyl)-1,4-diazepane-1-carboxylate was prepared according to the General Procedure A from methyl 3-cyanobenzaldehyde (65.5 mg, 0.500 mmol).

Column conditions: hexanes/EtOAc (v/v = 1/1, + 0.25% Et_3N). Isolated: 108 mg (72%, as a 53:47 mixture of rotamers).

^{1}H NMR ($CDCl_3$, 400 MHz): δ 7.74–7.68 (m, 1H), 7.63–7.58 (m, 1H), 7.55–7.51 (m, 1H), 7.44–7.38 (m, 1H), 4.03 (dd, J = 13.9, 3.1 Hz, 1H × 0.53), 3.96–3.79 (m, 2H + 1H × 0.47), 3.23–3.11 (m, 2H), 2.89 (dd, J = 14.1, 9.7 Hz, 1H × 0.47), 2.81–2.68 (m, 1H + 1H × 0.53), 2.07–1.92 (m, 1H), 1.86–1.82 (m, 1H), 1.77 (br. s, 1H), 1.48 (m, 9H).

^{13}C NMR ($CDCl_3$, 100 MHz): δ 155.68, 155.32, 144.28, 144.00, 131.69, 131.69, 131.38, 131.18, 131.16, 130.53, 129.46, 129.35, 118.86 (2C), 112.75, 112.67, 79.85, 79.73, 63.98, 63.79, 56.40, 56.33, 46.88, 46.50, 46.33, 45.68, 30.23, 30.13, 28.65 (3C), 28.58 (3C).

HRMS (ESI) m/z for $C_{17}H_{24}N_3O_2$ $[M+H]^+$ calcd 302.1863, found 302.1870.

1-(*tert*-Butyl) 3-ethyl 1,4-diazepane-1,3-dicarboxylate (4e)

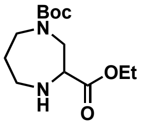

1-(*tert*-Butyl) 3-ethyl 1,4-diazepane-1,3-dicarboxylate was prepared according to the General Procedure A from ethyl 2-oxoacetate (51.0 mg, 0.500 mmol).

Column conditions: EtOAc (+ 0.25% Et_3N). Isolated: 51 mg (37%, as a 50:50 mixture of rotamers).

^{1}H NMR (400 MHz, $CDCl_3$): δ 4.26-4.11 (m, 2H and 1H × 0.5), 4.04 (dd, J = 14.3, 4.2 Hz, 1H × 0.5), 3.80–3.53 (m, 2H), 3.28–3.04 (m, 3H), 2.71–2.56 (m, 1H), 1.93–1.67 (m, 3H), 1.46 (s, 9H), 1.27 (m, 3H).

^{13}C NMR (100 MHz, $CDCl_3$): δ 172.4, 172.3, 155.6, 155.3, 79.9, 79.8, 61.4, 61.3, 61.3, 61.2, 51.8, 51.7, 46.9, 46.6, 45.9, 45.6, 30.7, 28.6, 14.3.

These spectral characteristics were identical to those previously reported.[12]

***tert*-Butyl 3-(quinolin-4-yl)-1,4-diazepane-1-carboxylate (4f)**

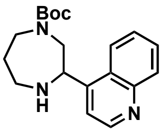

tert-Butyl 3-(quinolin-4-yl)-1,4-diazepane-1-carboxylatewas prepared according to the General Procedure A from quinoline-4-carbaldehyde (78.5 mg, 0.500 mmol).

Column conditions: EtOAc/MeOH (v/v = 20/1, + 0.25% Et_3N). Isolated: 98 mg (60%, as a 53:47 mixture of rotamers).

^{1}H NMR ($CDCl_3$, 400 MHz): δ 8.88–8.84 (m, 1H), 8.41 (d, J = 8.1 Hz, 1H × 0.47), 8.24 (d, J = 8.1 Hz, 1H × 0.53), 8.14–8.08 (m, 1H), 7.73 (m, 1H), 7.65 –7.52 (m, 2H), 4.70–4.62 (m, 1H), 4.36–4.32 (m, 1H × 0.53), 4.21–4.17 (m, 1H × 0.53), 4.07–4.01 (m, 1H × 0.53), 3.95–3.89 (m, 1H × 0.47), 3.35–3.28 (m, 1H), 3.24–3.12 (m, 1H), 2.92–2.74 (m, 1H + 2H × 0.47), 2.14–1.98 (m, 2H), 1.94–1.86 (m, 1H), 1.55–1.52 (m, 9H).

^{13}C NMR ($CDCl_3$, 100 MHz): δ 155.82, 155.27, 150.68, 150.42, 148.37 (2C), 148.02, 148.00, 130.56, 130.20, 129.32, 129.25, 127.11, 126.50, 126.28, 126.05, 123.67, 123.13, 118.24, 117.99, 80.23, 79.68, 60.39, 60.00, 56.33, 56.31, 47.23, 47.08, 46.57, 46.35, 30.19, 29.72, 28.79 (3C), 28.65 (3C).

HRMS (ESI) m/z for $C_{19}H_{26}N_3O_2$ $[M+H]^+$ calcd 328.2020, found 328.2026.

5. Automated Reductive Amination on the Console

5.1 General Procedure B for the Automated Reductive Amination on the Console

A 40 ml vial was charged with the chosen amine and carbonyl compound, dissolved in 5 ml of the appropriate solvent (see below) and connected to the console via screw cap. The Reductive Amination capsule was inserted into the console and the capsule holder was closed. The Reductive Amination program was selected either by scanning the RFID chip on the capsule or by manual selection, and then the desired sequence was selected manually. The reaction started automatically upon initiation of the sequence. After completion of the reaction sequence (3.5 to 5 hours) the solution in the vial was concentrated *in vacuo* to afford the pre-purified product without further purification.

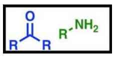 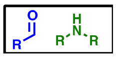 *Sequence A*: Used for primary amines and ketones or secondary amines and aldehydes. Amine (1 equiv.) and carbonyl compound (1–2 equiv) were dissolved in a mixture of DCM (4 ml) and HFIP (1 ml) prior connection to the console. *Sequence A* includes reduction and SCX purification. Time for reduction step: 2.5 hours

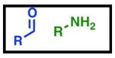 *Sequence B*: Used for primary amines and aldehydes. Amine (2 equiv) and aldehyde (1 equiv) were dissolved in a mixture of DCM (4 ml) and HFIP (1 ml) prior connection to the console. *Sequence B* includes reduction, PS-benzaldehyde purification and SCX purification. Time for reduction step: 3.0 hours

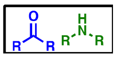 *Sequence C*: Used for secondary amines and ketones. Amine (1 equiv) and ketone (2 equiv) were dissolved in toluene (5 ml) prior connection to the console. *Sequence C* includes acid dissolution, reduction and SCX purification. Time for reduction step: 4.0 hours

Reductive amination capsule contents:

Compartment 1: Silica supported cyanoborohydride (2.00 equiv)
Compartment 2: Phenylacetic acid (2.00 equiv)
Compartment 3: SCX-2 (3.00 equiv)
Compartment 4: Polymer supported Benzaldehyde (3.00 equiv)

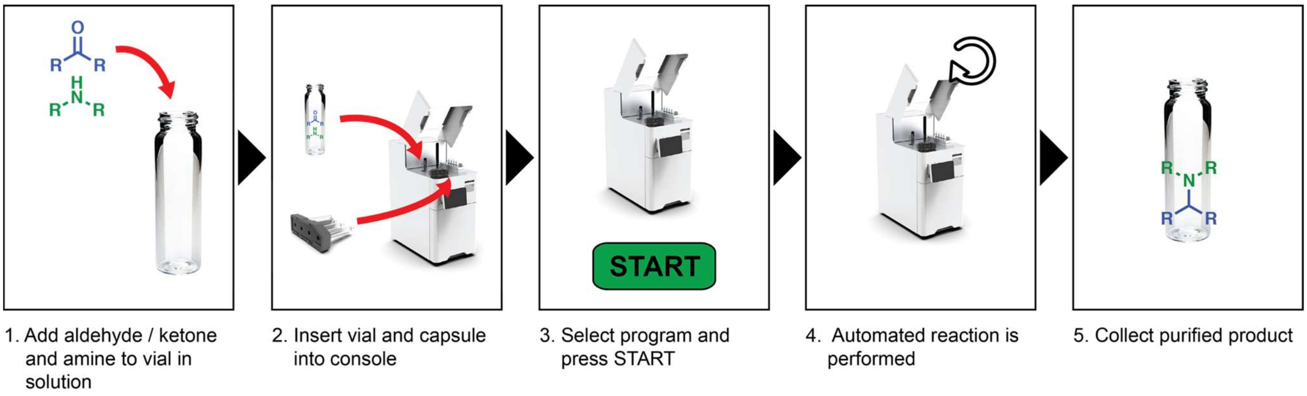

Fig. S2: 5 Steps to use the automated synthesizer for redutive aminations.

A video of the reaction setup is available at: https://youtu.be/a01CglvgUVQ

Description of console process

1) Imine formation / Reduction

In the first step the solution is circulated through cartridge compartment 1 (silica supported cyanoborohydride) at 2 mL/min at room temperature for several hours depending on the utilized Sequence.

Reductive Amination Type	Time for reduction step (h)
Sequence A: Aldehyde + primary amine	2.5
Sequence A: Aldehyde + secondary amine	2.5
Sequence B: Ketone + primary amine	3.0

Sequence C: Ketone + secondary amine	4.0

After the reduction is complete, compartment 1 is rinsed into the vial using CH_2Cl_2 (5 mL).
Note: For the reaction of ketones with secondary amines the reaction mixture is passed through compartment 2 (phenylacetic acid) before the start of the reduction. The compartment is then rinsed into the vial with CH_2Cl_2 (2mL).

2) Excess Amine Scavenger (only for aldehydes + primary amines)
The reaction mixture is passed through compartment 4 (polymer supported benzaldehyde) at 1 mL/min. The compartment is then washed with CH_2Cl_2.
3) SCX purification
The reaction mixture is loaded into compartment 3 (SCX) at 2 mL/min. The compartment is then washed with CH_2Cl_2 and MeOH.
4) Product release:
Compartment 3 is washed into the vial with 2.5 M DIPA/MeOH (15mL). The filtrate contains the reductive amination product.

5.2 Characterization of Reaction Products

Benzyl 4-(benzylamino)piperidine-1-carboxylate (5a)

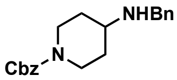

Benzyl 4-(benzylamino)piperidine-1-carboxylate was prepared according to the General Procedure B (Sequence A) from Benzylamine (0.0550 ml, 0.500 mmol) and N-Cbz-4-piperidone (117 mg, 0.500 mmol).

Isolated without any further purification as a colorless oil, 144 mg (89%).

^{1}H NMR ($CDCl_3$, 400 MHz): δ 7.39–7.33 (m, 9H), 7.32–7.23 (m, 1H), 5.16 (s, 2H), 4.22–4.15 (m, 2H), 3.85 (s, 2H), 2.94 (t, J = 12.4 Hz, 2H), 2.73 (tt, J = 10.0, 3.8 Hz, 1H), 1.98–1.81 (m, 2H), 1.44–1.28 (m, 3H).

^{13}C NMR ($CDCl_3$, 100 MHz): δ 155.28, 140.48, 136.92, 128.49 (4C), 128.04 (2C), 127.95, 127.84 (2C), 127.01, 67.03, 53.97, 50.86, 42.66 (2C), 32.41 (2C).

HRMS (ESI) m/z for $C_{20}H_{24}N_2NaO_2$ $[M+Na]^+$ calcd 347.1730, found 347.1731.

2-Morpholino-*N*-(thiophen-3-ylmethyl)ethan-1-amine (5b)

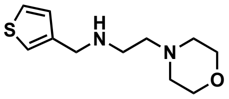

2-Morpholino-*N*-(thiophen-3-ylmethyl)ethan-1-amine was prepared according to the General Procedure B (Sequence A) from thiophene-3-carbaldehyde (0.0460 ml, 0.500 mmol) and 2-morpholinoethan-1-amine (0.131 ml, 1.00 mmol).

Isolated without any further purification as an orange oil, 85.0 mg (75%)

^{1}H NMR ($CDCl_3$, 400 MHz): δ 7.31–7.26 (m, 1H), 7.15–7.11 (m, 1H), 7.05 (d, J = 4.8 Hz, 1H), 3.83 (s, 2H), 3.69 (t, J = 4.7 Hz, 4H), 2.72 (t, J = 6.0 Hz, 2H), 2.50 (t, J = 6.0 Hz, 2H), 2.47–2.34 (m, 4H), 1.94 (bs, 1H).

^{13}C NMR ($CDCl_3$, 100 MHz): δ 141.53, 127.55, 125.72, 121.51, 67.01 (2C), 58.21, 53.70 (2C), 48.87, 45.30.

HRMS (ESI) m/z for $C_{11}H_{19}N_2OS$ $[M+H]^+$ calcd. 227.1213, found 227.1217.

***N*-(3-(1*H*-Imidazol-1-yl)propyl)-1,4-dioxaspiro[4.5]decan-8-amine (5c)**

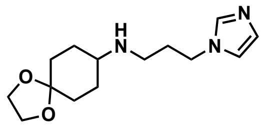

N-(3-(1*H*-Imidazol-1-yl)propyl)-1,4-dioxaspiro[4.5]decan-8-amine was prepared according to the General Procedure B (Sequence A) from 3-(1*H*-imidazol-1-yl)propan-1-amine (62.6 mg, 0.500 mmol) and 1,4-dioxaspiro[4.5]decan-8-one (78.0 mg, 0.500 mmol).

Isolated without any further purification as a colorless oil, 113 mg (82% + 3% dimethylacetal impurity)

^{1}H NMR ($CDCl_3$, 400 MHz): 7.43 (t, *J* = 1.2 Hz, 1H), 7.00 (t, *J* = 1.1 Hz, 1H), 6.88 (t, *J* = 1.3 Hz, 1H), 4.01 (t, *J* = 6.9 Hz, 2H), 3.89 (s, 4H), 2.54 (t, *J* = 6.7 Hz, 2H), 2.44 (tt, *J* = 9.9, 3.7 Hz, 1H), 1.86 (quin, *J* = 6.8 Hz, 2H), 1.82–1.75 (m, 3H), 1.75–1.68 (m, 2H), 1.50 (td, *J* = 12.6, 3.9 Hz, 2H), 1.39–1.30 (m, 2H).

^{13}C NMR ($CDCl_3$, 100 MHz): 137.17, 129.24, 118.85, 108.56, 64.25, 64.20, 55.13, 44.65, 43.52, 32.88 (2C), 31.78, 30.28 (2C).

HRMS (ESI) m/z for $C_{14}H_{24}N_3O_2$ $[M+H]^+$ calcd. 266.1863, found 266.1864

Benzyl 4-(cyclopropylamino)piperidine-1-carboxylate (5d)

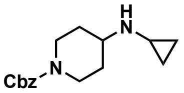

Benzyl 4-(cyclopropylamino)piperidine-1-carboxylate was prepared according to the General Procedure B (Sequence A) from Cyclopropylamine (0.0350 ml, 0.500 mmol) and N-Cbz-4-piperidone (117 mg, 0.500 mmol).

Isolated without any further purification as a colorless oil, 129 mg (94%).

^{1}H NMR ($CDCl_3$, 400 MHz): 7.40–7.30 (m, 5H), 5.15 (s, 2H), 4.21–4.04 (m, 2H), 2.96–2.89 (m, 2H), 2.79 (tt, *J* = 10.4, 3.9 Hz, 1H), 2.15 (tt, *J* = 6.6, 3.6 Hz, 1H), 1.97–1.90 (m, 2H), 1.72 (bs, 1H), 1.36–1.26 (m, 2H), 0.51–0.45 (m, 2H), 0.38–0.33 (m, 2H).

^{13}C NMR ($CDCl_3$, 100 MHz): 155.27, 136.92, 128.47 (2C), 127.93, 127.82 (2C), 67.01, 55.26, 42.86 (2C), 32.68 (2C), 28.01, 6.47 (2C).

HRMS (ESI) m/z for $C_{16}H_{23}N_2O_2$ $[M+H]^+$ calcd. 275.1754, found 275.1757.

2-Fluoro-5-((methyl(prop-2-yn-1-yl)amino)methyl)benzonitrile (5e)

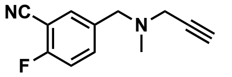

2-Fluoro-5-((methyl(prop-2-yn-1-yl)amino)methyl)benzonitrile was prepared according to the General Procedure B (Sequence A) from N-methylpropargylamine (0.0420 ml, 0.500 mmol) and 2-fluoro-5-formylbenzonitrile (149 mg, 1.00 mmol).

Isolated without any further purification as a pale yellow oil, 65 mg (61%).

^{1}H NMR ($CDCl_3$, 400 MHz): δ 7.66–7.56 (m, 2H), 7.17 (t, *J* = 8.6 Hz, 1H), 3.57 (s, 2H), 3.30 (d, *J* = 2.4 Hz, 2H), 2.32 (s, 3H), 2.30 (t, *J* = 2.4 Hz, 1H).

^{13}C NMR ($CDCl_3$, 100 MHz): δ 162.30 (d, $J_{C\text{-}F}$ = 258.3 Hz), 135.87 (d, $J_{C\text{-}F}$ = 3.7 Hz), 135.56 (d, $J_{C\text{-}F}$ = 8.2 Hz), 133.62, 116.31 (d, $J_{C\text{-}F}$ = 19.5 Hz), 113.98, 101.26 (d, $J_{C\text{-}F}$ = 15.5 Hz), 77.82, 73.86, 58.22, 44.93, 41.63.

^{19}F NMR ($CDCl_3$, 376 MHz): δ -108.99

HRMS (ESI) m/z for $C_{12}H_{11}FN_2Na$ $[M+Na]^+$ calcd. 225.0798, found 225.0796.

2-(4-(Benzo[*d*][1,3]dioxol-5-ylmethyl)piperazin-1-yl)pyrimidine (5f)

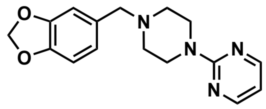

2-(4-(Benzo[*d*][1,3]dioxol-5-ylmethyl)piperazin-1-yl)pyrimidine was prepared according to the General Procedure B (Sequence A) from 2-(piperazin-1-yl)pyrimidine (0.0710 ml, 0.500 mmol) and Piperonal (150 mg, 1.00 mmol).

Isolated without any further purification as a white crystalline solid, 130 mg (86%).

^{1}H NMR ($CDCl_3$, 400 MHz): δ 8.30 (d, *J* = 4.8 Hz, 2H), 6.92–6.88 (m, 1H), 6.77 (d, *J* = 0.9 Hz, 2H), 6.47 (t, *J* = 4.7 Hz, 1H), 5.96 (s, 2H), 3.87–3.80 (m, 4H), 3.46 (s, 2H), 2.52–2.46 (m, 4H).

^{13}C NMR ($CDCl_3$, 100 MHz): δ 161.67, 157.67 (2C), 147.68, 146.66, 131.89, 122.22, 109.70, 109.48, 107.89, 100.89, 62.88, 52.85 (2C), 43.70 (2C).

HRMS (ESI) m/z for $C_{16}H_{19}N_4O_2$ $[M+H]^+$ calcd. 299.1503, found 299.1507

These spectral characteristics were identical to those previously reported.[13]

N-(Quinolin-4-ylmethyl)cyclopropanamine (5g)

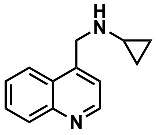

N-(Quinolin-4-ylmethyl)cyclopropanamine was prepared according to the General Procedure B (Sequence B) from quinoline-4-carbaldehyde (78.6 mg, 0.500 mmol) and cyclopropylamine (0.0690 ml, 1.00 mmol).

Isolated without any further purification as an orange oil, 90.0 mg (91%).

^{1}H NMR ($CDCl_3$, 400 MHz): δ 8.83 (d, *J* = 4.4 Hz, 1H), 8.10 (ddd, *J* = 15.4, 8.4, 1.3 Hz, 2H), 7.71 (ddd, *J* = 8.4, 6.8, 1.4 Hz, 1H), 7.57 (ddd, *J* = 8.3, 6.8, 1.3 Hz, 1H), 7.40 (d, *J* = 4.4 Hz, 1H), 4.31 (s, 2H), 2.28–2.21 (m, 1H), 0.53–0.42 (m, 4H).

^{13}C NMR ($CDCl_3$, 100 MHz): δ 150.13, 148.05, 146.07, 129.91, 129.20, 127.03, 126.58, 123.31, 120.02, 49.89, 30.62, 6.62 (2C).
HRMS (ESI) m/z for $C_{13}H_{15}N_2$ $[M+H]^+$ calcd. 199.1230, found 199.1228.

Benzyl 4-(1-((benzyloxy)carbonyl)piperidin-4-yl)piperazine-1-carboxylate (5h)

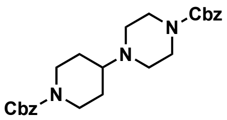

Benzyl 4-(1-((benzyloxy)carbonyl)piperidin-4-yl)piperazine-1-carboxylate was prepared according to the General Procedure B (Sequence C) from N-Cbz-4-piperidone (233 mg, 1.00 mmol) and benzyl piperazine-1-carboxylate (0.0960 ml, 0.500 mmol).

Isolated without any further purification as a pale yellow oil, 205 mg (94%)

^{1}H NMR ($CDCl_3$, 400 MHz): δ 7.41–7.31 (m, 10H), 5.16 (s, 2H), 5.15 (s, 2H), 4.34–4.16 (m, 2H), 3.57–3.49 (m, 4H), 2.90–2.74 (m, 2H), 2.59–2.51 (m, 4H), 2.45 (tt, *J* = 11.3, 3.5 Hz, 1H), 1.88–1.74 (m, 2H), 1.52–1.38 (m, 2H).

^{13}C NMR ($CDCl_3$, 100 MHz): δ 155.17, 155.15, 136.81, 136.74, 128.50 (4C), 128.03, 128.01, 127.91 (2C), 127.89 (2C), 67.12, 67.10, 61.82, 48.84 (2C), 44.17 (2C), 43.45 (2C), 28.08 (2C).

HRMS (ESI) m/z for $C_{25}H_{32}N_3O_4$ $[M+H]^+$ calcd. 438.2387, found 438.2387.

13) Huang, H.; Kang, J. Y.: Oxidation-Reduction Condensation of Diazaphosphites for Carbon-Heteroatom Bond Formation Based on Mitsunobu Mechanism. *Org. Lett.* **2017**, 19, 544–547.

(6-(4-(((3-(1*H*-Imidazol-1-yl)propyl)amino)methyl)phenyl)pyridin-2-yl)methanol (5i)

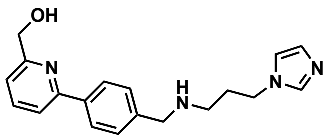

(6-(4-(((3-(1*H*-Imidazol-1-yl)propyl)amino)methyl)phenyl)pyridin-2-yl)methanol was prepared according to the General Procedure B (Sequence B) from 4-(6-(hydroxymethyl)pyridin-2-yl)benzaldehyde (107 mg, 0.500 mmol) and 3-(1*H*-imidazol-1-yl)propan-1-amine (0.119 ml, 1.00 mmol).

Isolated without any further purification as a colorless oil, 135 mg (84%).

^{1}**H NMR** ($CDCl_3$, 400 MHz): δ 7.98 (d, *J* = 7.9 Hz, 2H), 7.74 (t, *J* = 7.7 Hz, 1H), 7.62 (d, *J* = 7.8 Hz, 1H), 7.45 (s, 1H), 7.40 (d, *J* = 7.9 Hz, 2H), 7.22 (d, *J* = 7.6 Hz, 1H), 7.03 (s, 1H), 6.89 (s, 1H), 4.82 (s, 2H), 4.05 (t, *J* = 6.9 Hz, 2H), 3.81 (s, 2H), 2.62 (t, *J* = 6.8 Hz, 2H), 1.93 (quin, *J* = 6.8 Hz, 2H).

^{13}**C NMR** ($CDCl_3$, 100 MHz): δ 159.13, 155.82, 141.21, 137.83, 137.44, 137.17, 129.31, 128.46 (2C), 126.99 (2C), 118.88, 118.81, 118.73, 64.08, 53.59, 45.68, 44.69, 31.34.

HRMS (ESI) m/z for $C_{19}H_{23}N_4O$ $[M+H]^+$ calcd. 323.1866, found 323.1870.

Benzyl 4-(4-phenylbutan-2-yl)piperazine-1-carboxylate (5j)

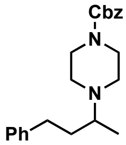

Benzyl 4-(4-phenylbutan-2-yl)piperazine-1-carboxylate was prepared according to the General Procedure B (Sequence C) from 4-phenylbutan-2-one (0.150 ml, 1.00 mmol) and benzyl piperazine-1-carboxylate (0.0960 ml, 0.500 mmol).

Isolated after filtration on silica pad (EtOAc) as a colorless oil, 84.0 mg (48%)

^{1}**H NMR** ($CDCl_3$, 400 MHz): δ 7.44–7.26 (m, 7H), 7.25–7.17 (m, 3H), 5.17 (s, 2H), 3.62–3.48 (m, 4H), 2.77–2.51 (m, 5H), 2.50–2.36 (m, 2H), 1.87 (ddt, *J* = 13.2, 8.8, 6.5 Hz, 1H), 1.60 (ddt, *J* = 13.8, 9.1, 7.0 Hz, 1H), 1.01 (d, *J* = 6.6 Hz, 3H).

^{13}**C NMR** ($CDCl_3$, 100 MHz): δ 155.26, 142.53, 136.83, 128.50 (2C), 128.44 (2C), 128.32 (2C), 127.99, 127.89 (2C), 125.73, 67.05, 58.37, 47.88 (2C), 44.31 (2C), 35.41, 32.90, 13.81.

HRMS (ESI) m/z for $C_{22}H_{28}N_2NaO_2$ $[M+Na]^+$ calcd. 375.2043, found 375.2042.

2-((Benzyl(methyl)amino)methyl)-6-methoxyphenol (5k)

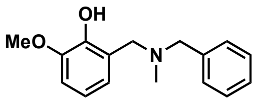

2-((Benzyl(methyl)amino)methyl)-6-methoxyphenol was prepared according to the General Procedure B (Sequence A) from N-methylbenzylamine (0.0650 ml, 0.500 mmol) and 2-hydroxy-3-methoxybenzaldehyde (152 mg, 1.00 mmol).

Isolated without any further purification as a yellow-orange solid, 128 mg (99%).

^{1}**H NMR** ($CDCl_3$, 400 MHz): δ 7.41–7.29 (m, 5H), 6.84 (d, *J* = 8.1 Hz, 1H), 6.77 (t, *J* = 7.8 Hz, 1H), 6.64 (d, *J* = 7.5 Hz, 1H), 3.91 (s, 3H), 3.78 (s, 2H), 3.64 (s, 2H), 2.25 (s, 3H).

^{13}**C NMR** ($CDCl_3$, 100 MHz): δ 147.95, 147.13, 136.84, 129.29 (2C), 128.62 (2C), 127.67, 122.06, 120.53, 118.72, 110.92, 61.70, 60.67, 55.86, 41.19.

HRMS (ESI) m/z for $C_{16}H_{20}NO_2$ $[M+H]^+$ calcd. 258.1489, found 258.1490.

***tert*-Butyl 4-(4-(pyrimidin-2-yl)piperazin-1-yl)piperidine-1-carboxylate (5l)**

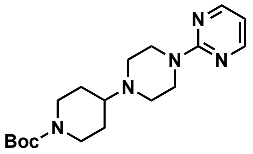

tert-Butyl 4-(4-(pyrimidin-2-yl)piperazin-1-yl)piperidine-1-carboxylate was prepared according to the General Procedure B (Sequence C) from N-Boc-4-piperidone (199 mg, 1.00 mmol) and 2-(piperazin-1-yl)pyrimidine (0.0710 ml, 0.500 mmol).

Isolated without any further purification as a white solid, 145 mg (83%)

^{1}H NMR ($CDCl_3$, 400 MHz): δ 8.29 (d, J = 4.7 Hz, 2H), 6.47 (t, J = 4.7 Hz, 1H), 4.25–4.04 (m, 2H), 3.84–3.77 (m, 4H), 2.78–2.64 (m, 1H), 2.63–2.58 (m, 4H), 2.42 (tt, J = 11.3, 3.5 Hz, 1H), 1.85–1.77 (m, 2H), 1.48–1.36 (m, 2H), 1.44 (s, 9H).

^{13}C NMR ($CDCl_3$, 100 MHz): δ 161.58, 157.69 (2C), 154.69, 109.82, 79.44, 62.03, 49.00 (2C), 43.97 (2C), 43.34 (2C), 28.44 (3C), 28.22 (2C).

HRMS (ESI) m/z for $C_{18}H_{30}N_5O_2$ $[M+H]^+$ calcd. 348.2394, found 348.2397.

6. Combination of Heterocycle Formation and Reductive Amination on the Console

6.1 General Procedure C

A 40 mL vial was charged with the aldehyde (1.00 equiv.) and loaded onto the console. The vial was placed on the console and connected to the console via screw cap. The heterocycle formation capsule with the desired SnAP reagent was inserted into the console and the capsule holder was closed. The reaction program was selected, either by scanning the RFID chip on the capsule or by manual selection. The reaction started automatically upon initiation of the sequence. After completion of the reaction sequence, the vial was transferred to a rotary evaporator using a vial adapter and concentrated to dryness. Amine (1.00 equiv.) was added and vial was returned to the console, a reductive amination capsule was inserted in the same manner as for the previous setup and the loaded reaction sequence was started. Purification was then carried out according to the substrate.

Heterocycle forming capsule contents:

Compartment 1: Top: 4 Å molecular sieves powder (100 mg). Bottom: SnAP resin (1.00 equiv).
Compartment 2: Top: 2,6-Lutidine triflate (1.00 equiv). Bottom: Copper (II) triflate (1.00 equiv),
Compartment 3: Polystyrene support tosylic acid (2.50 equiv), SCX-2 (4.00 equiv)
Compartment 4: Silica (5.00 g)

Reductive amination capsule contents:

Compartment 1: Silica supported cyanoborohydride (2.00 equiv)
Compartment 2: Empty
Compartment 3: SCX-2 (4.00 equiv)
Compartment 4: Polymer supported Benzaldehyde (3.00 equiv)

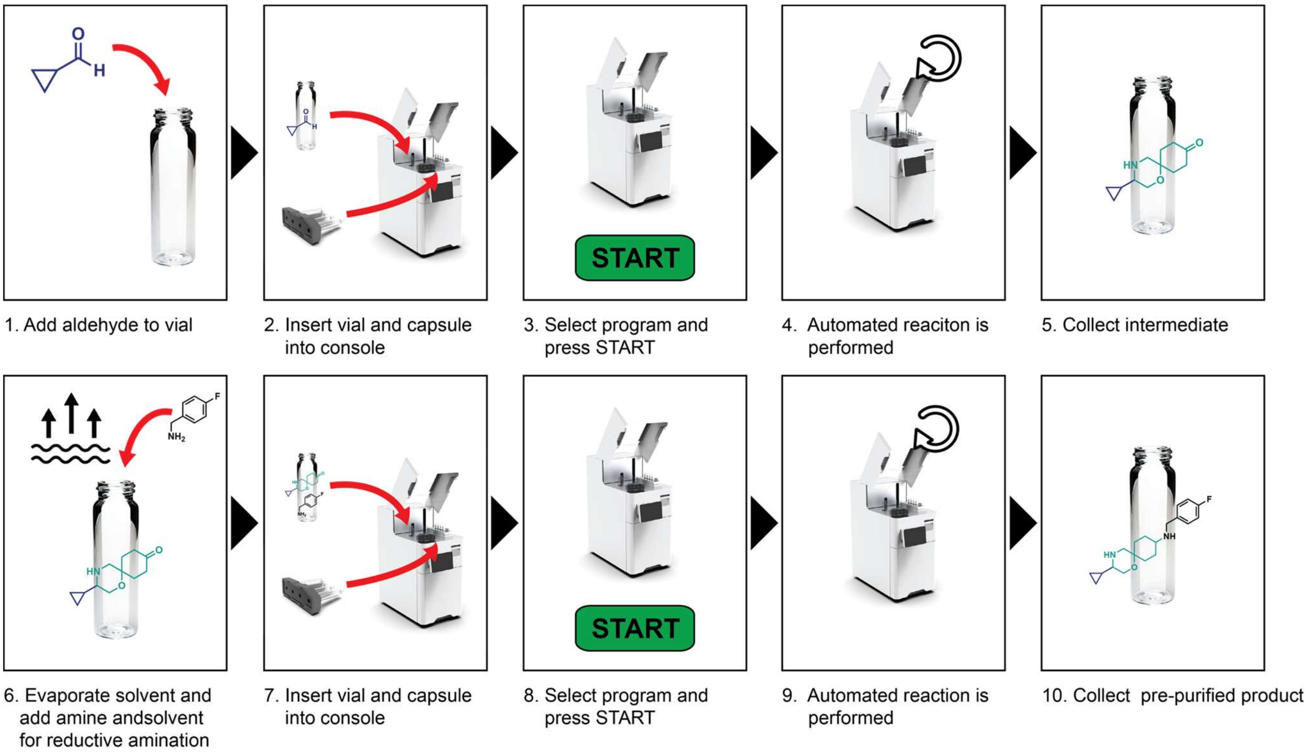

Fig. S3: 10 Steps to use the automated synthesizer for the combination of N-heterocycle synthesis and reductive amination

Description of console process

Step 1

1) Imine formation:

In the first step the neat aldehyde is dissolved in anhydrous (trifluoromethyl)benzene (4.5 mL). The solution is then circulated through cartridge compartment 1 (SnAP reagent) at 1 mL/min for 5 hours. Compartment 1 is heated at 80 °C and the reaction vial is heated at 80 °C. After the imine formation is complete, compartment 1 is rinsed into the vial with anhydrous (trifluoromethyl)benzene (2.5 mL).

2) Cyclization:

Anhydrous HFIP (7.0 mL) is added via compartment 1 into the reaction vial. The solution of imine in anhydrous (trifluoromethyl)benzene and HFIP is circulated through compartment 2 at 2 mL/min for 4 hours. Compartment 2 is heated at 40 °C and the reaction vial is heated at 37 °C. After the reaction time, compartment 2 is rinsed with anhydrous (trifluoromethyl)benzene (2 mL).

3) Purification:

The reaction mixture is loaded into compartment 4 (Silica). The filtrate is then loaded into compartment 3 (SCX) and the filtrate is discarded to waste.

Compartment 4 is washed with MeOH (12 mL) and the filtrate is loaded again into compartment 3. The filtrate from compartment 4 is discarded to waste. This step is repeated once more.

Compartment 3 is washed with MeOH (12 mL) and the filtrate is discarded to waste. This step is repeated once more.

Compartment 3 is washed with (trifluoromethyl)benzene (10 mL) and the filtrate is discarded to waste. This step is repeated once more.

Compartment 3 is washed with acetone (10 mL) and the filtrate is discarded to waste.

Acetone (10 mL) is added via compartment 3 into the reaction vial. The solution is circulated through compartment 3 at 1 mL/min for 6 h. Compartment 3 is heated to 50 °C and the reaction vial is heated to 50 °C. The filtrate is discarded to waste.

4) Product release:

Compartment 3 is washed into the vial with 2.5 M DIPA/THF (20 mL). The filtrate contains the N-heterocycle product.

Step 2

5) Imine formation

Anhydrous (trifluoromethyl)benzene (4 mL) and HFIP (1 mL) are added via compartment 1 The solution is circulated through cartridge compartment 1 (silica supported cyanoborohydride) at 2 mL/min at room temperature for 3 hours. After the reduction is complete, compartment 1 is rinsed into the vial using (trifluoromethyl)benzene (5 mL).

6) Excess Amine Scavenger

The reaction mixture is passed through compartment 4 (polymer supported benzaldehyde) at 1 mL/min. The compartment is then washed with (trifluoromethyl)benzene (5 mL)

7) **SCX purification**

The reaction mixture is loaded into compartment 3 (SCX) at 2 mL/min. The compartment is then washed with (trifluoromethyl)benzene and MeOH.

8) Product release:

Compartment 3 is washed into the vial with 2.5 M DIPA/MeOH (15mL). The filtrate contains the reductive amination product.

5.2 Characterization of Reaction Products

***N*-Cyclopropyl-3-phenyl-1-oxa-4-azaspiro[5.5]undecan-9-amine (6a)**

Benzaldehyde (42 mg, 0.40 mmol) and cyclopropylamine (23 mg, 0.40 mmol) were reacted according to the General Procedure C and purified using chromatography (1:9 MeOH : MeCN) to afford the title compound (32 mg, 0.12 mmol, 30% yield) as a 2:1 mixture of diastereomers. These diastereomers were resolved using preparative HPLC.

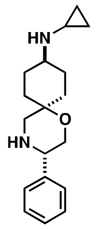

(3*S,6*s**,9*R**)-*N*-cyclopropyl-3-phenyl-1-oxa-4-azaspiro[5.5]undecan-9-amine**

^{1}H NMR ($CDCl_3$, 400 MHz): δ 7.44–7.39 (m, 2H), 7.38–7.27 (m, 3H), 3.88 (dd, J = 10.3, 3.8 Hz, 1H), 3.62 (dd, J = 11.6, 3.8 Hz, 1H), 3.55 (dd, J = 11.6, 10.3 Hz, 1H), 2.90 (d, J = 11.5 Hz, 1H), 2.89 (m, 1H), 2.82 (d, J = 11.5 Hz, 1H), 2.79 (m, 1H), 2.30 (m, 1H), 1.95–1.81 (m, 2H), 1.76–1.62 (m, 2H), 1.52–1.33 (m, 2H), 1.17 (td, J = 14.2, 3.5 Hz, 1H), 0.57 (m, 4H). (Exchangeable 2 x NH not observed).

^{13}C NMR ($CDCl_3$, 100 MHz): δ 140.6, 128.5 (2C), 127.7, 127.1 (2C), 69.9, 66.9, 60.8, 57.6, 55.9, 35.1, 27.9, 26.9, 26.7, 26.5, 5.6, 5.5.

HRMS (ESI) m/z for $C_{18}H_{26}N_2O$ $[M+Na]^+$ calcd 309.1937, found 309.1941.

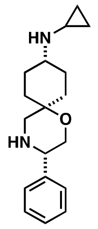

(3*S,6*r**,9*S**)-3-cyclopropyl-*N*-(4-fluorobenzyl)-1-oxa-4-azaspiro[5.5]undecan-9-amine**

^{1}H NMR ($CDCl_3$, 400 MHz): δ 7.45–7.40 (m, 2H), 7.38–7.26 (m, 3H), 3.89 (dd, J = 10.3, 3.8 Hz, 1H), 3.71 (dd, J = 11.4, 10.3 Hz, 1H), 3.62 (dd, J = 11.4, 3.8 Hz, 1H), 3.25 (d, J = 11.7 Hz, 1H), 2.78 (m, 1H), 2.74 (dd, J = 11.7, 1.1 Hz, 1H), 2.57

(m, 1H), 2.13 (m, 1H), 2.03–1.90 (m, 2H), 1.71 (m, 1H), 1.65–1.49 (m, 3H), 1.40–1.22 (m, 3H), 0.55–0.46 (m, 2H), 0.37–0.34 (m, 2H).

^{13}C NMR ($CDCl_3$, 100 MHz): δ 140.8, 128.4 (2C), 127.7, 127.2 (2C), 71.8, 67.5, 61.0, 56.4, 52.5, 34.9, 28.9, 28.7, 28.6, 26.6, 6.4, 6.3.

HRMS (ESI) m/z for $C_{18}H_{26}N_2O$ $[M+H]^+$ calcd 287.2118, found 287.2120.

3-Cyclopropyl-*N*-(4-fluorobenzyl)-1-oxa-4-azaspiro[5.5]undecan-9-amine (6b)

Cyclopropane carboxaldehyde (21 mg, 0.30 mmol) and 4-Fluorobenzylamine (38 mg, 0.3 mmol) were reacted according to the General Procedure C and purified using chromatography (1:1 MeOH : ethyl acetate) to afford the title compound (15 mg, 0.05 mmol, 17% yield) as a 2:1 mixture of diastereomers. These diastereomers were resolved using preparative HPLC.

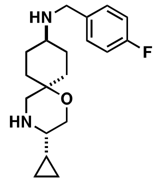

(3*S,6*s**,9*R**)-3-cyclopropyl-*N*-(4-fluorobenzyl)-1-oxa-4-azaspiro[5.5]undecan-9-amine**

^{1}H NMR ($CDCl_3$, 400 MHz): δ 7.34–7.29 (m, 2H), 7.08–6.95 (m, 2H), 3.86 (s, 2H), 3.66 (dd, J = 11.7, 3.6 Hz, 1H), 3.52 (dd, J = 11.6, 10.4 Hz, 1H), 2.72 – 2.52 (m, 4H), 1.94 (td, J = 10.0, 3.6 Hz, 1H), 1.84–1.73 (m, 2H), 1.66–1.57 (m, 2H), 1.45 (m, 1H), 1.38–1.20 (m, 2H), 1.06 (td, J = 14.0, 3.5 Hz, 1H), 0.71 (m, 1H), 0.53–0.42 (m, 2H), 0.28–0.13 (m, 2H). (Exchangeable 1 x NH not observed).

^{13}C NMR ($CDCl_3$, 100 MHz): δ 162.13 (d, J = 245 Hz, 1C), 143.3, 130.2 (d, J = 8.1 Hz, 2C), 115.4 (d, J = 21.3, 2C), 69.8, 64.9, 60.7, 56.1, 55.2, 49.5, 34.8, 26.9, 26.7 (2C), 12.4, 2.1 (2C)

HRMS (ESI) m/z for $C_{19}H_{27}FN_2O$ $[M+H]^+$ calcd 319.2180, found 319.2184.

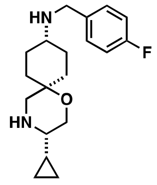

(3*S,6*r**,9*S**)-3-cyclopropyl-*N*-(4-fluorobenzyl)-1-oxa-4-azaspiro[5.5]undecan-9-amine**

^{1}H NMR ($CDCl_3$, 400 MHz): δ 7.36–7.26 (m, 2H), 7.02 (m, 2H), 3.78 (s, 2H), 3.69–3.58 (m, 2H), 3.14 (d, J = 11.8 Hz, 1H), 2.69 (m, 1H), 2.54 (d, J = 11.8 Hz, 1H), 2.38 (m, 1H), 1.96–1.83 (m, 3H), 1.71–1.61 (m, 2H), 1.60–1.48 (m, 1H), 1.46 (ddd, J = 13.3, 12.3, 4.0 Hz, 1H), 1.37–1.23 (m, 3H), 0.68 (m, 1H), 0.53–0.43 (m, 2H), 0.20–0.19 (m, 2H).

^{13}C NMR ($CDCl_3$, 126 MHz): δ 161.9 (d, J = 244 Hz), 136.2, 129.5 (d, J = 8 Hz, 2C), 115.1 (d, J = 21 Hz, 2C), 71.7, 65.9, 61.1, 55.1, 52.4, 50.8, 34.5, 28.6, 28.27, 26.6, 12.7, 2.08, 1.96.

HRMS (ESI) m/z for $C_{19}H_{27}FN_2O$ $[M+H]^+$ calcd 319.2180, found 319.2179.

3-(4-(1*H*-1,2,4-Triazol-1-yl)phenyl)-*N*-(2,2,2-trifluoroethyl)-1-oxa-4-azaspiro[5.5]undecan-9-amine (6c)

4-(1*H*-1,2,4-Triazol-1-yl)benzaldehyde (52 mg, 0.30 mmol) and 2,2,2-trifluoroethan-1-amine (30 mg, 0.30 mmol) were reacted according to the General Procedure C and purified using chromatography (1:9 MeOH : ethyl acetate) to afford the title compound (40 mg, 0.10 mmol, 34% yield) as a 2:1 mixture of diastereomers. These diastereomers were then resolved using preparative HPLC.

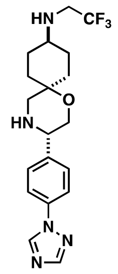

(3*S,6*s**,9*R**)-3-(4-(1*H*-1,2,4-triazol-1-yl)phenyl)-*N*-(2,2,2-trifluoroethyl)-1-oxa-4-azaspiro[5.5]undecan-9-amine**

^{1}H NMR ($CDCl_3$, 400 MHz): δ 8.58 (s, 1H), 8.12 (s, 1H), 7.69 (d, J = 8.6 Hz, 2H), 7.60 (d, J = 8.6 Hz, 2H), 4.06 (m, 1H), 3.80–3.68 (m, 2H), 3.24 (q, J = 9.4 Hz, 2H), 2.88 (d, J = 12.1 Hz, 1H), 2.84–2.72 (m, 2H), 2.60 (m, 1H), 1.83–1.65 (m, 3H), 1.54 (m, 1H), 1.41–1.25 (m, 2H), 1.15 (m, 1H). (Exchangeable 2 x NH not observed).

^{13}C NMR ($CDCl_3$, 100 MHz): δ 152.6, 140.9, 140.8, 136.4, 128.5 (2C), 125.6 (q, J = 279 Hz, 1C), 120.1 (2C), 70.2, 66.8, 60.2, 56.1, 55.7, 47.7 (q, J = 31 Hz, 1C), 35.0, 27.7, 27.5, 26.8.

HRMS (ESI) m/z for $C_{19}H_{24}F_3N_5O$ $[M+Na]^+$ calcd 418.1825, found 418.1822.

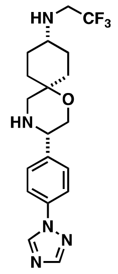

(3*S,6*r**,9*S**)-3-(4-(1*H*-1,2,4-triazol-1-yl)phenyl)-*N*-(2,2,2-trifluoroethyl)-1-oxa-4-azaspiro[5.5]undecan-9-amine**

^{1}H NMR ($CDCl_3$, 400 MHz): δ 8.55 (s, 1H), 8.12 (s, 1H), 7.66 (d, J = 8.7 Hz, 2H), 7.58 (d, J = 8.5 Hz, 2H), 3.96 (dd, J = 9.0, 5.0 Hz, 1H), 3.70–3.59 (m, 2H), 3.23 (q, J = 9.5 Hz, 2H), 3.20 (d, J = 11.7 Hz, 2H), 2.82 (m, 1H), 2.78 (d, J = 11.7 Hz, 1H), 1.97–1.83 (m, 2H), 1.80–1.69 (m, 2H), 1.52 (ddd, J = 13.6, 10.1, 4.0 Hz, 1H), 1.34 (m, 2H). (Exchangeable 2 x NH not observed).

^{13}C NMR ($CDCl_3$, 126 MHz): δ 152.6, 141.1, 140.8, 136.4, 128.6 (2C), 125.62 (q, J = 278Hz, 1C), 120.1 (2C), 71.7, 67.4, 60.4, 54.2, 53.0, 48.07 (q, J = 31 Hz, 1C), 33.9, 28.1, 27.8, 25.7.

HRMS (ESI) m/z for $C_{19}H_{24}F_3N_5O$ $[M+H]^+$ calcd 396.2006, found 396.2007.

2-Methoxy-*N*-(4-(morpholin-3-yl)benzyl)ethan-1-amine (6d)

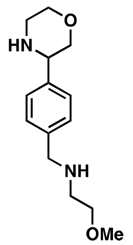

4-(Diethoxymethyl)benzaldehyde (104 mg, 0.50 mmol) and 2-methoxyethan-1-amine (38 mg, 0.50 mmol) were reacted according to the General Procedure C and purified using chromatography (94:5:1 ethyl acetate : MeOH : NEt_3) to afford the title compound (38 mg, 0.15 mmol, 30% yield).

^{1}H NMR ($CDCl_3$, 400 MHz): δ 7.35 (d, J = 8.2 Hz, 2H), 7.31 (d, J = 8.2 Hz, 2H), 3.91 (dd, J = 10.2, 3.2 Hz, 1H), 3.90–3.86 (m, 1H), 3.84–3.79 (m, 3H), 3.66 (td, J = 11.3, 2.7 Hz, 1H), 3.52 (t, J = 5.0 Hz, 2H), 3.43–3.37 (m, 1H), 3.36 (s, 3H), 3.14 (td, J = 11.6, 3.2 Hz, 1H), 3.01 (dt, J = 11.8, 2.0 Hz, 1H), 2.81 (t, J = 5.1 Hz, 2H), 1.86 (br s, 2H).

^{13}C NMR ($CDCl_3$, 100 MHz): δ 139.9, 139.2, 128.3 (2C), 127.2 (2C), 73.7, 72.0, 67.2, 60.3, 58.8, 53.7, 48.8, 46.6.

HRMS (ESI) m/z for $C_{14}H_{22}N_2O_2$ $[M+H]^+$ calcd 251.1754, found 251.1757.

N-(4-(1,4-Oxazepan-3-yl)benzyl)-3-(1H-imidazol-1-yl)propan-1-amine (6e)

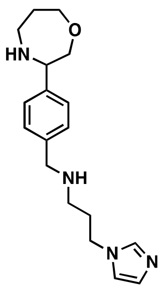

4-(Diethoxymethyl)benzaldehyde (104 mg, 0.5 mmol) and 3-(1H-imidazol-1-yl)propan-1-amine (63 mg, 0.5 mmol) were reacted according to the General Procedure C and purified using chromatography (89:10:1 ethyl acetate : MeOH : NEt_3) to afford the title compound (33 mg, 0.10 mmol, 21% yield).

^{1}H NMR ($CDCl_3$, 400 MHz): δ 7.45 (s, 1H), 7.34 (d, J = 8.4 Hz, 2H), 7.27 (d, J = 8.4 Hz, 2H), 7.06 (s, 1H), 6.90 (s, 1H), 4.06 (t, J = 6.9 Hz, 2H), 4.04–3.91 (m, 3H), 3.90–3.82 (m, 1H), 3.75 (s, 2H), 3.51 (dd, J = 13.0, 10.4 Hz, 1H), 3.25 (dt, J = 13.4, 4.9 Hz, 1H), 3.02 (dt, J = 13.4, 7.1 Hz, 1H), 2.62 (t, J = 6.7 Hz, 2H), 2.05–1.98 (m, 2H), 2.00–1.86 (m, 2H), 1.66 (br s, 2H).

^{13}C NMR ($CDCl_3$, 100 MHz): δ 140.2, 139.5, 137.2, 129.5, 128.3 (2C), 127.2 (2C), 118.8, 78.7, 69.9, 66.5, 53.6, 46.5, 45.7, 44.6, 32.6, 31.4.

HRMS (ESI) m/z for $C_{18}H_{26}N_4O$ $[M+H]^+$ calcd 315.2179, found 315.2181.

N-(4-(Morpholin-3-yl)benzyl)cyclopropanamine (6f)

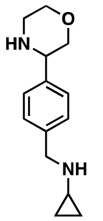

4-(Diethoxymethyl)benzaldehyde (104 mg, 0.5 mmol) and cyclopropylamine (29 mg, 0.5 mmol) were reacted according to the General Procedure C and purified using chromatography (94:5:1 ethyl acetate : MeOH : NEt_3) to afford the title compound (24 mg, 0.10 mmol, 21% yield).

^{1}H NMR ($CDCl_3$, 500 MHz): δ 7.35 (d, J = 8.0 Hz, 2H), (d, J = 8.0 Hz, 2H), 3.92 (dd, J = 10.3, 3.4 Hz 1H), 3.90–3.86 (m, 1H), 3.85–3.79 (m, 3H), 3.66 (td, J = 11.3, 2.6 Hz, 1H), 3.40 (dd, J = 11.0, 10.1 Hz, 1H), 3.14 (td, J = 11.6, 3.3 Hz, 1H), 3.06–2.97 (m, 1H), 2.17 (m, 1H), 1.90 (br s, 2H), 0.48–0.42 (m, 2H), 0.42–0.36 (m, 2H).

^{13}C NMR ($CDCl_3$, 126 MHz): δ 140.2, 139.1, 128.3 (2C), 127.2 (2C), 73.7, 67.2, 60.3, 53.4, 46.6, 30.1, 6.5 (2C).

HRMS (ESI) m/z for $C_{14}H_{20}N_2O$ $[M+H]^+$ calcd 233.1648, found 233.1652.

4-(9-(isopropylamino)-1-oxa-4-azaspiro[5.5]undecan-3-yl)benzonitrile (6g)

4-formylbenzonitrile (65 mg, 0.5 mmol) and propan-2-amine (30 mg, 0.5 mmol) were reacted according to the General Procedure C and purified using preparative HPLC (67 mg, 43% yield, dr:11:9).

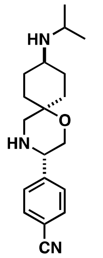

4-((3S*,6s*,9R*)-9-(isopropylamino)-1-oxa-4-azaspiro[5.5]undecan-3-yl)benzonitrile

^{1}H NMR ($CDCl_3$, 400 MHz): δ 7.66 – 7.58 (m, 2H), 7.57 – 7.50 (m, 2H), 3.93 (dd, J = 10.3, 3.7 Hz, 1H), 3.59 (dd, J = 11.6, 3.7 Hz, 1H), 3.48 (dd, J = 11.6, 10.2 Hz, 1H), 3.10 – 3.00 (m, 1H), 2.91 – 2.75 (m, 3H), 2.69 – 2.58 (m, 1H), 1.75

(ddt, J = 12.1, 3.8, 2.0 Hz, 2H), 1.67 (dq, J = 13.3, 3.3 Hz, 1H), 1.58 – 1.46 (m, 1H), 1.42 – 1.24 (m, 3H), 1.15 (td, J = 13.9, 3.3 Hz, 1H), 1.08 (d, J = 6.2 Hz, 6H). (Exchangeable 1 x NH not observed).

^{13}C NMR ($CDCl_3$, 100 MHz): δ 146.2, 132.3 (2C), 127.9 (2C), 118.8, 111.5, 70.4, 66.5, 60.5, 55.6, 53.4, 44.9, 35.2, 27.8, 27.6, 27.1, 23.0, 23.0.

HRMS (ESI) m/z for $C_{19}H_{27}N_3O$ $[M+Na]^+$ calcd 336.2046, found 336.2049.

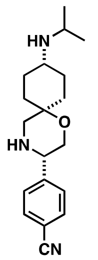

4-((3*S,6*r**,9*S**)-9-(isopropylamino)-1-oxa-4-azaspiro[5.5]undecan-3-yl)benzonitrile**
^{1}H NMR ($CDCl_3$, 400 MHz): δ 7.66 – 7.58 (m, 2H), 7.57 – 7.50 (m, 2H), 3.93 (dd, J = 9.3, 4.6 Hz, 1H), 3.67 – 3.54 (m, 2H), 3.27 (d, J = 11.7 Hz, 1H), 3.03 – 2.89 (m, 1H), 2.78 – 2.67 (m, 2H), 2.64 – 2.54 (m, 1H), 1.94 – 1.84 (m, 2H), 1.74 – 1.66 (m, 2H), 1.59 – 1.45 (m, 2H), 1.31 – 1.15 (m, 2H), 1.08 (d, J = 6.3 Hz, 6H). (Exchangable 1 x NH not observed).

^{13}C NMR ($CDCl_3$, 100 MHz): δ 146.3, 132.3 (2C), 128.0 (2C), 118.8, 111.5, 72.1, 67.1, 60.6, 52.6, 51.8, 45.4, 35.1, 29.4, 29.0, 27.0, 23.4, 23.3.

HRMS (ESI) m/z for $C_{19}H_{27}N_3O$ $[M+H]^+$ calcd 314.2227, found 314.2225.

Assignment of relative stereochemistry (6a – 6c)
The relative stereochemistry of 6g was assigned by x-ray crystallography. After preparation and resolution by preparative HPLC, both diastereomers were crystalized by dissolving in ethyl acetate : toluene (1:1) and adding 2 equiv. of 2,2,2-trifluoroacetic acid. "A" and "B" were assigned by elution order from reverse phase HPLC. A suitable crystal was selected and measured on a XtaLAB Synergy, Dualflex, Pilatus 300K diffractometer. The crystal was kept at 100.0(1) K during data collection. Using Olex2[14], the structure was solved with the SHELXT[15] structure solution program using Intrinsic Phasing and refined with the SHELXL [16] refinement package using Least Squares minimisation. The relative stereochemistry of 6a - 6c were then assigned by analogy.

The supplementary crystallographic data is provided by the Cambridge Crystallographic Data Centre (CCDC). The data can be accessed using the following deposition numbers via https://www.ccdc.cam.ac.uk/structures/.

[14] Dolomanov, O.V., Bourhis, L.J., Gildea, R.J, Howard, J.A.K. & Puschmann, H., *J. Appl. Cryst.*, 2009, **42**, 339.

[15] Sheldrick, G.M., *Acta Cryst.*, 2015, A71, 3-8.

[16] Sheldrick, G.M., *Acta Cryst.*, 2015, C71, 3-8.

6g DiaA (CCDC deposition number 2064033)

Crystal Data for $C_{21}H_{28}F_3N_3O_3$ (M =427.46 g/mol): monoclinic, space group C2/c (no. 15), a = 52.9651(13) Å, b = 8.6539(2) Å, c = 19.4197(4) Å, β = 91.443(2)°, V = 8898.3(4) Å^3, Z = 16, T = 100.0(1) K, μ(Cu Kα) = 0.869 mm^{-1}, $Dcalc$ = 1.276 g/cm^3, 34793 reflections measured (3.338° ≤ 2Θ ≤ 116.918°), 6226 unique (R_{int} = 0.0506, R_{sigma} = 0.0424) which were used in all calculations. The final R_1 was 0.0618 (I > 2σ(I)) and wR_2 was 0.1935 (all data).

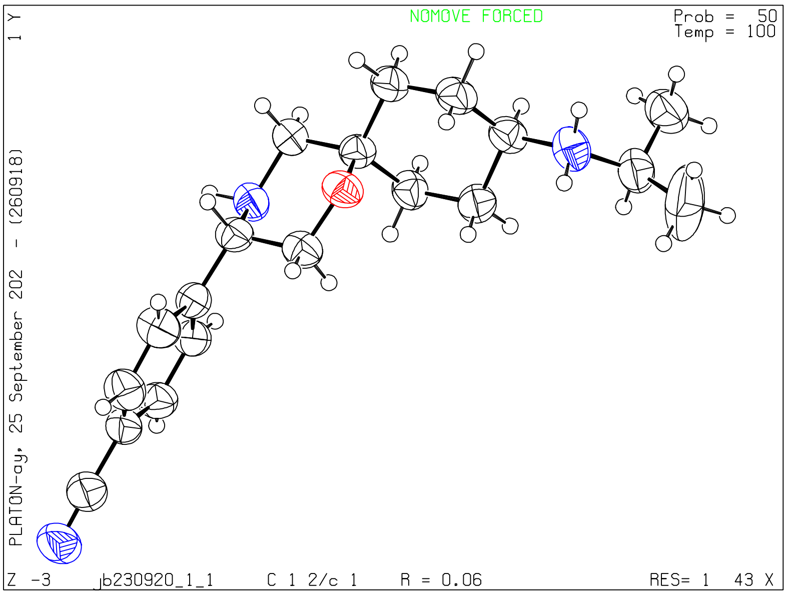

6g DiaB (CCDC deposition number 2064034)

Crystal Data for $C_{21}H_{28}F_3N_3O_3$ (M =427.46 g/mol): triclinic, space group P-1 (no. 2), a = 8.7250(3) Å, b = 11.2299(6) Å, c = 12.2001(5) Å, α = 79.147(4)°, β = 80.009(3)°, γ = 73.949(4)°, V = 1118.85(9) Å^3, Z = 2, T = 250.0(1) K, μ(Cu Kα) = 0.864 mm^{-1}, $Dcalc$ = 1.269 g/cm^3, 17567 reflections measured (7.44° ≤ 2Θ ≤ 161.254°), 4740 unique (R_{int} = 0.0267, R_{sigma} = 0.0286) which were used in all calculations. The final R_1 was 0.0480 (I > 2σ(I)) and wR_2 was 0.1644 (all data).

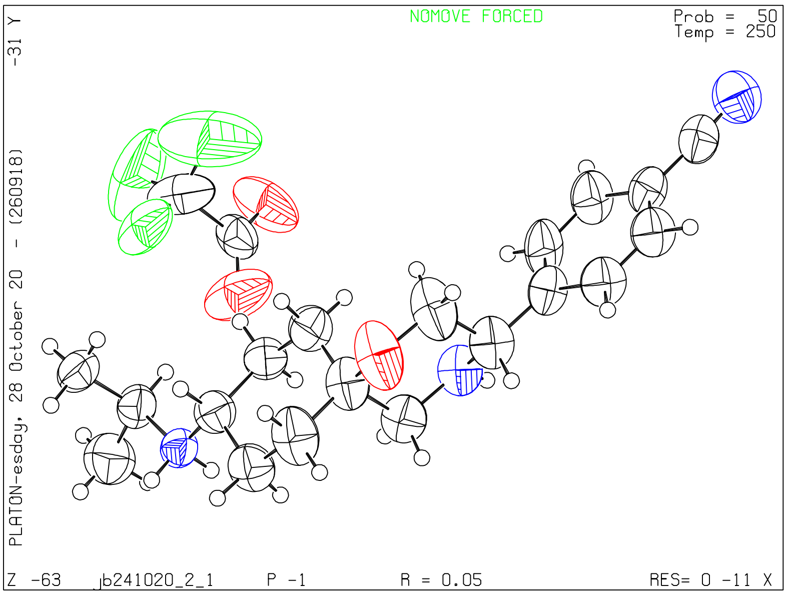

7. NMR Spetra of product compounds

^{1}H NMR of **OA3**

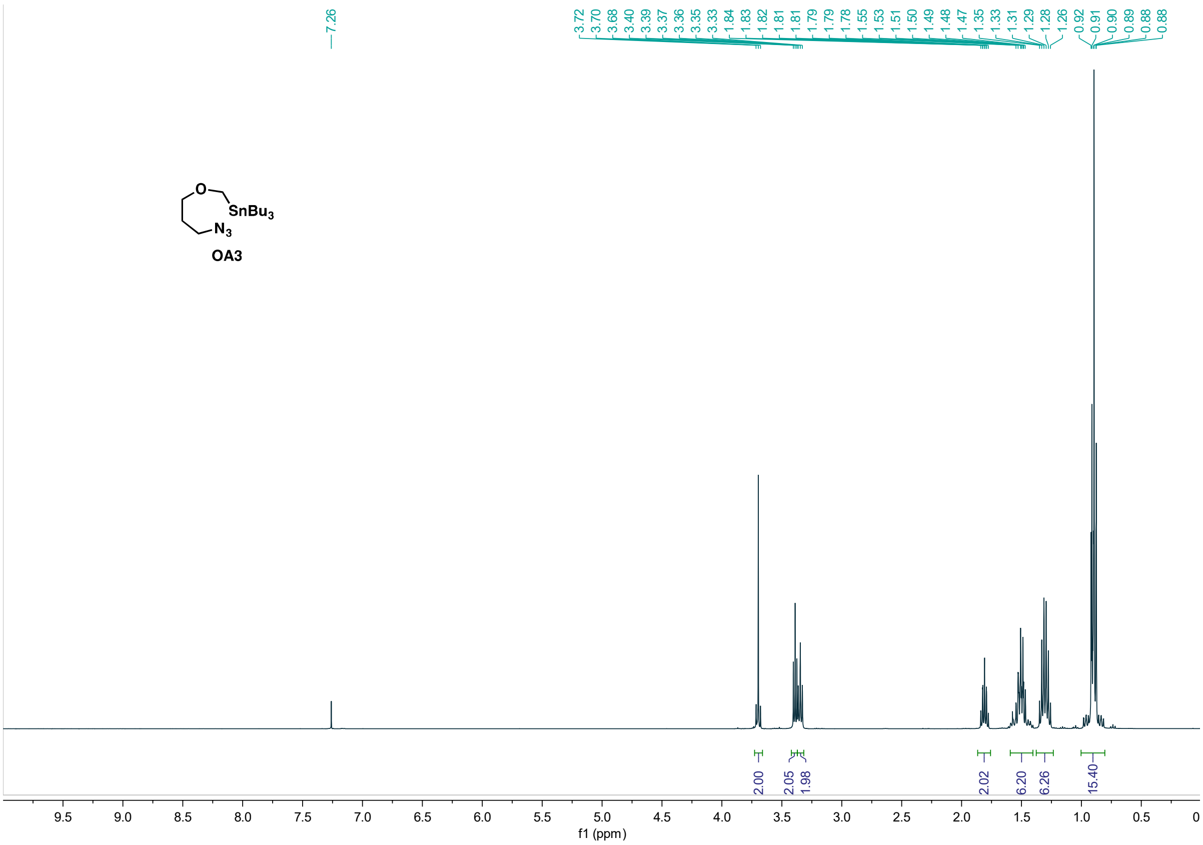

^{13}C NMR of **OA3**

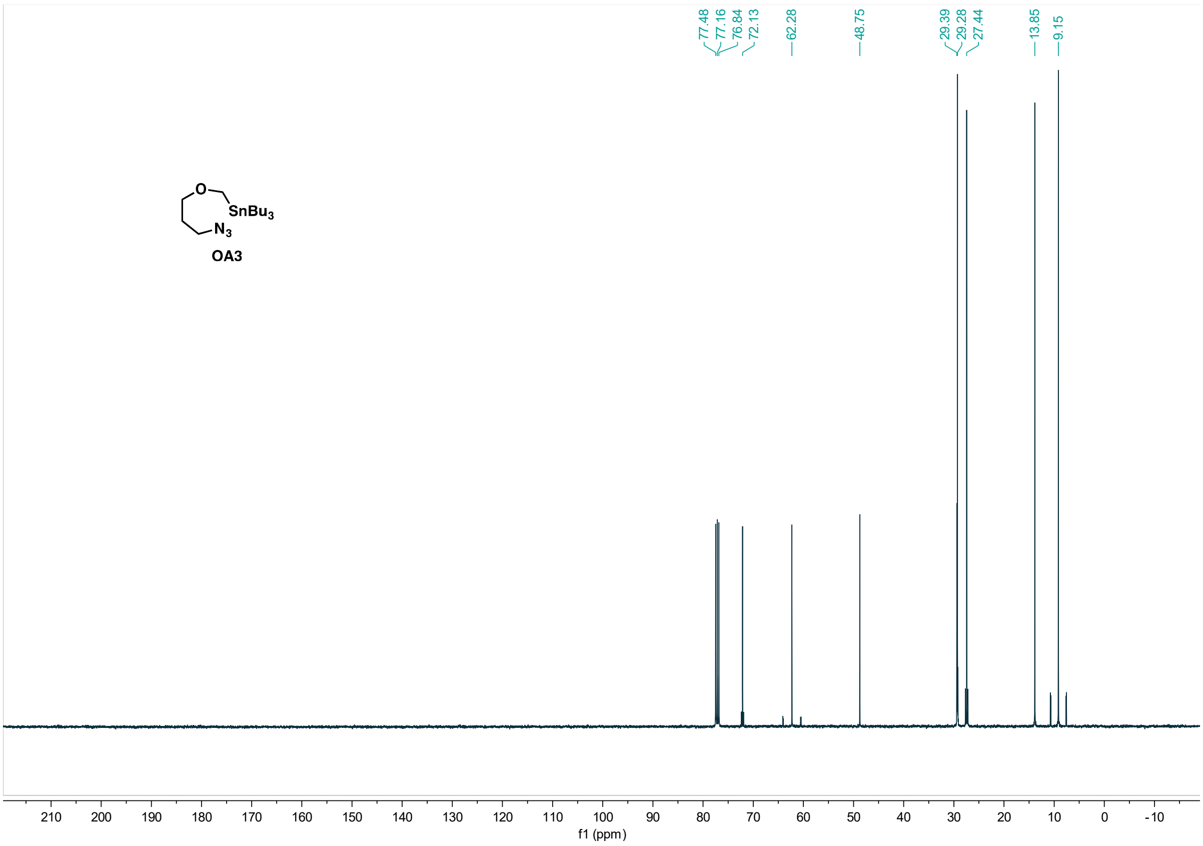

^{1}H NMR of **DA4**

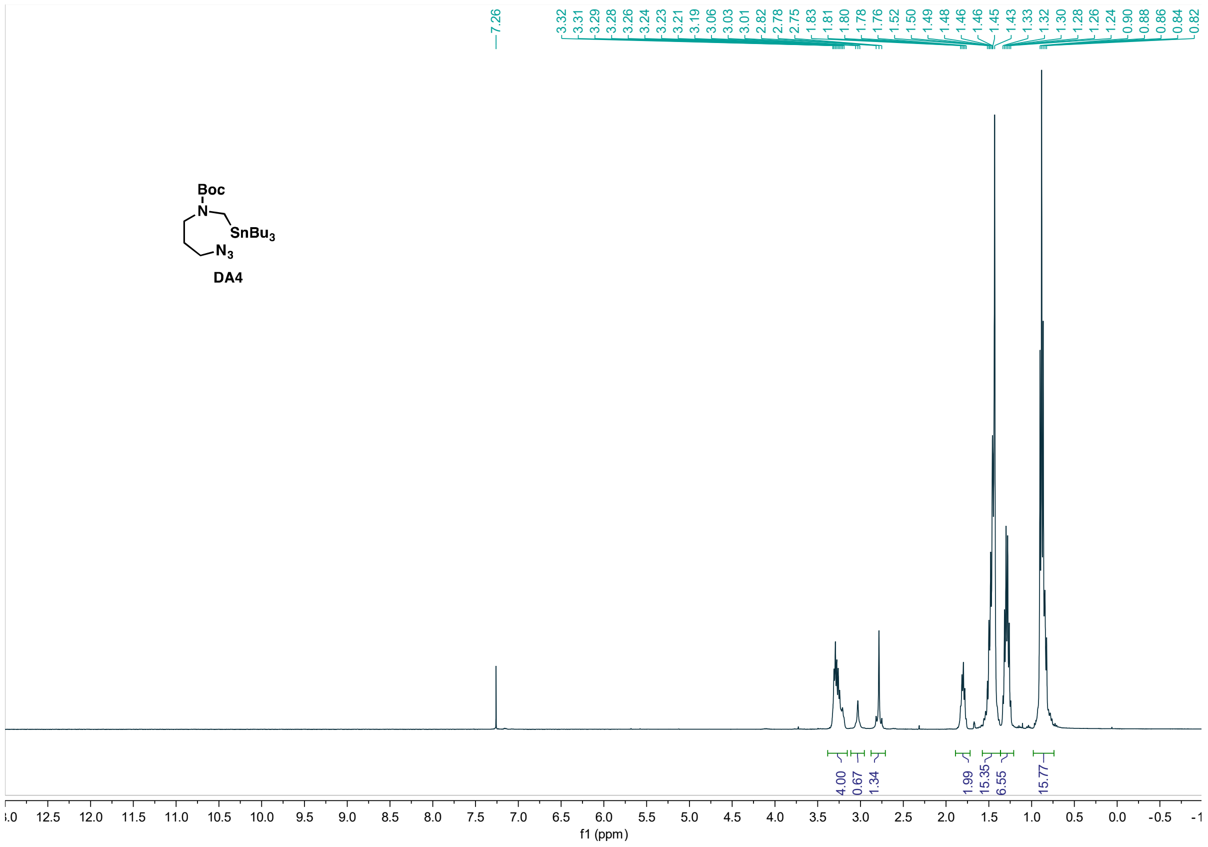

^{13}C NMR of **DA4**

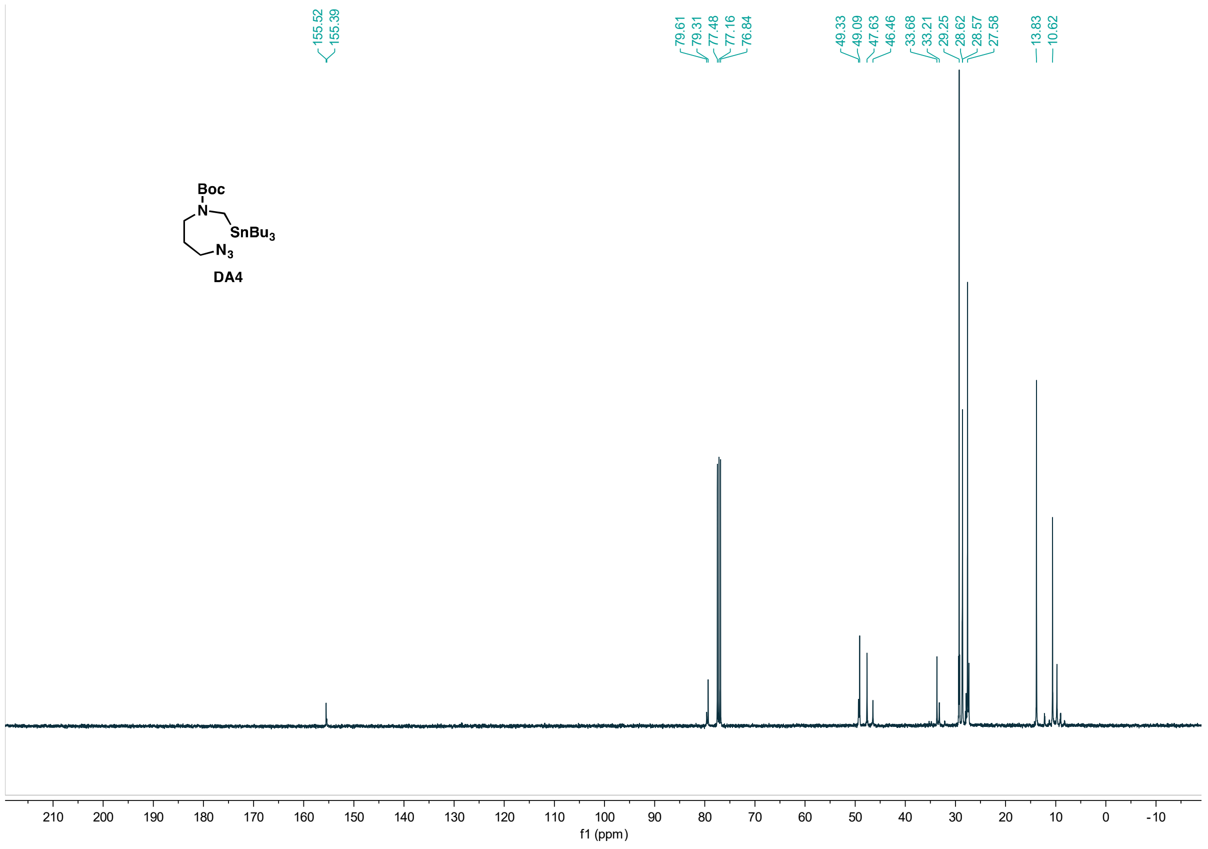

^{1}H NMR of the product **1a** obtained from Synple Chem synthesizer without purification:

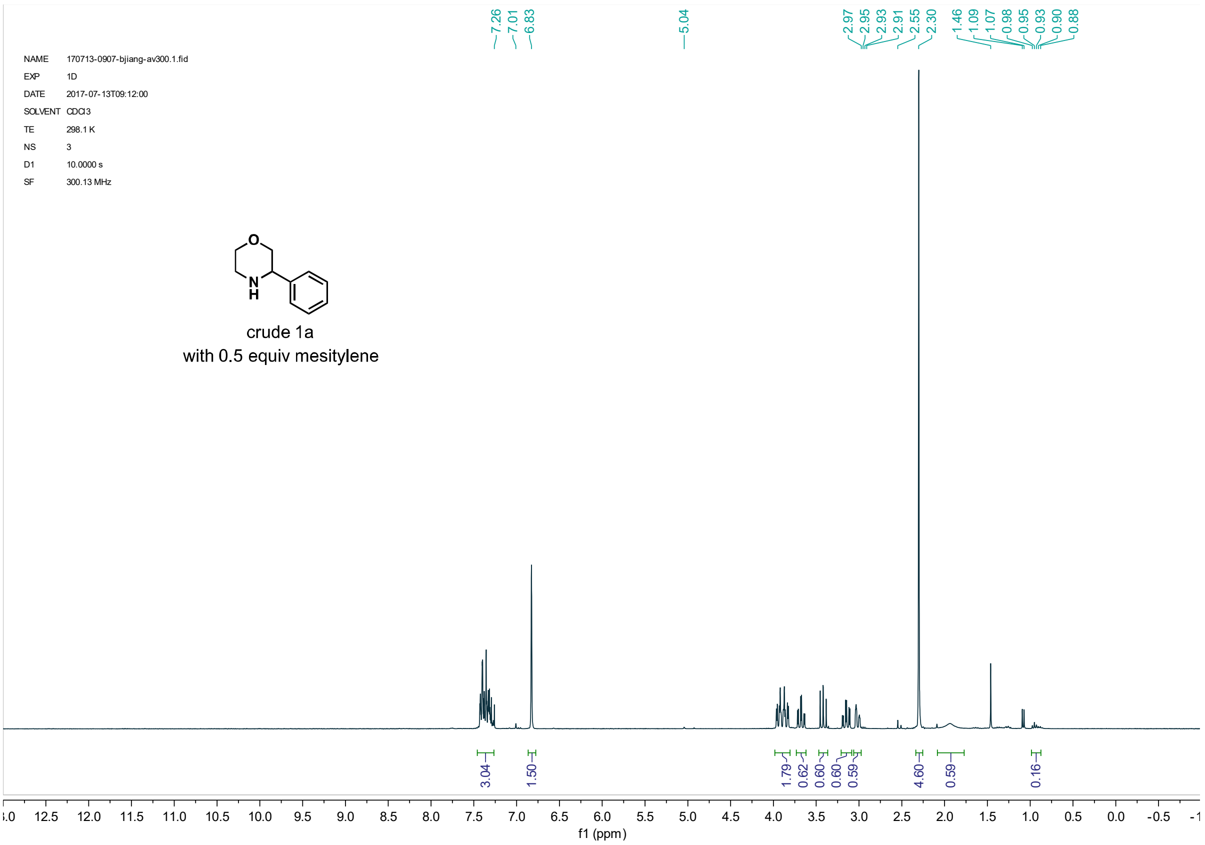

Internal standard: 0.5 equiv mesitylene (*δ* 6.83, 2.30). Volatile impurity: DIPA (*δ* 2.97–2.91, 1.09–1.07). Other impurity: BHT (*δ* 7.01, 5.04, 2.55, 1.46), "Sn" (*δ* 0.98–0.88).

^{1}H NMR of the purified product **1a**:

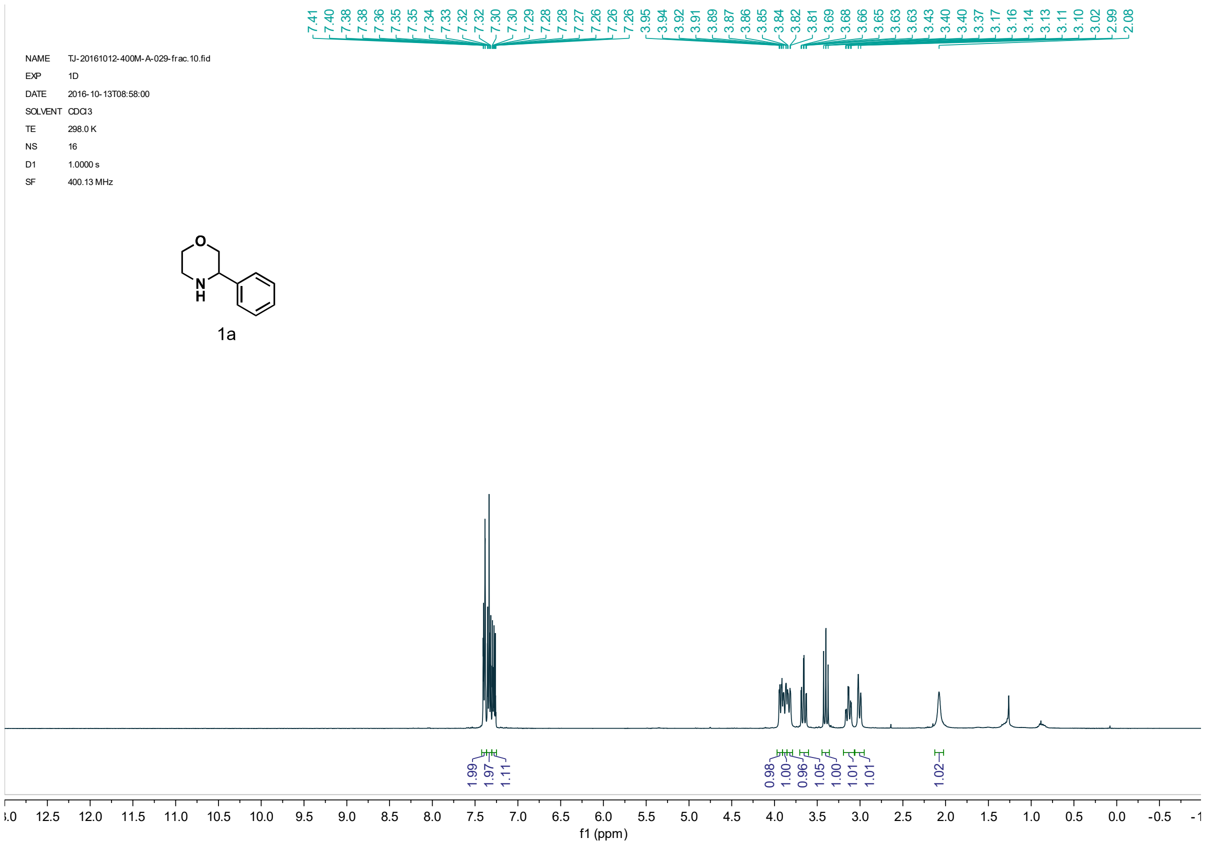

^{1}H NMR of the product **1b** obtained from Synple Chem synthesizer without purification:

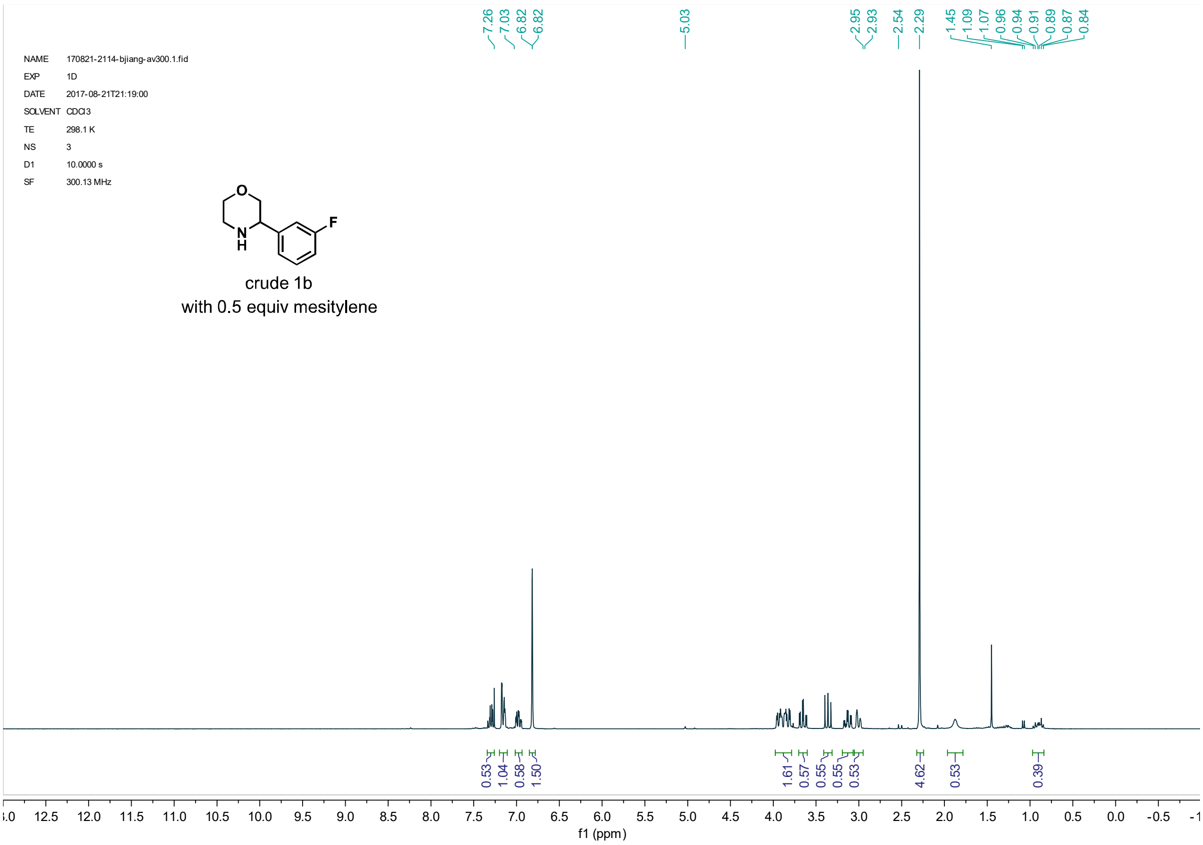

Internal standard: 0.5 equiv mesitylene (δ 6.82, 2.29). Volatile impurity: DIPA (δ 2.95–2.93, 1.09–1.07). Other impurity: BHT (δ 7.03, 5.03, 2.54, 1.45), "Sn" (δ 0.96–0.84).

^{1}H NMR of the purified product **1b**:

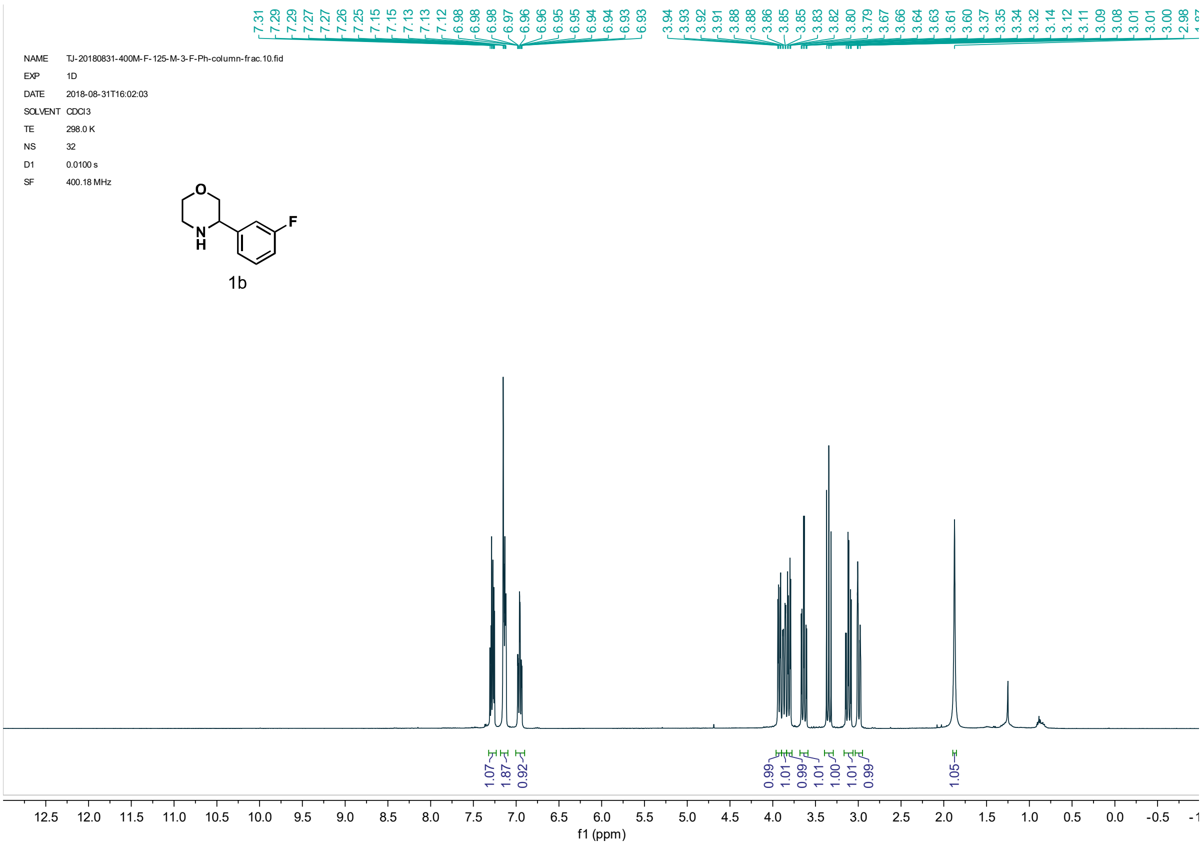

^{13}C NMR of the purified product **1b**:

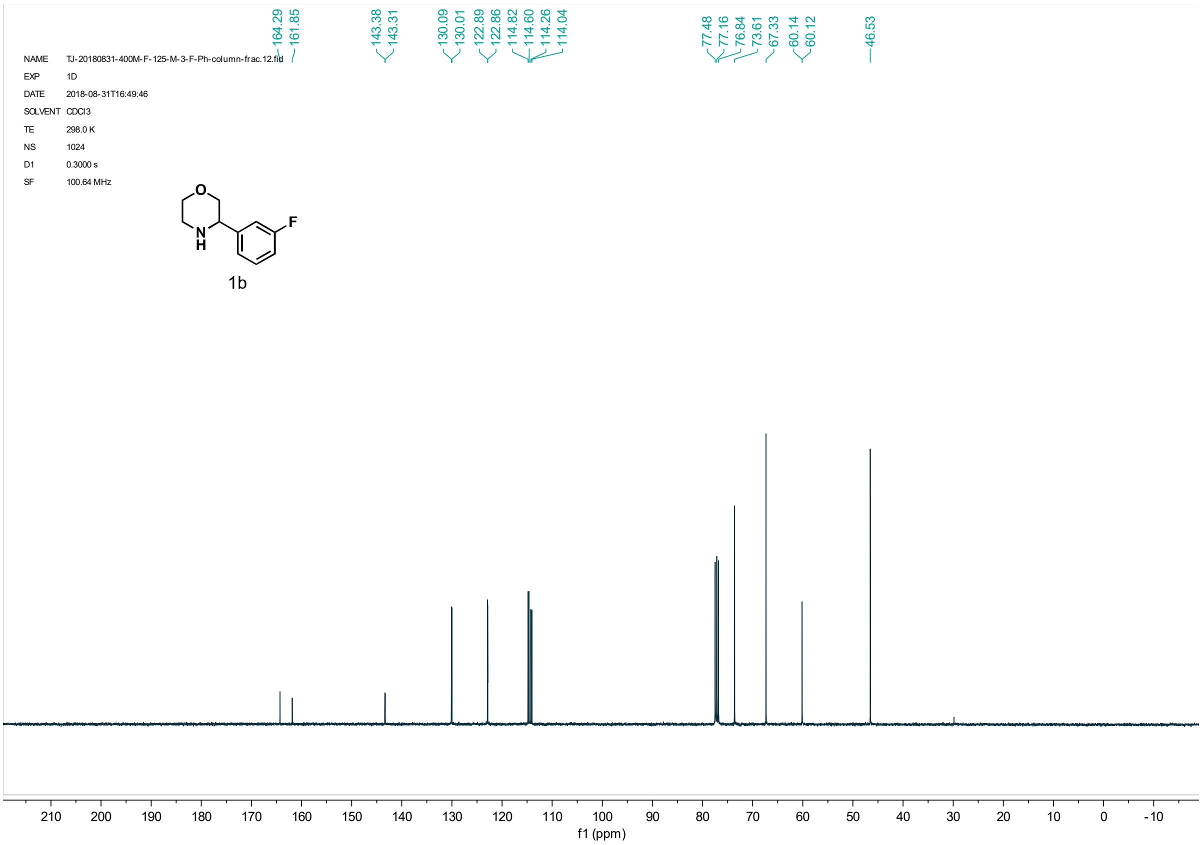

^{1}H NMR of the product **1c** obtained from Synple Chem synthesizer without purification:

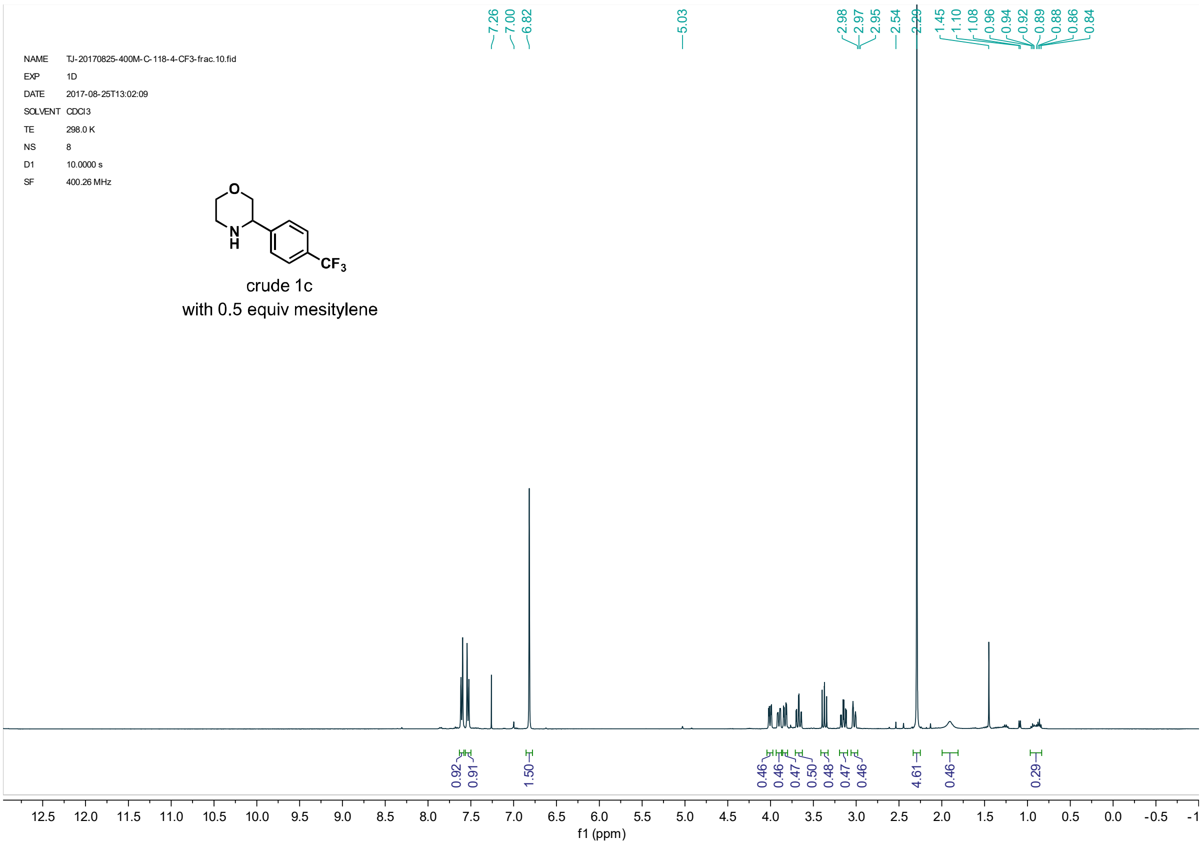

Internal standard: 0.5 equiv mesitylene (δ 6.82, 2.29). Volatile impurity: DIPA (δ 2.98–2.95, 1.10–1.08). Other impurity: BHT (δ 7.00, 5.03, 2.54, 1.45), "Sn" (δ 0.96–0.84).

^{1}H NMR of the purified product **1c**:

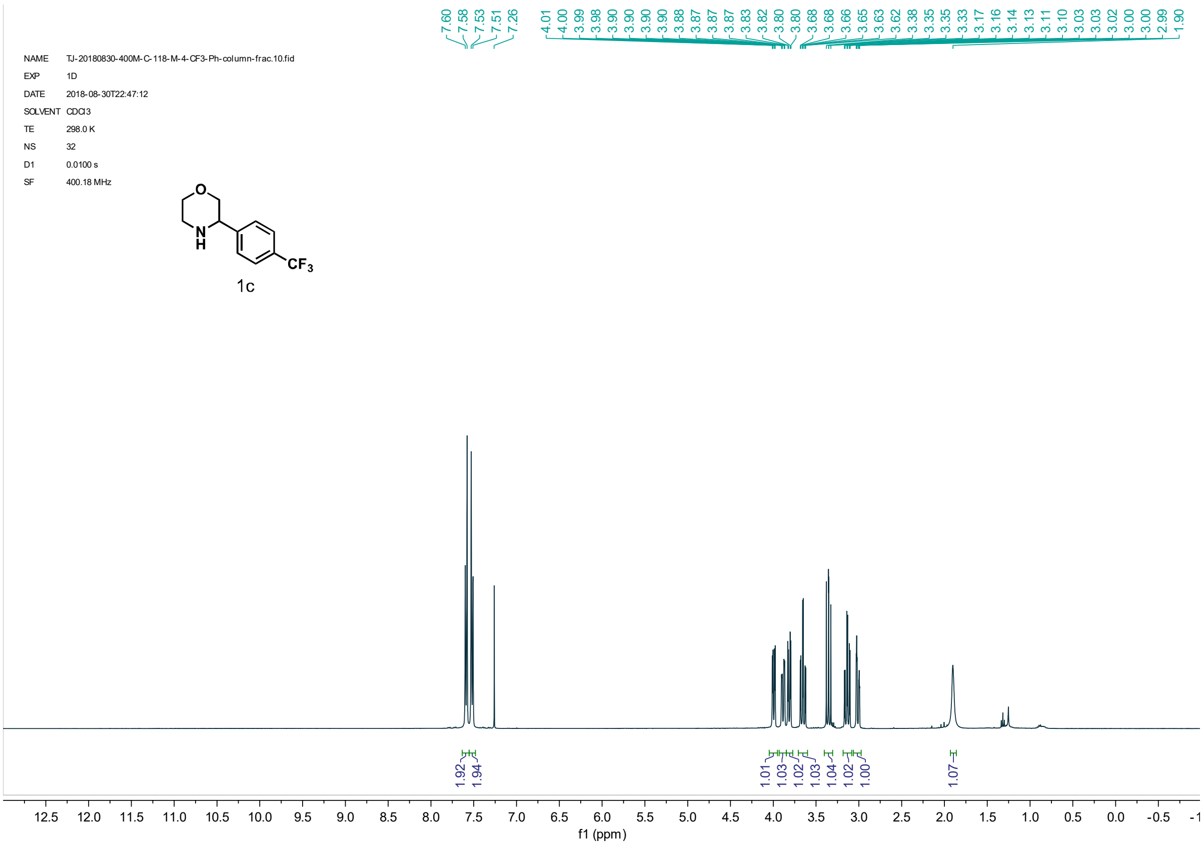

^{13}C NMR of the purified product **1c**:

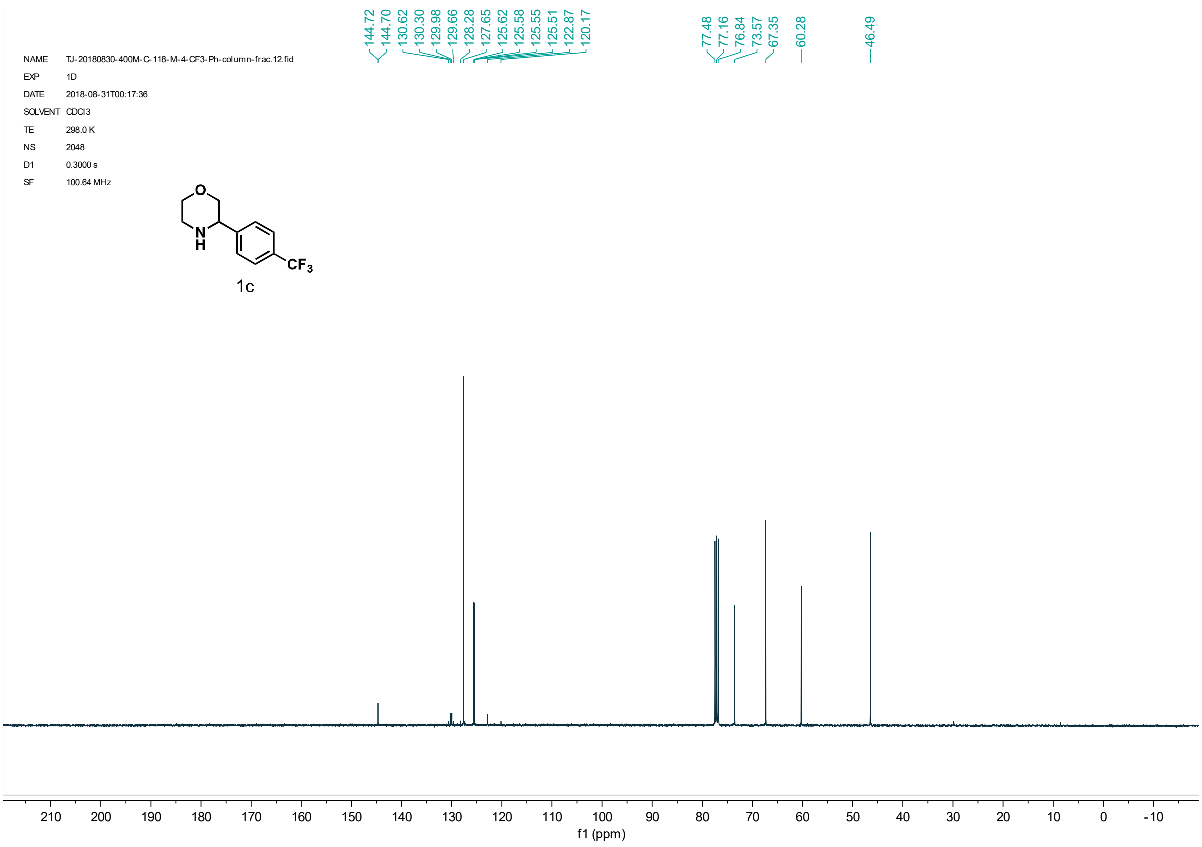

^{1}H NMR of the product **1d** obtained from Synple Chem synthesizer without purification:

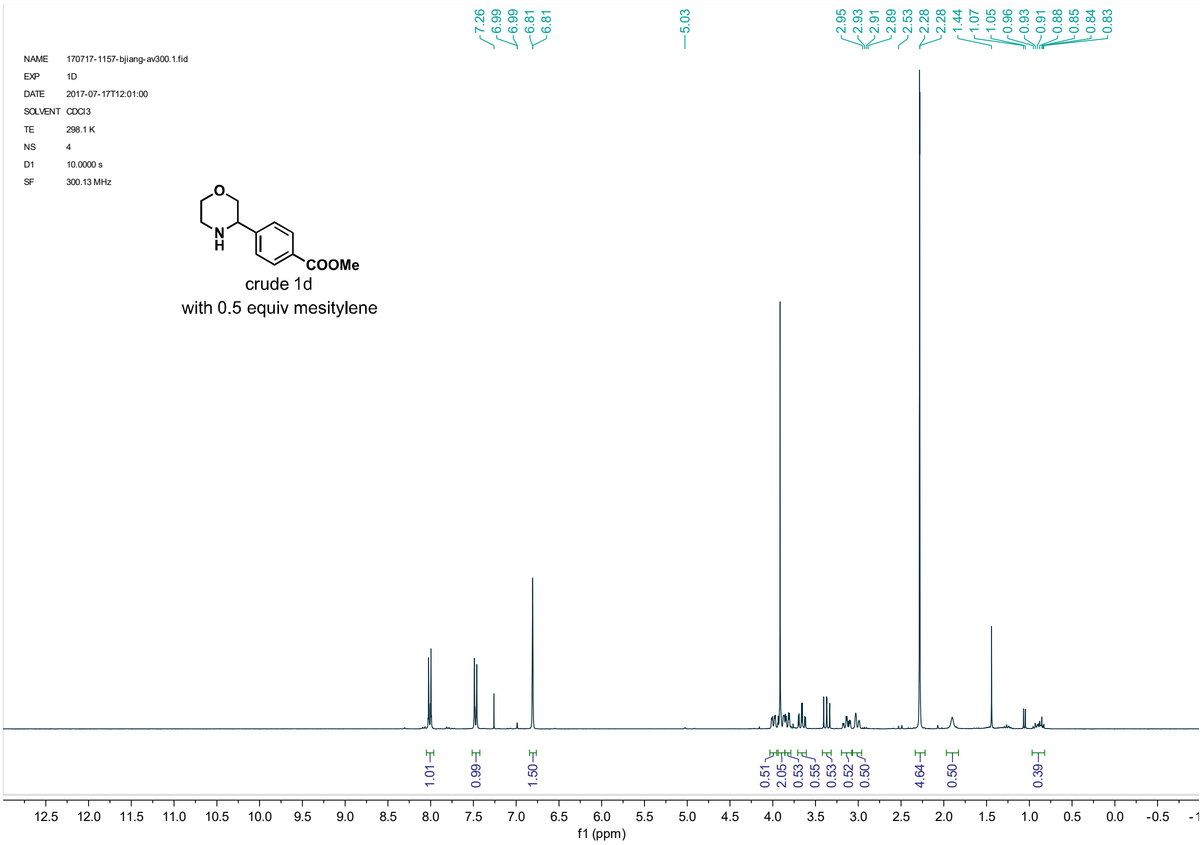

Internal standard: 0.5 equiv mesitylene (δ 6.81, 2.28). Volatile impurity: DIPA (δ 2.95–2.89, 1.07–1.05). Other impurity: BHT (δ 6.99, 5.03, 2.53, 1.44), “Sn” (δ 0.96–0.83).

^{1}H NMR of the purified product **1d**:

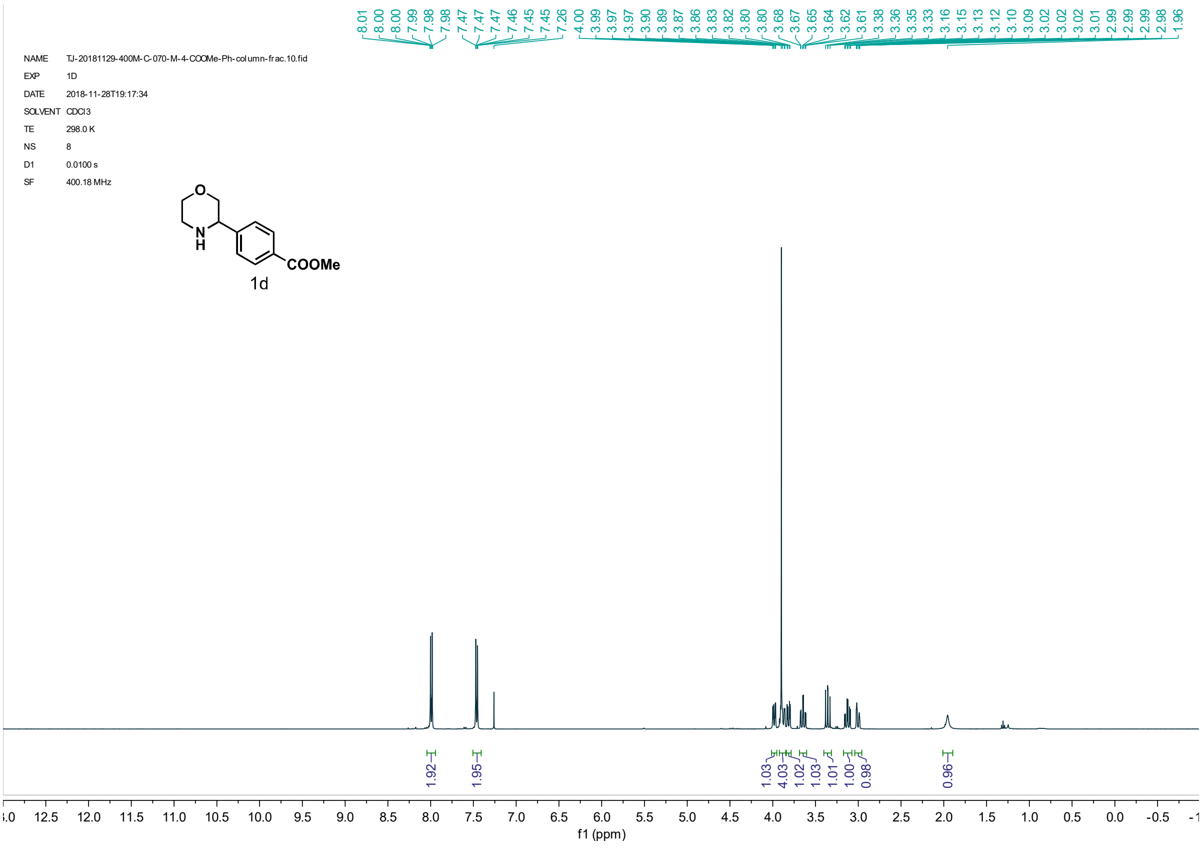

^{1}H NMR of the product **1e** obtained from Synple Chem synthesizer without purification:

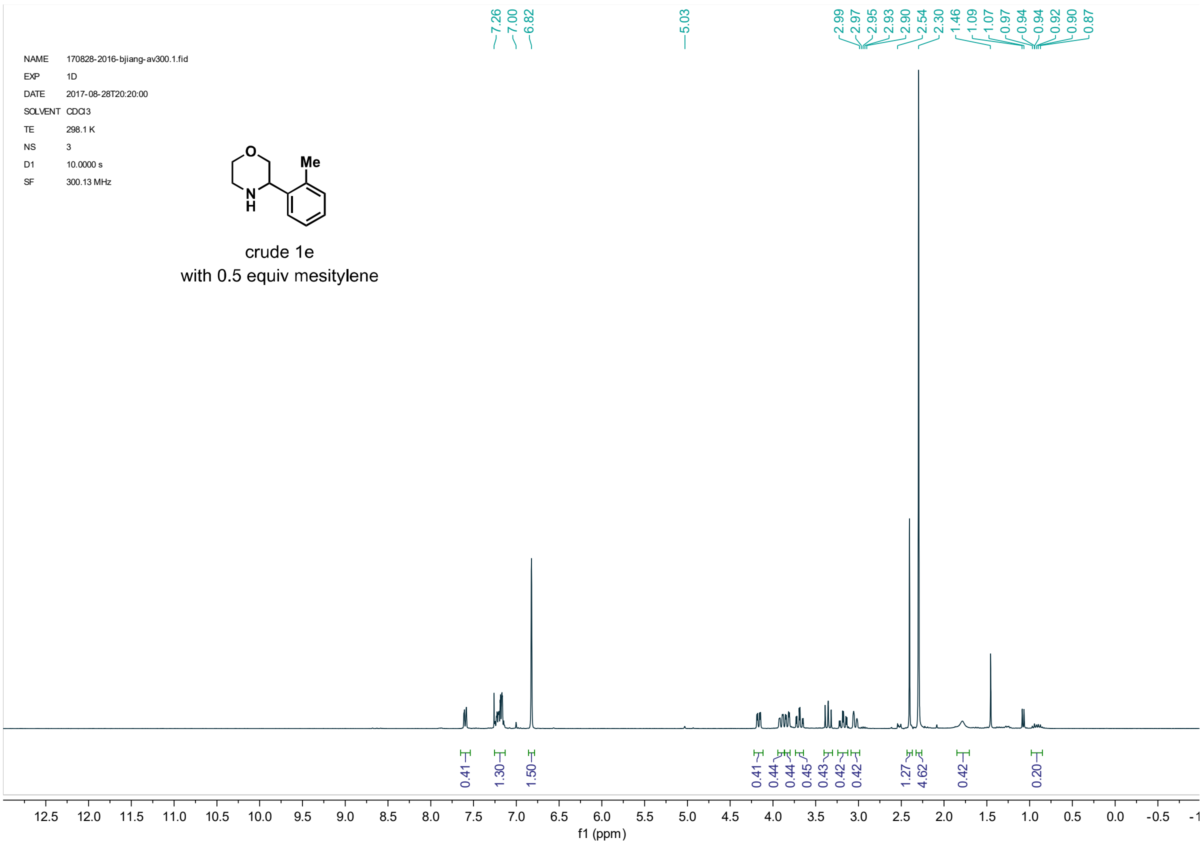

Internal standard: 0.5 equiv mesitylene (δ 6.82, 2.30). Volatile impurity: DIPA (δ 2.99–2.90, 1.09–1.07). Other impurity: BHT (δ 7.00, 5.03, 2.54, 1.46), "Sn" (δ 0.97–0.87).

^{1}H NMR of the purified product **1e**:

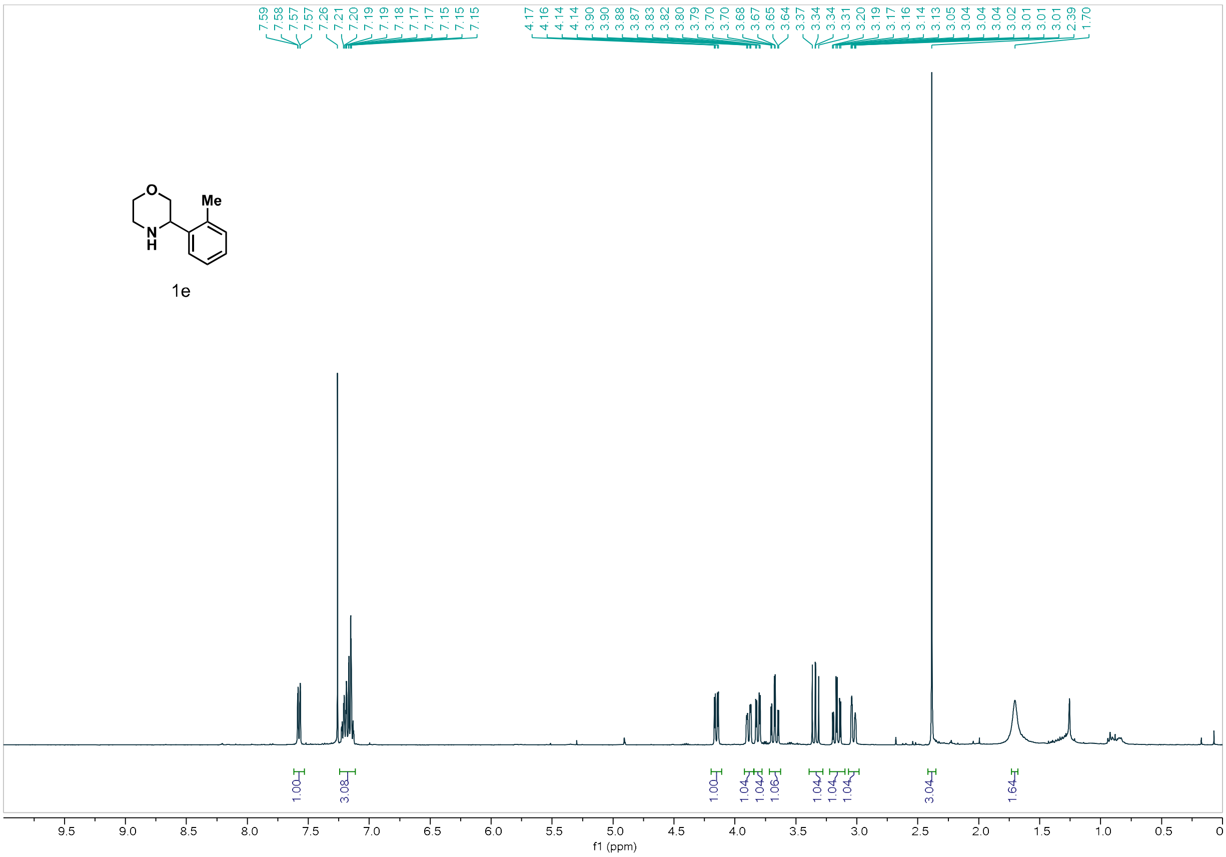

^{1}H NMR of the product **1f** obtained from Synple Chem synthesizer without purification:

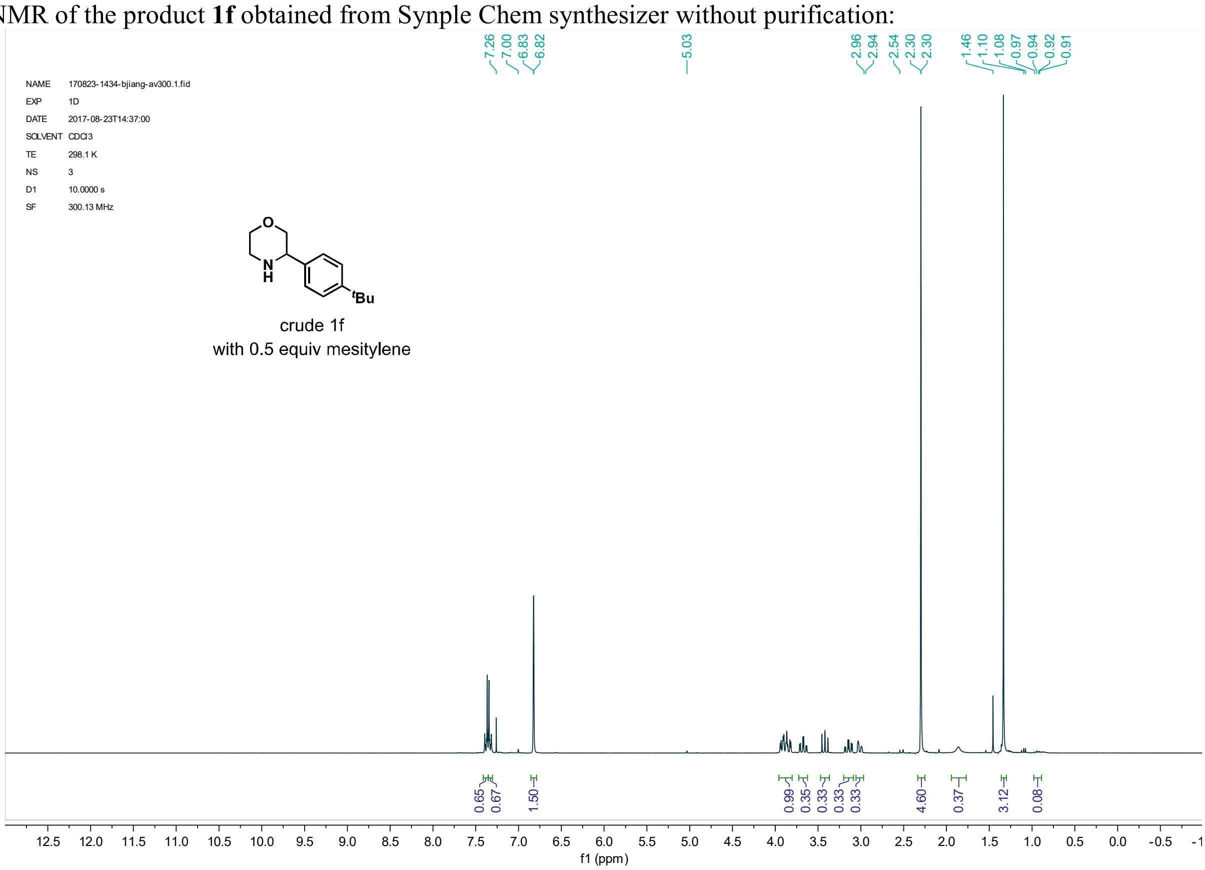

Internal standard: 0.5 equiv mesitylene (*δ* 6.83–6.82, 2.30). Volatile impurity: DIPA (*δ* 2.96–2.94, 1.10–1.08). Other impurity: BHT (*δ* 7.00, 5.03, 2.54, 1.46), "Sn" (*δ* 0.97–0.91).

^{1}H NMR of the purified product **1f**:

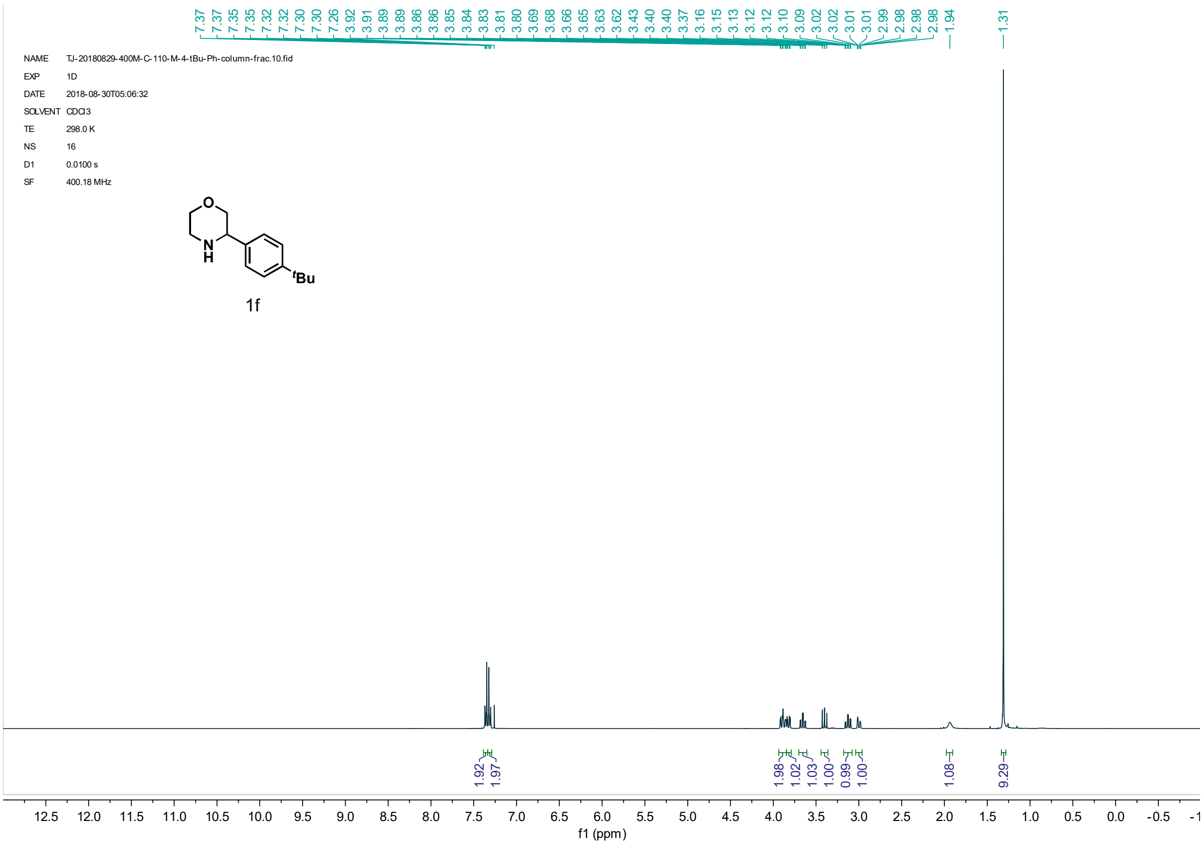

^{13}C NMR of the purified product **1f**:

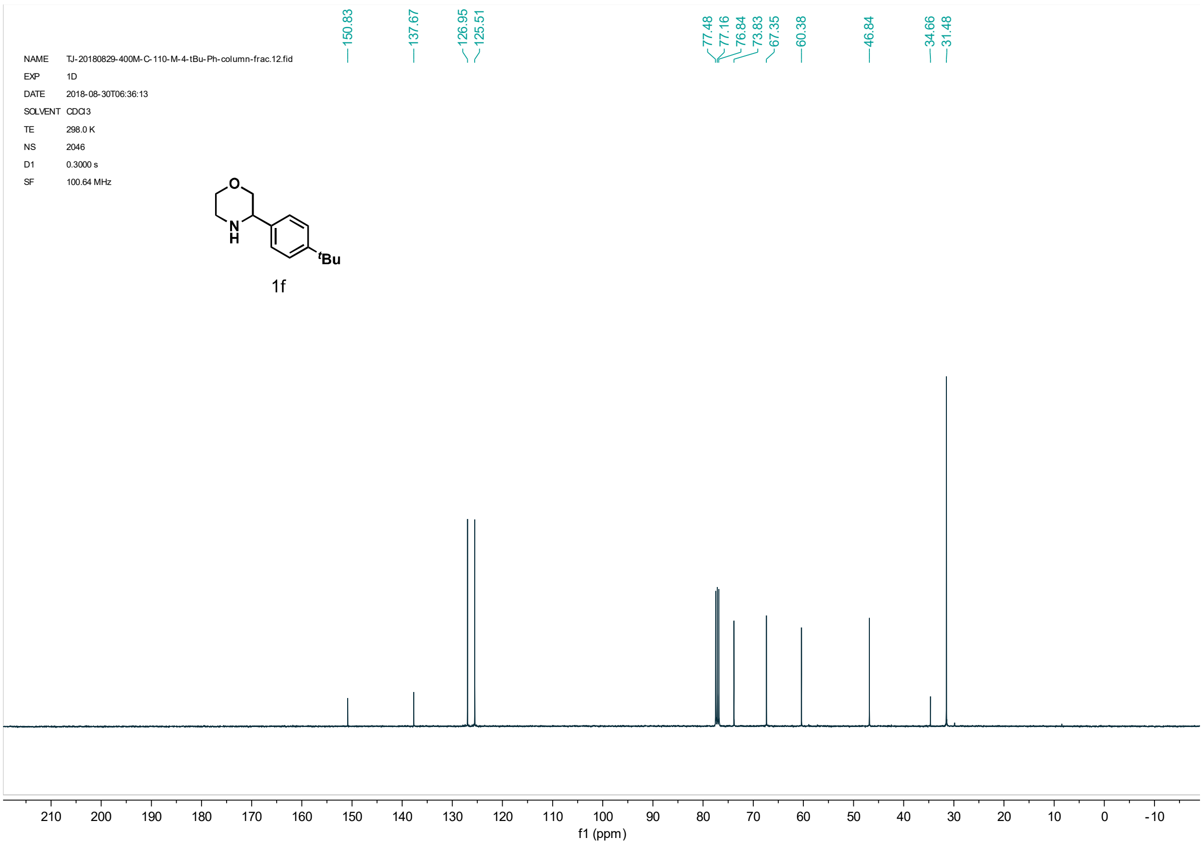

^{1}H NMR of the product **1g** obtained from Synple Chem synthesizer without purification:

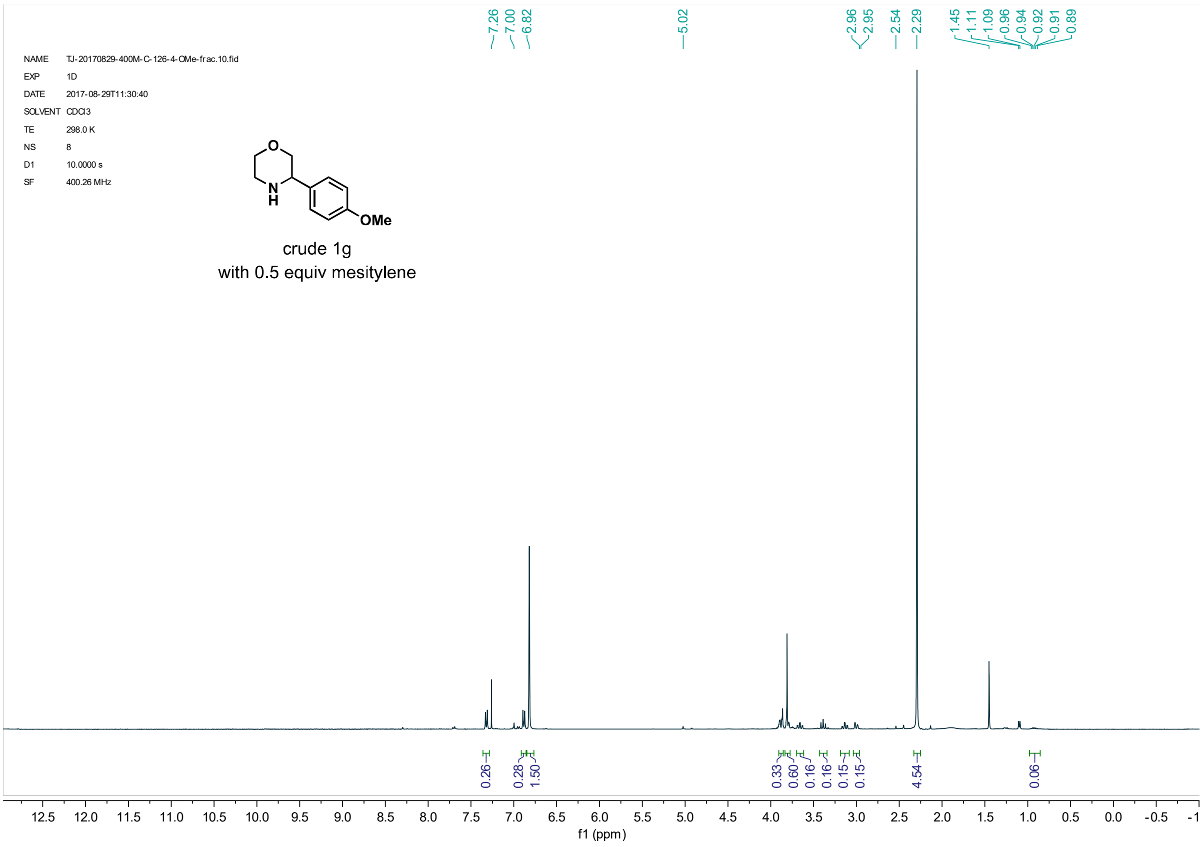

Internal standard: 0.5 equiv mesitylene (δ 6.82, 2.29). Volatile impurity: DIPA (δ 2.96–2.95, 1.11–1.09). Other impurity: BHT (δ 7.00, 5.02, 2.54, 1.45), “Sn” (δ 0.96–0.89).

^{1}H NMR of the purified product **1g**:

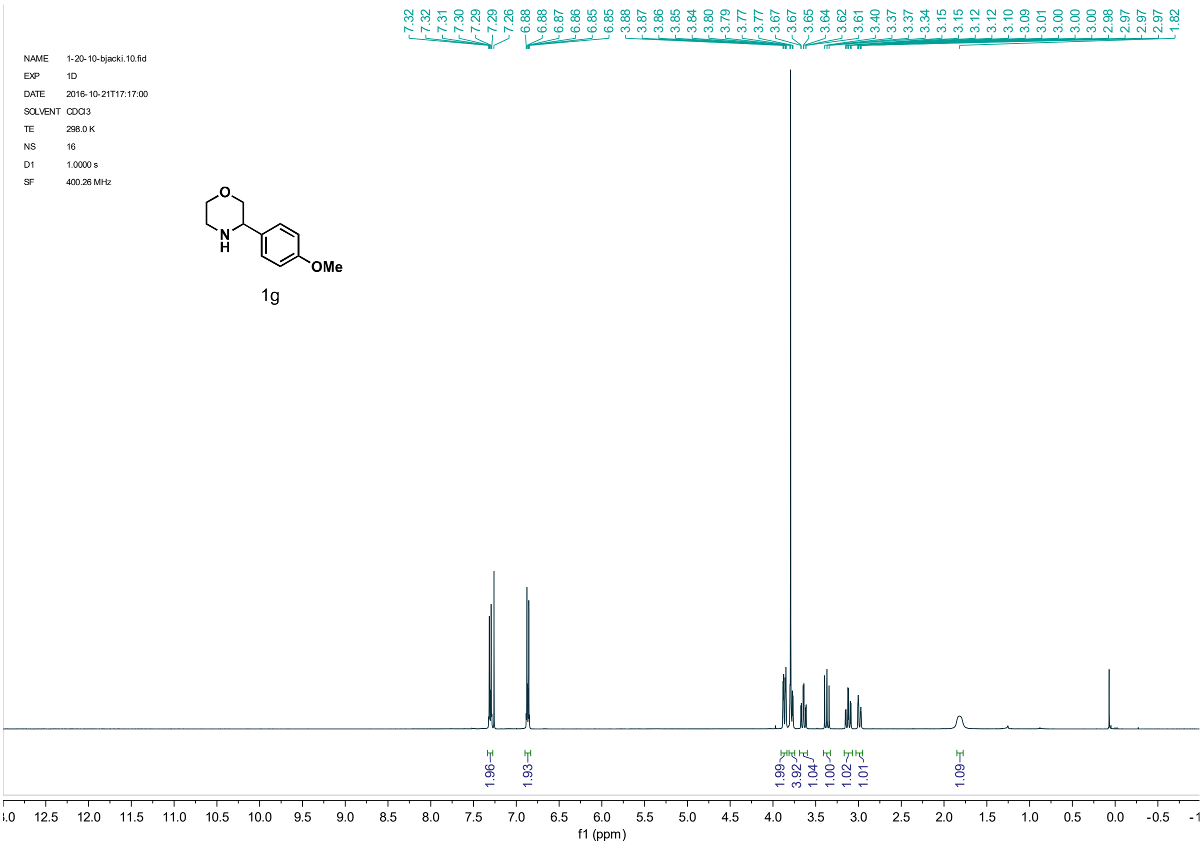

^{1}H NMR of the product **1h** obtained from Synple Chem synthesizer without purification:

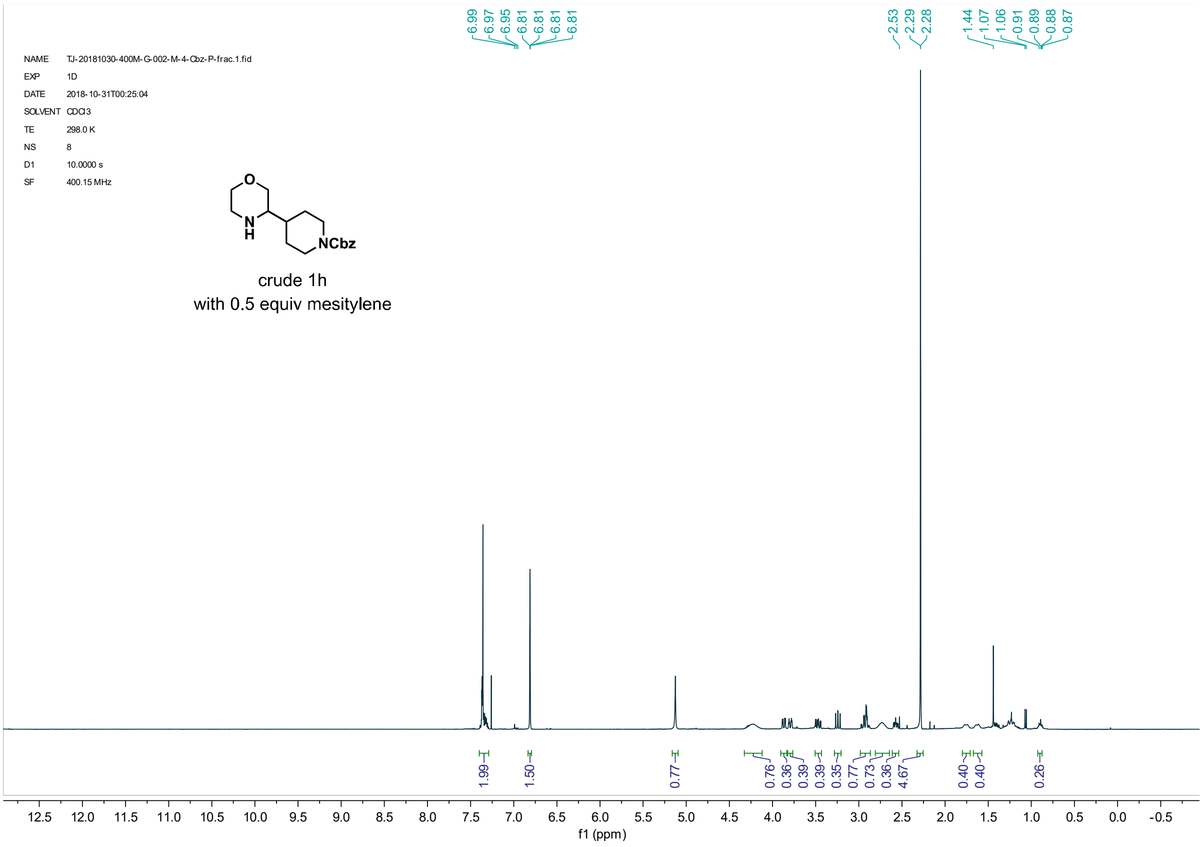

Internal standard: 0.5 equiv mesitylene (*δ* 6.81–6.80, 2.28). Volatile impurity: DIPA (*δ* 1.07–1.06). Other impurity: BHT (*δ* 6.99–6.95, 2.53, 1.44), “Sn” (*δ* 0.91–0.87).

^{1}H NMR of the purified product **1h**:

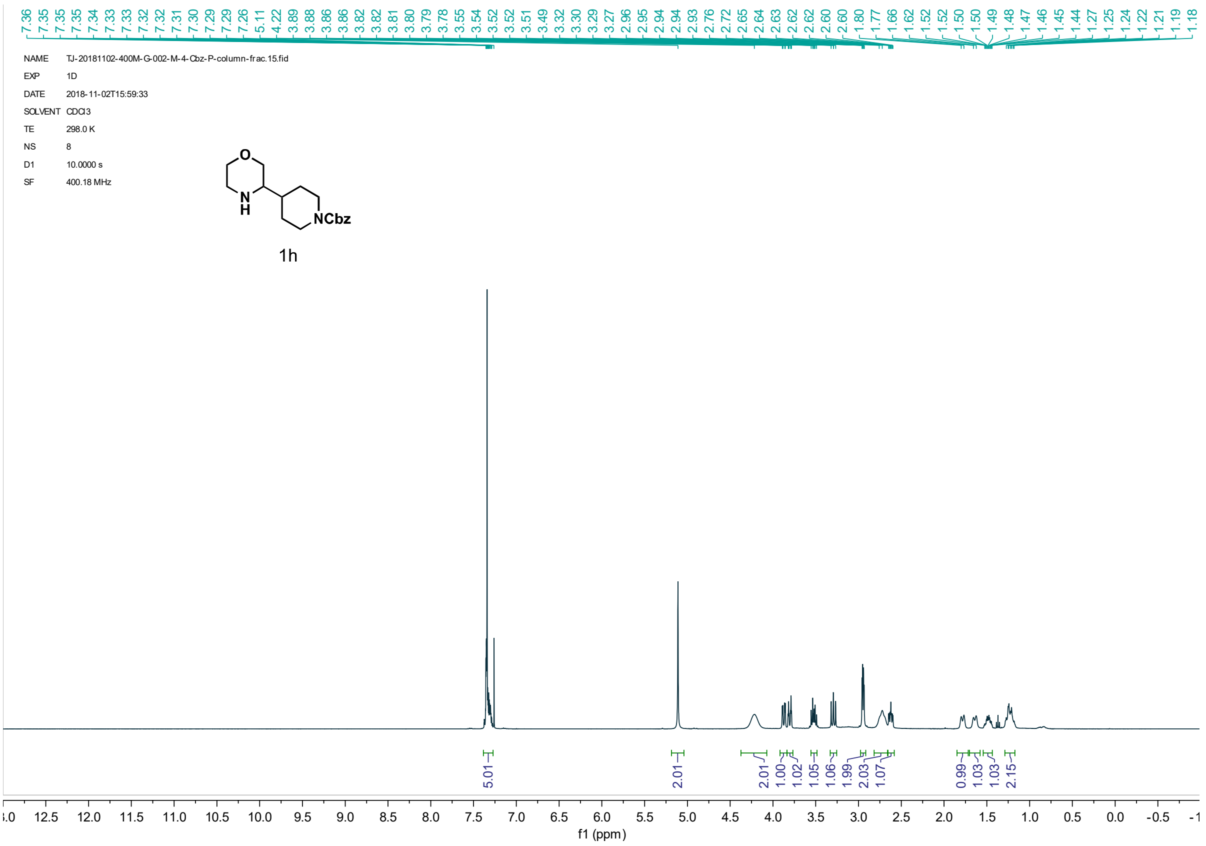

^{13}C NMR of the purified product **1h**:

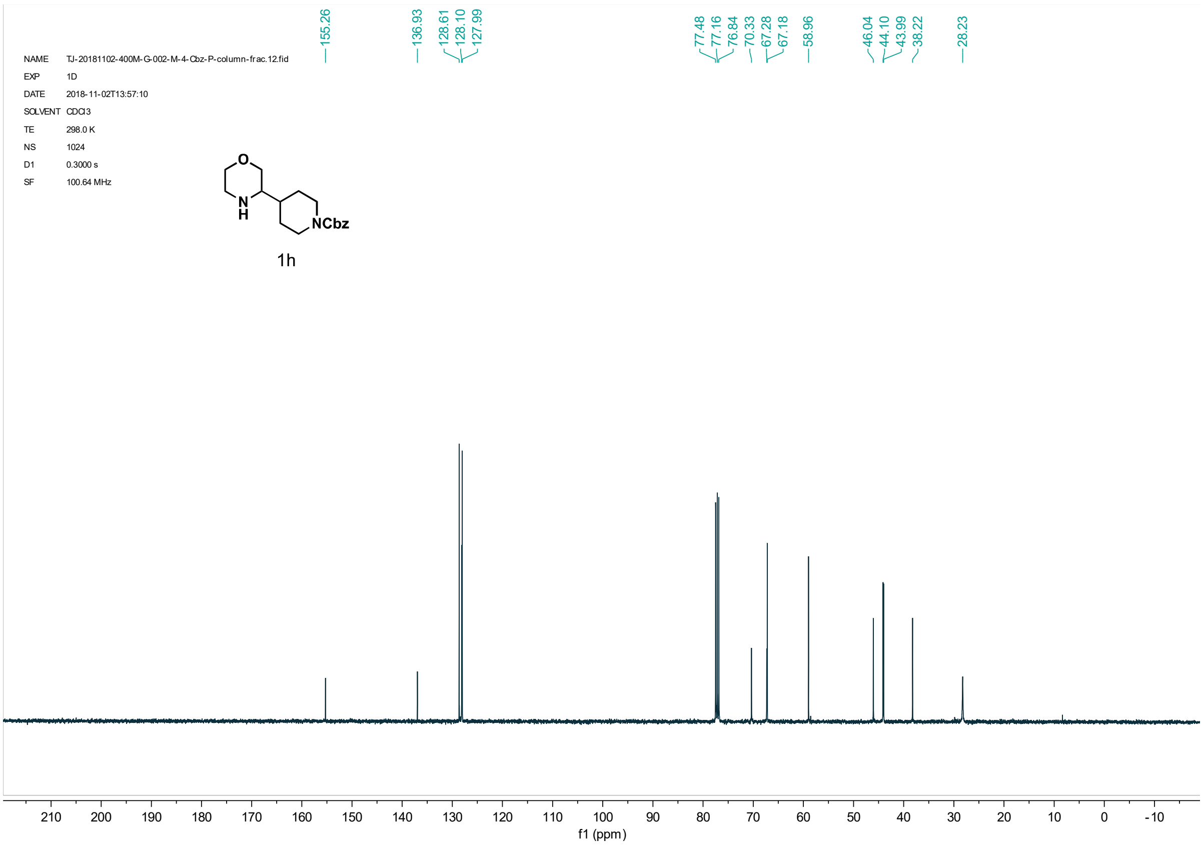

^{1}H NMR of the product **1i** obtained from Synple Chem synthesizer without purification:

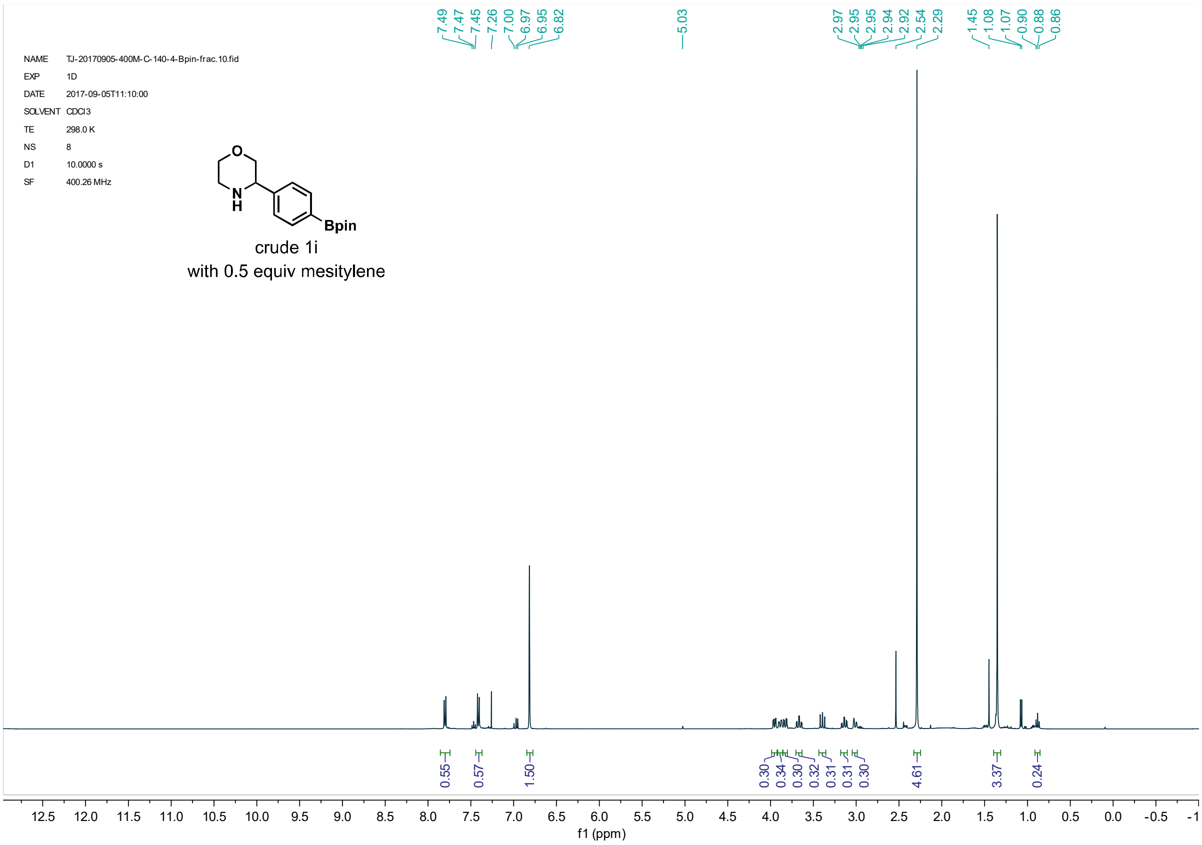

Internal standard: 0.5 equiv mesitylene (δ 6.82, 2.29). Volatile impurity: 2,6-lutidine (δ 7.49–7.45, 6.97–6.95, 2.54), DIPA (δ 2.97–2.92, 1.08–1.07). Other impurity: BHT (δ 7.00, 5.03, 2.54, 1.45), “Sn” (δ 0.90–0.86).

^{1}H NMR of the purified product **1i**:

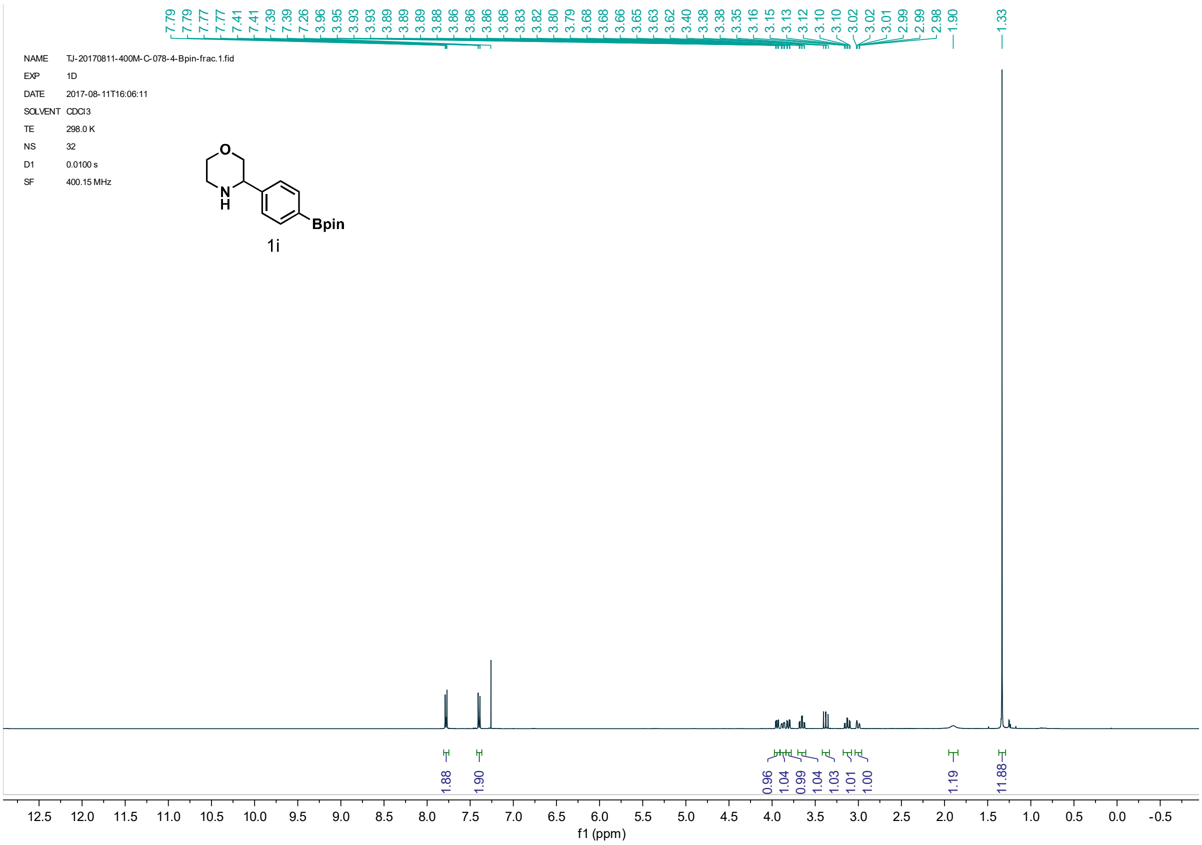

^{13}C NMR of the purified product **1i**:

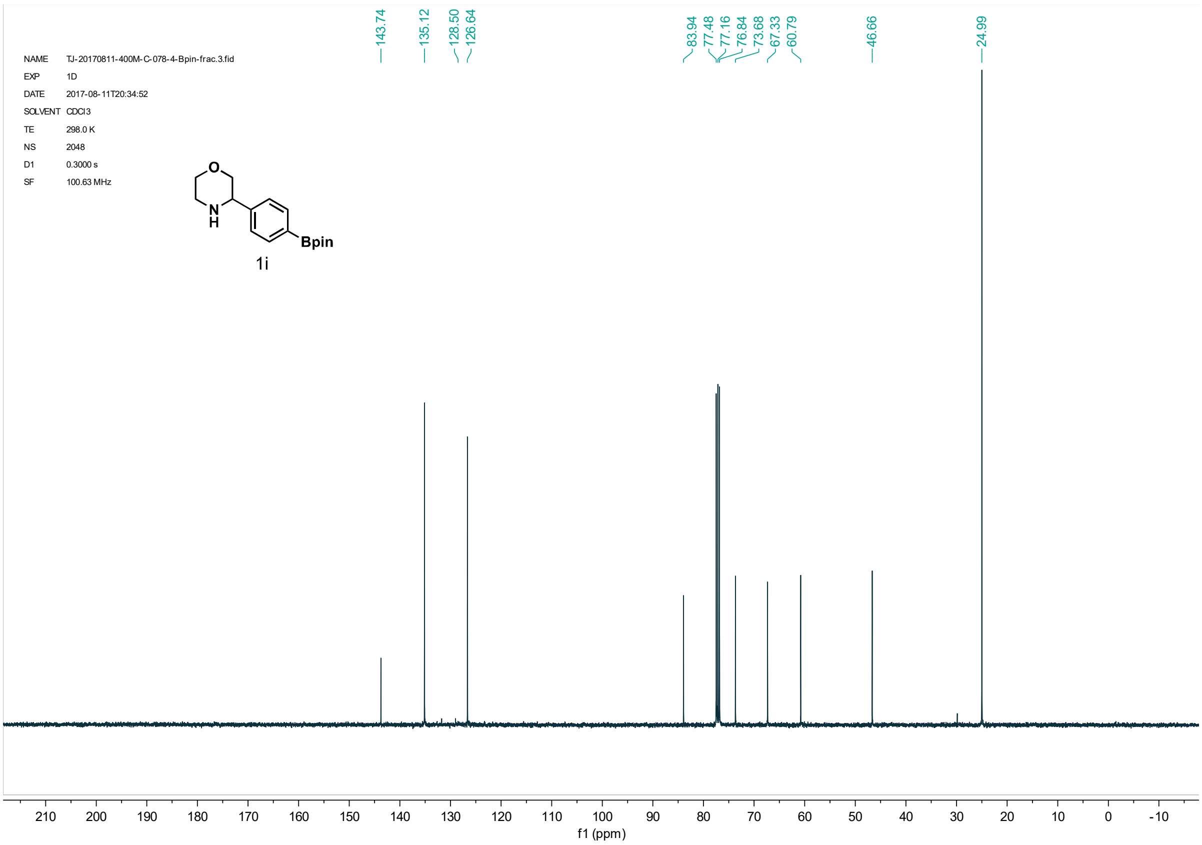

^{1}H NMR of the product **1j** obtained from Synple Chem synthesizer without purification:

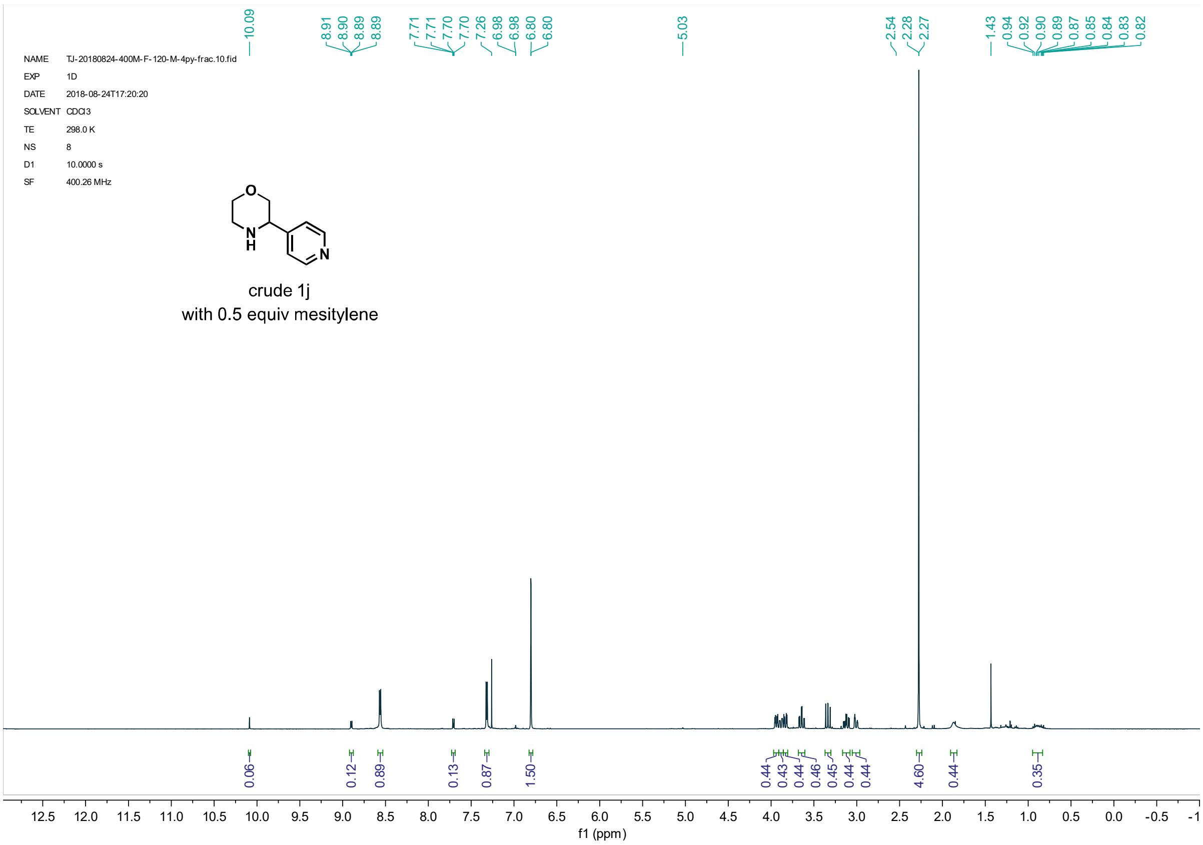

Internal standard: 0.5 equiv mesitylene (δ 6.80, 2.28–2.27). Volatile impurity: - .Other impurity: 4-formylpyridine (δ 10.1, 8.91–8.89, 7.71–7.70), BHT (δ 6.98, 5.03, 2.54, 1.43), "Sn" (δ 0.94–0.82).

^{1}H NMR of the purified product **1j**:

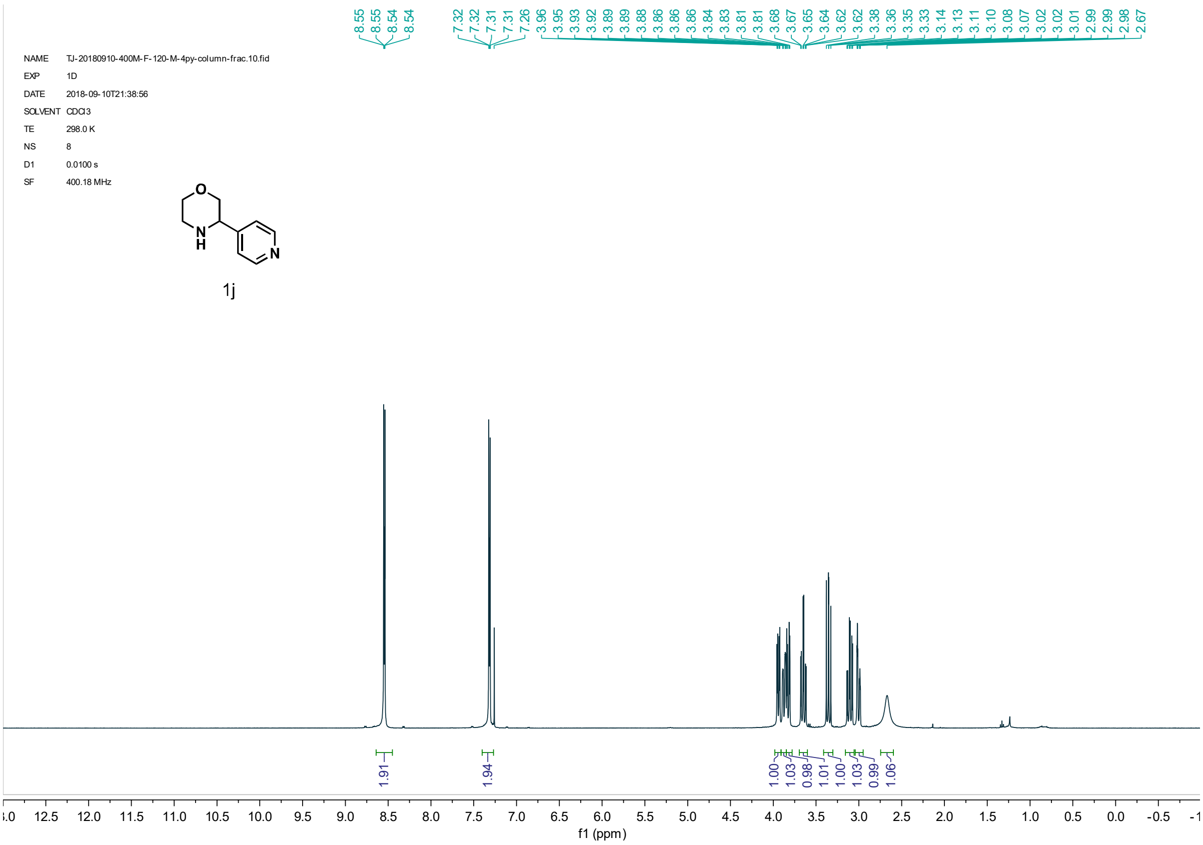

^{13}C NMR of the purified product **1j**:

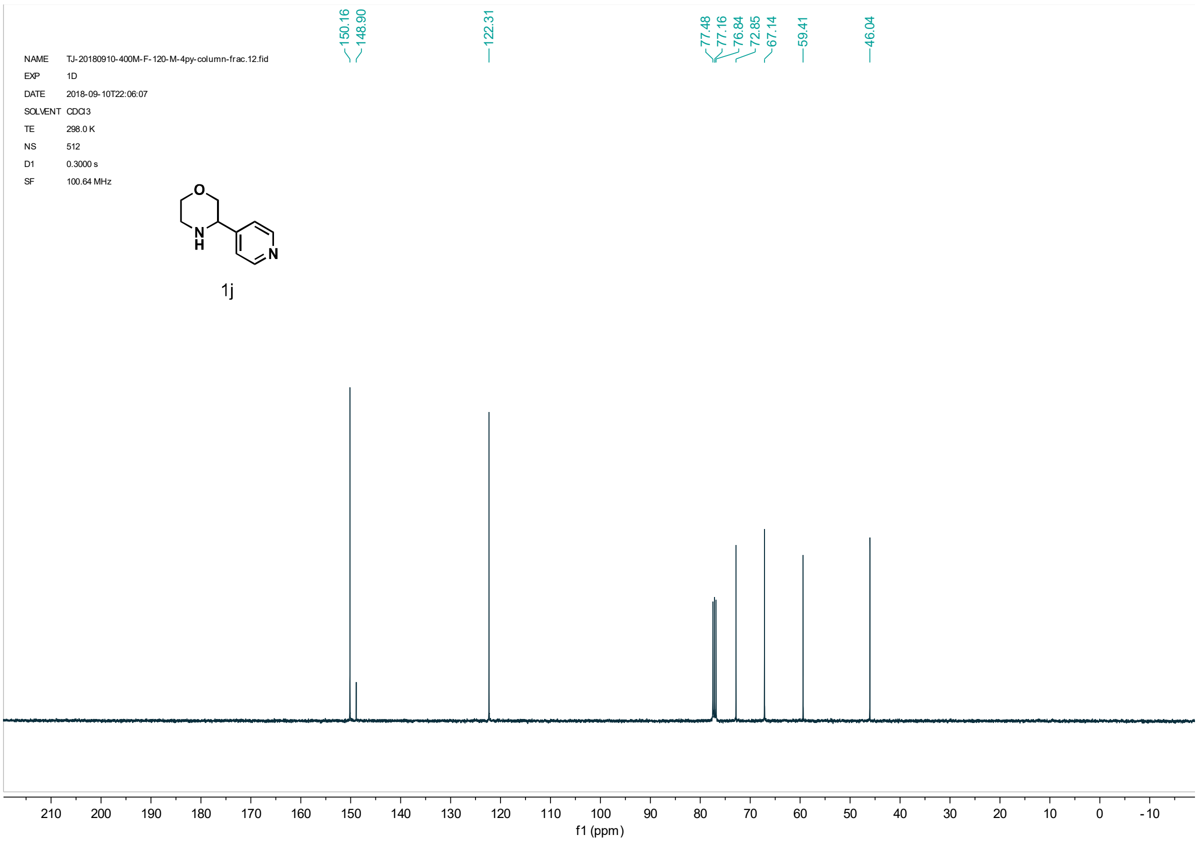

^{1}H NMR of the product **2a** obtained from Synple Chem synthesizer without purification:

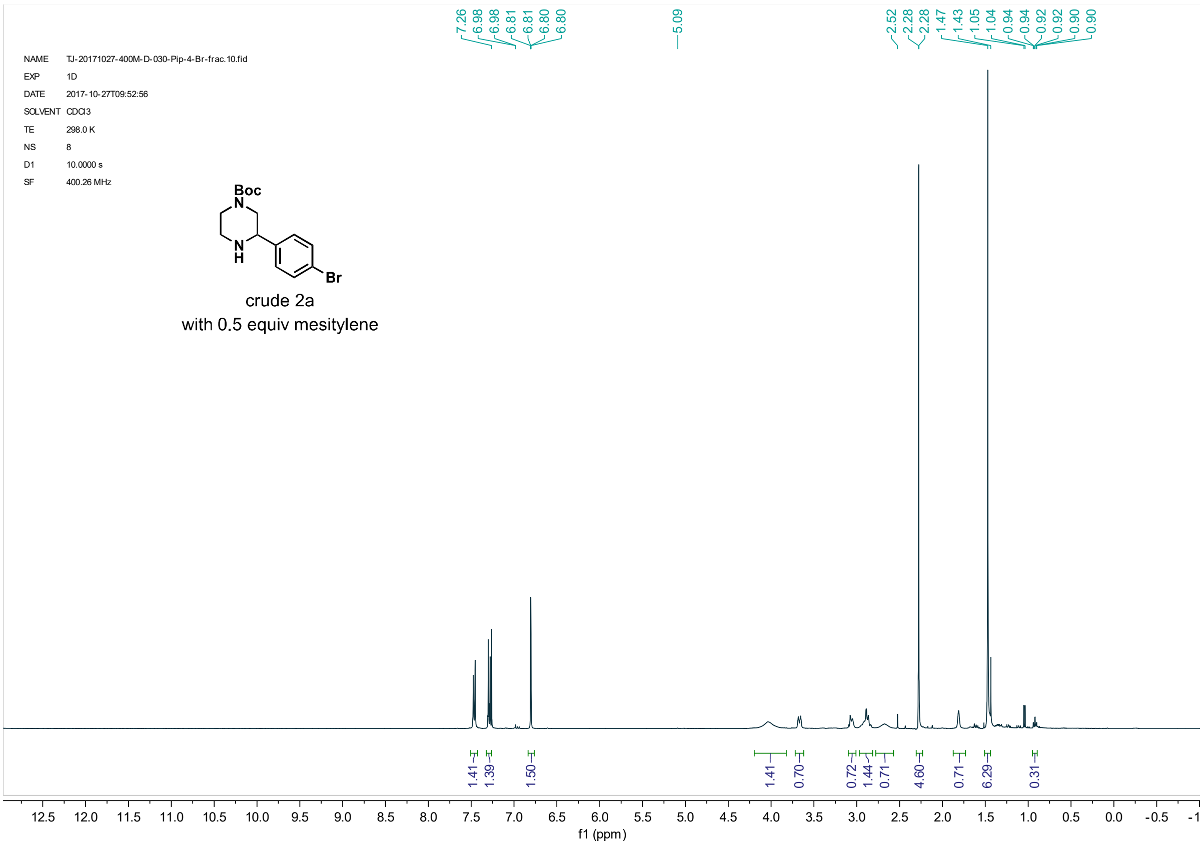

Internal standard: 0.5 equiv mesitylene (δ 6.81–6.80, 2.28). Volatile impurity: DIPA (δ 1.05–1.04). Other impurity: BHT (δ 6.98, 5.09, 2.52, 1.43), “Sn” (δ 0.94–0.90).

^{1}H NMR of the purified product **2a**:

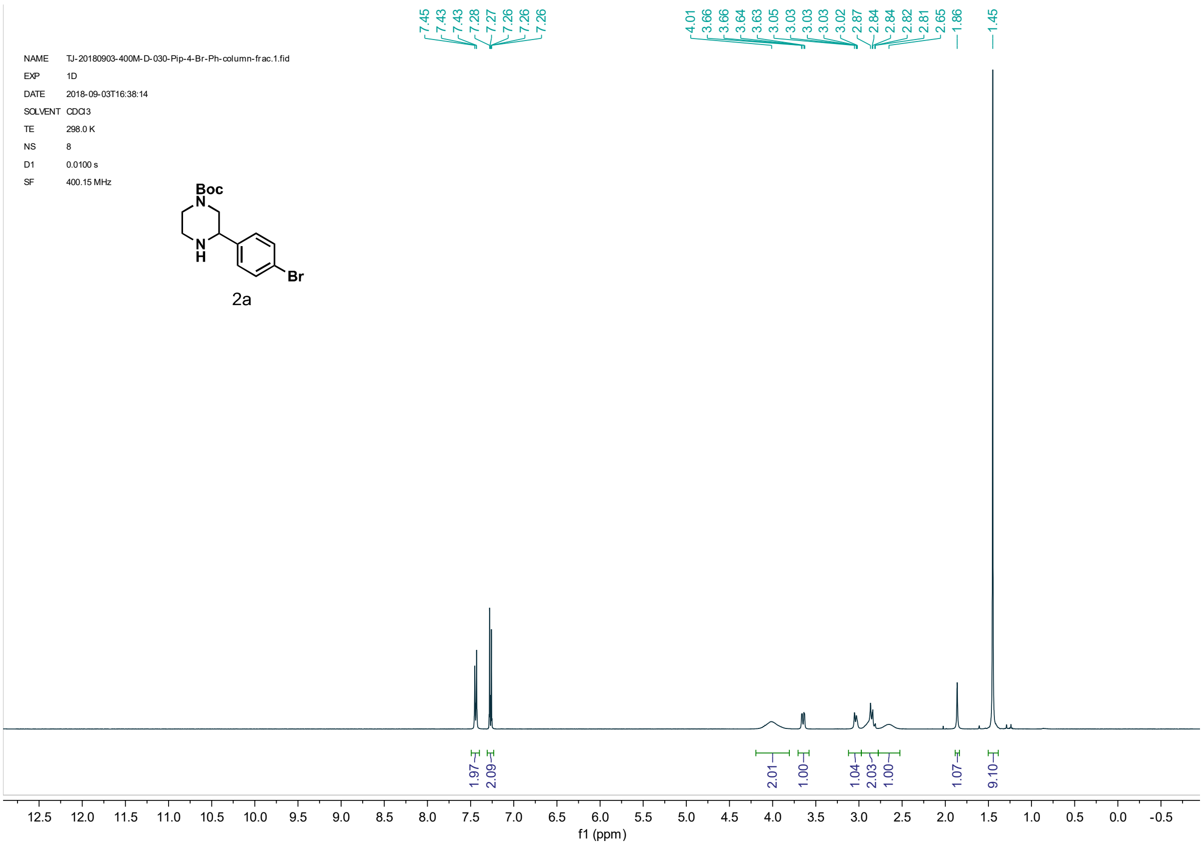

^{13}C NMR of the purified product **2a**:

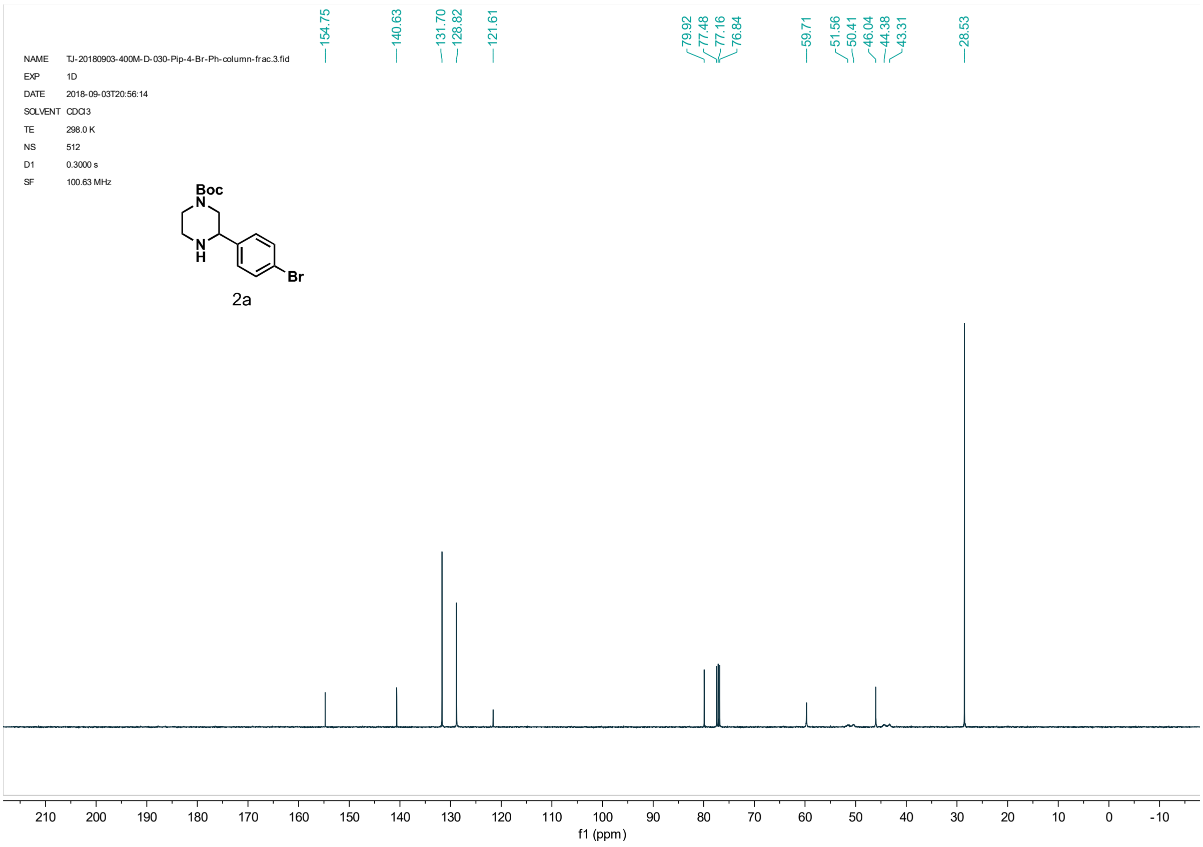

^{1}H NMR of the product **2b** obtained from Synple Chem synthesizer without purification:

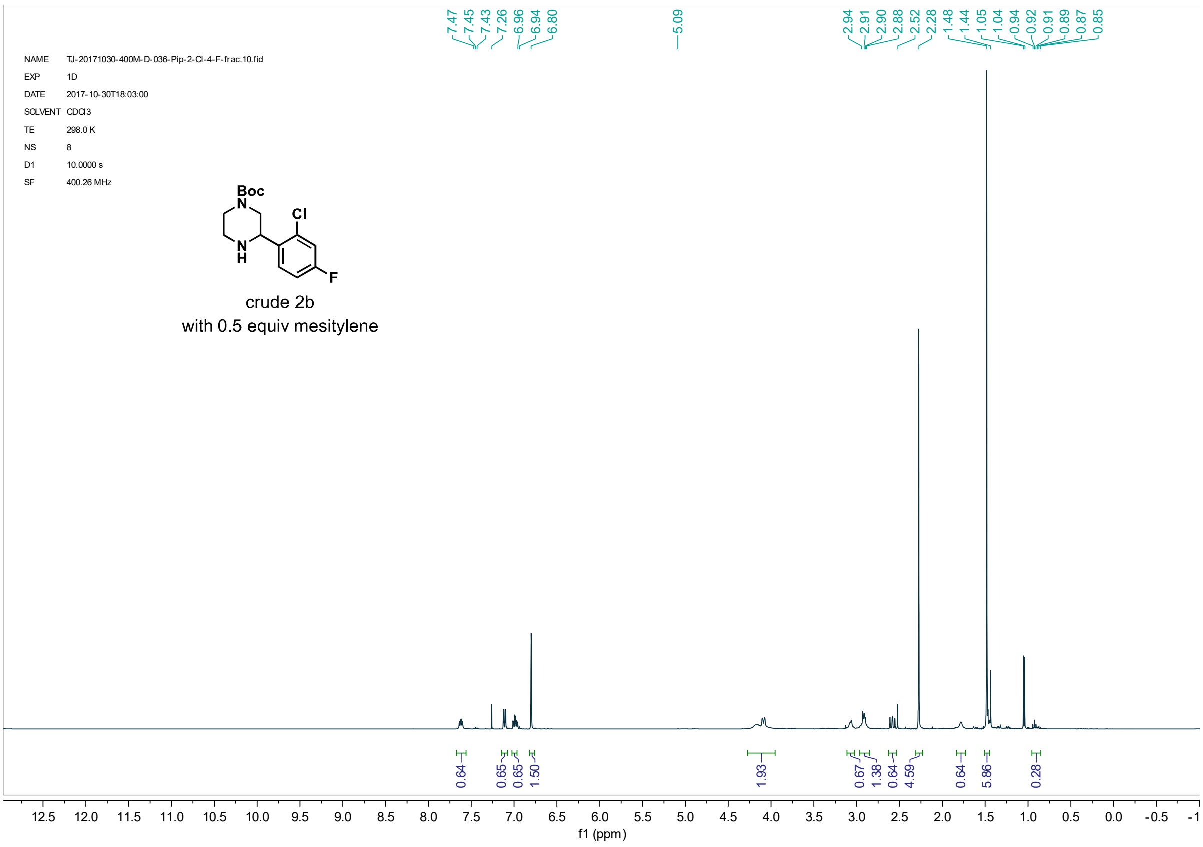

Internal standard: 0.5 equiv mesitylene (δ 6.80, 2.28). Volatile impurity: 2,6-lutidine (δ 7.47–7.43, 6.96–6.94, 2.52), DIPA (δ 1.05–1.04). Other impurity: BHT (δ 6.96, 5.09, 2.52, 1.44), “Sn” (δ 0.94–0.85).

^{1}H NMR of the purified product **2b**:

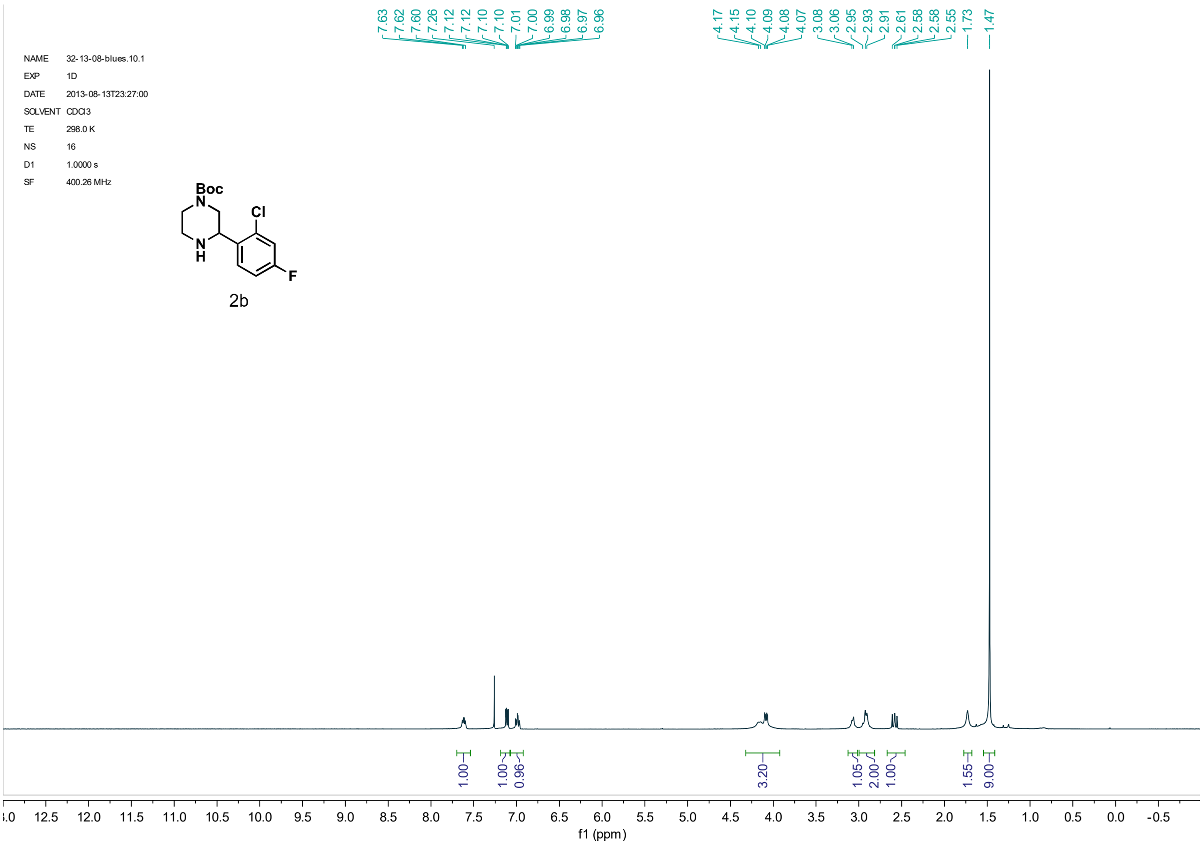

^{1}H NMR of the product **2c** obtained from Synple Chem synthesizer without purification:

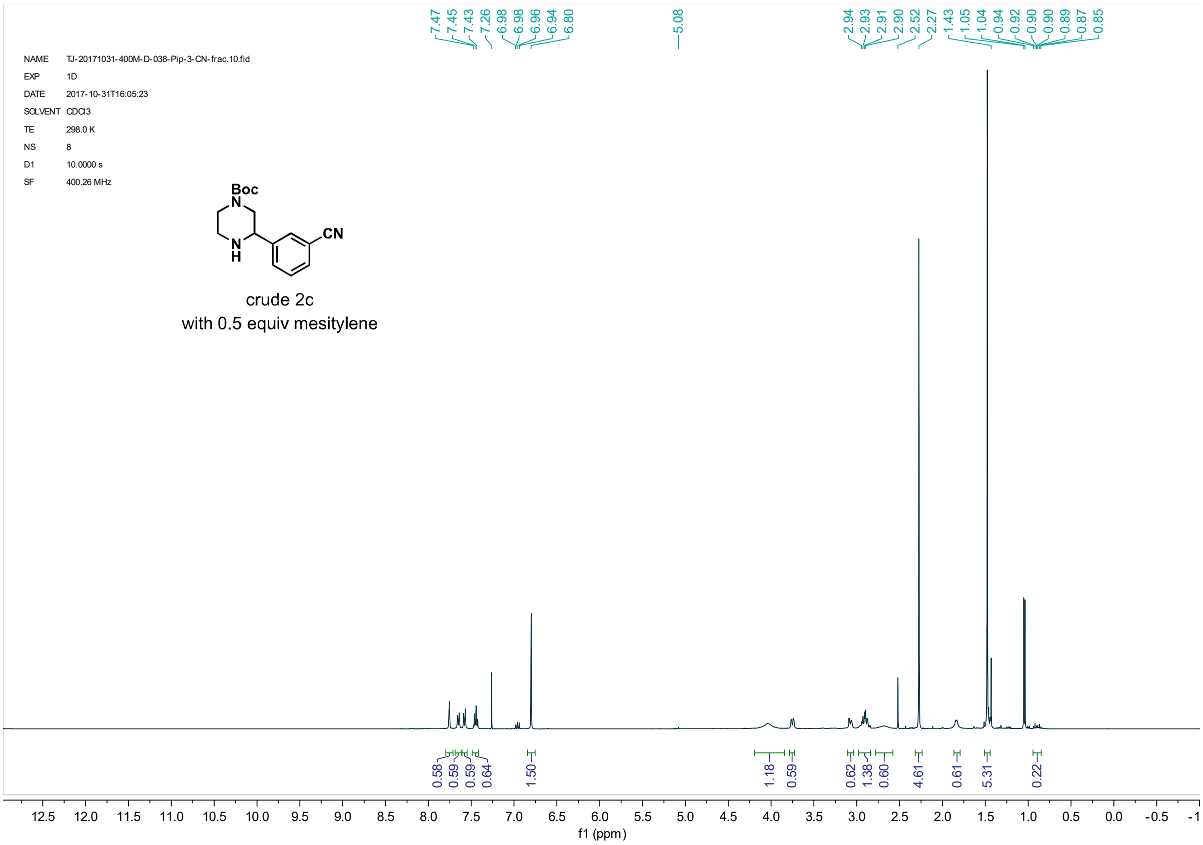

Internal standard: 0.5 equiv mesitylene (δ 6.80, 2.27). Volatile impurity: 2,6-lutidine (δ 7.47–7.43, 6.96–6.94, 2.52), DIPA (δ 2.94–2.90, 1.05–1.04). Other impurity: BHT (δ 6.98, 5.08, 2.52, 1.43), "Sn" (δ 0.94–0.85).

^{1}H NMR of the purified product **2c**:

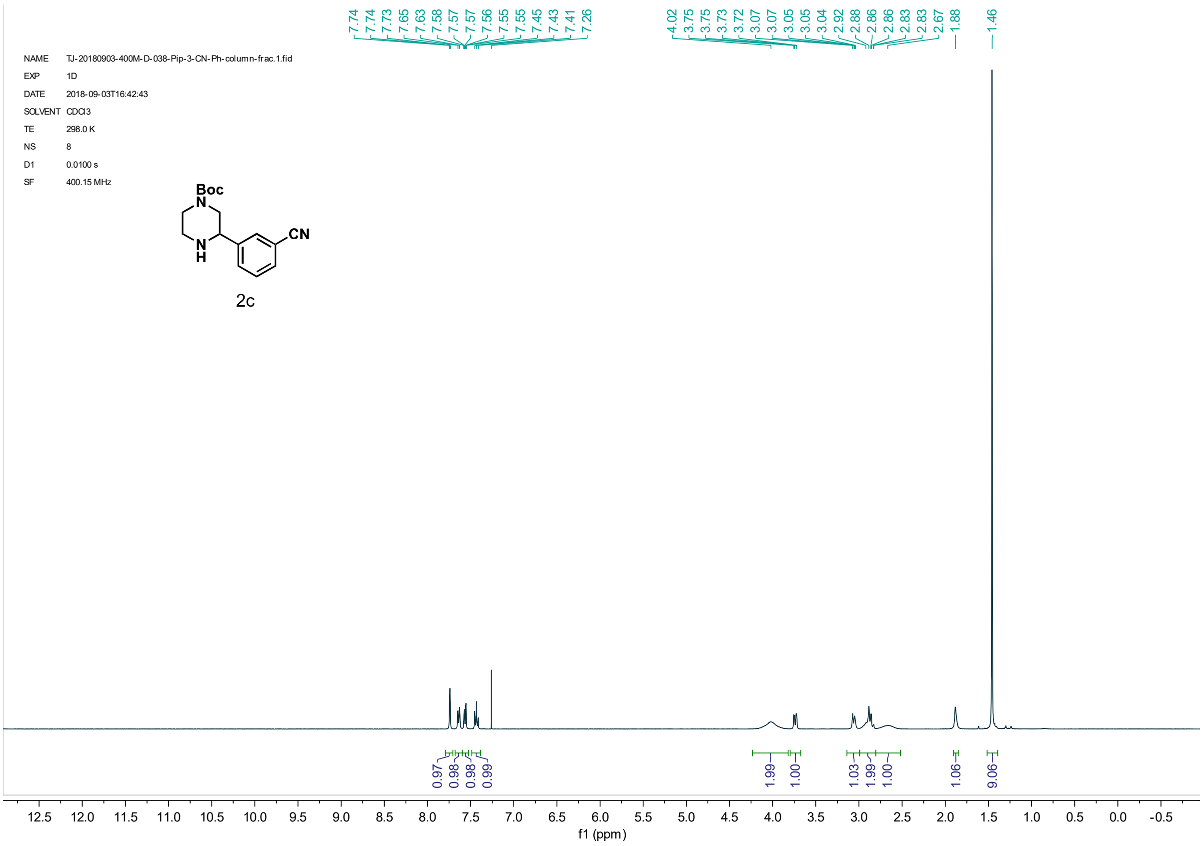

^{13}C NMR of the purified product **2c**:

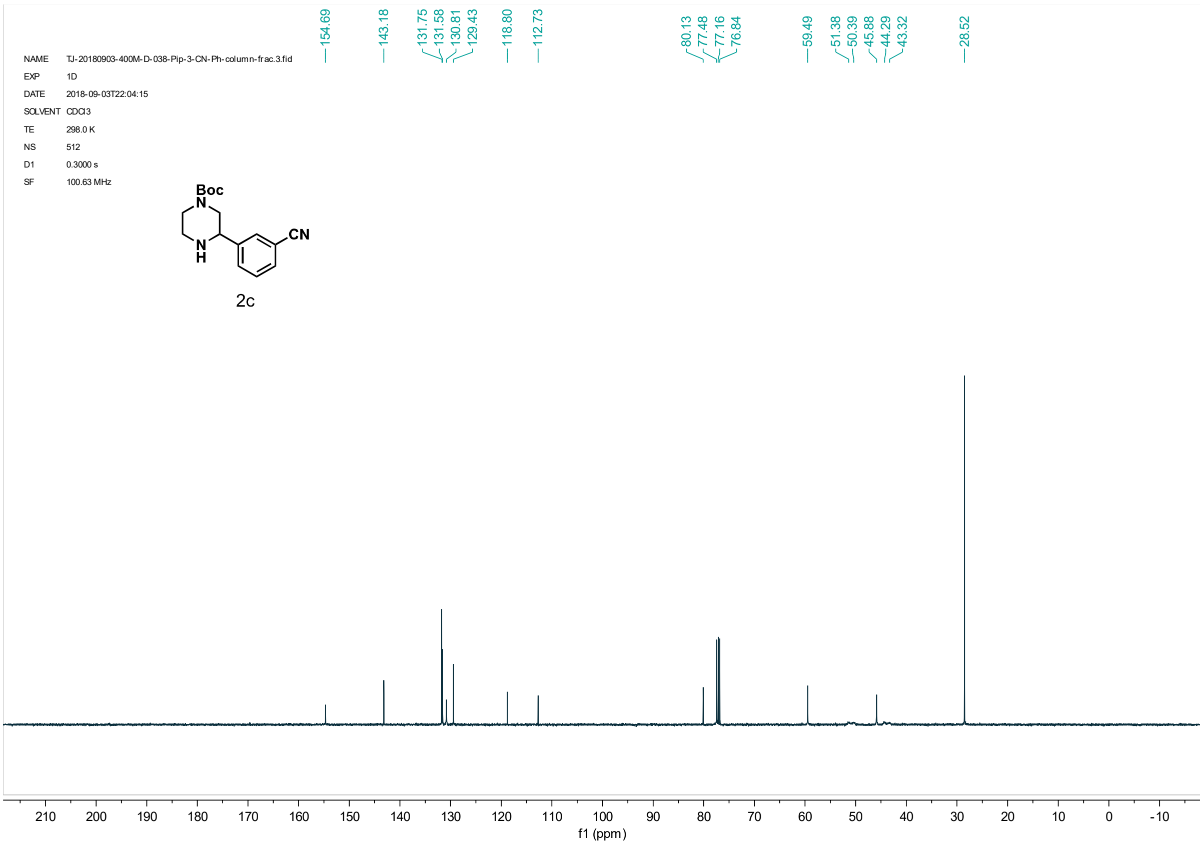

^{1}H NMR of the product **2d** obtained from Synple Chem synthesizer without purification:

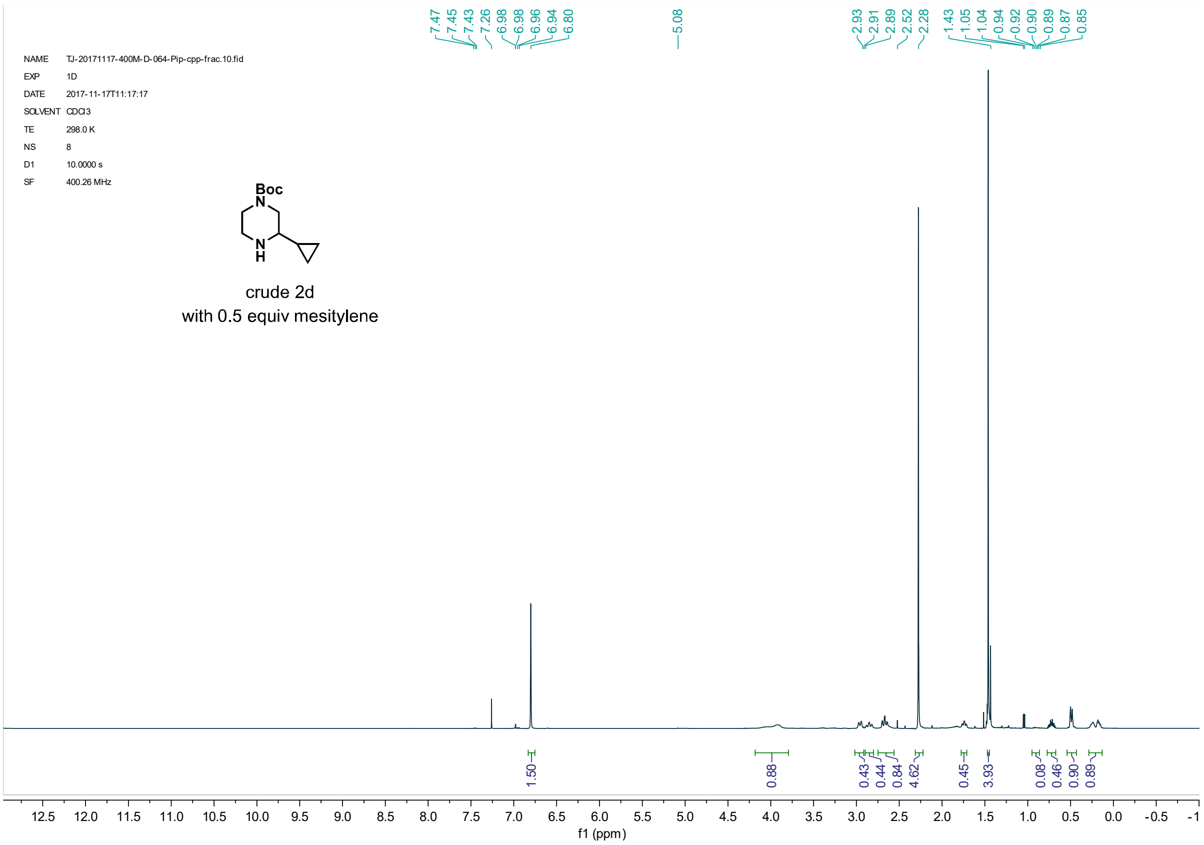

Internal standard: 0.5 equiv mesitylene (δ 6.80, 2.28). Volatile impurity: 2,6-lutidine (δ 7.47–7.43, 6.96–6.94, 2.52), DIPA (δ 2.93–2.89, 1.05–1.04). Other impurity: BHT (δ 6.98, 5.08, 2.52, 1.43), “Sn” (δ 0.94–0.85).

^{1}H NMR of the purified product **2d**:

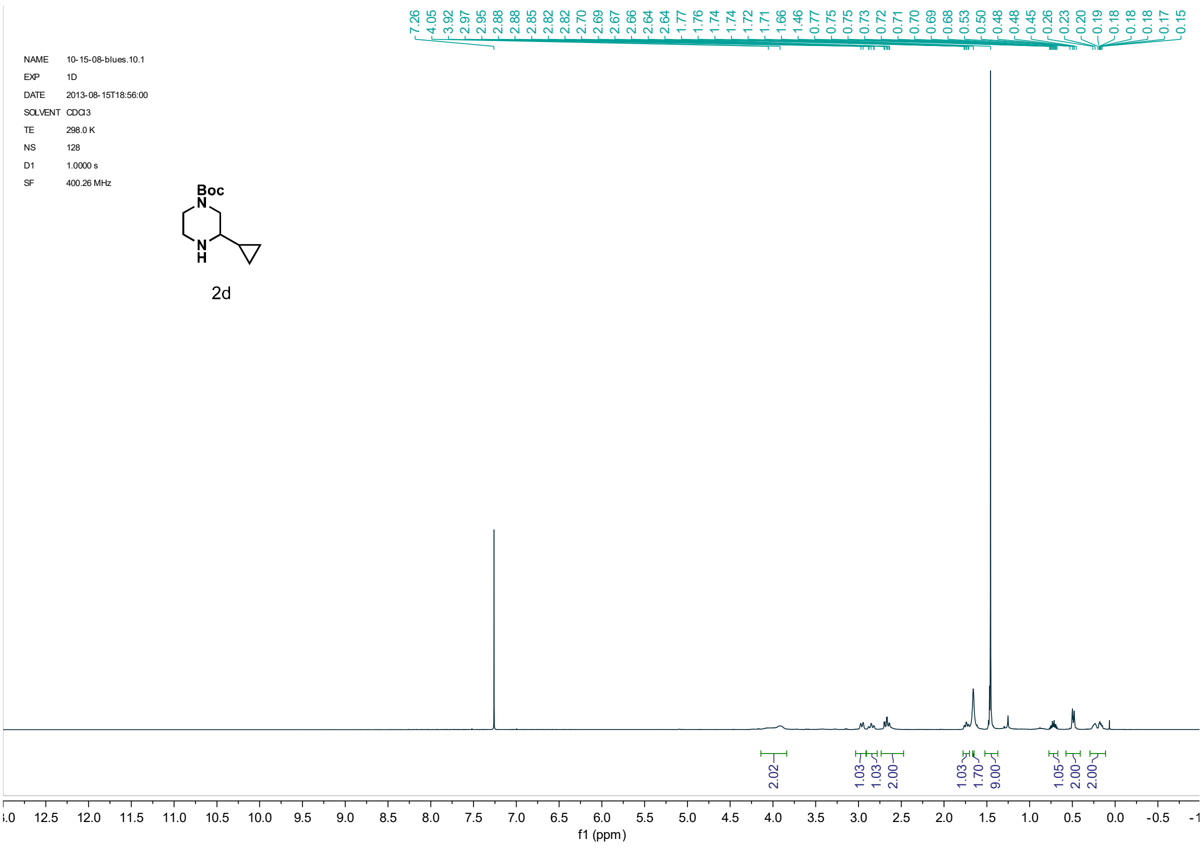

^{1}H NMR of the product **2e** obtained from Synple Chem synthesizer without purification:

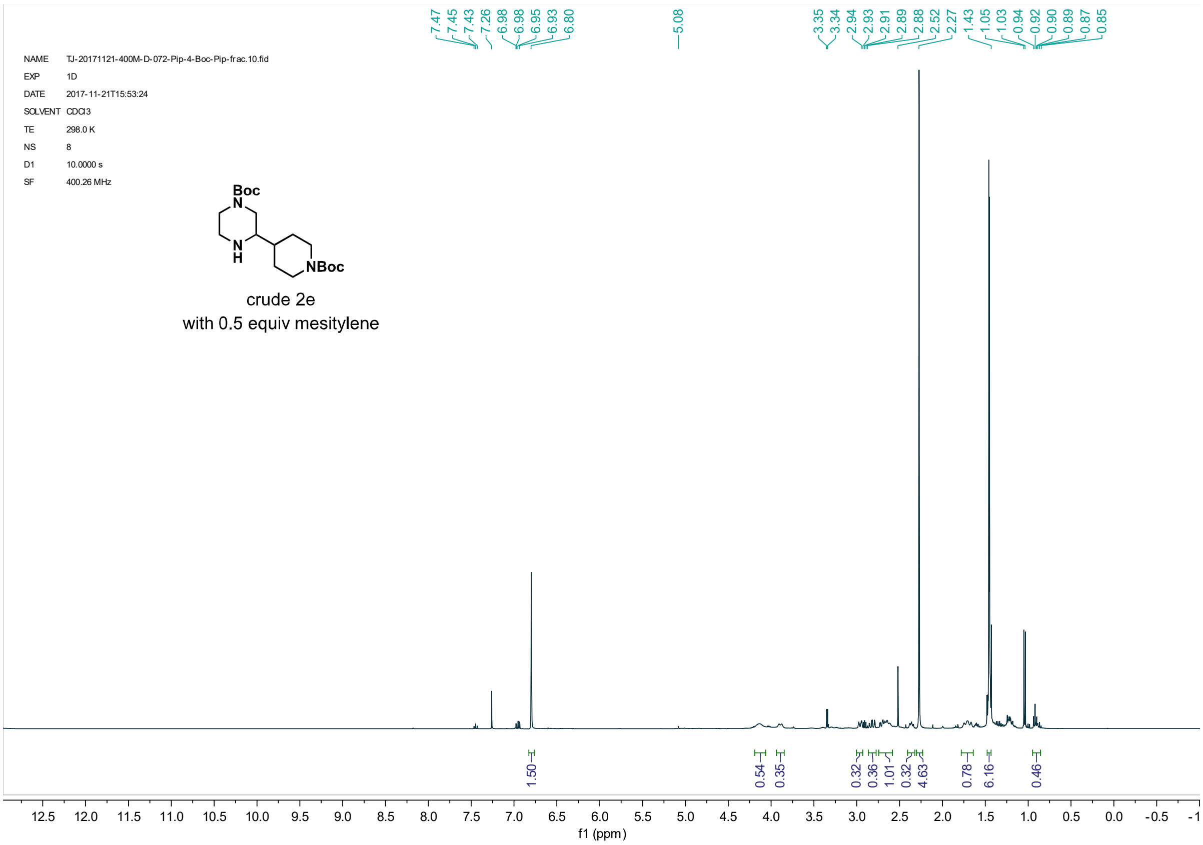

Internal standard: 0.5 equiv mesitylene (δ 6.80, 2.27). Volatile impurity: 2,6-lutidine (δ 7.47–7.43, 6.95–6.93, 2.52), MeOH (δ 3.35–3.34), DIPA (δ 2.94–2.88, 1.05–1.03). Other impurity: BHT (δ 6.98, 5.08, 2.52, 1.43), "Sn" (δ 0.94–0.85).

^{1}H NMR of the purified product **2e**:

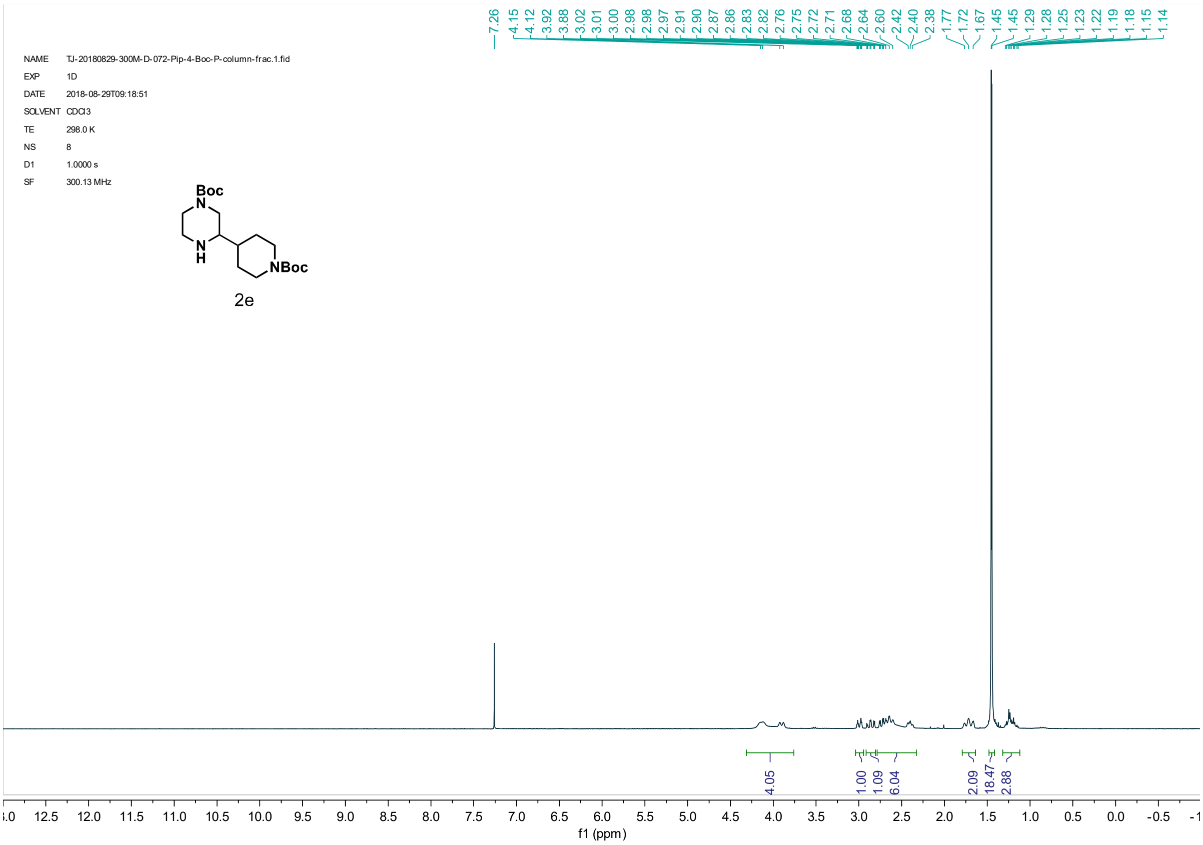

^{1}H NMR of the product **2f** obtained from Synple Chem synthesizer without purification:

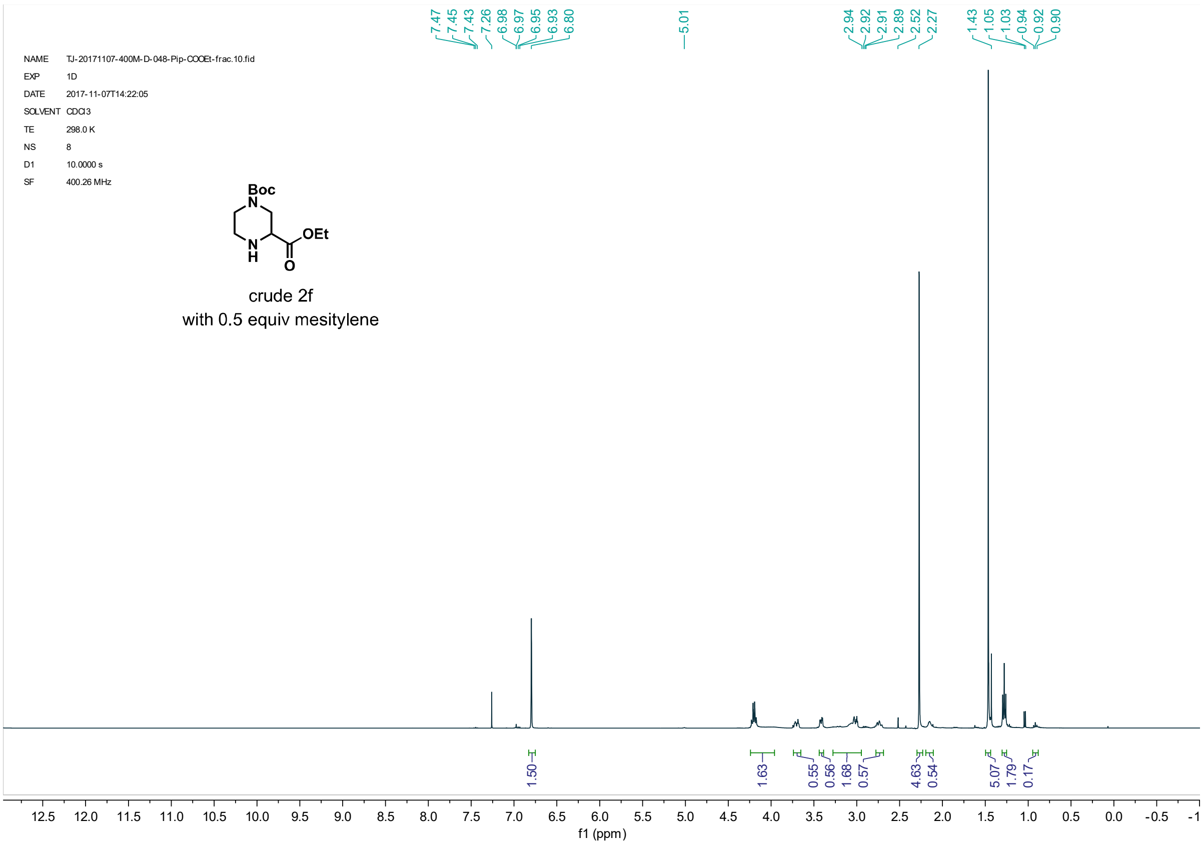

Internal standard: 0.5 equiv mesitylene (δ 6.80, 2.27). Volatile impurity: 2,6-lutidine (δ 7.47–7.43, 6.95–6.93, 2.52), DIPA (δ 2.94–2.89, 1.05–1.03). Other impurity: BHT (δ 6.98–6.97, 5.01, 2.52, 1.43), “Sn” (δ 0.94–0.90).

^{1}H NMR of the purified product **2f**:

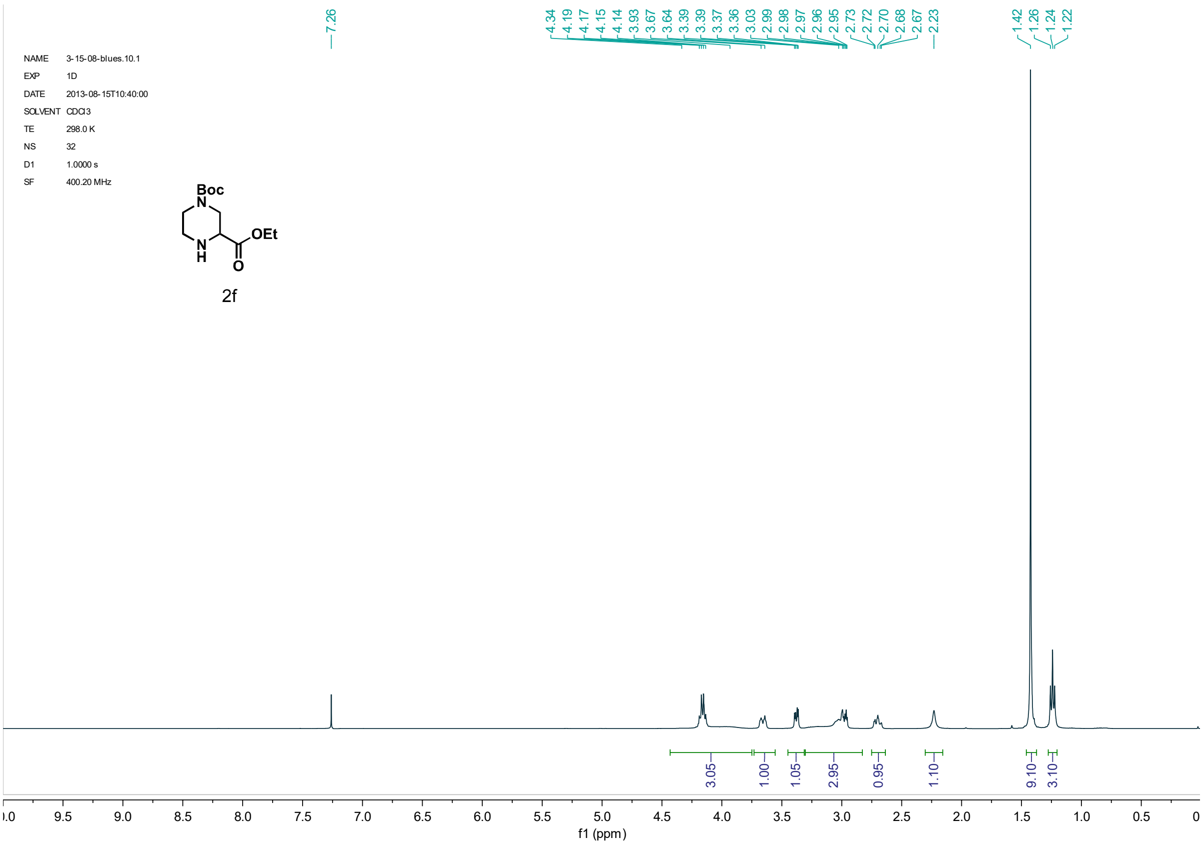

^{1}H NMR of the product **2g** obtained from Synple Chem synthesizer without purification:

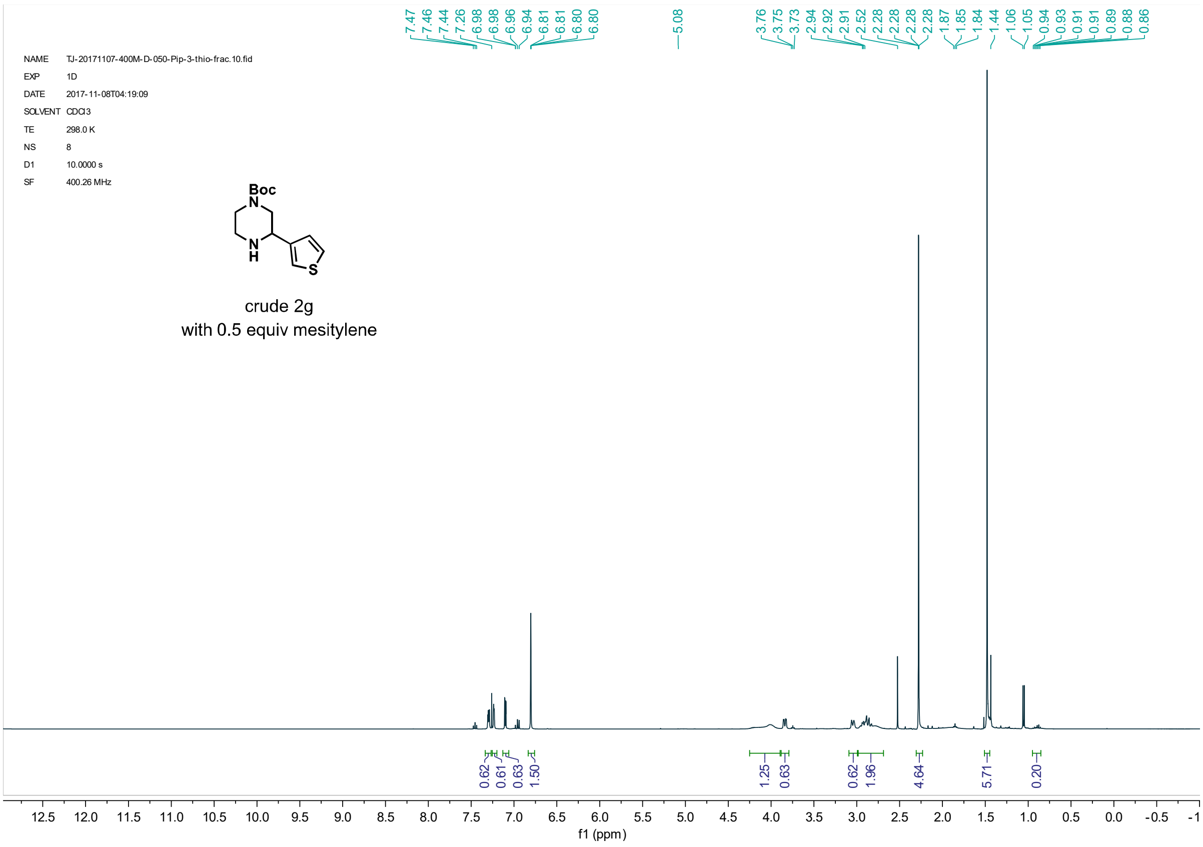

Internal standard: 0.5 equiv mesitylene (*δ* 6.81–6.80, 2.28). Volatile impurity: 2,6-lutidine (*δ* 7.47–7.44, 6.96–6.94, 2.52), THF (*δ* 3.76–3.73, 1.87–1.84), DIPA (*δ* 1.06–1.05). Other impurity: BHT (*δ* 6.98, 5.08, 2.52, 1.44), “Sn” (*δ* 0.94–0.86).

^{1}H NMR of the purified product **2g**:

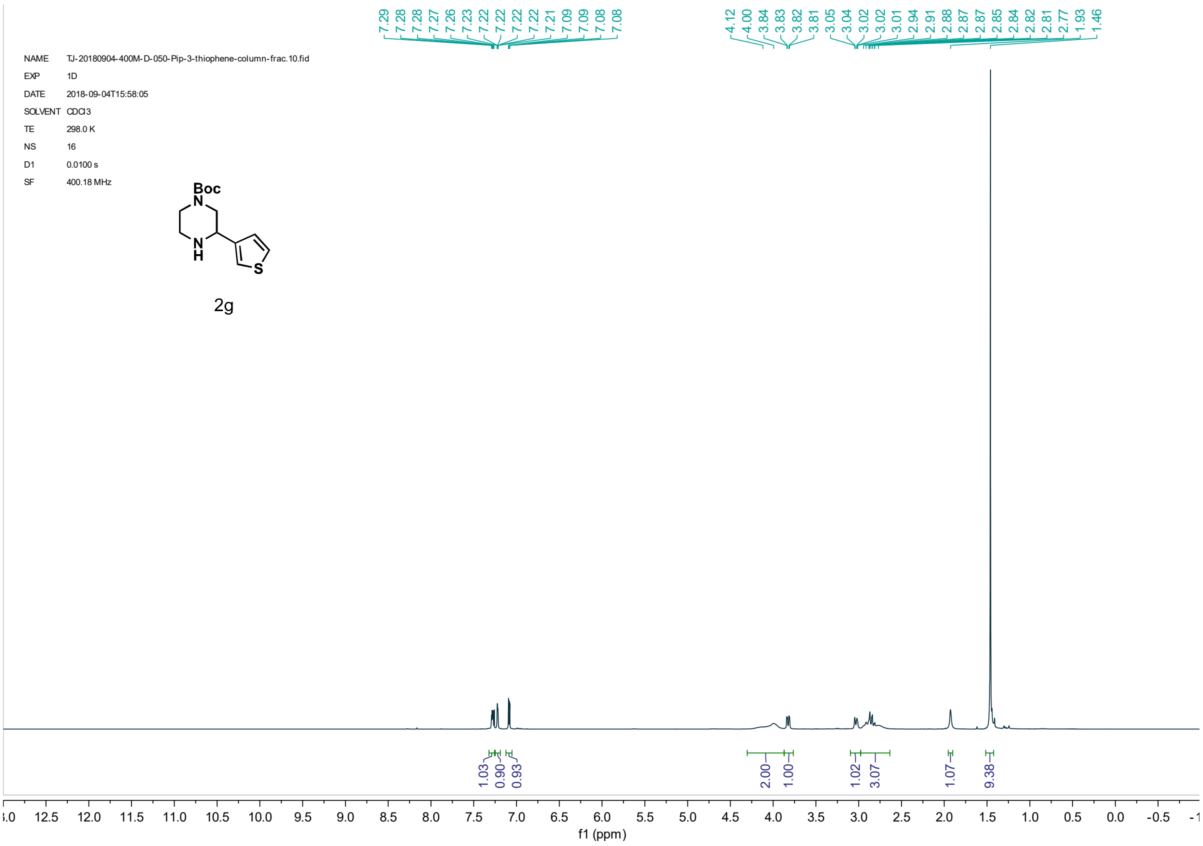

^{13}C NMR of the purified product **2g**:

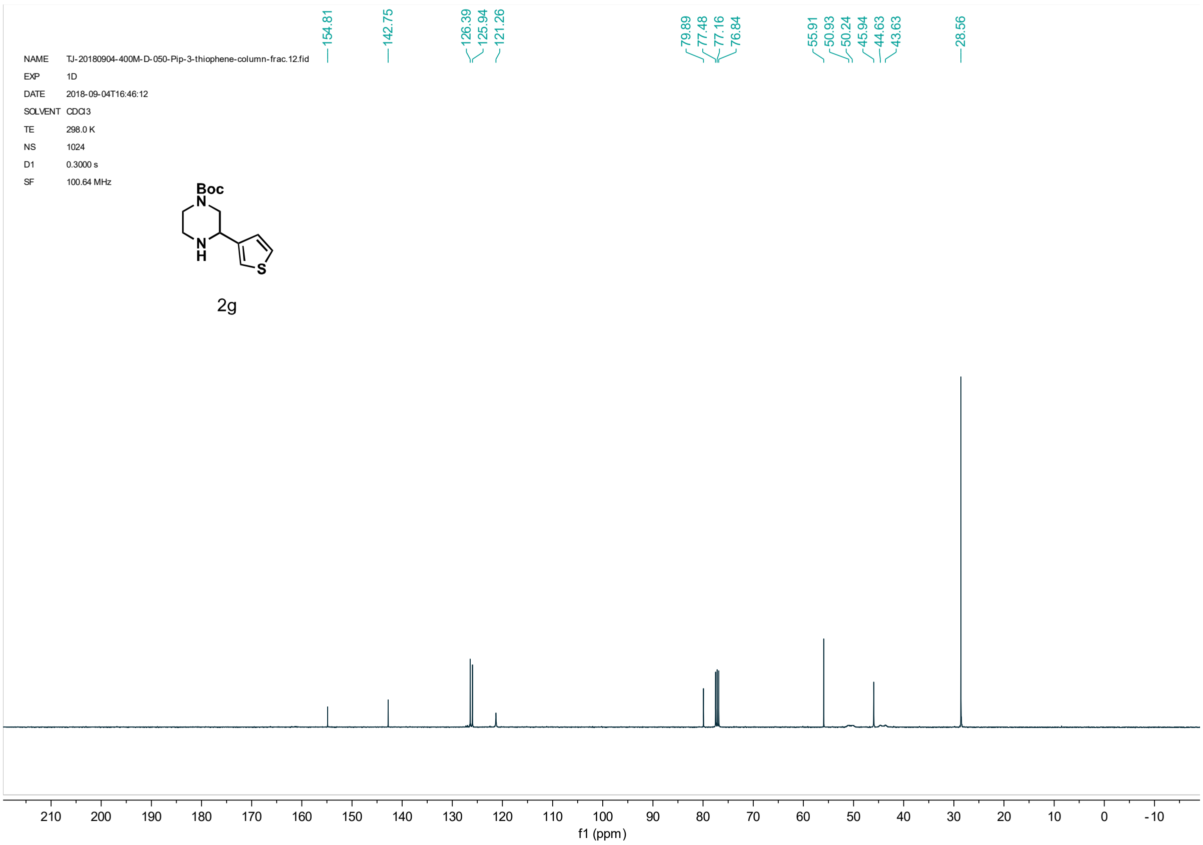

^{1}H NMR of the product **2h** obtained from Synple Chem synthesizer without purification:

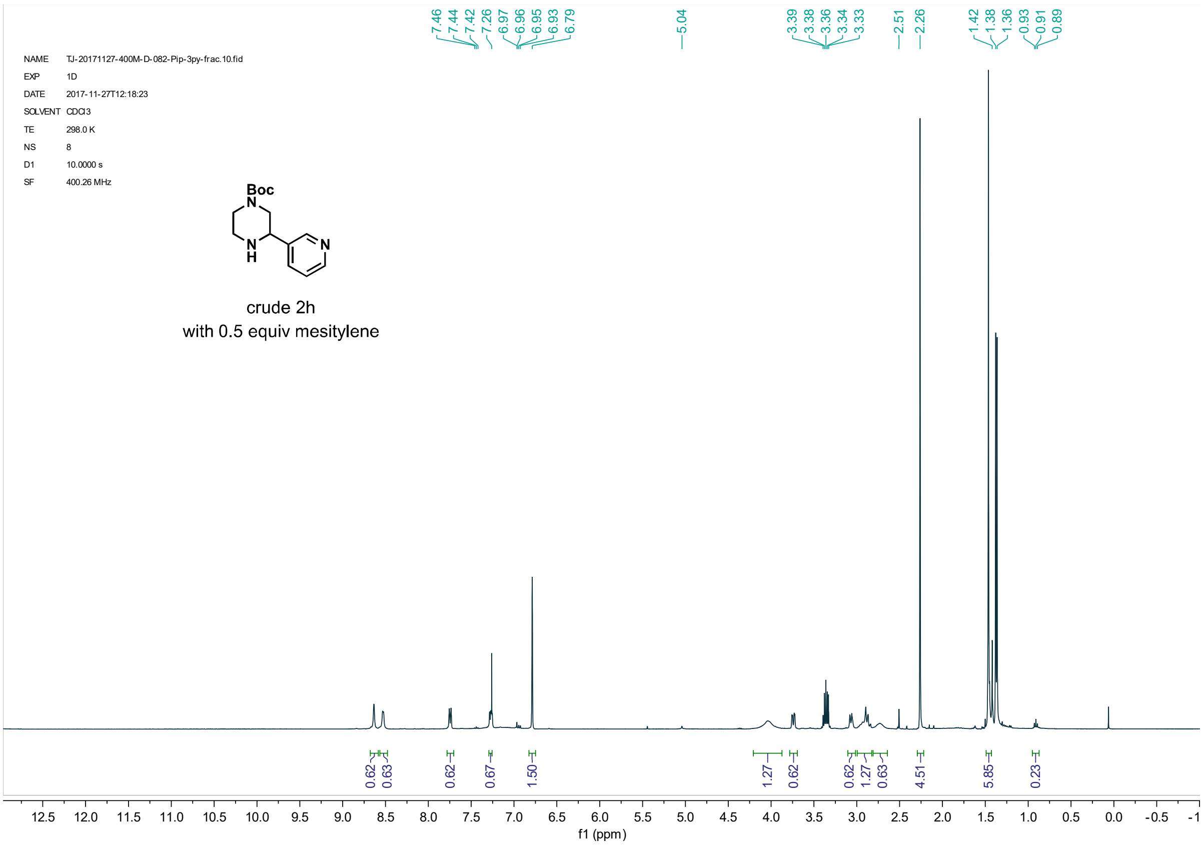

Internal standard: 0.5 equiv mesitylene (*δ* 6.79, 2.26). Volatile impurity: 2,6-lutidine (*δ* 7.46–7.42, 6.95–6.93, 2.51). Other impurity: DIPA·HX (*δ* 3.39–3.33, 1.38–1.36), BHT (*δ* 6.97–6.96, 5.04, 2.51, 1.42), “Sn” (*δ* 0.93–0.89).

^{1}H NMR of the purified product **2h**:

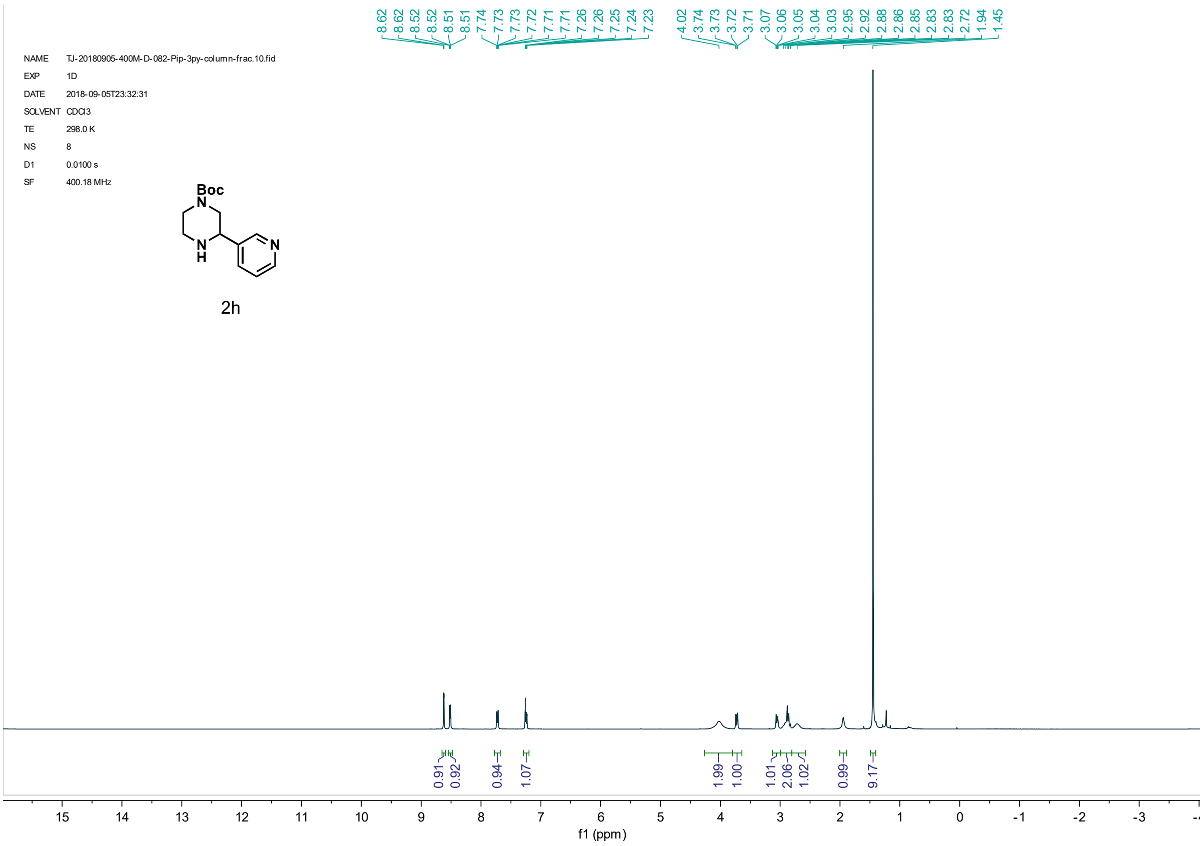

^{13}C NMR of the purified product **2h**:

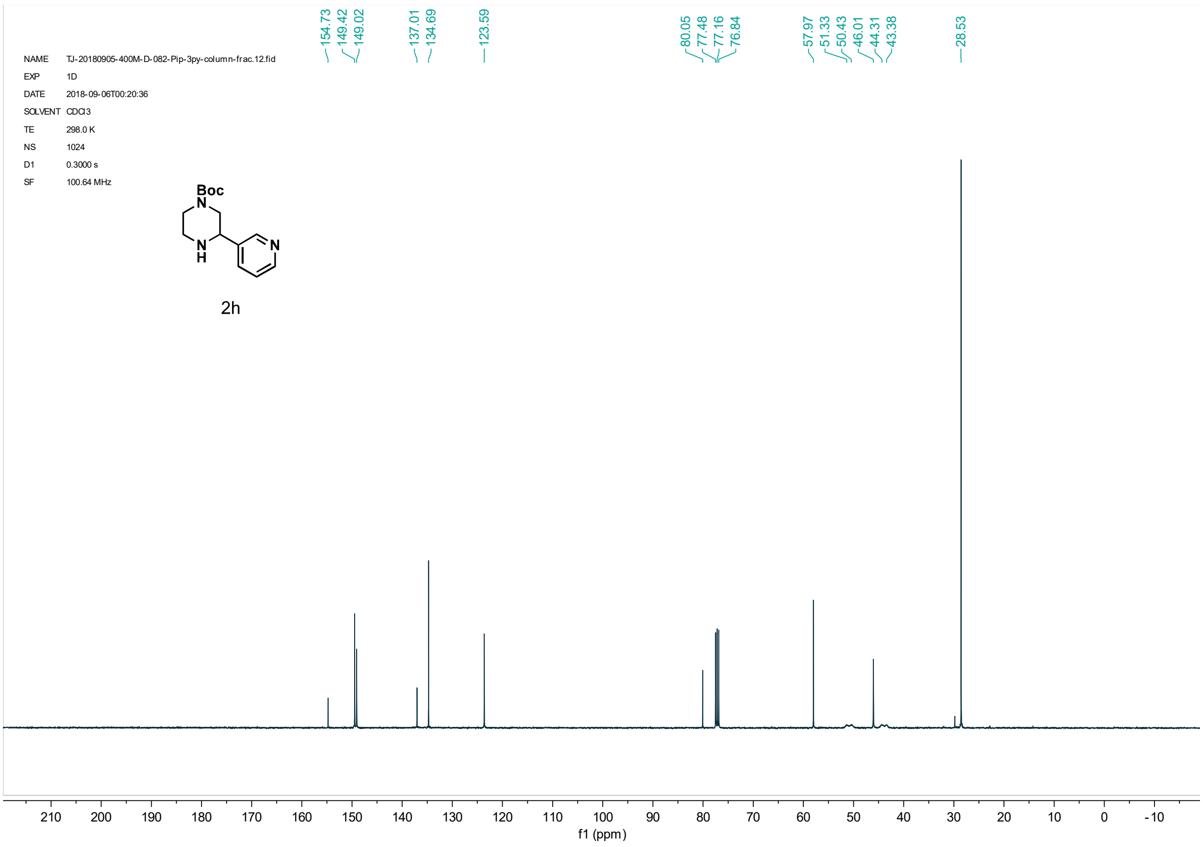

^{1}H NMR of the product **2i** obtained from Synple Chem synthesizer without purification:

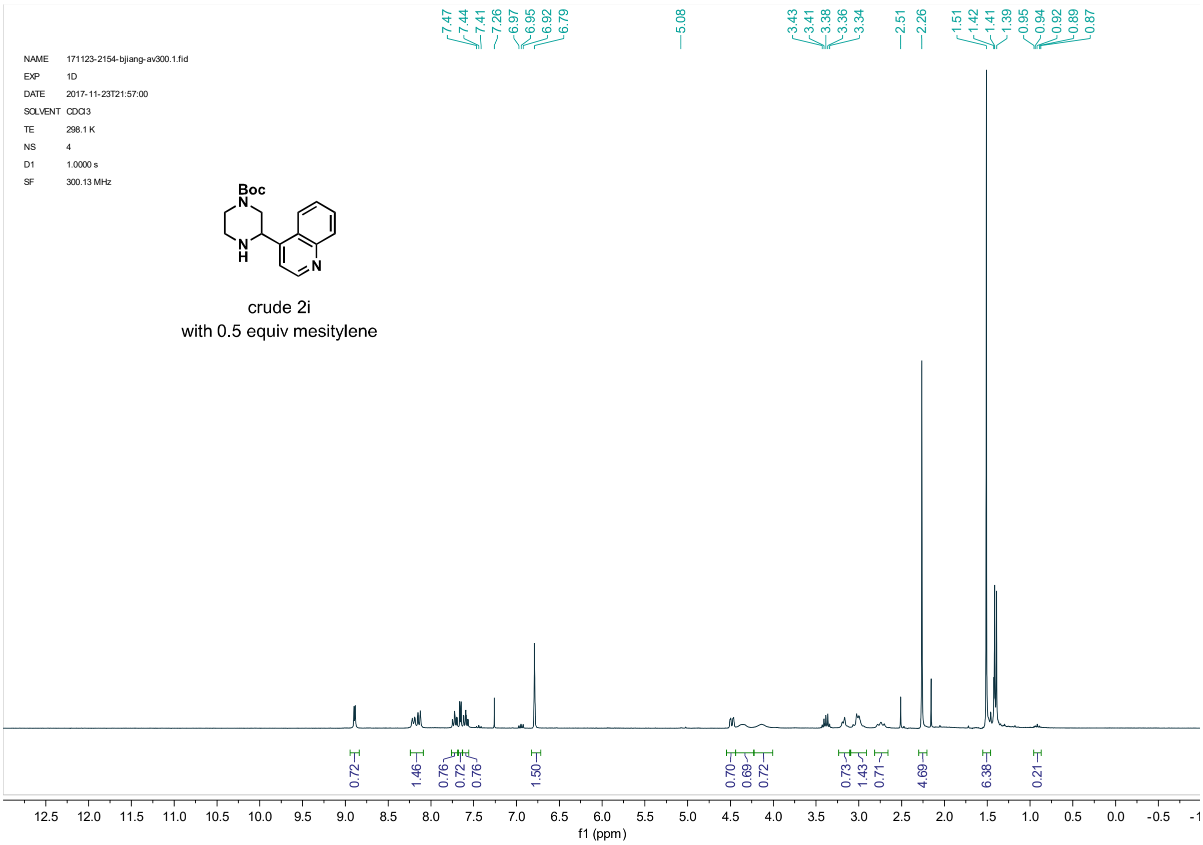

Internal standard: 0.5 equiv mesitylene (δ 6.79, 2.26). Volatile impurity: 2,6-lutidine (δ 7.47–7.41, 6.95–6.92, 2.51). Other impurity: DIPA·HX (δ 3.43–3.34, 1.41–1.1.39), BHT (δ 6.97, 5.08, 2.51, 1.42), "Sn" (δ 0.95–0.87).

^{1}H NMR of the purified product **2i**:

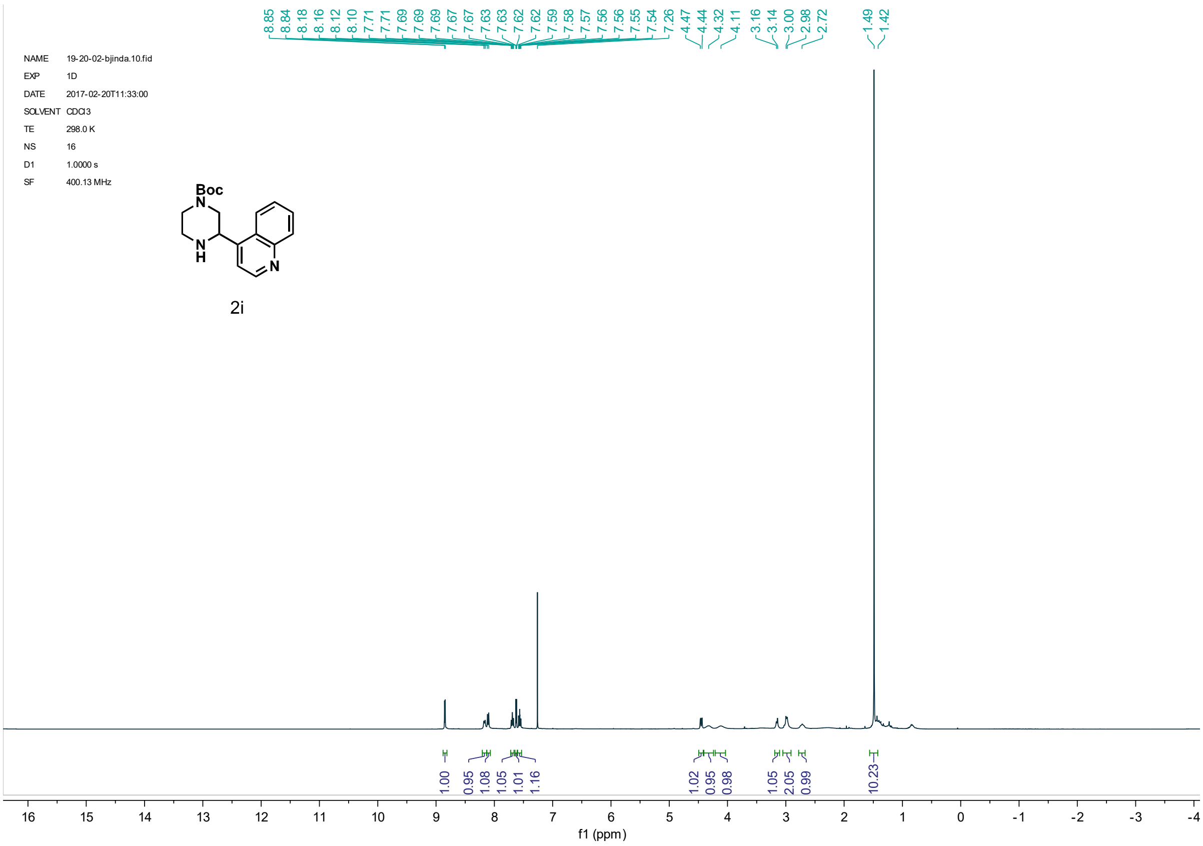

^{1}H NMR of the product **2j** obtained from Synple Chem synthesizer without purification:

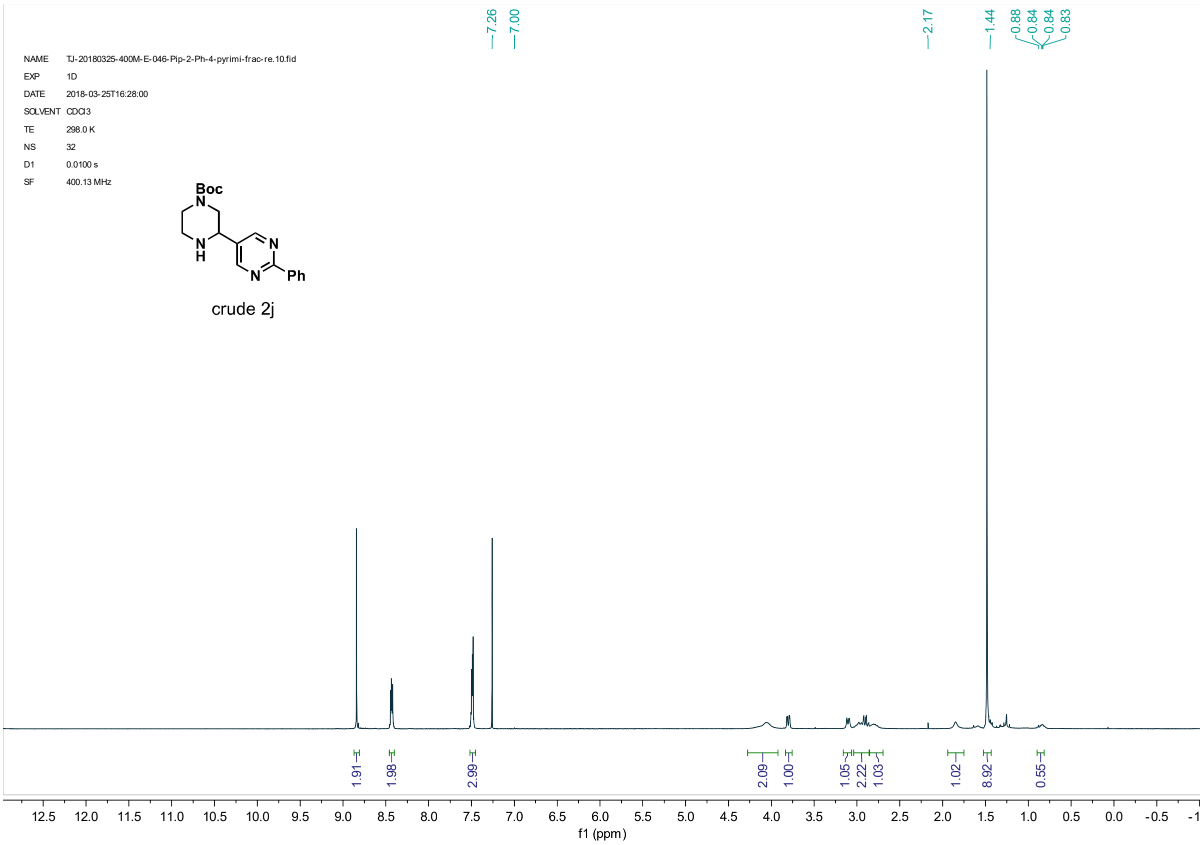

Internal standard: - .Volatile impurity: - . Other impurity: BHT (δ 7.00, 2.17, 1.44), “Sn” (δ 0.88–0.83).

^{1}H NMR of the purified product **2j**:

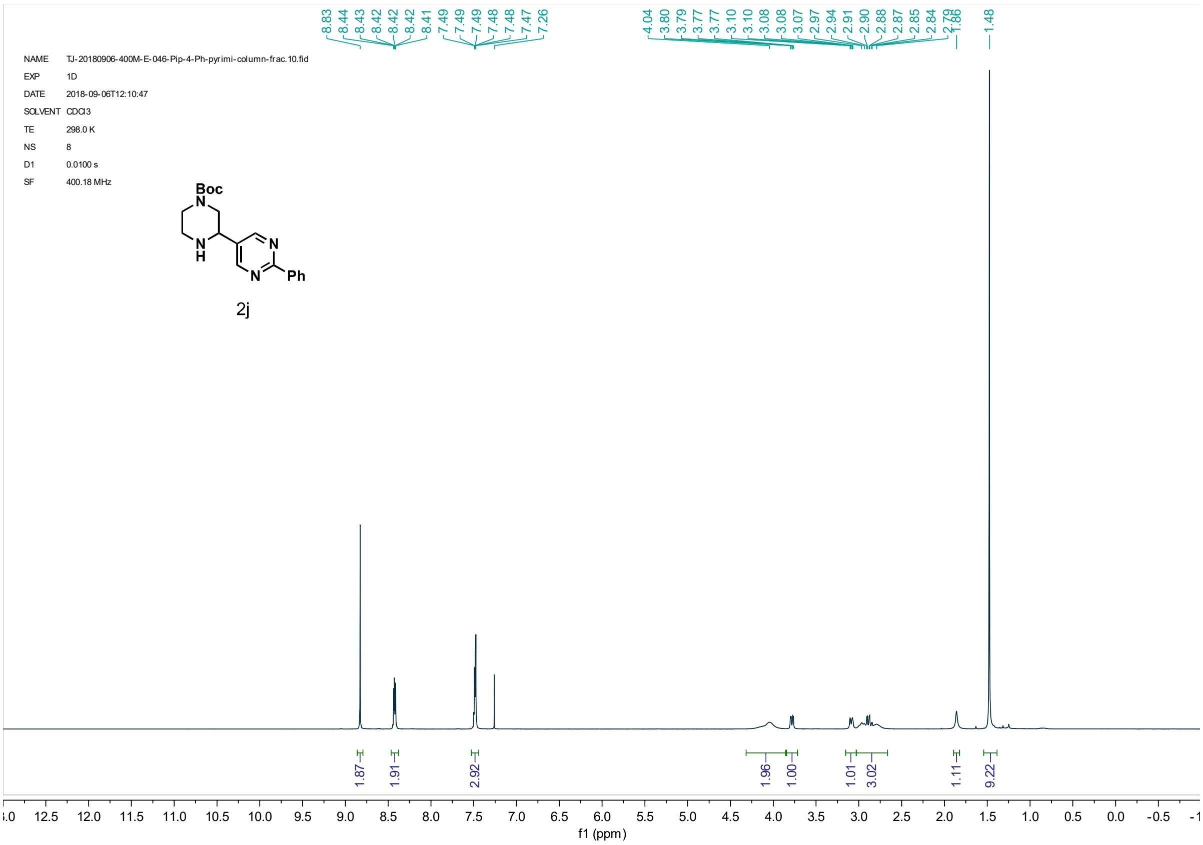

^{13}C NMR of the purified product **2j**:

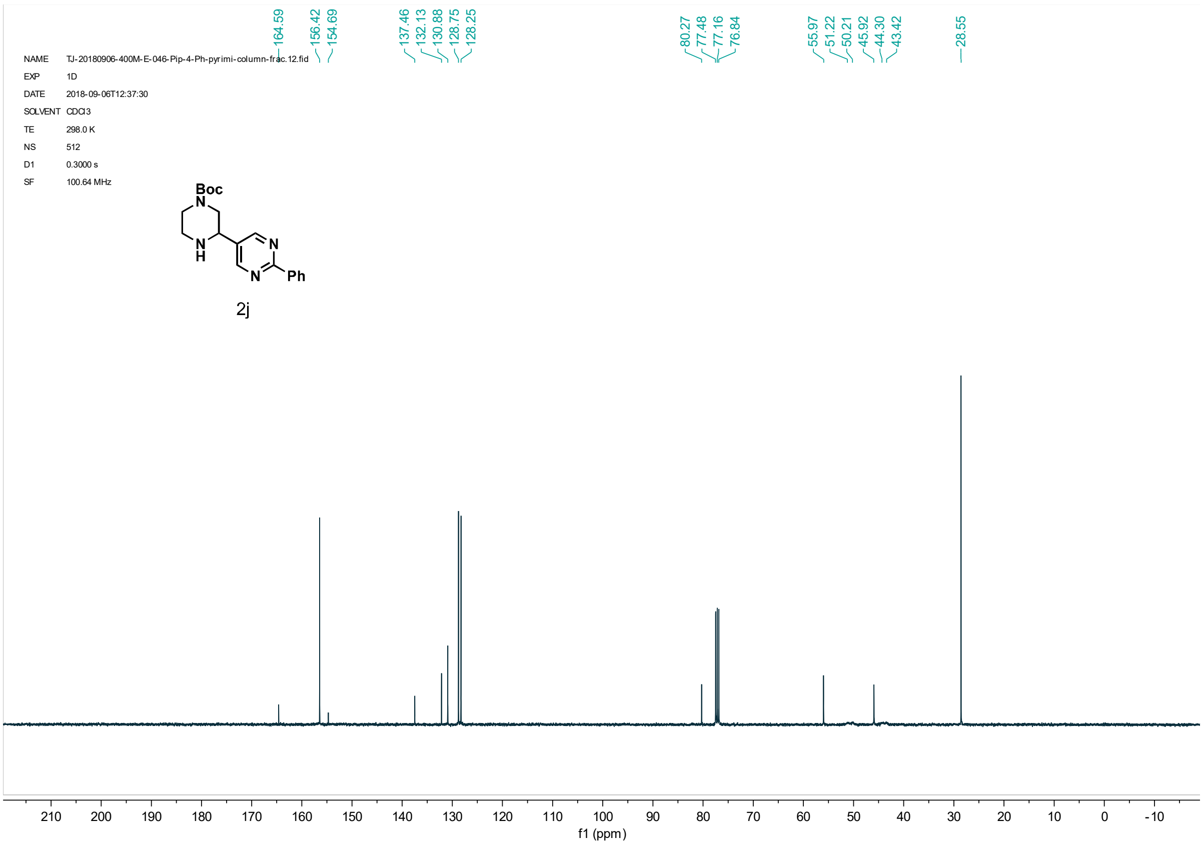

^{1}H NMR of the product **2k** obtained from Synple Chem synthesizer without purification:

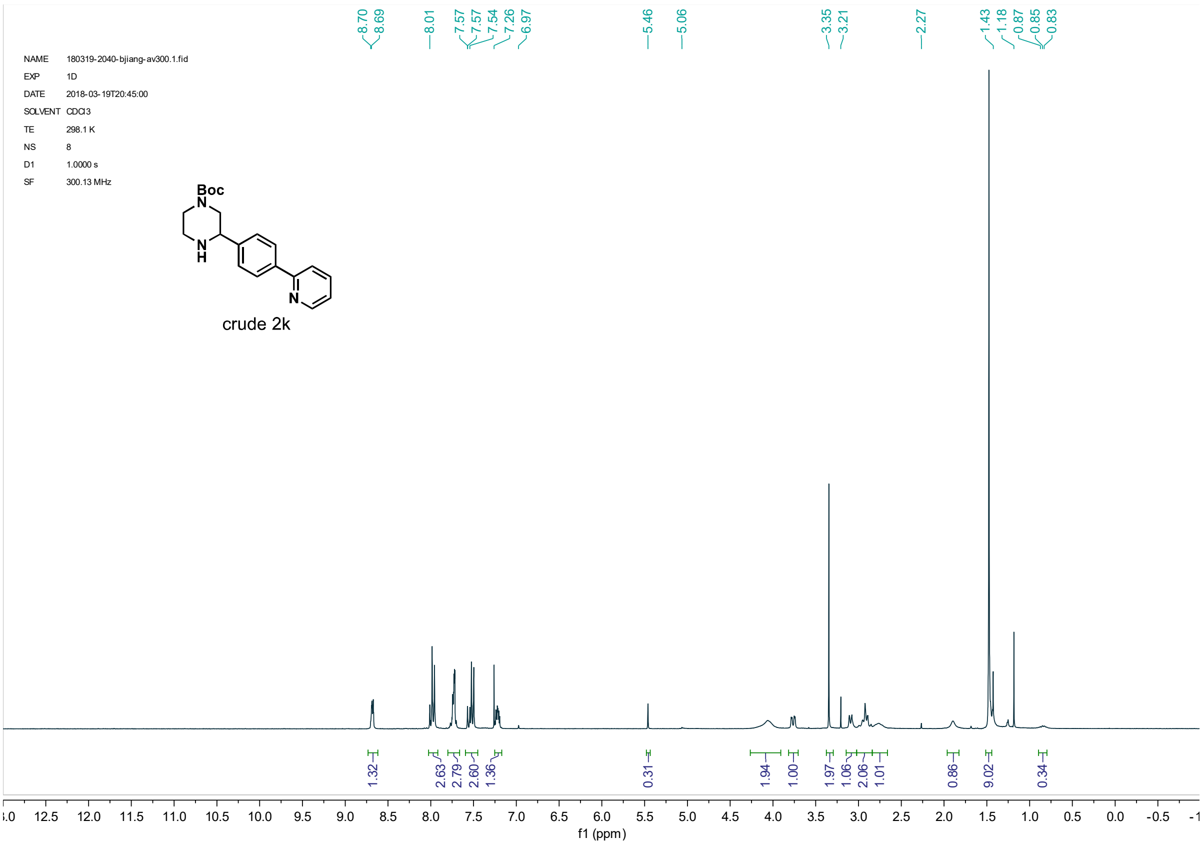

Internal standard: - . Volatile impurity: MeOH (δ 3.21). Other impurity: Me-acetal (δ 8.70–8.69, 8.01, 7.57–7.54, 5.46, 3.35), BHT (δ 6.97, 5.06, 2.27, 1.43), "Sn" (δ 0.87–0.83).

^{1}H NMR of the purified product **2k**:

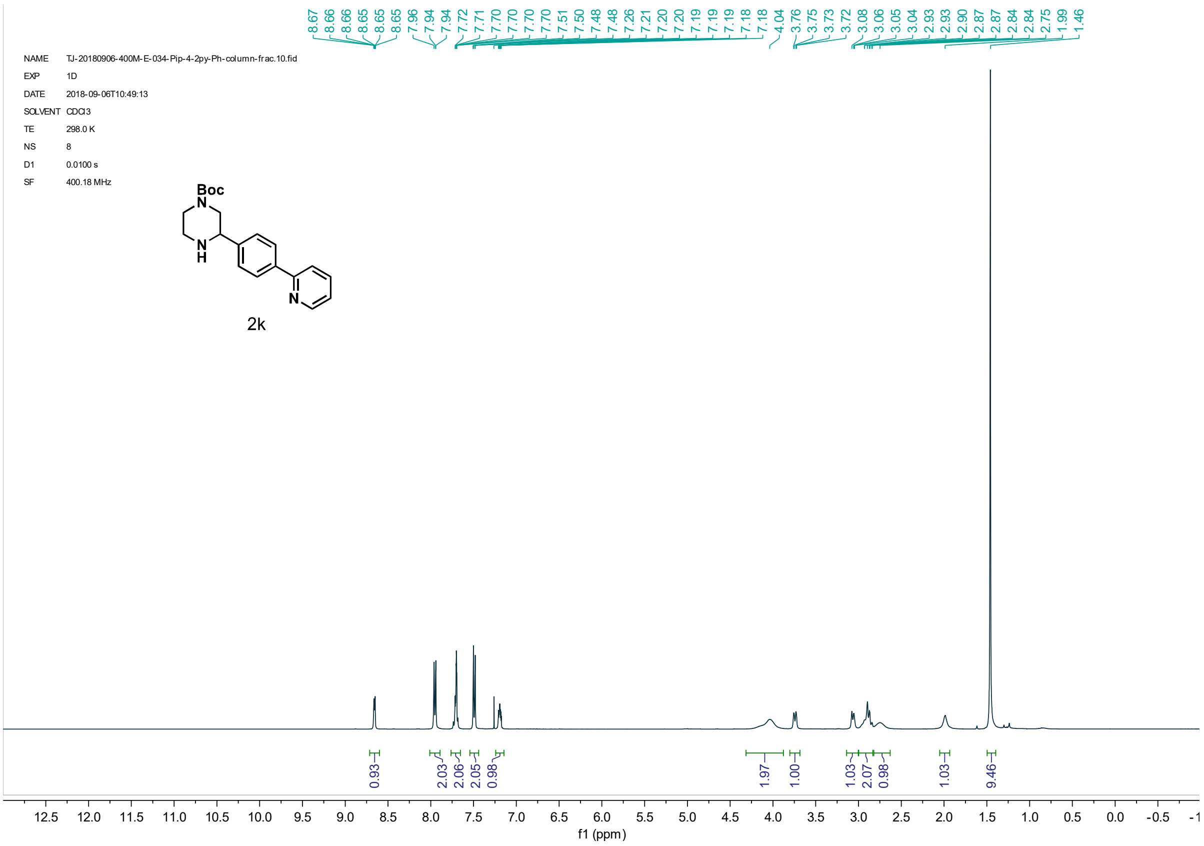

^{13}C NMR of the purified product **2k**:

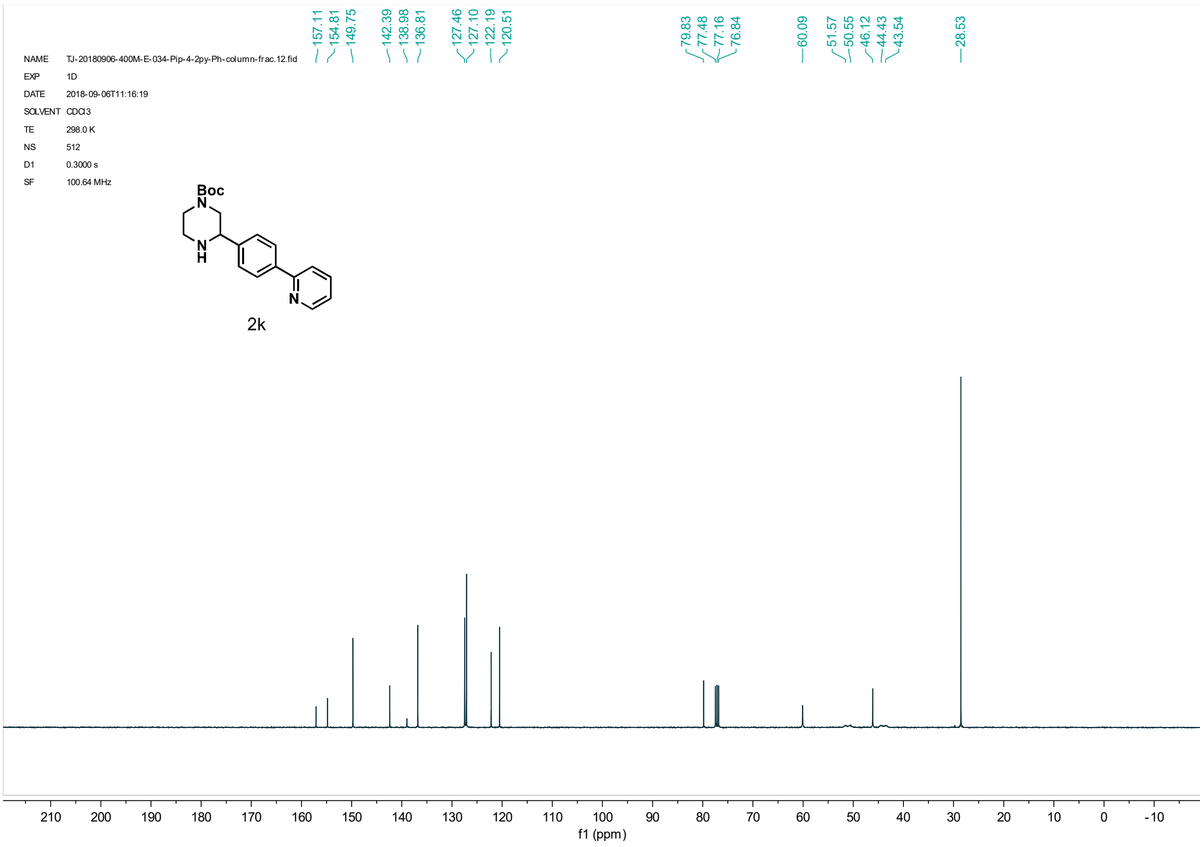

^{1}H NMR of the product **2l** obtained from Synple Chem synthesizer without purification:

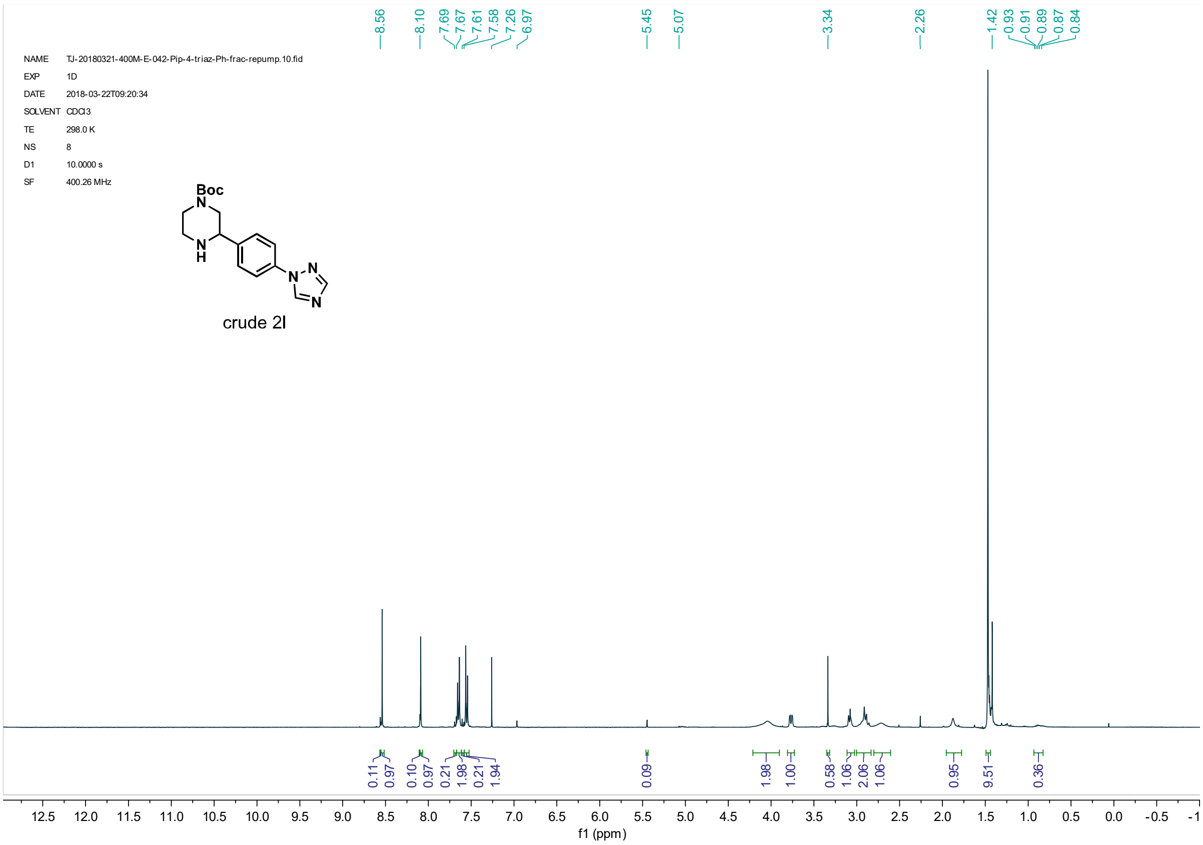

Internal standard: - .Volatile impurity: - . Other impurity: Me-acetal (δ 8.56, 8.10, 7.69–7.67, 7.61–7.58, 5.45, 3.34), BHT (δ 6.97, 5.07, 2.26, 1.42), "Sn" (δ 0.93–0.84).

^{1}H NMR of the purified product **2l**:

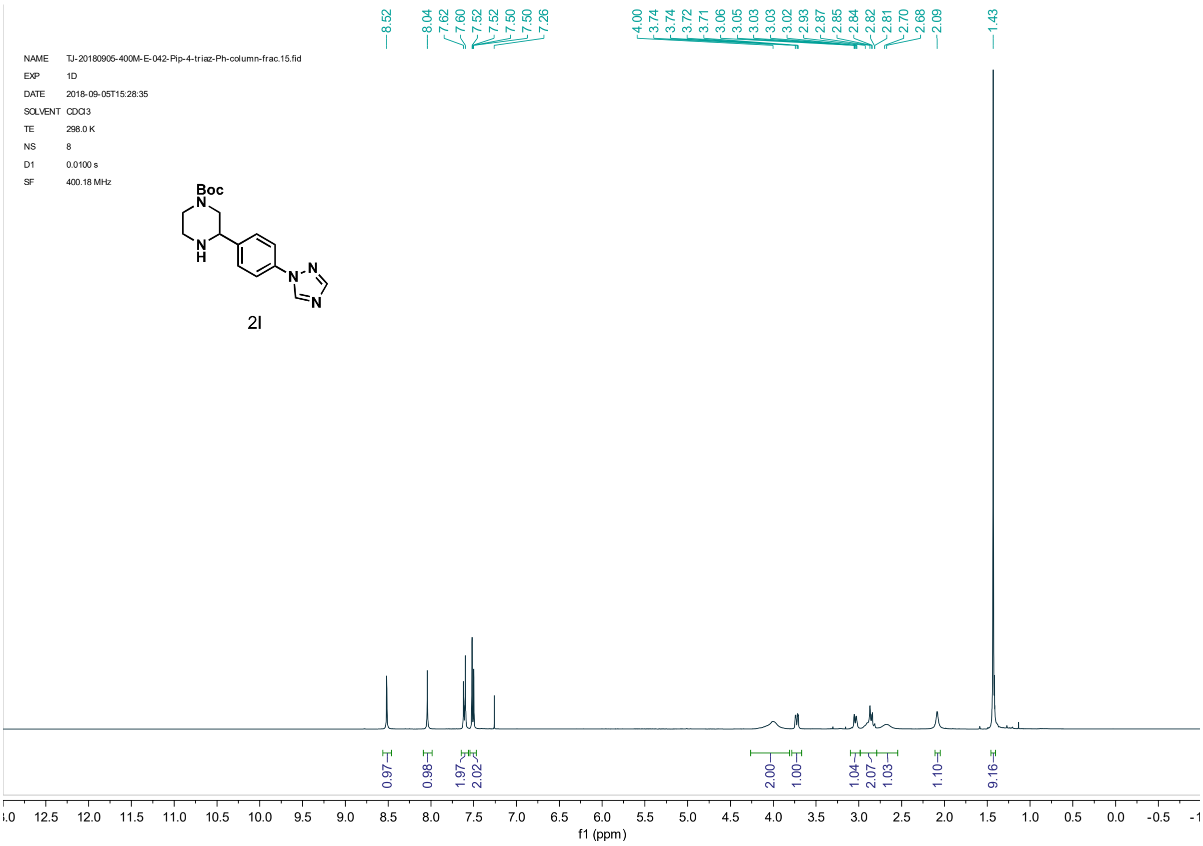

^{13}C NMR of the purified product **2l**:

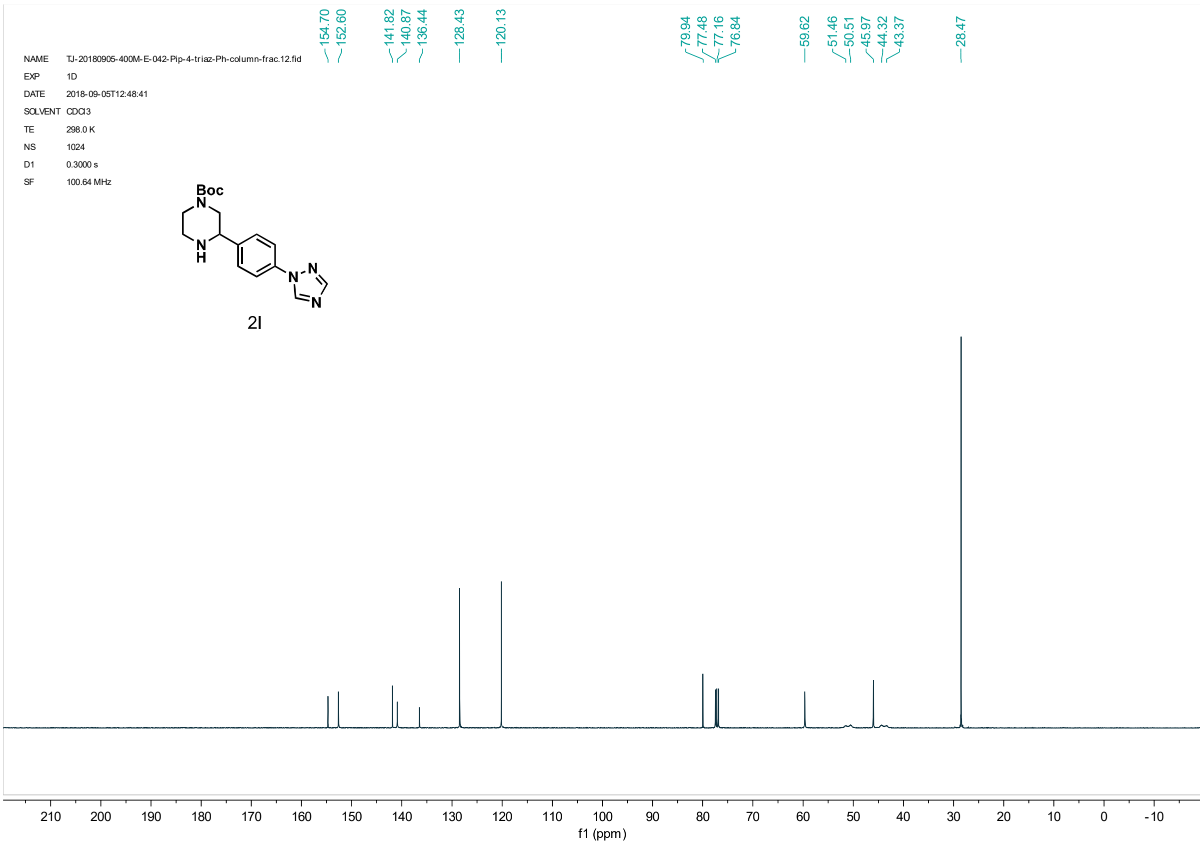

^{1}H NMR of the product **3a** obtained from Synple Chem synthesizer without purification:

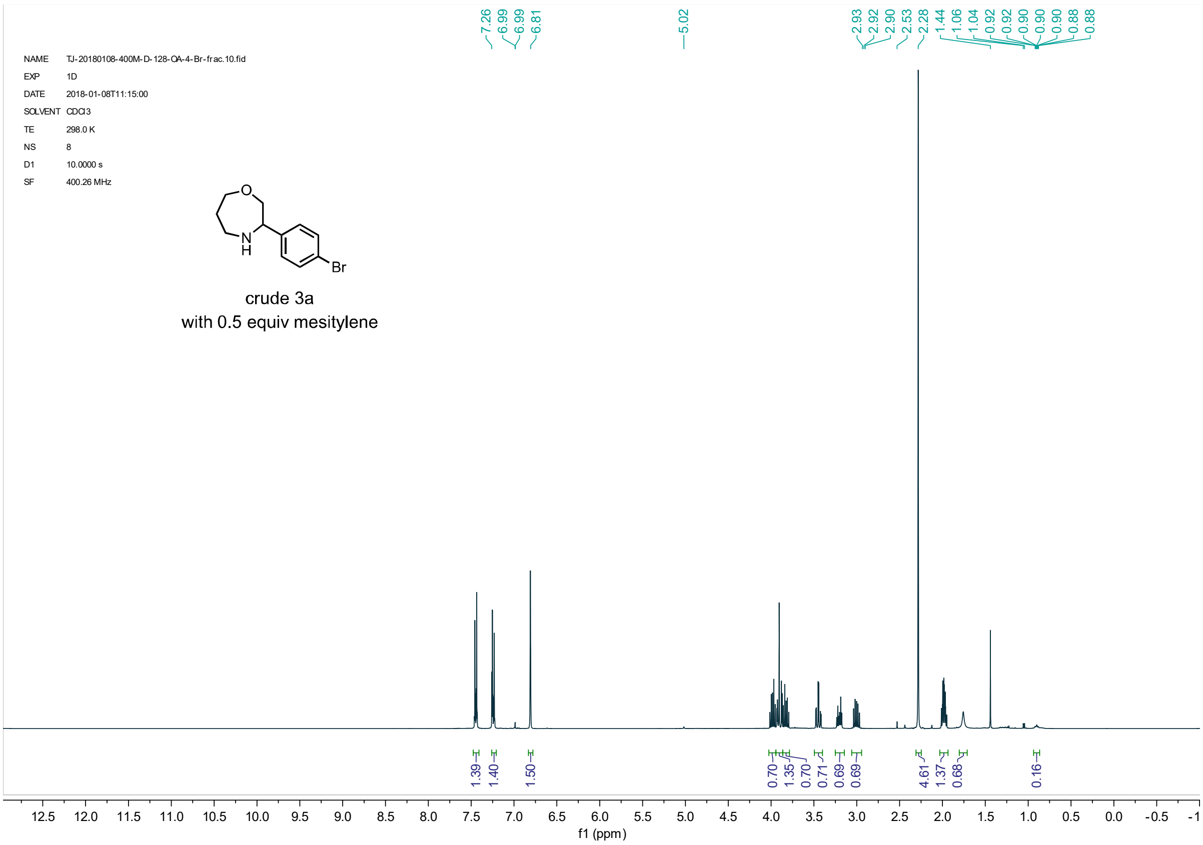

Internal standard: 0.5 equiv mesitylene (*δ* 6.81, 2.28). Volatile impurity: - .Other impurity: BHT (*δ* 6.99, 5.02, 2.53, 1.44), "Sn" (*δ* 0.92–0.88).

^{1}H NMR of the purified product **3a**:

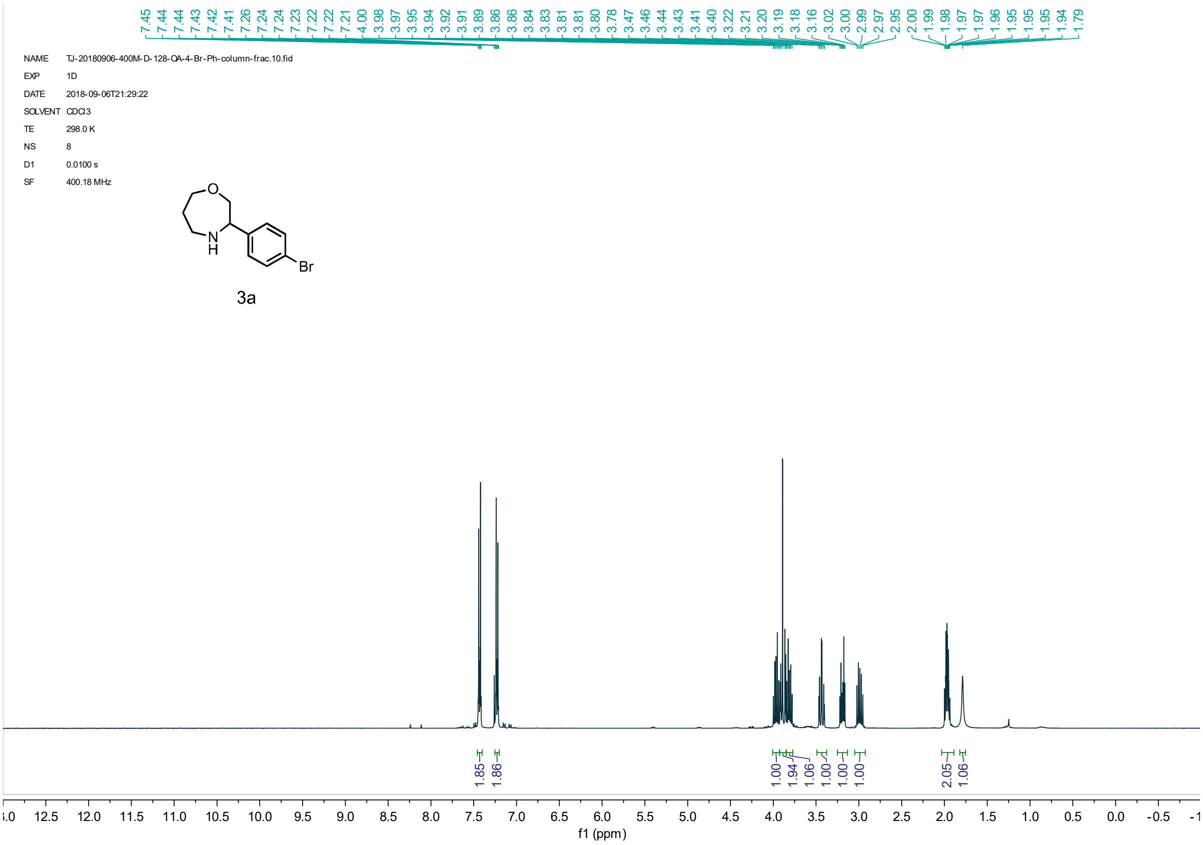

^{13}C NMR of the purified product **3a**:

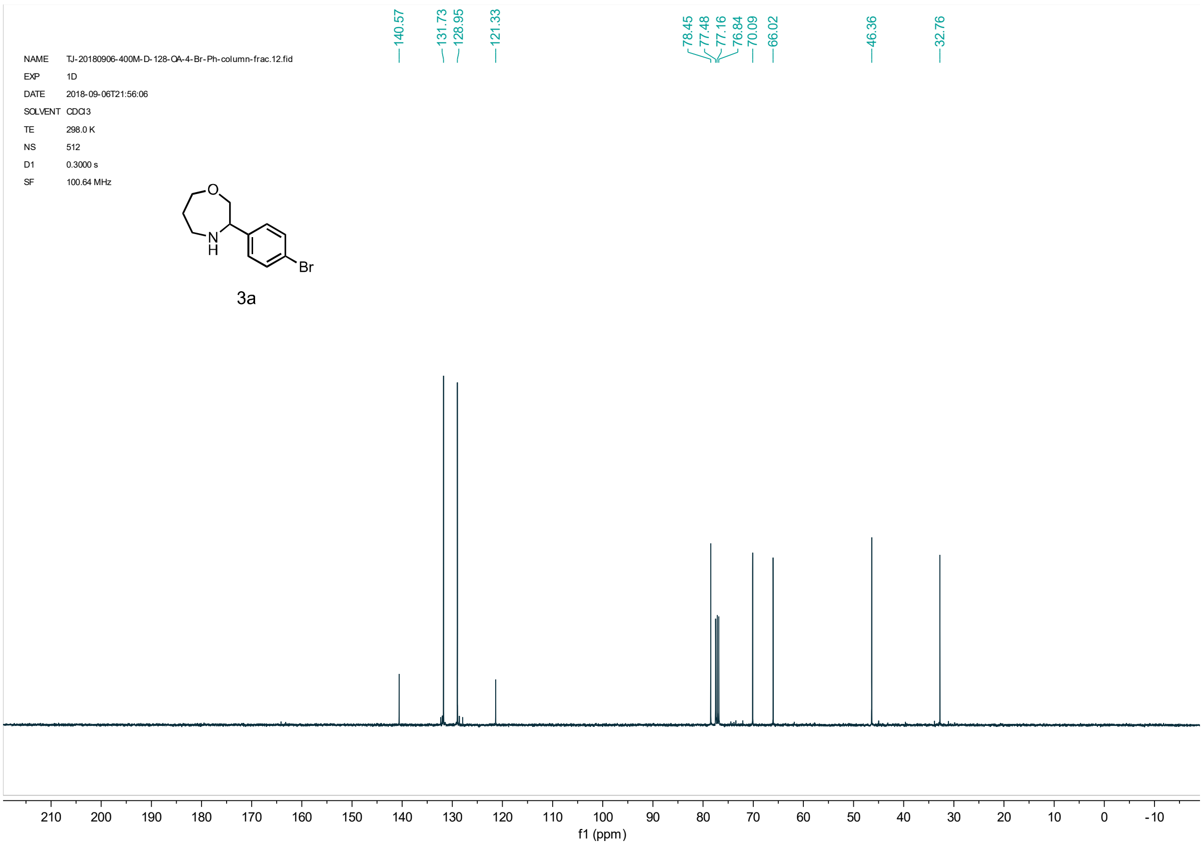

^{1}H NMR of the product **3b** obtained from Synple Chem synthesizer without purification:

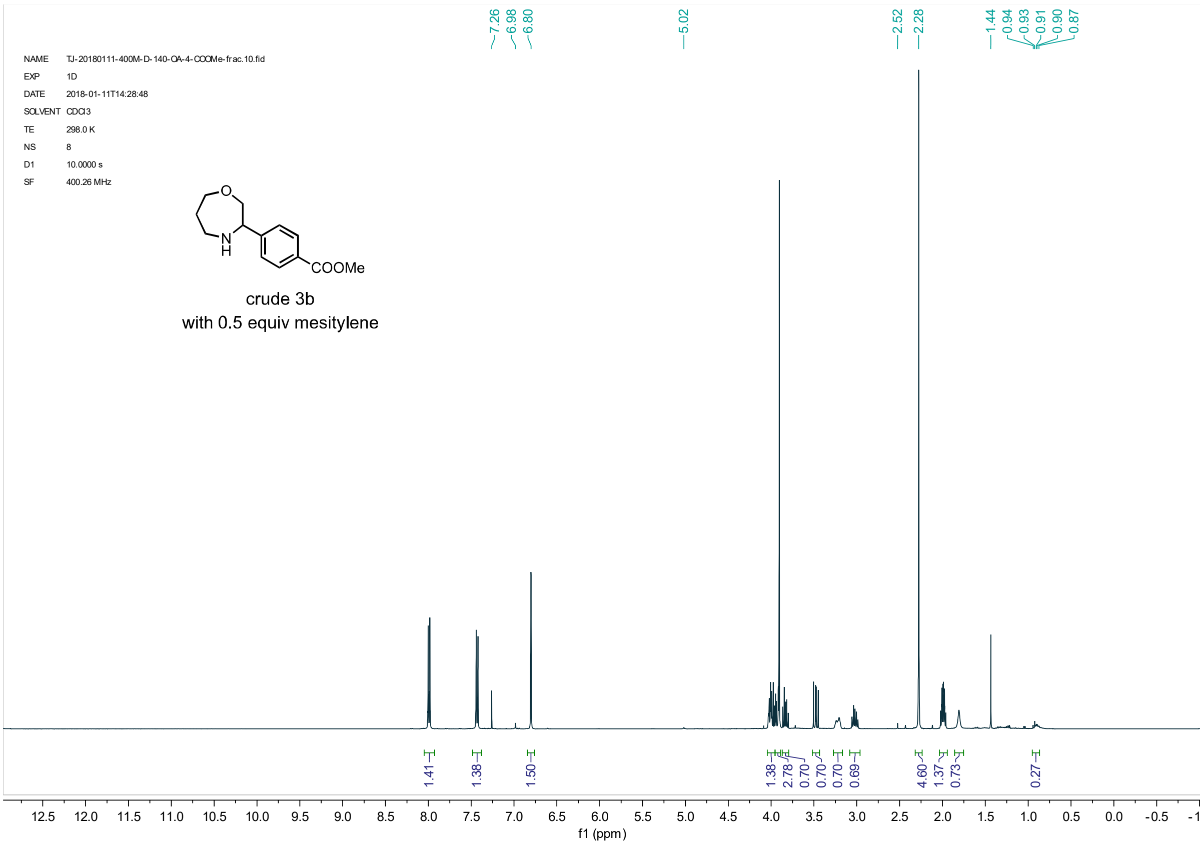

Internal standard: 0.5 equiv mesitylene (*δ* 6.80, 2.28). Volatile impurity: - .Other impurity: BHT (*δ* 6.98, 5.02, 2.52, 1.44), "Sn" (*δ* 0.94–0.87).

^{1}H NMR of the purified product **3b**:

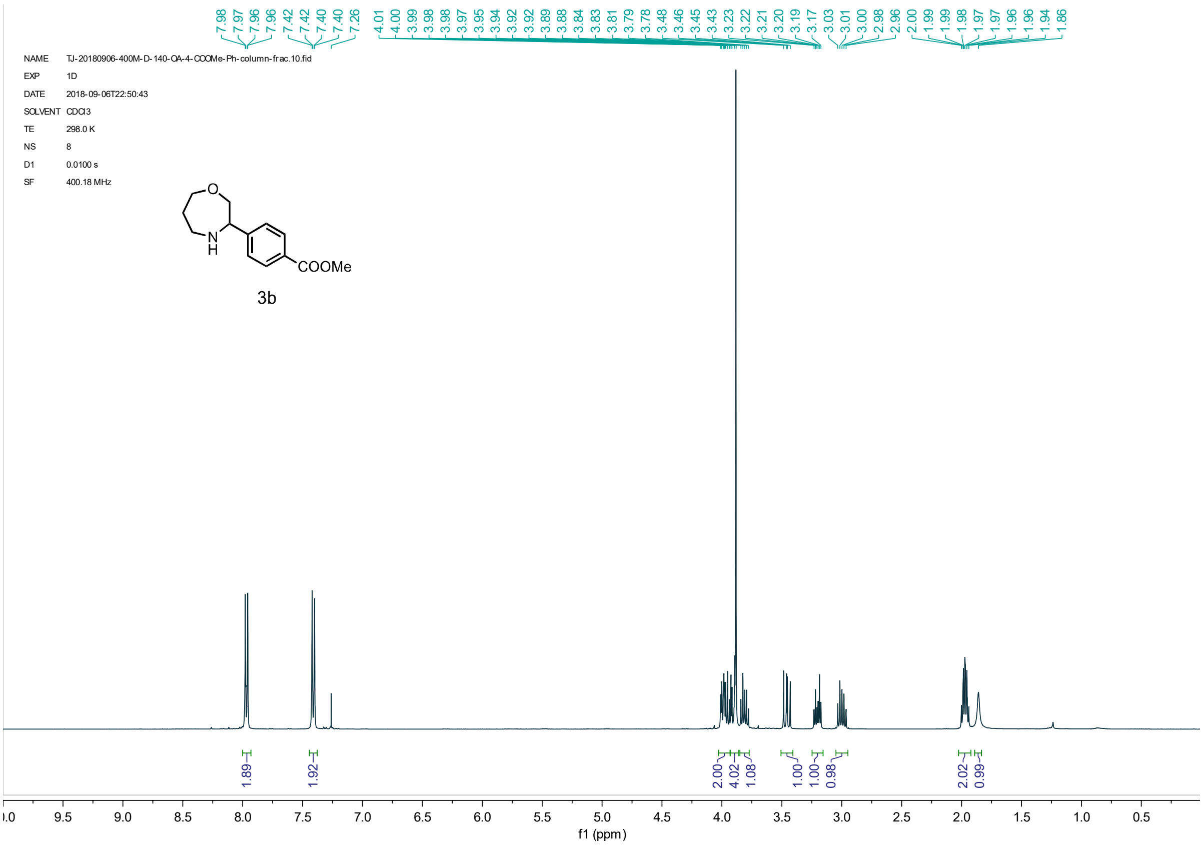

^{13}C NMR of the purified product **3b**:

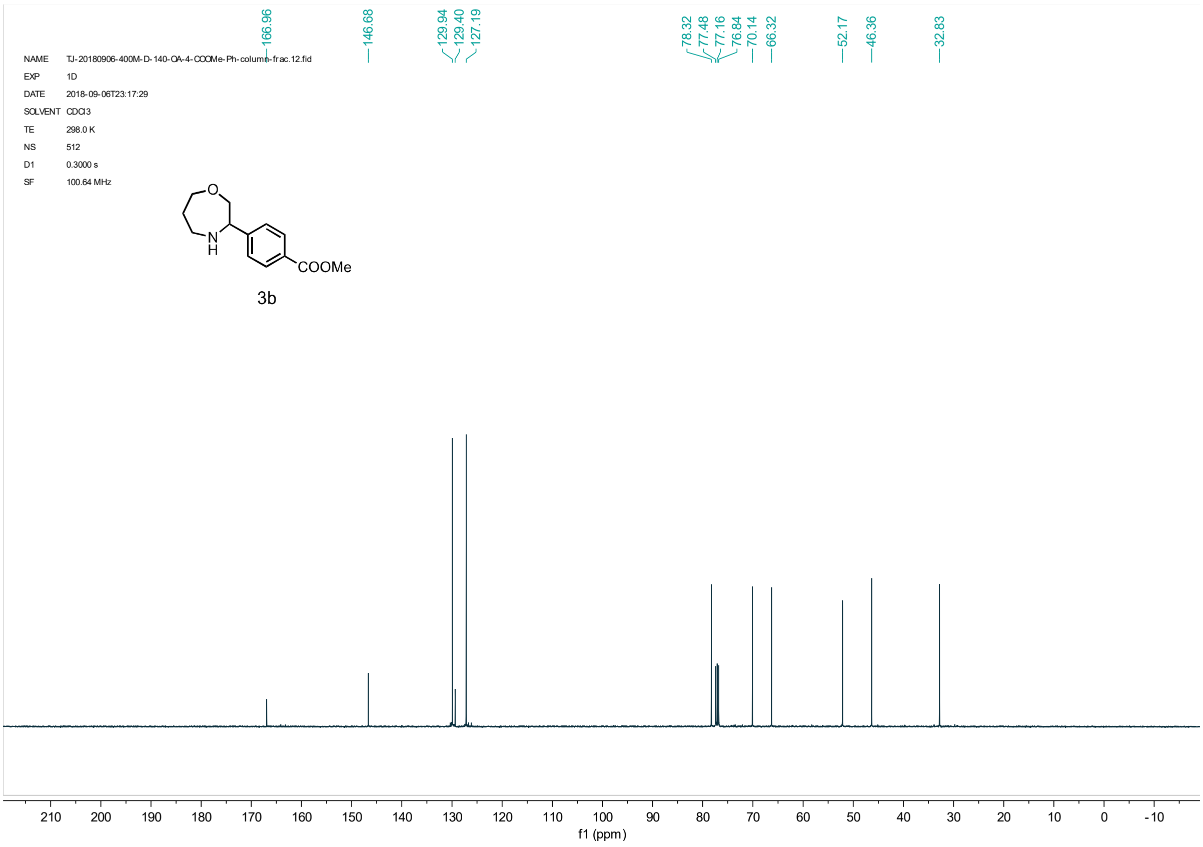

^{1}H NMR of the product **3c** obtained from Synple Chem synthesizer without purification:

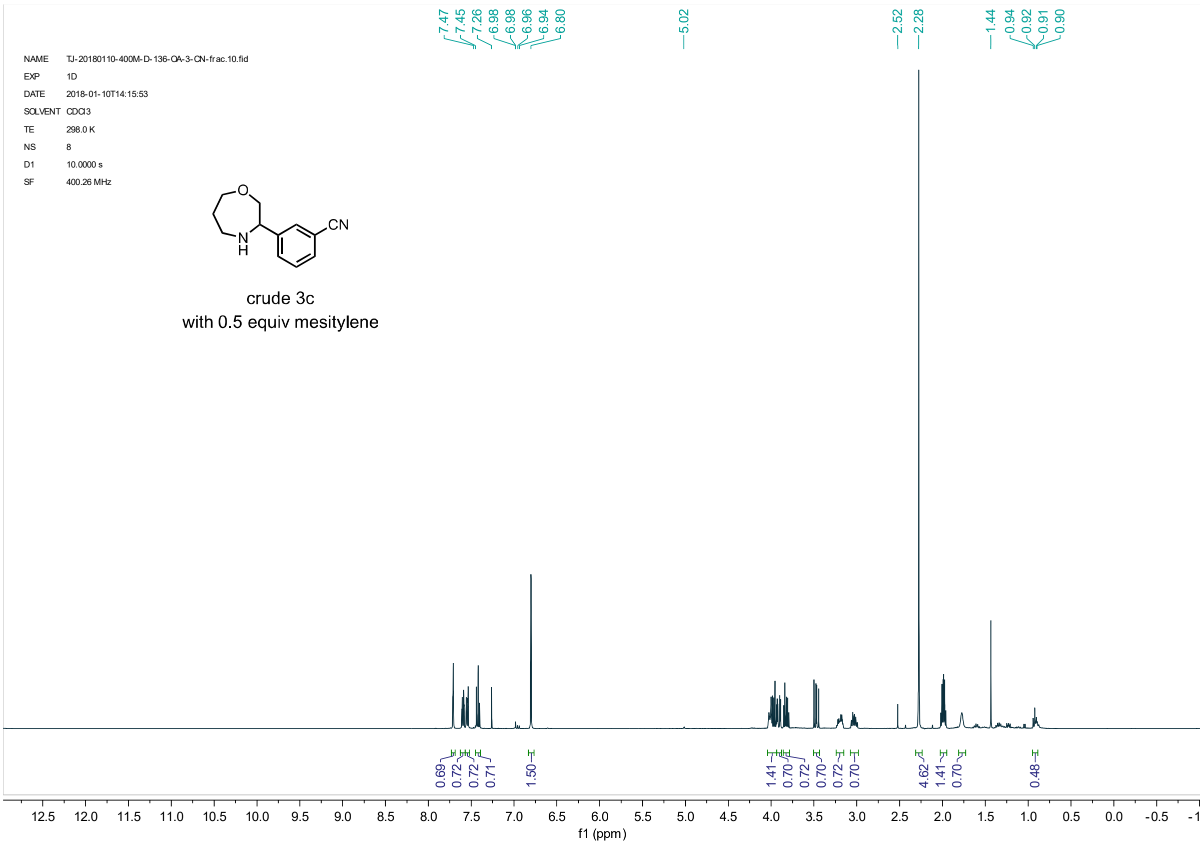

Internal standard: 0.5 equiv mesitylene (δ 6.80, 2.28). Volatile impurity: 2,6-lutidine (δ 7.47–7.45, 6.96–6.94, 2.52). Other impurity: BHT (δ 6.98, 5.02, 2.52, 1.44), “Sn” (δ 0.94–0.90).

^{1}H NMR of the purified product **3c**:

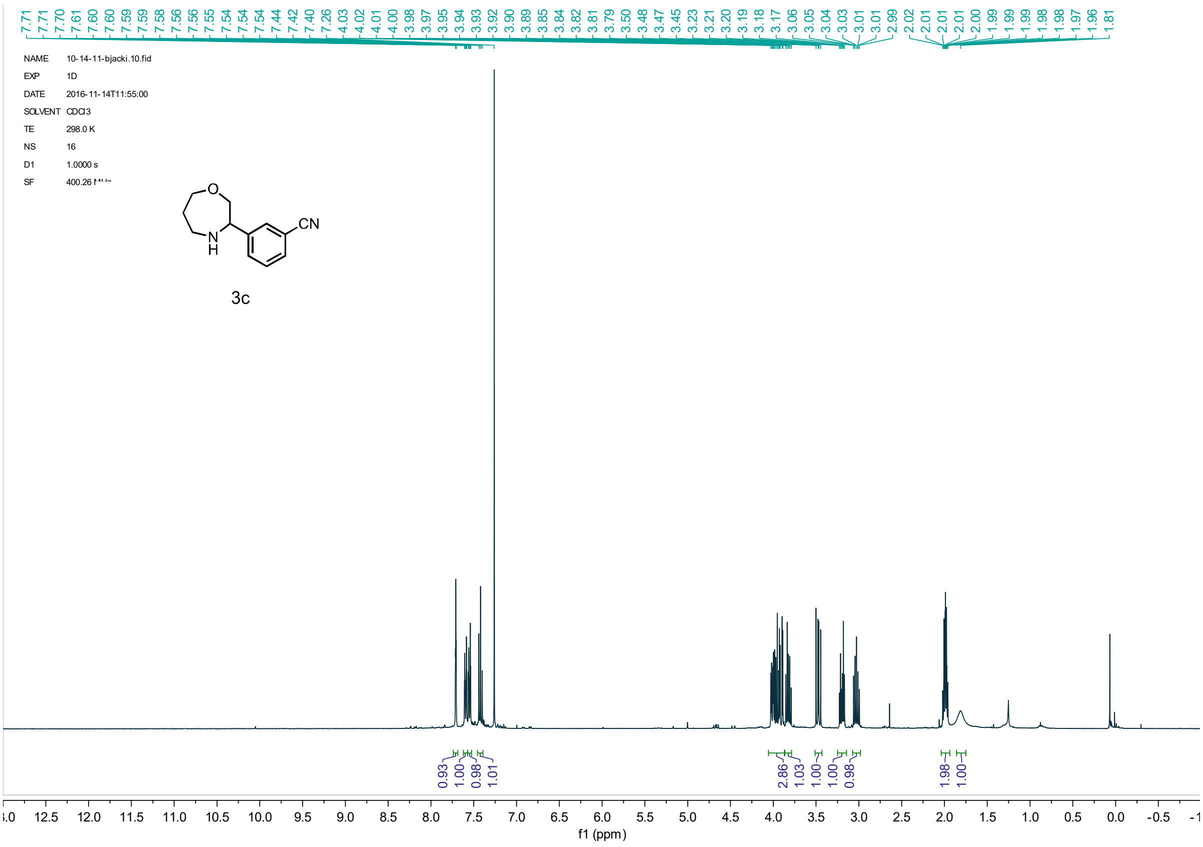

^{1}H NMR of the product **3d** obtained from Synple Chem synthesizer without purification:

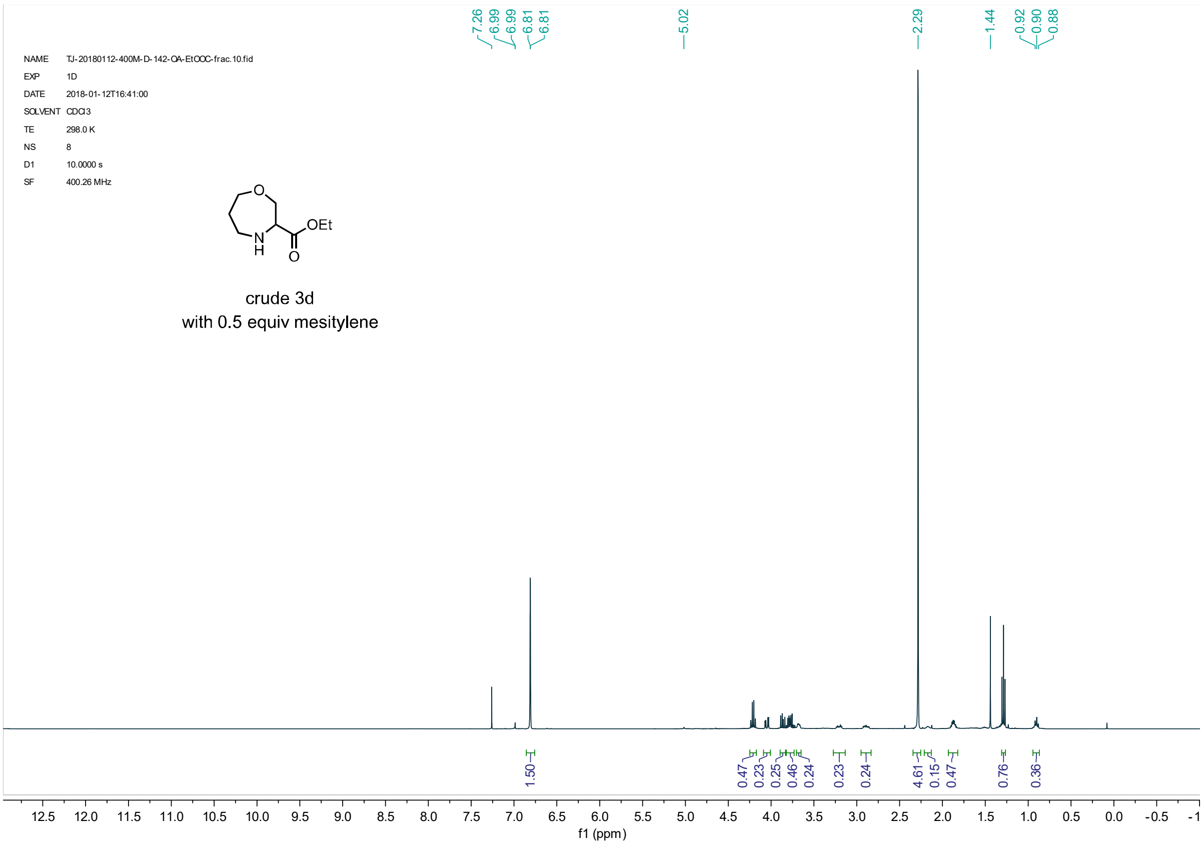

Internal standard: 0.5 equiv mesitylene (δ 6.81, 2.29). Volatile impurity: - . Other impurity: BHT (δ 6.99, 5.02, 1.44), "Sn" (δ 0.90–0.88).

^{1}H NMR of the purified product **3d**:

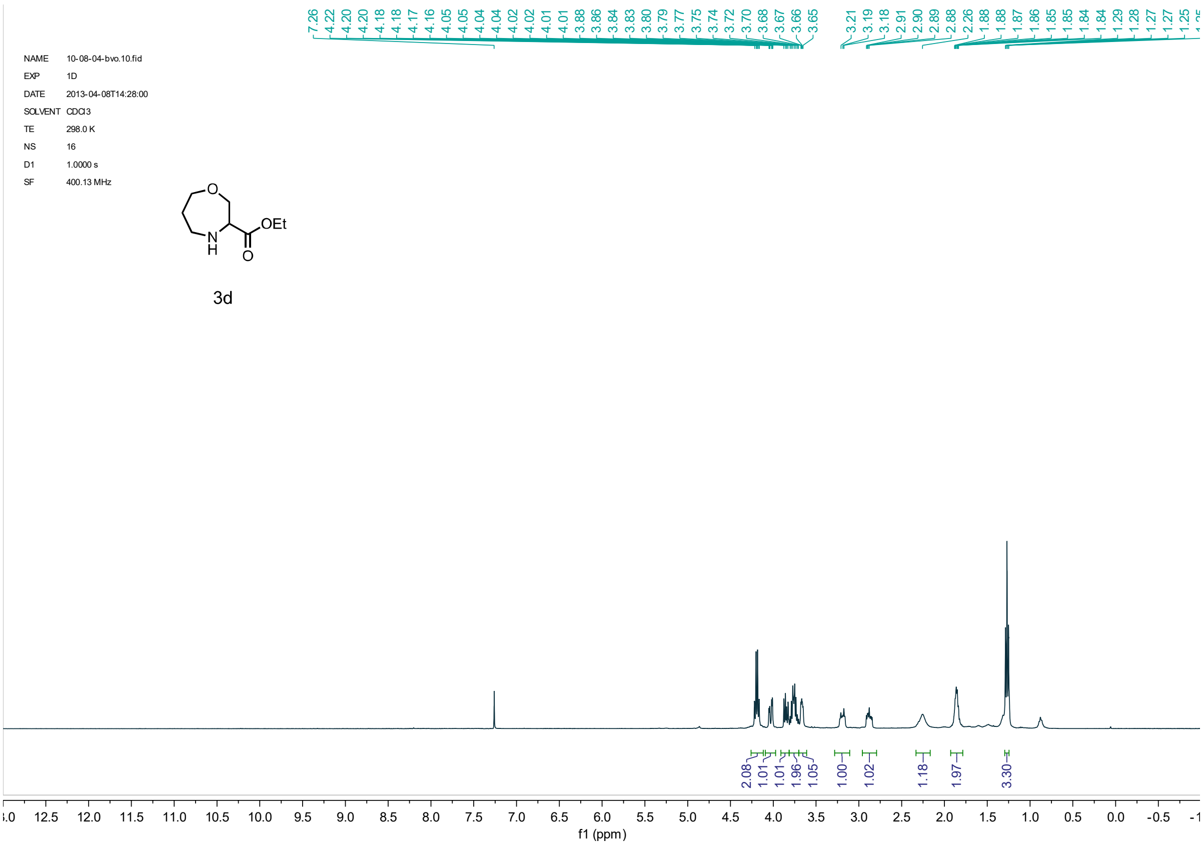

^{1}H NMR of the product **3e** obtained from Synple Chem synthesizer without purification:

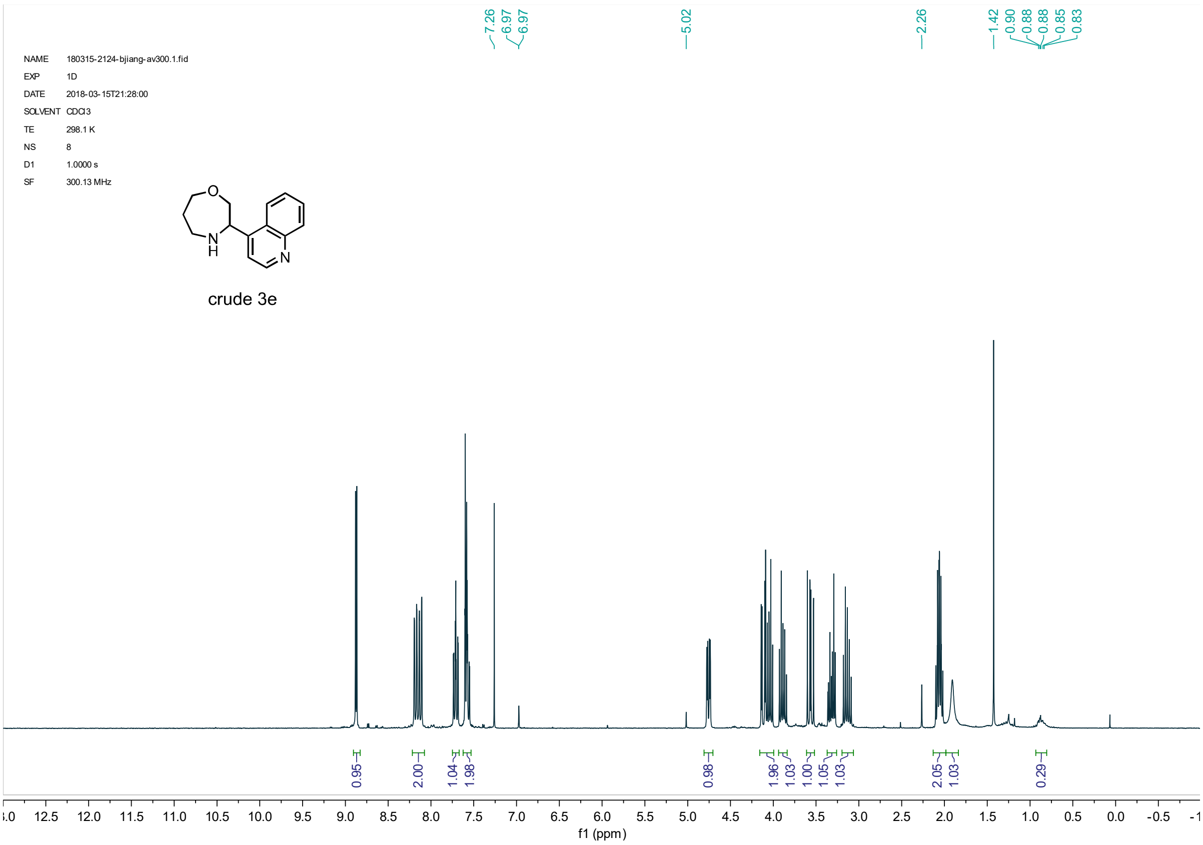

Internal standard: - . Volatile impurity: - . Other impurity: BHT (δ 6.97, 5.02, 2.26, 1.42), "Sn" (δ 0.90–0.83).

^{1}H NMR of the purified product **3e**:

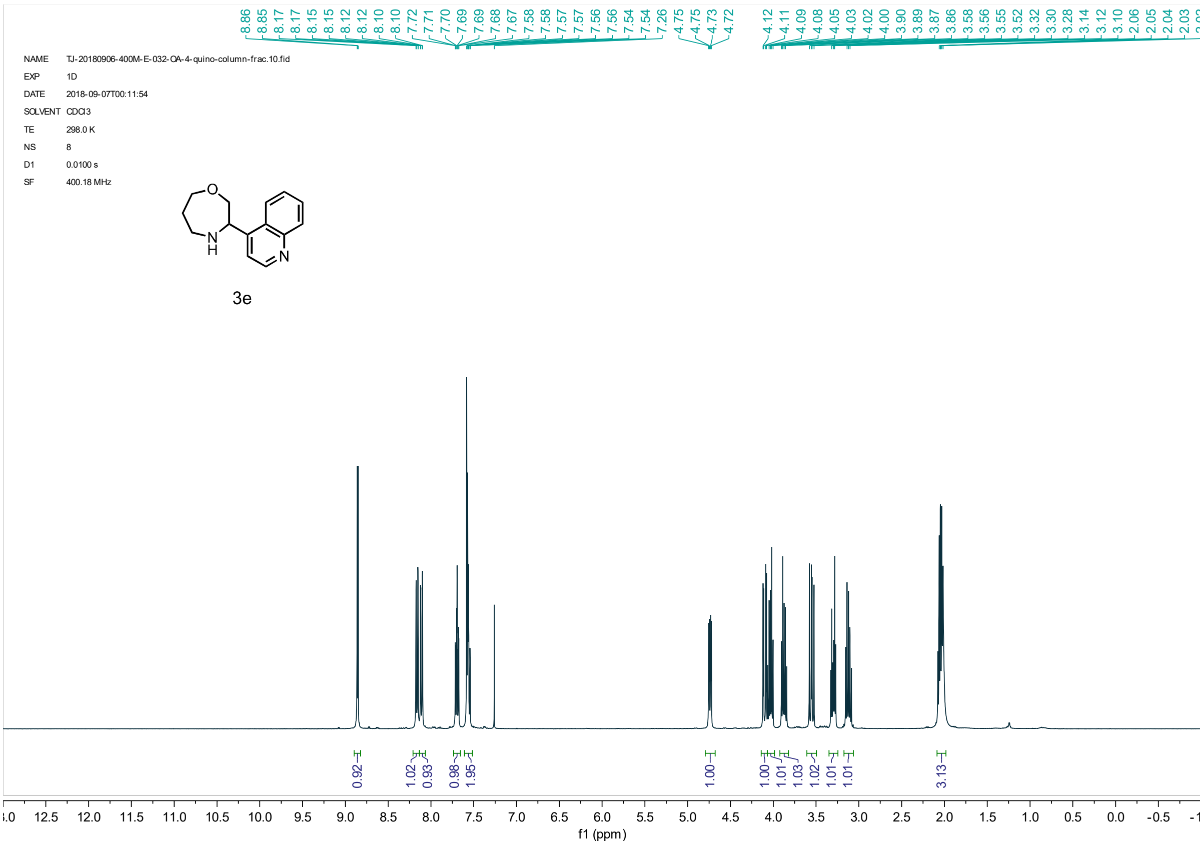

^{13}C NMR of the purified product **3e**:

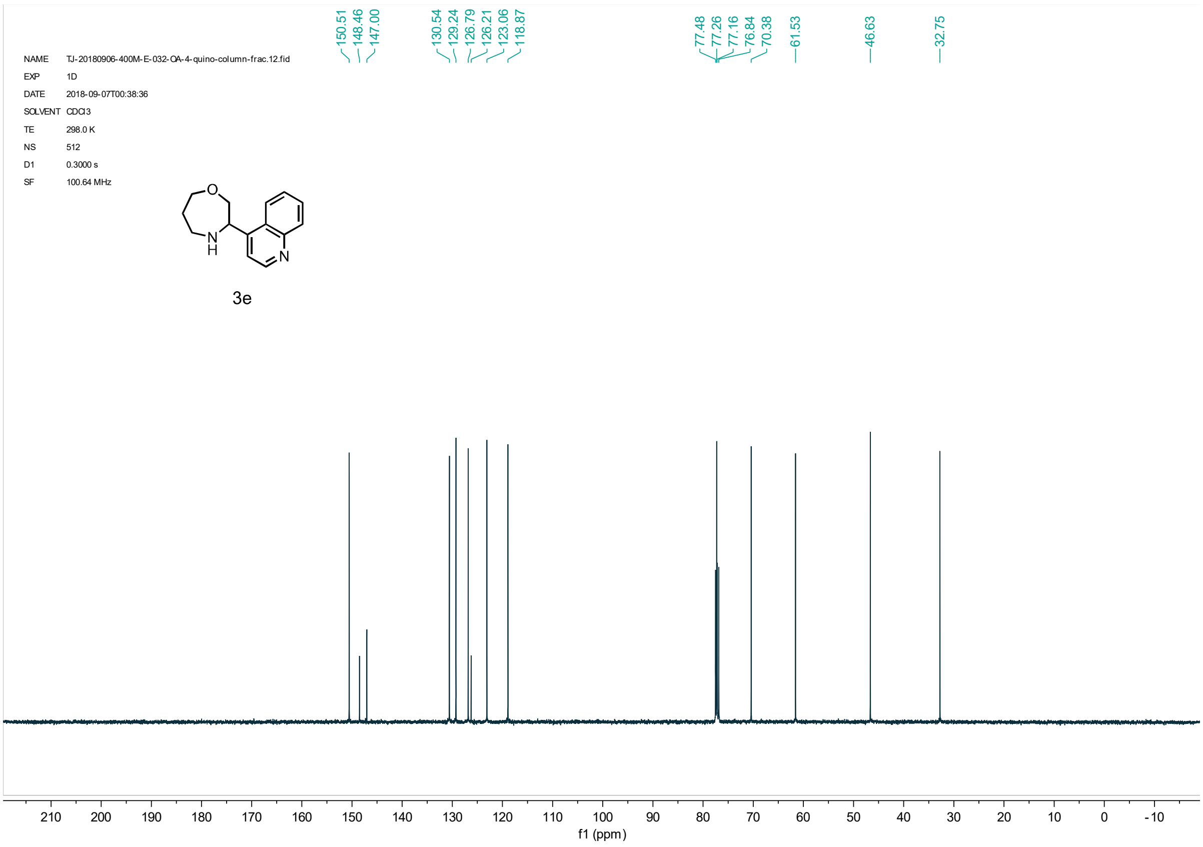

^{1}H NMR of the product **4a** obtained from Synple Chem synthesizer without purification:

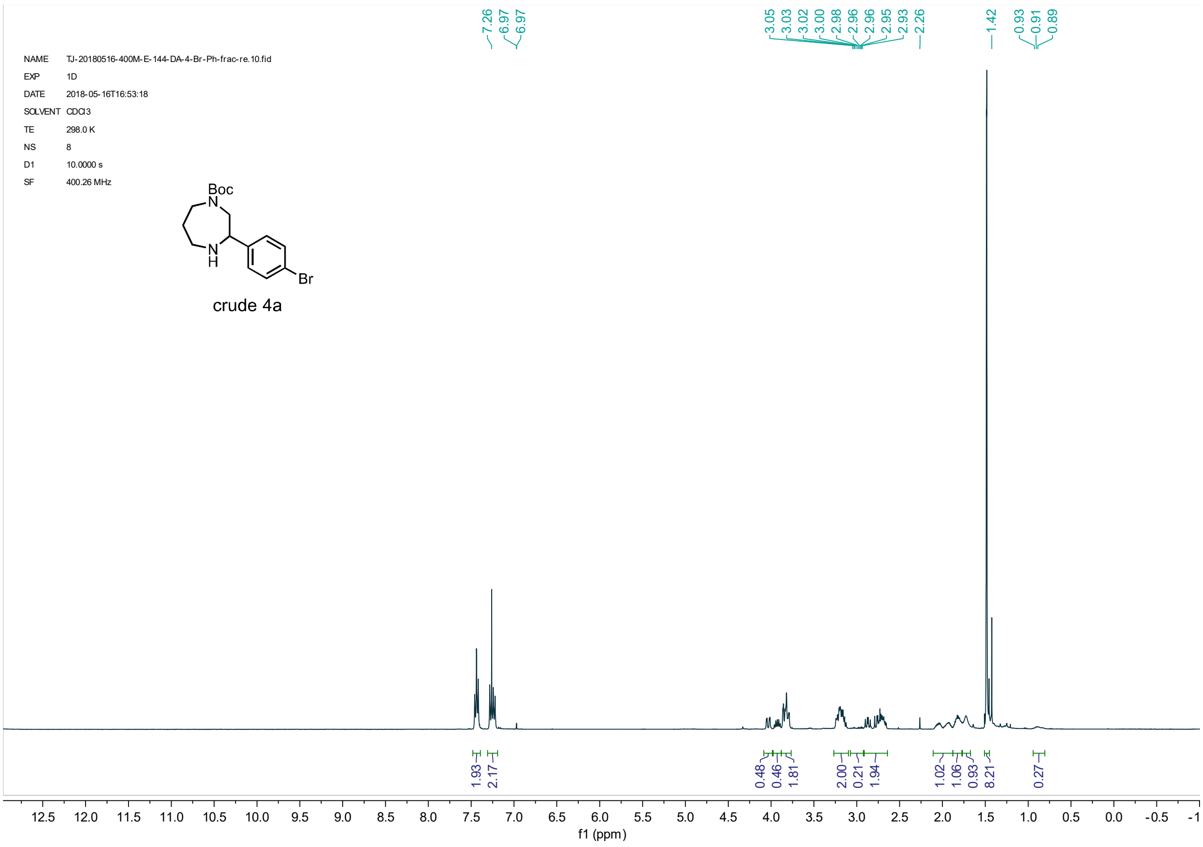

Internal standard: - . Volatile impurity: - . Other impurity: de-Boc product (δ 3.05–2.93), BHT (δ 6.97, 2.26, 1.42), “Sn” (δ 0.93–0.89).

^{1}H NMR of the purified product **4a**:

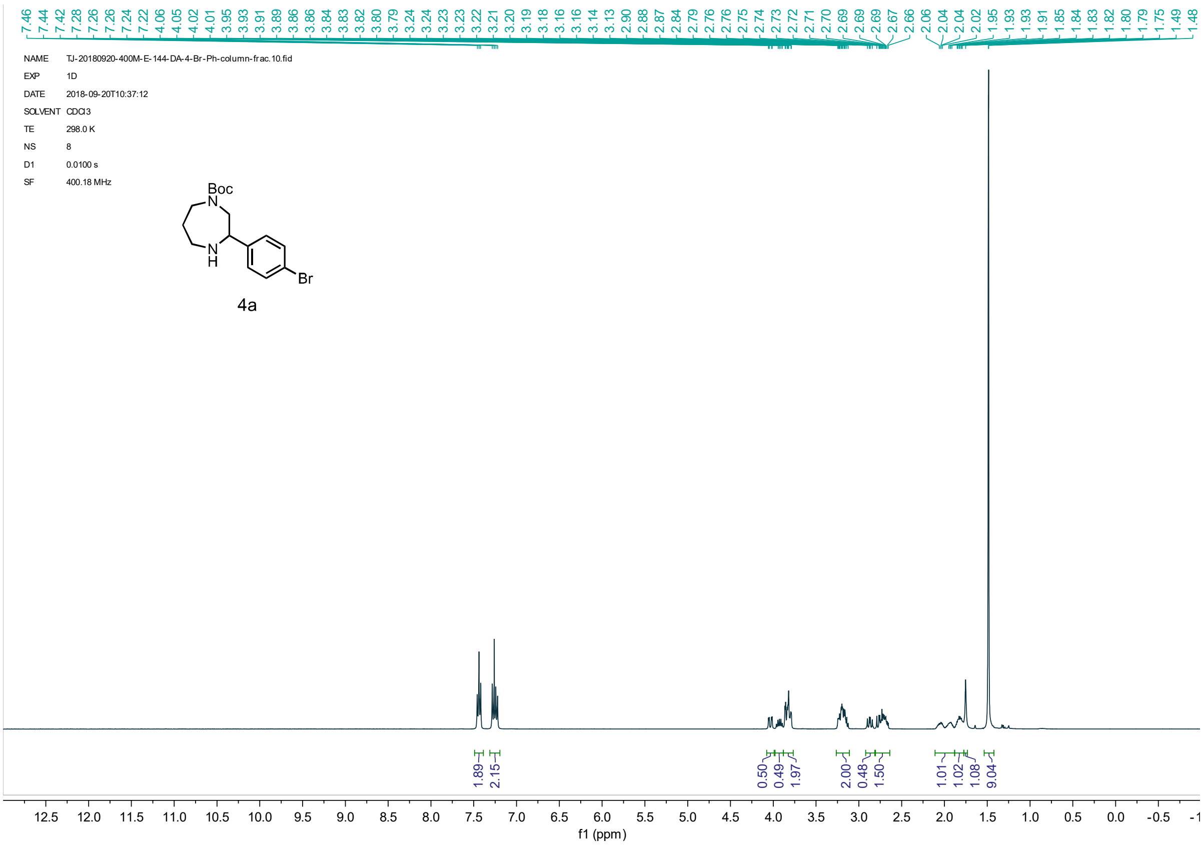

^{13}C NMR of the purified product **4a**:

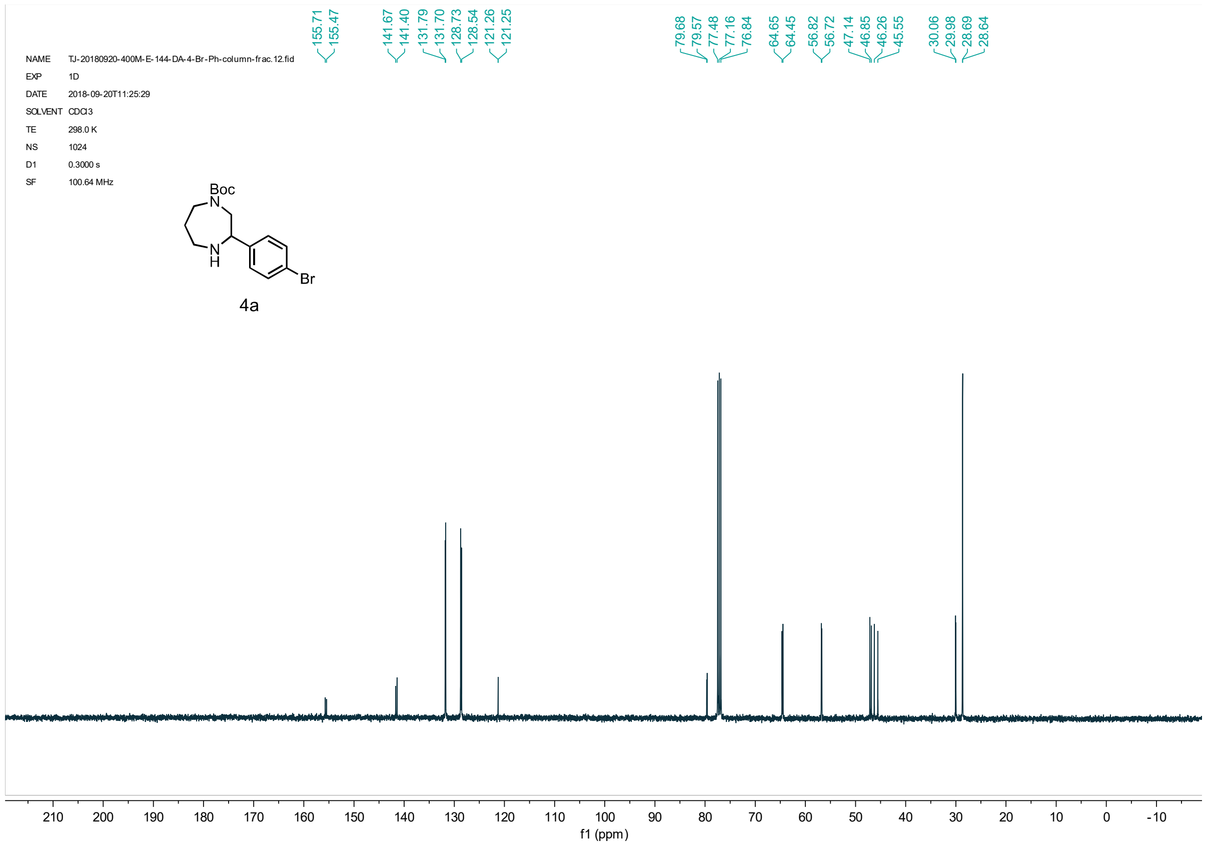

^{1}H NMR of the product **4b** obtained from Synple Chem synthesizer without purification:

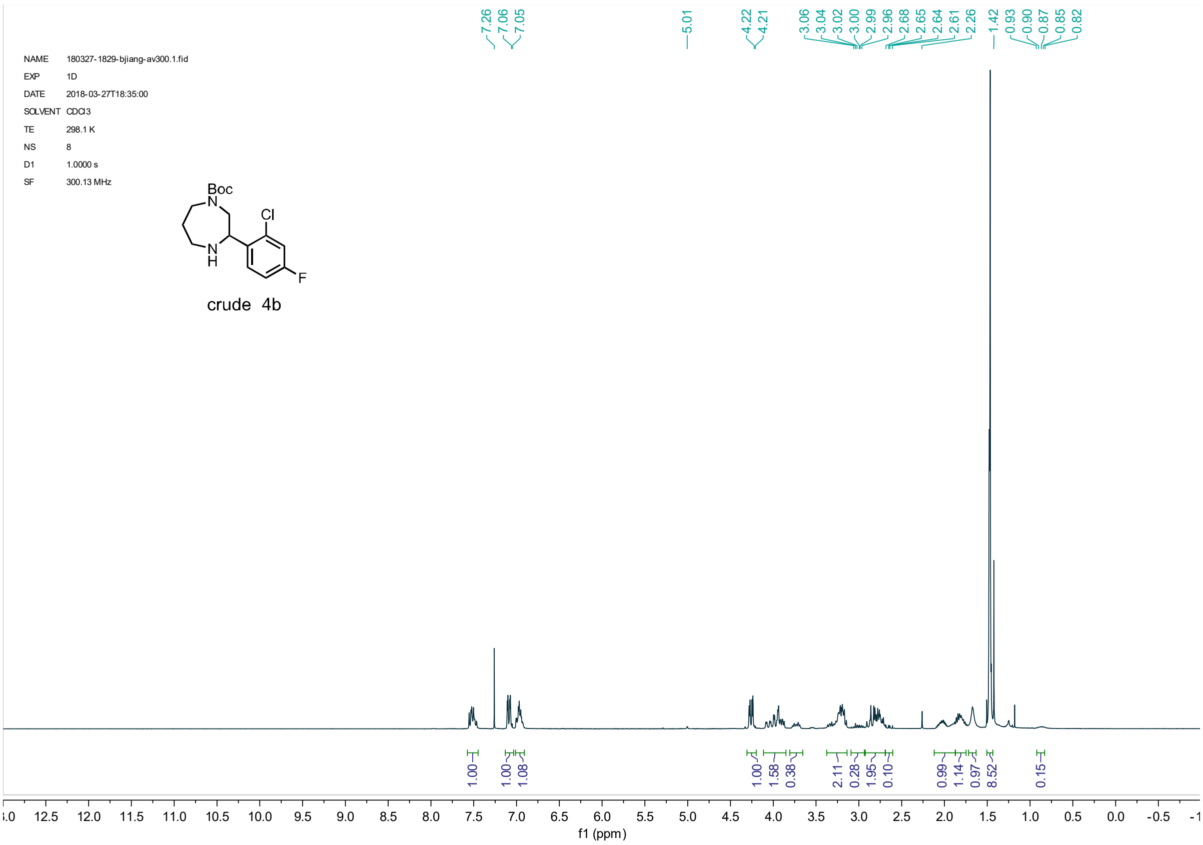

Internal standard: - . Volatile impurity: - . Other impurity: de-Boc product (δ 7.06, 4.22–4.21, 3.06–2.96, 2.68–2.61), BHT (δ 5.01, 2.26, 1.42), "Sn" (δ 0.93–0.82).

^{1}H NMR of the purified product **4b**:

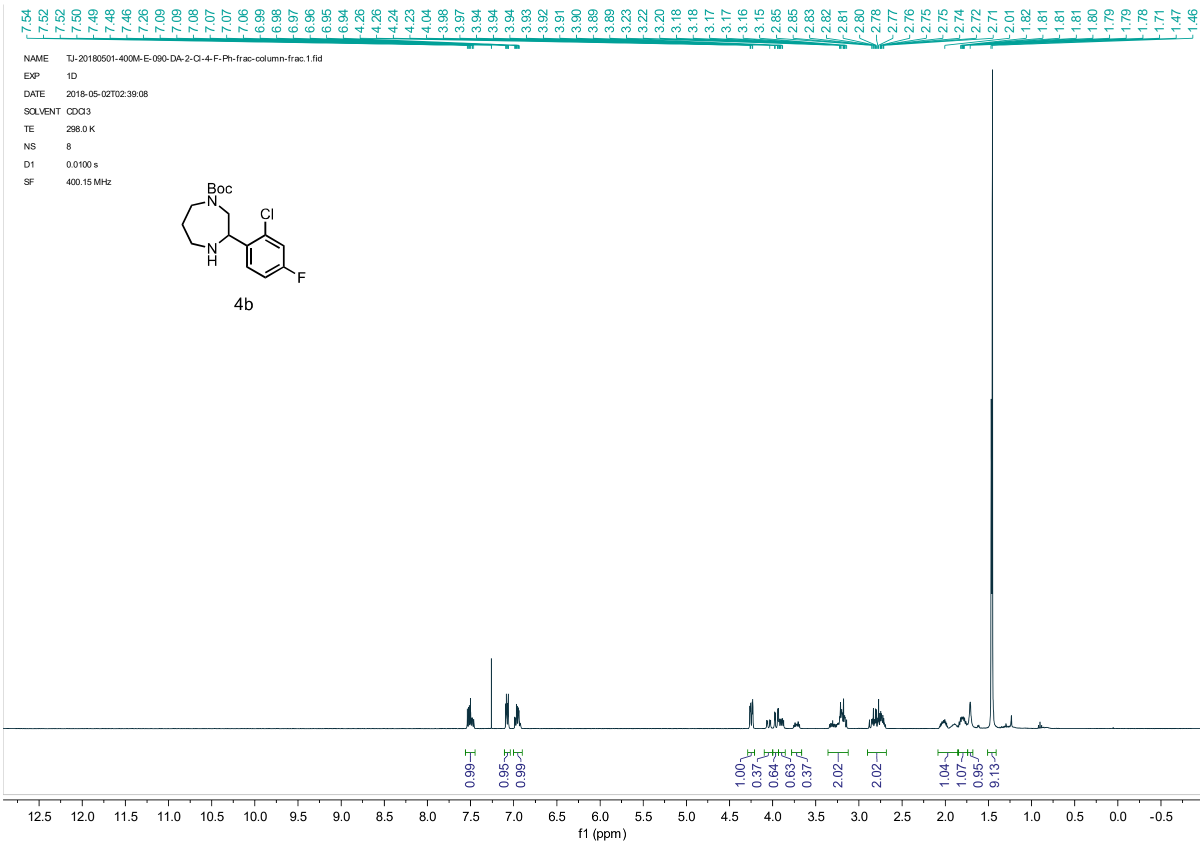

^{13}C NMR of the purified product **4b**:

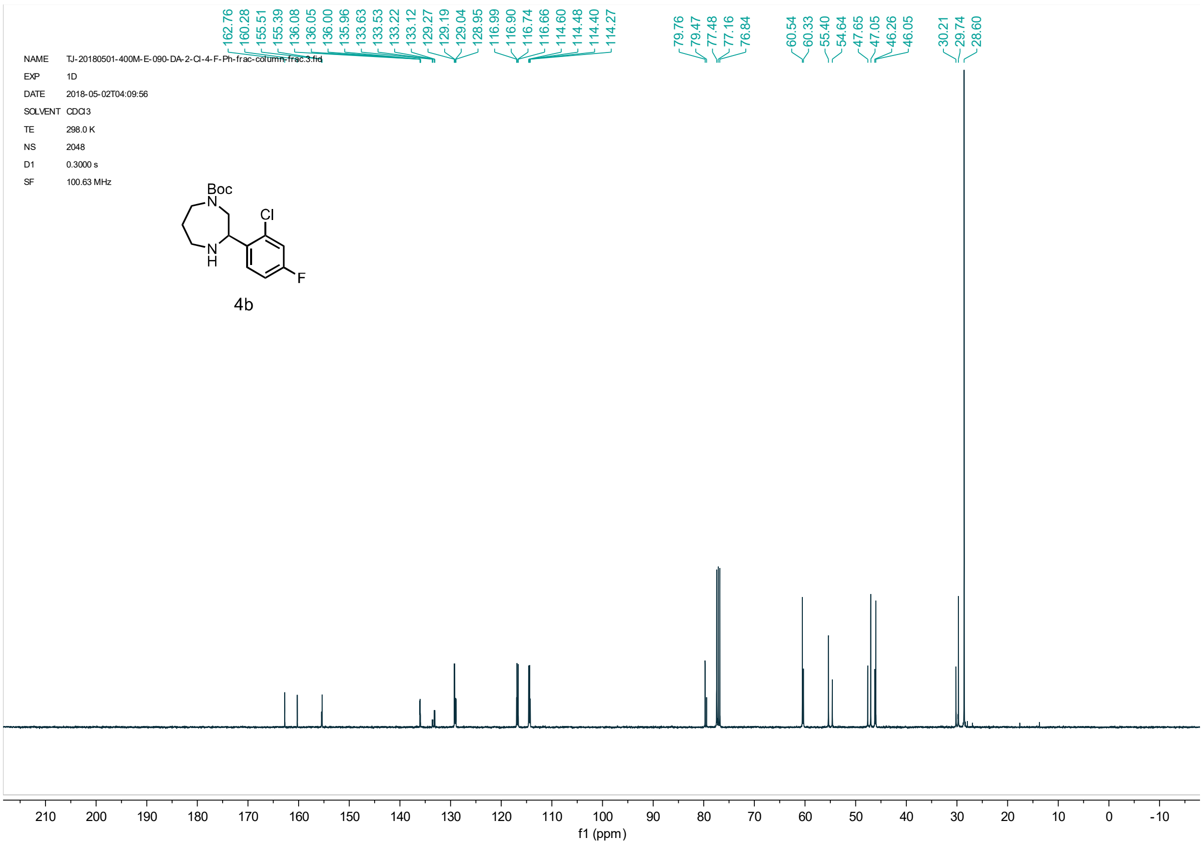

^{1}H NMR of the product **4c** obtained from Synple Chem synthesizer without purification:

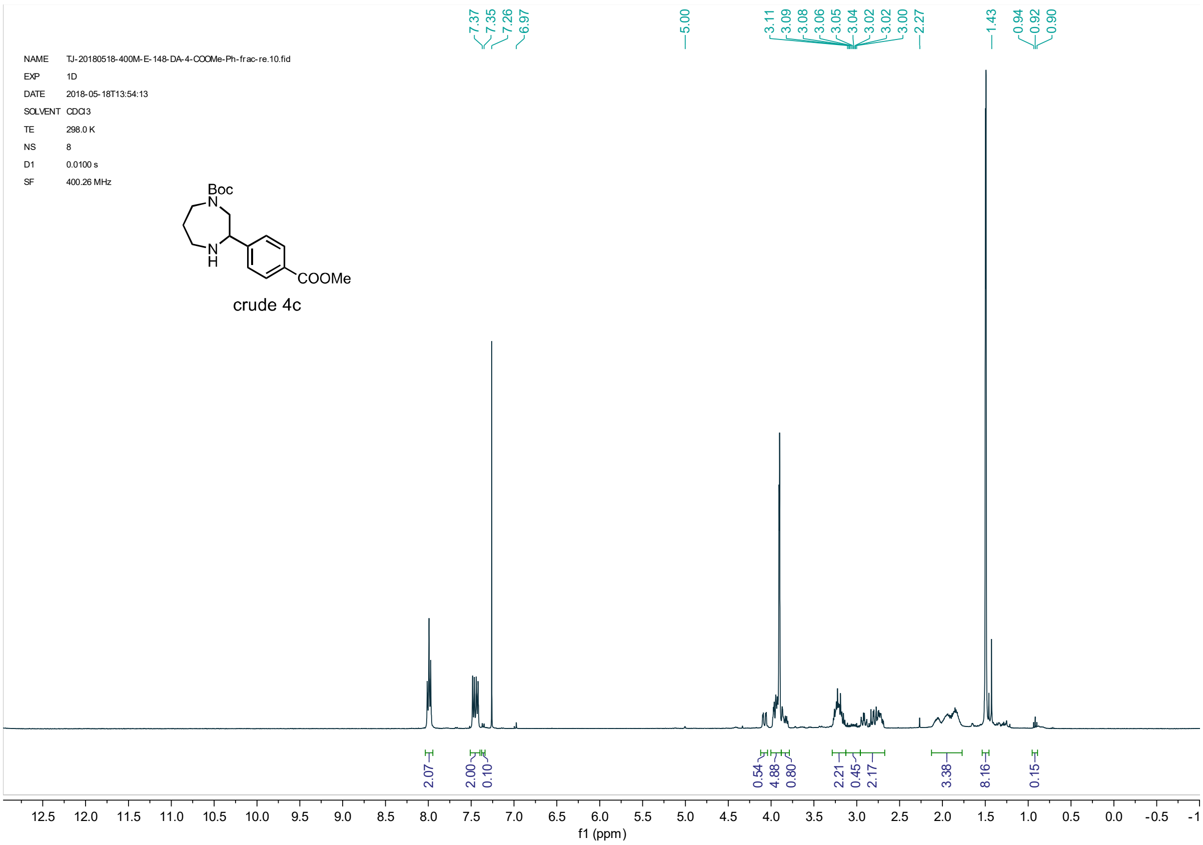

Internal standard: - .Volatile impurity: - . Other impurity: de-Boc product (δ 7.37–7.35, 3.11–3.00), BHT (δ 6.97, 5.00, 2.27, 1.43), “Sn” (δ 0.94–0.90).

^{1}H NMR of the purified product **4c**:

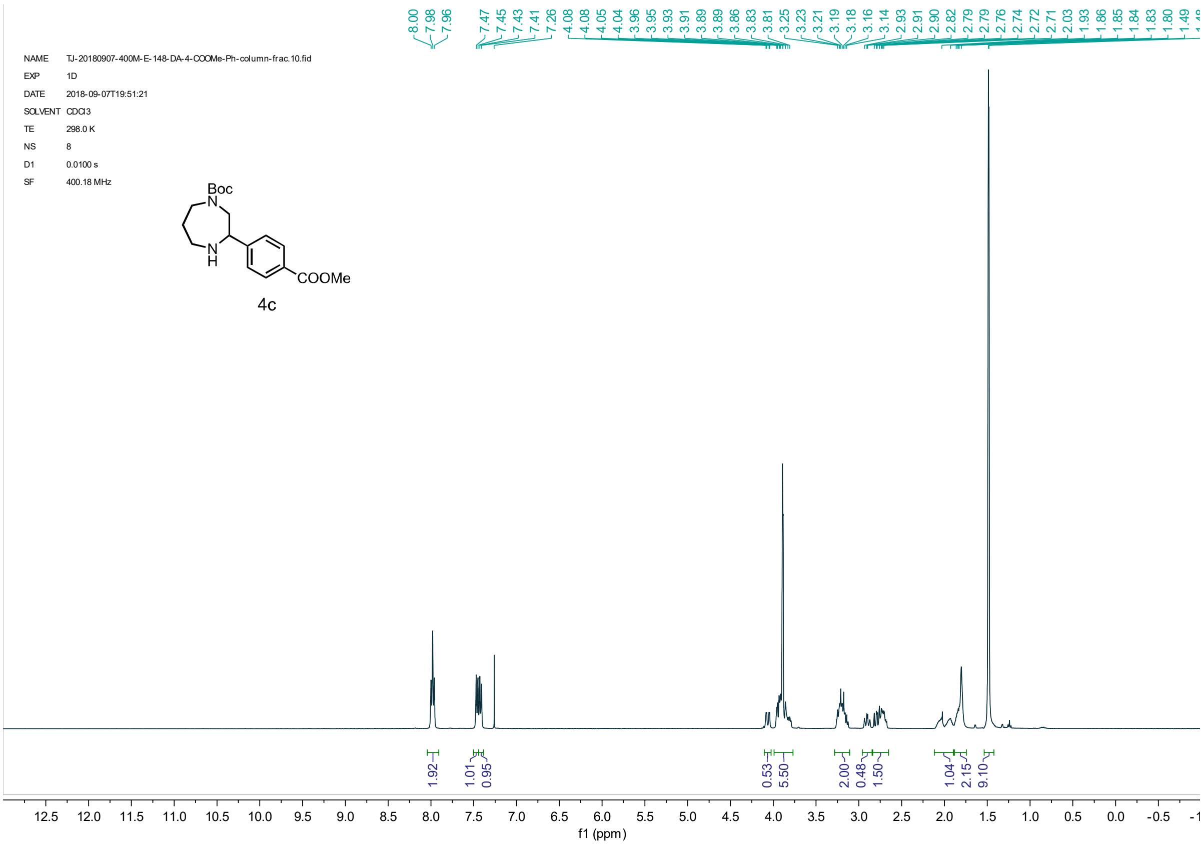

^{13}C NMR of the purified product **4c**:

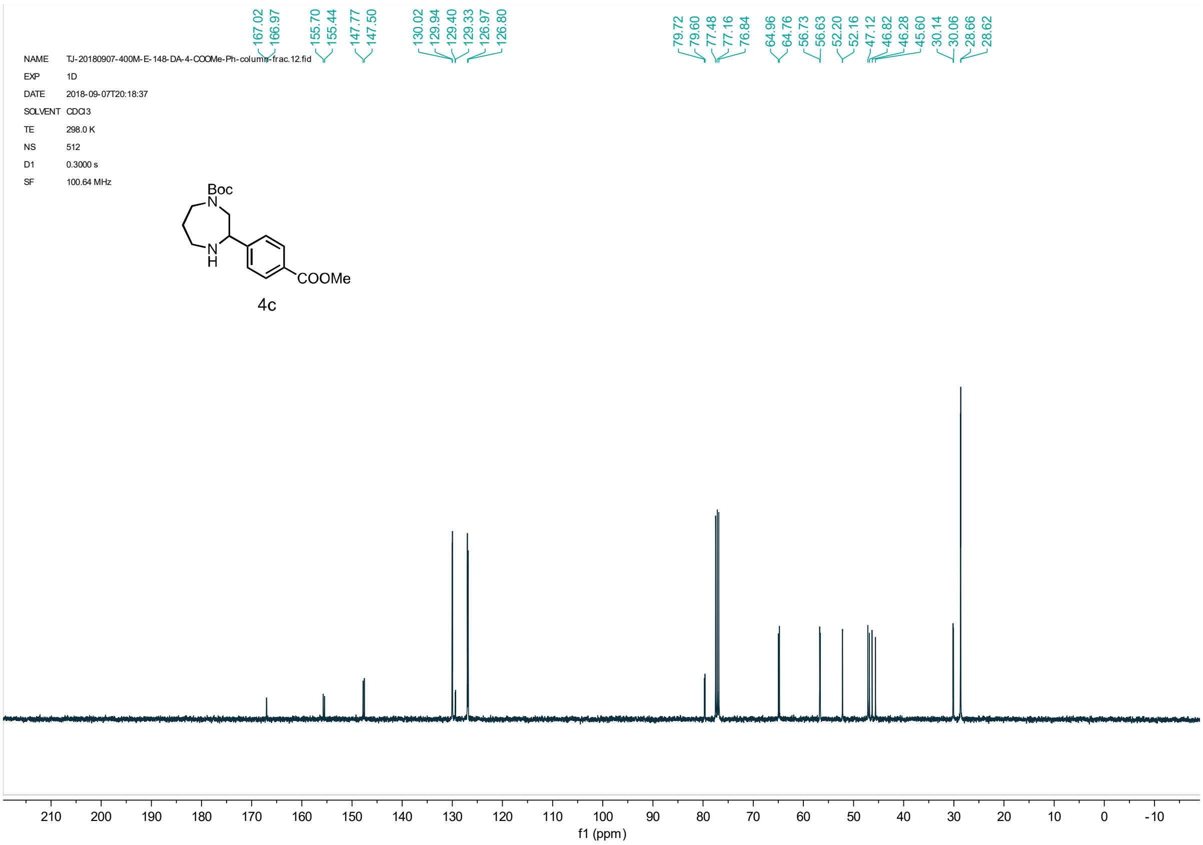

^{1}H NMR of the product **4d** obtained from Synple Chem synthesizer without purification:

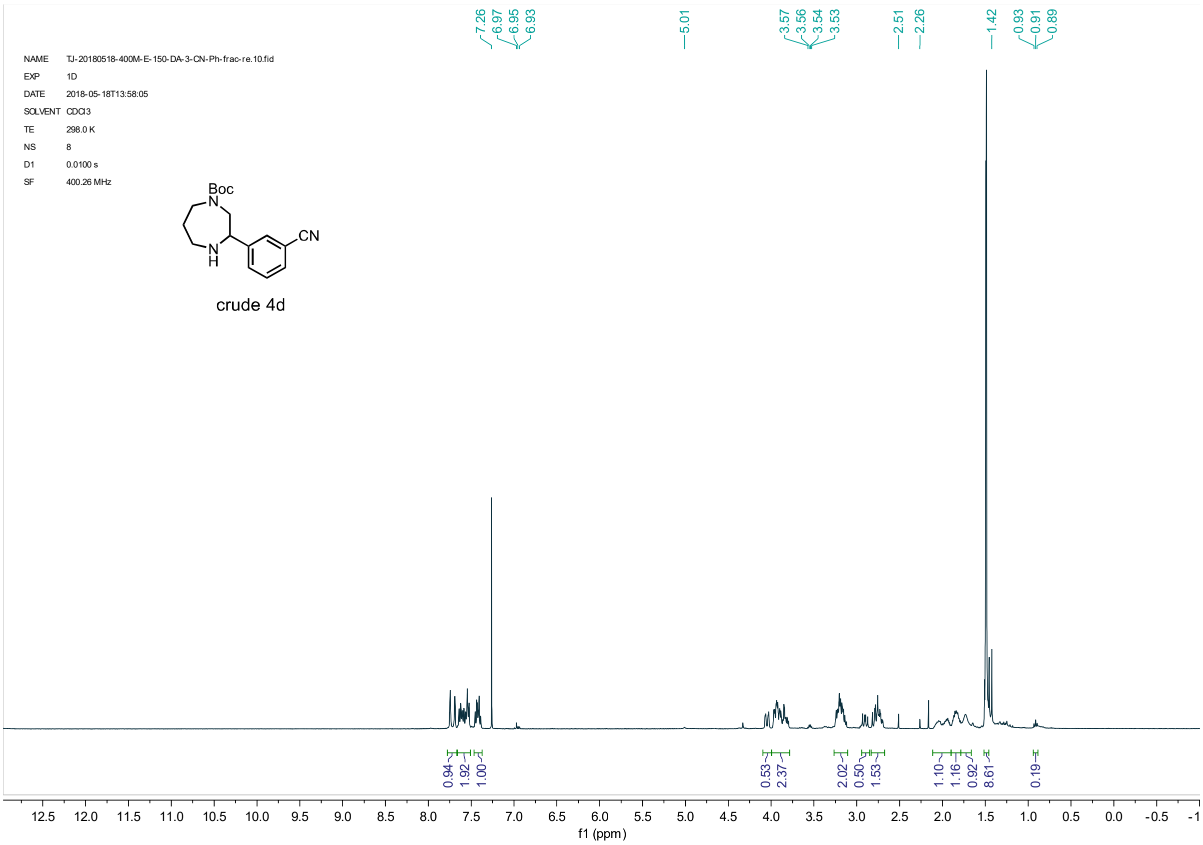

Internal standard: - .Volatile impurity: 2,6-lutidine (δ 6.95–6.93, 5.01, 2.51). Other impurity: BHT (δ 6.97, 5.00, 2.26, 1.42), “Sn” (δ 0.93–0.89).

^{1}H NMR of the purified product **4d**:

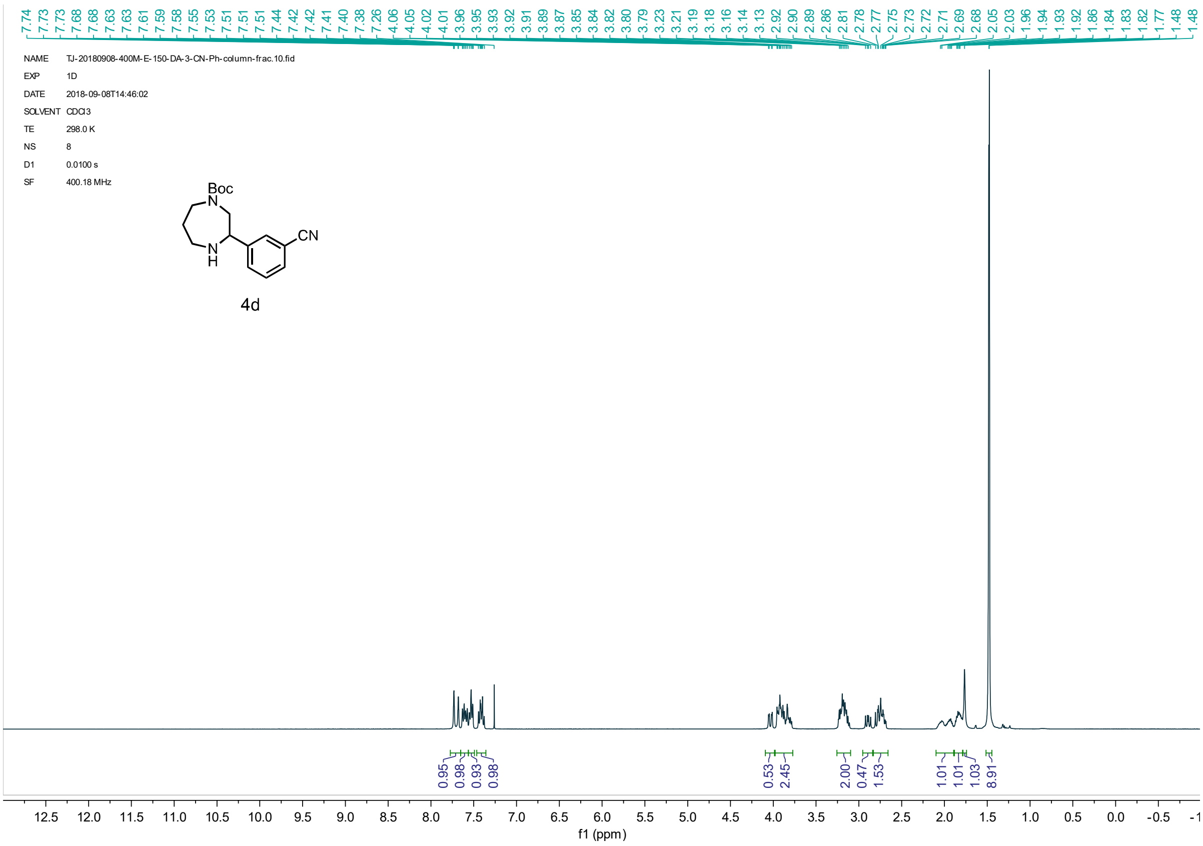

^{13}C NMR of the purified product **4d**:

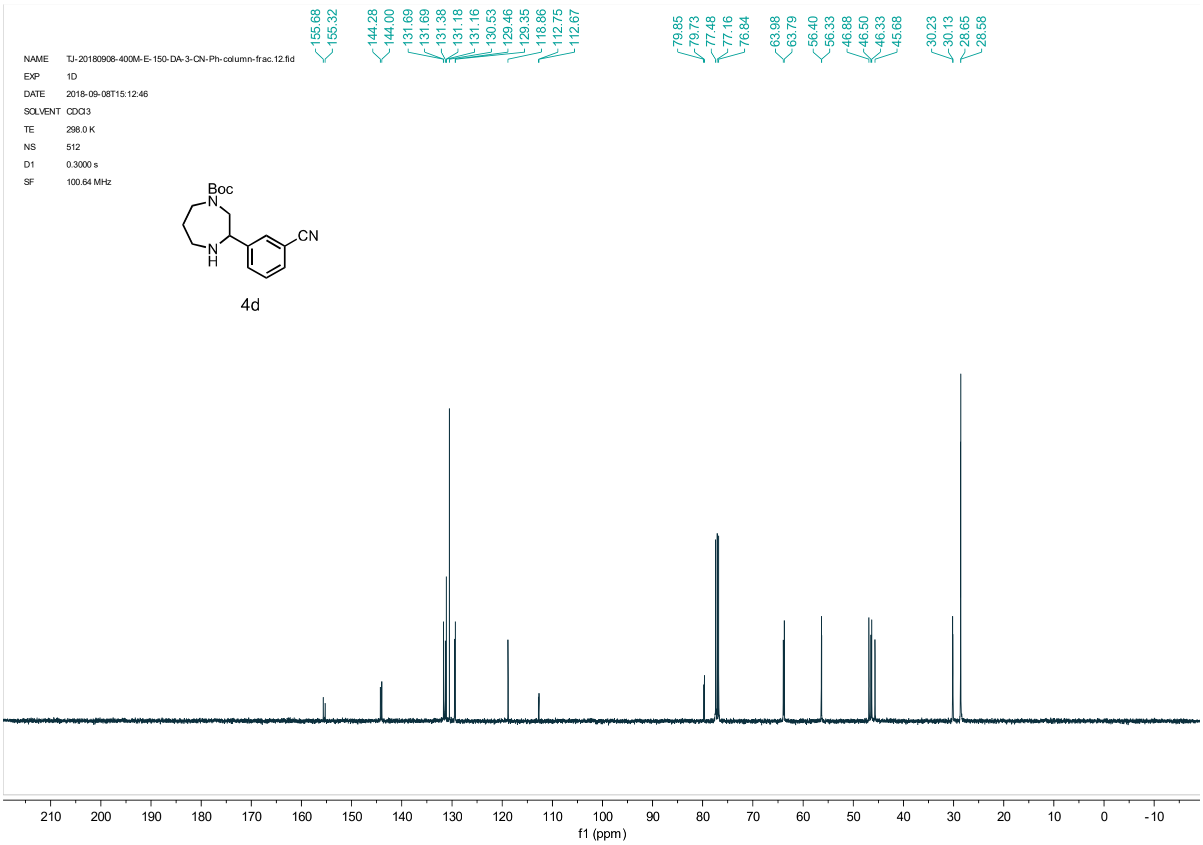

^{1}H NMR of the product **4e** obtained from Synple Chem synthesizer without purification:

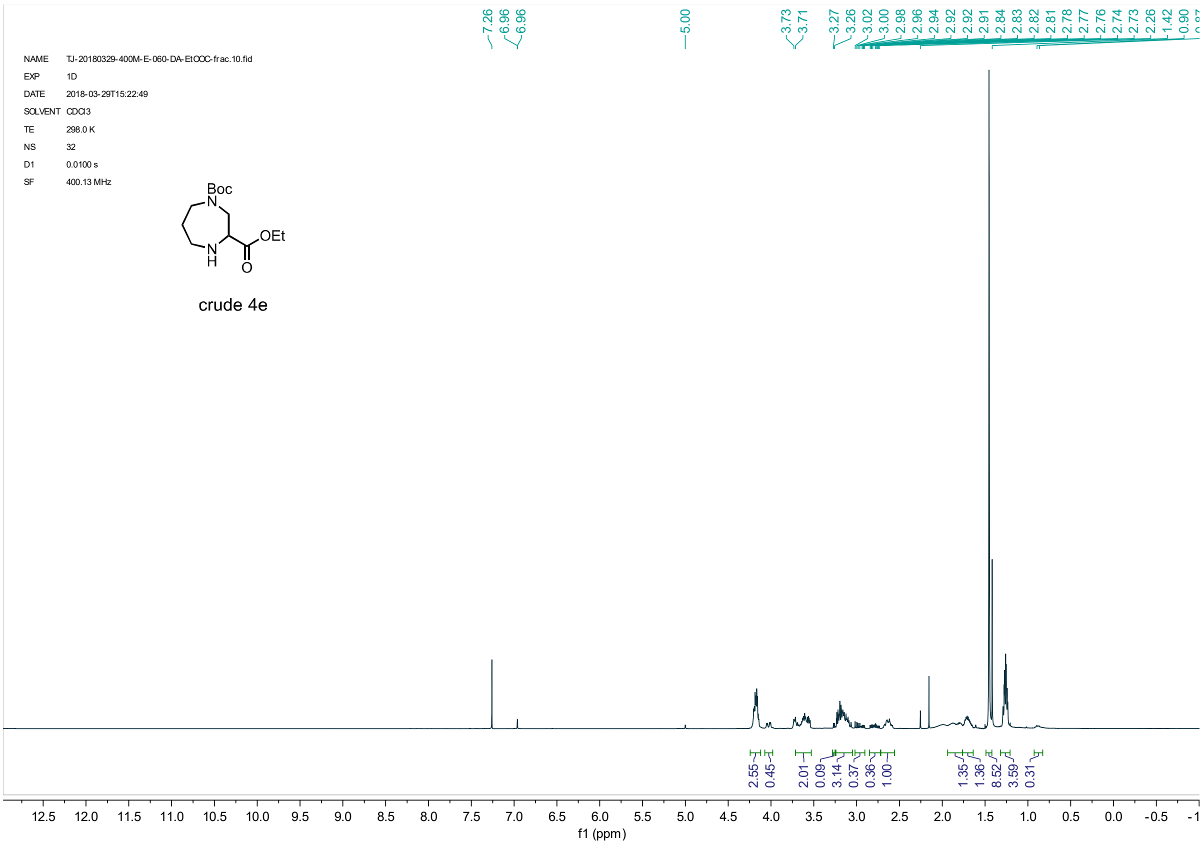

Internal standard: - . Volatile impurity: - .Other impurity: de-Boc product (*δ* 3.73–3.71, 3.02–2.73), BHT (*δ* 6.96, 5.00, 2.26, 1.42), "Sn" (*δ* 0.91–0.87).

^{1}H NMR of the purified product **4e**:

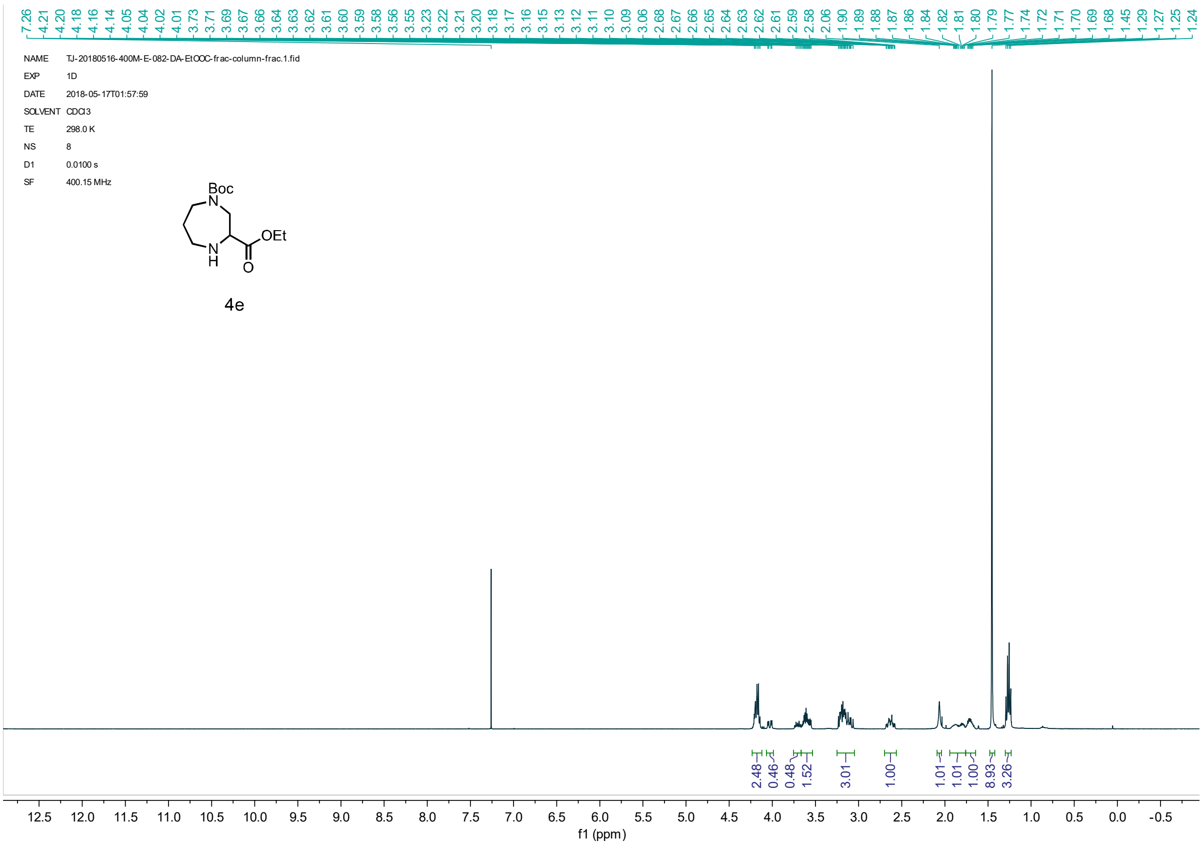

^{13}C NMR of the purified product **4e**:

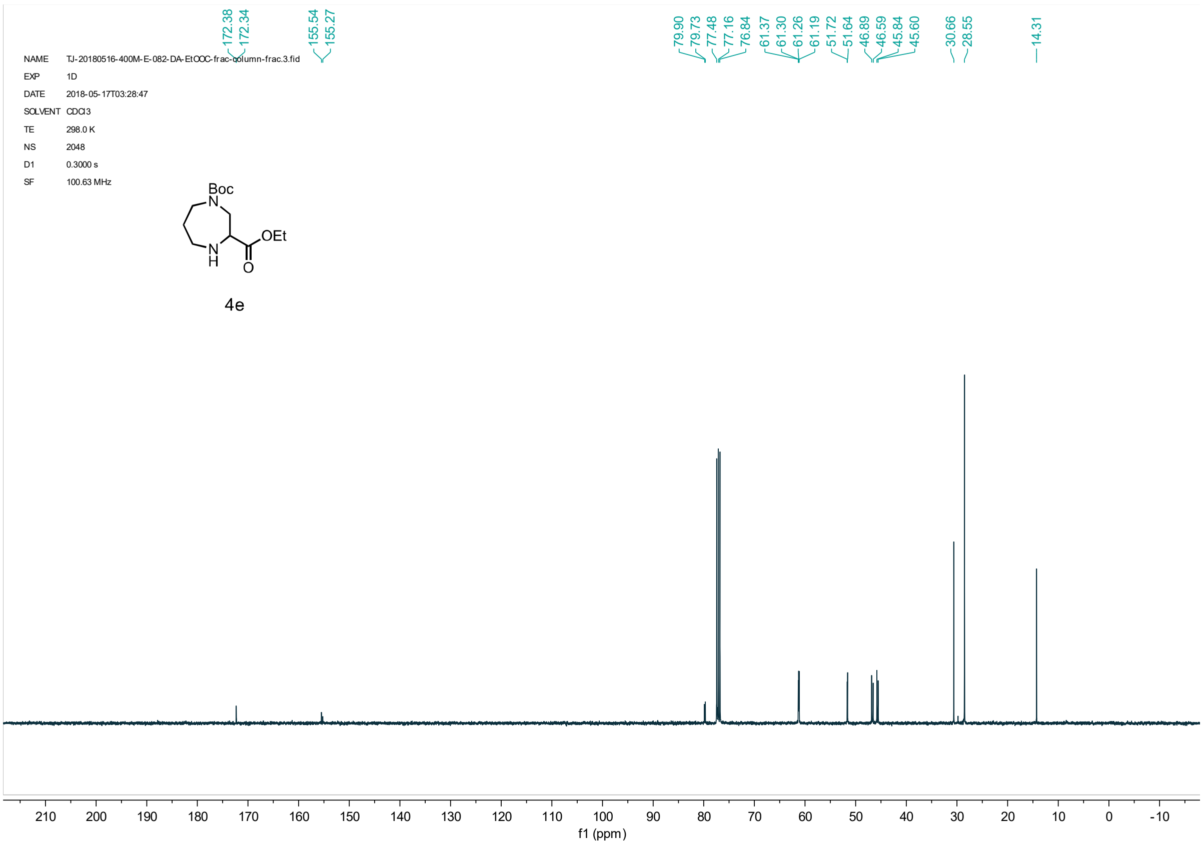

^{1}H NMR of the product **4f** obtained from Synple Chem synthesizer without purification:

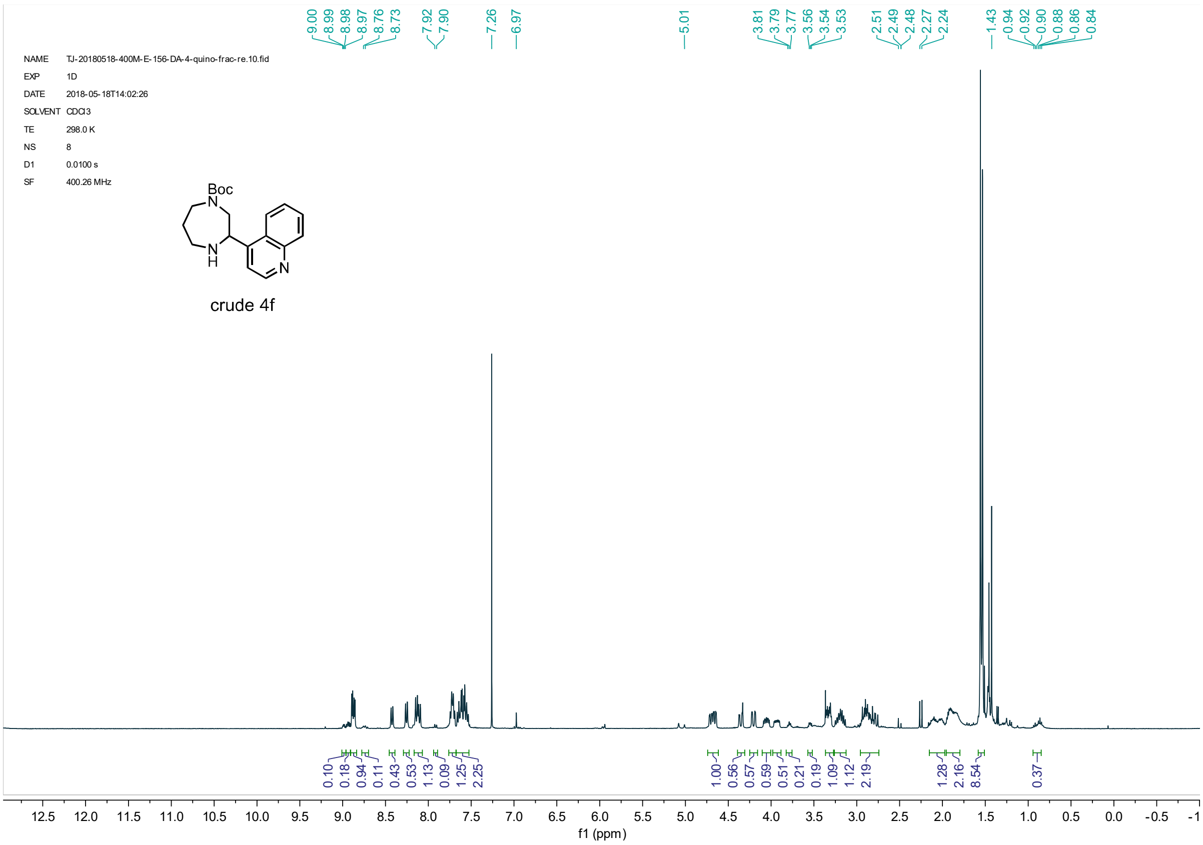

Internal standard: - . Volatile impurity: - . Other impurity: de-Boc product (*δ* 9.00–8.97, 7.92–7.90, 3.81–3.77, 3.56–3.53, 2.51–2.48, 2.24), BHT (*δ* 6.97, 5.01, 2.27, 1.43), “Sn” (*δ* 0.94–0.84).

^{1}H NMR of the purified product **4f**:

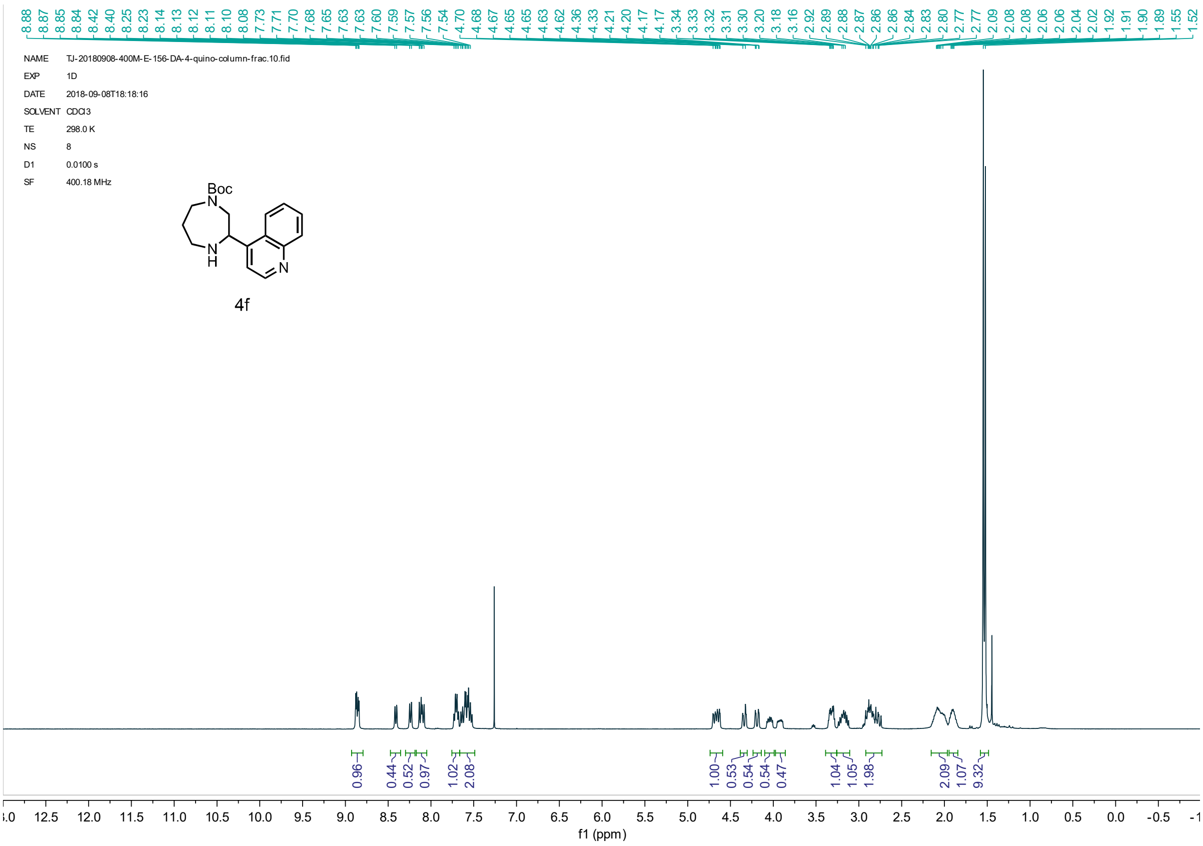

^{13}C NMR of the purified product **4f**:

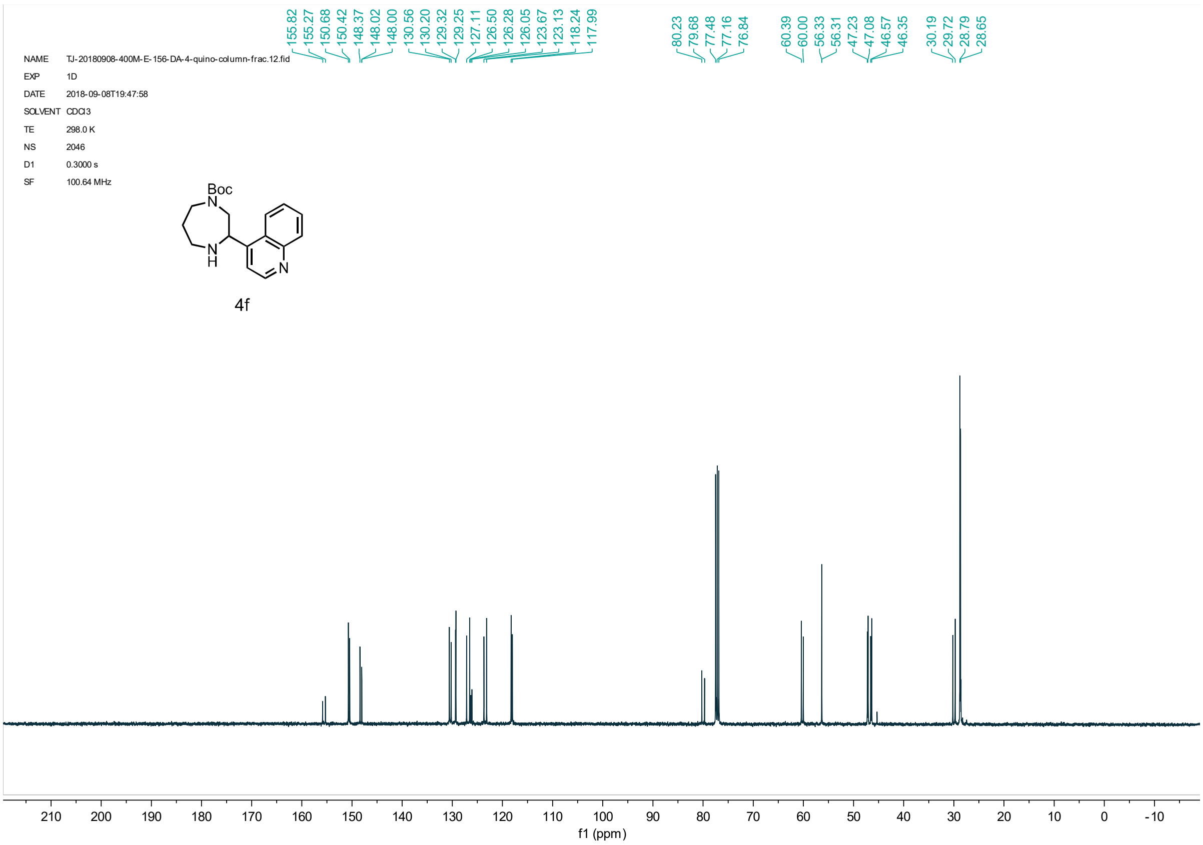

^{1}H NMR of the product **5a** obtained from Synple Chem synthesizer without purification:

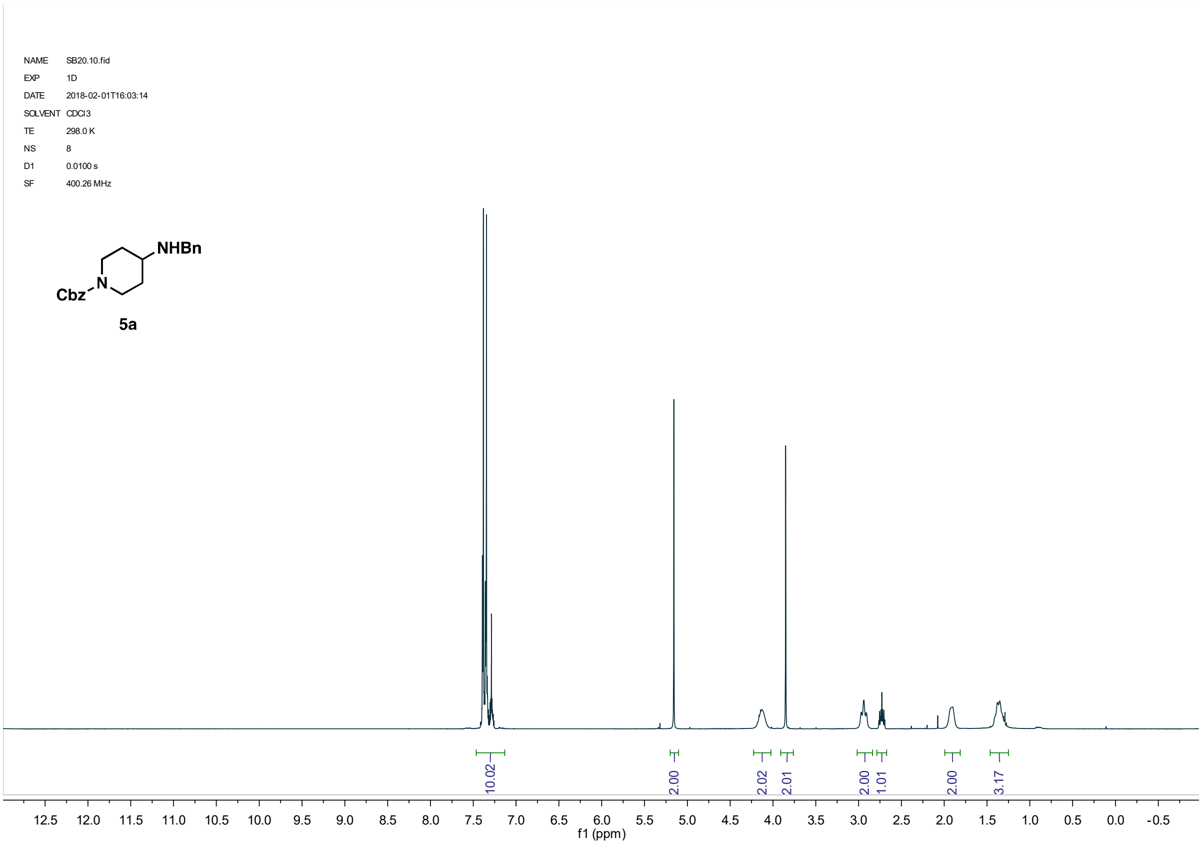

^{13}C NMR of the product **5a**:

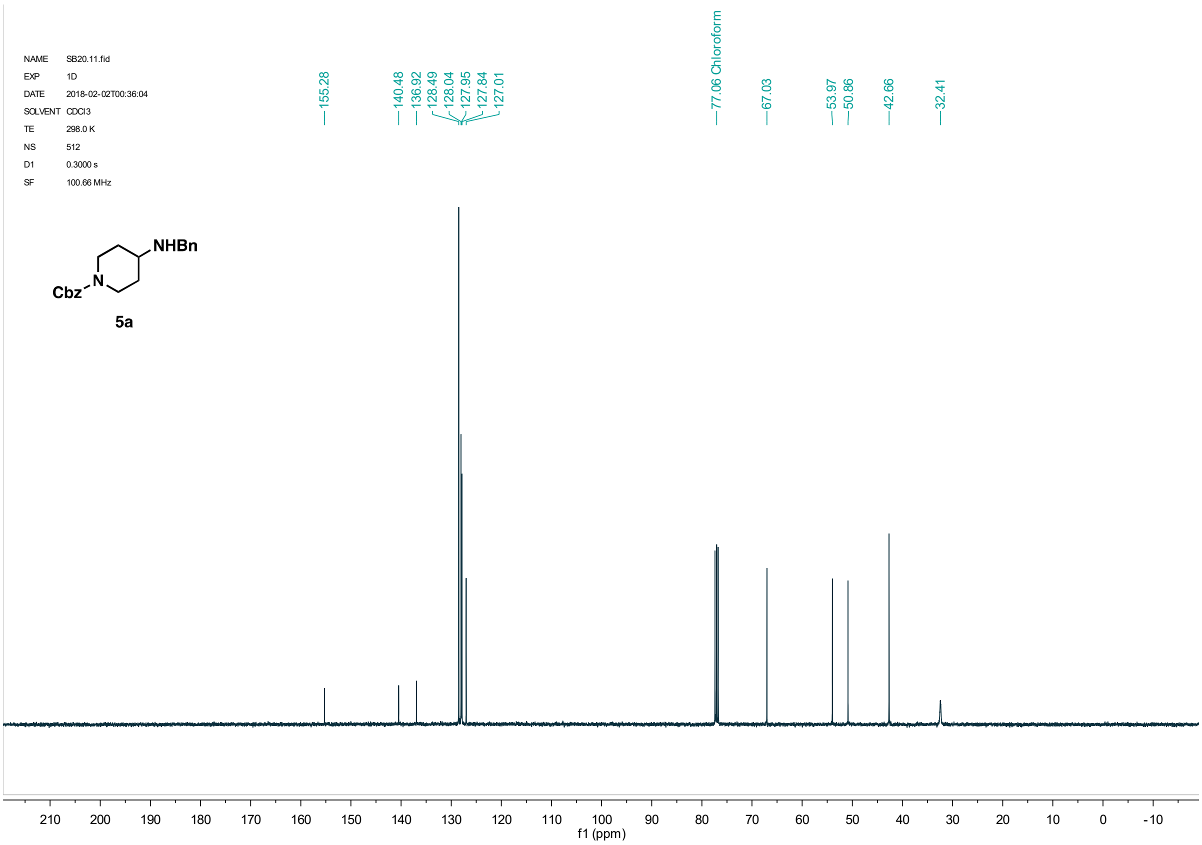

^{1}H NMR of the product **5b** obtained from Synple Chem synthesizer without purification:

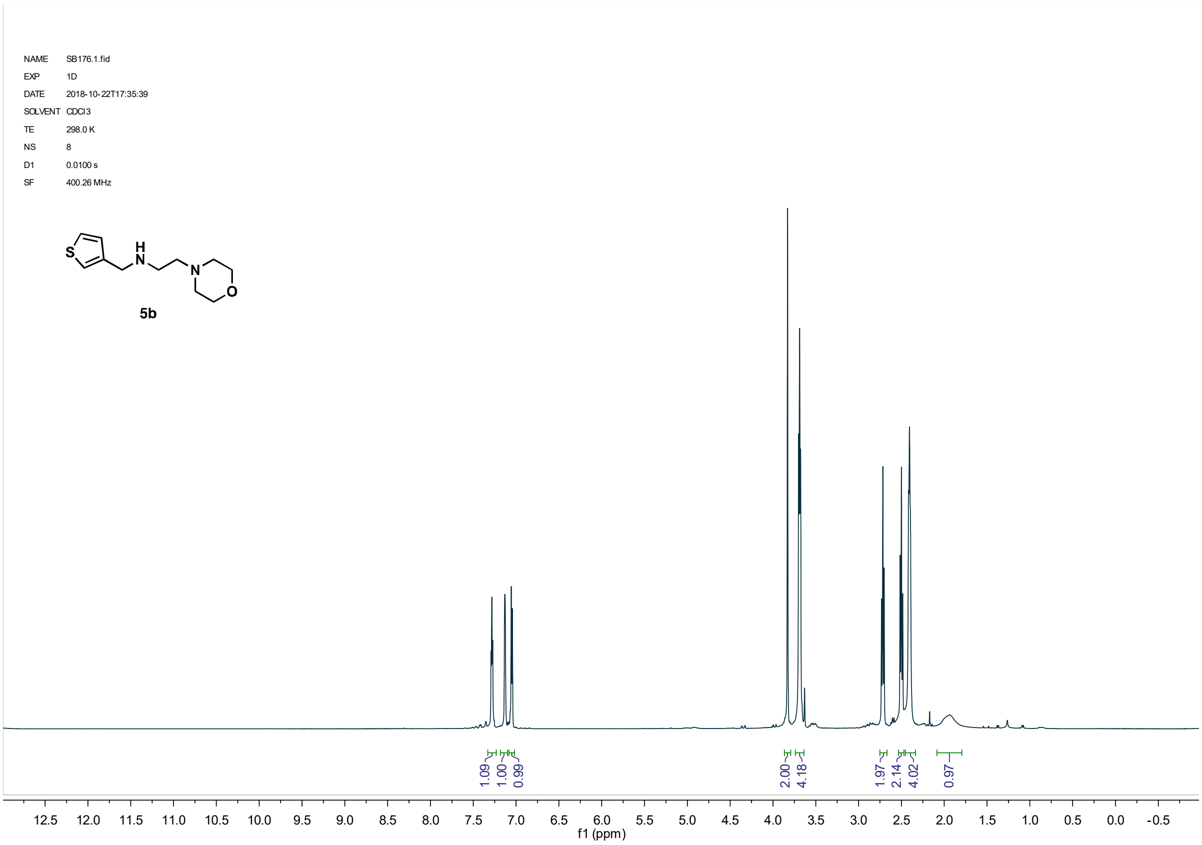

^{13}C NMR of the product **5b**:

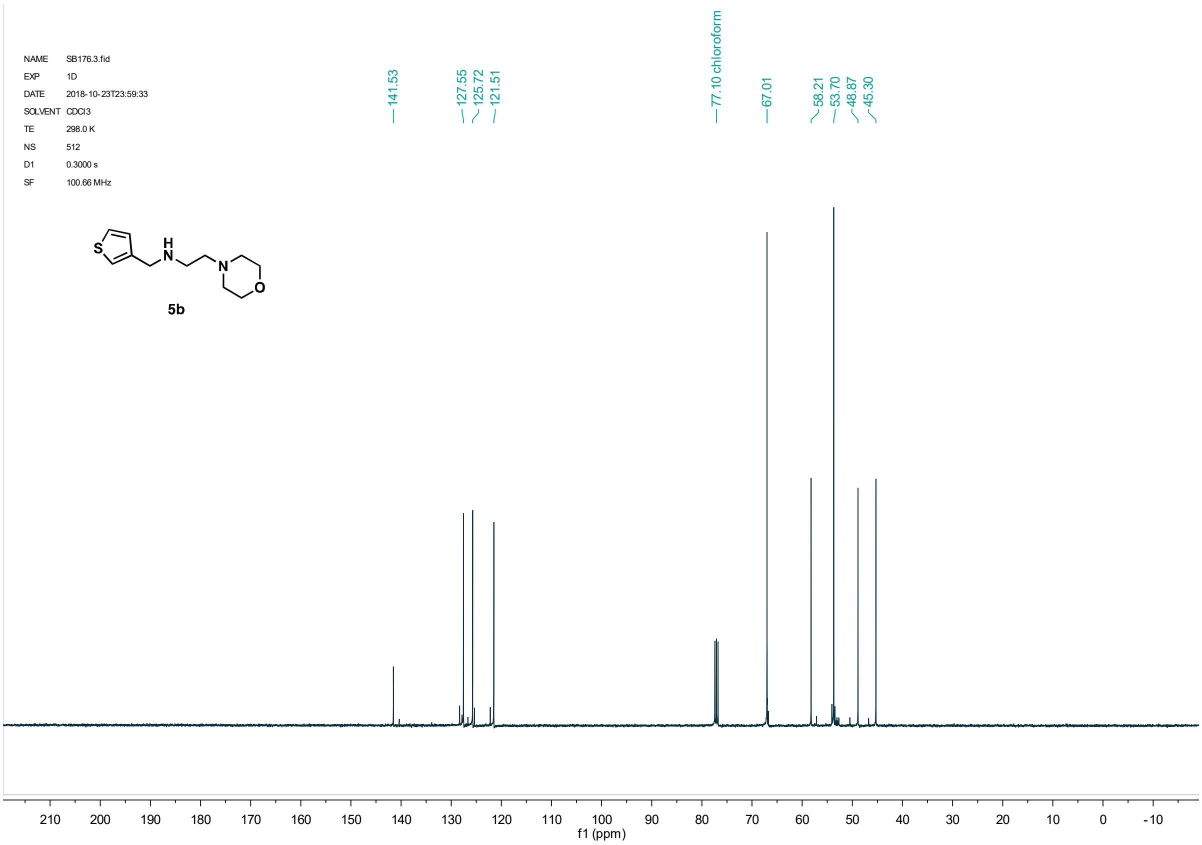

^{1}H NMR of the product **5c** obtained from Synple Chem synthesizer without purification:

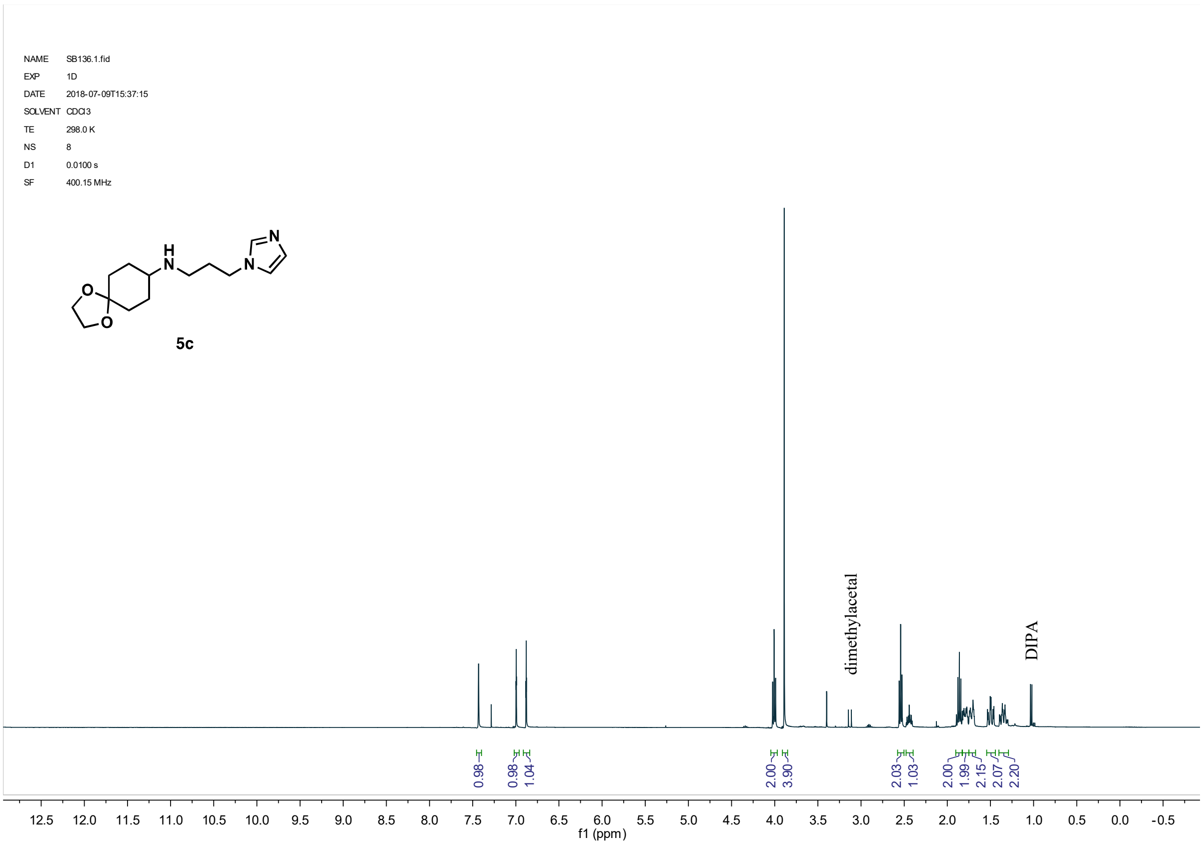

^{13}C NMR of the product **5c**:

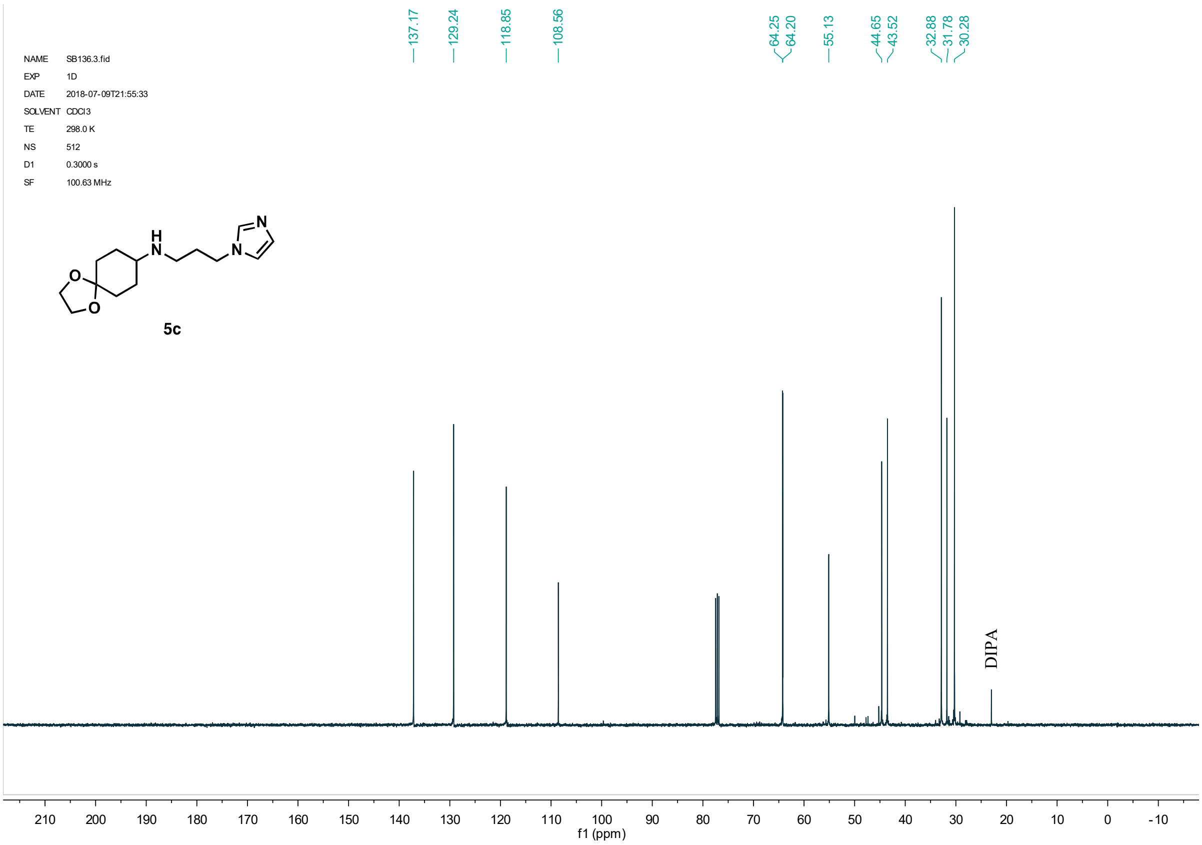

^{1}H NMR of the product **5d** obtained from Synple Chem synthesizer without purification:

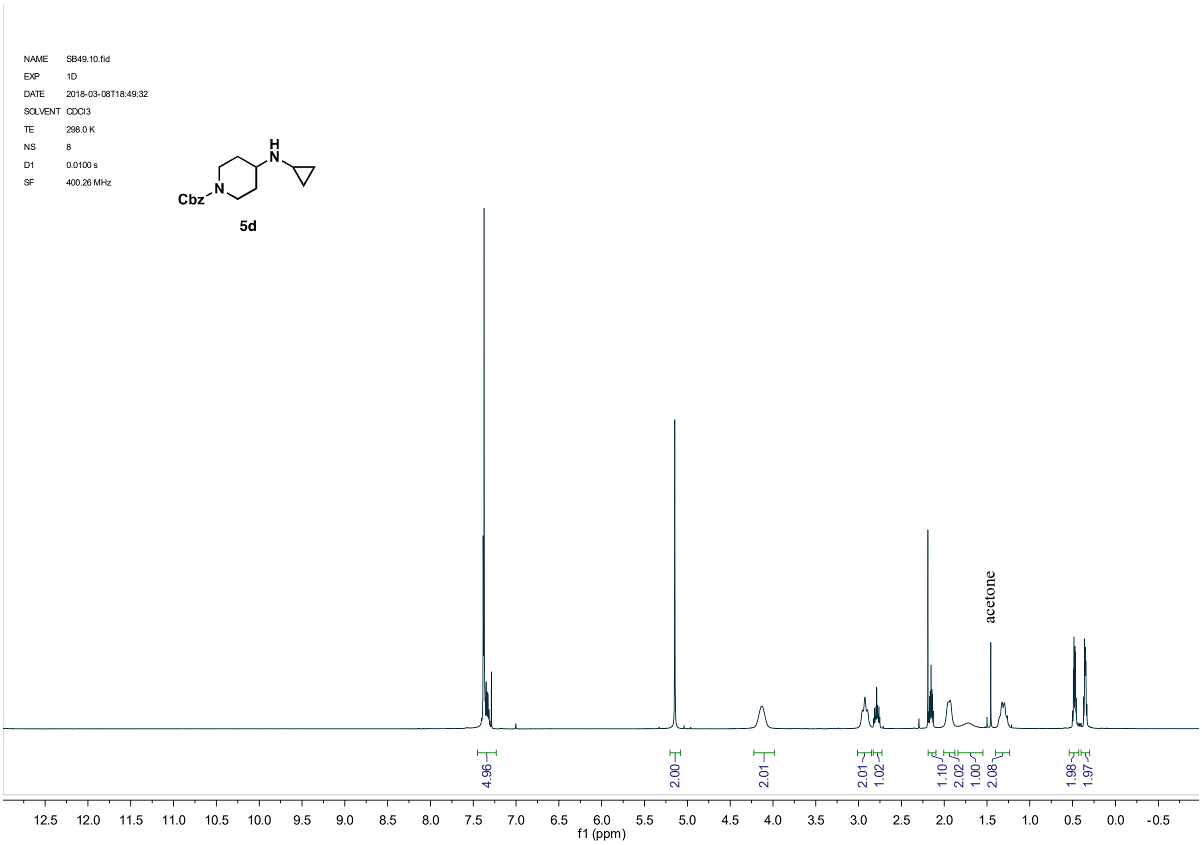

^{13}C NMR of the product **5d**:

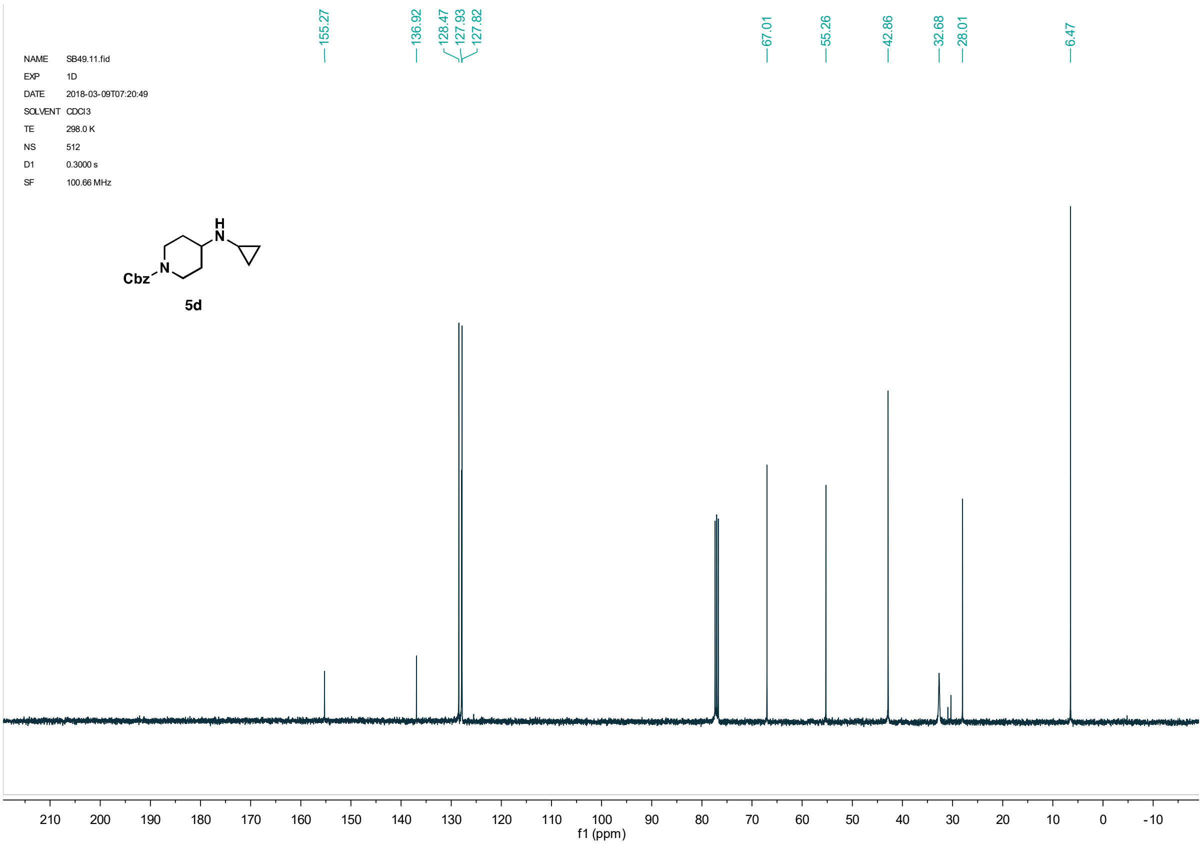

^{1}H NMR of the product **5e** obtained from Synple Chem synthesizer without purification:

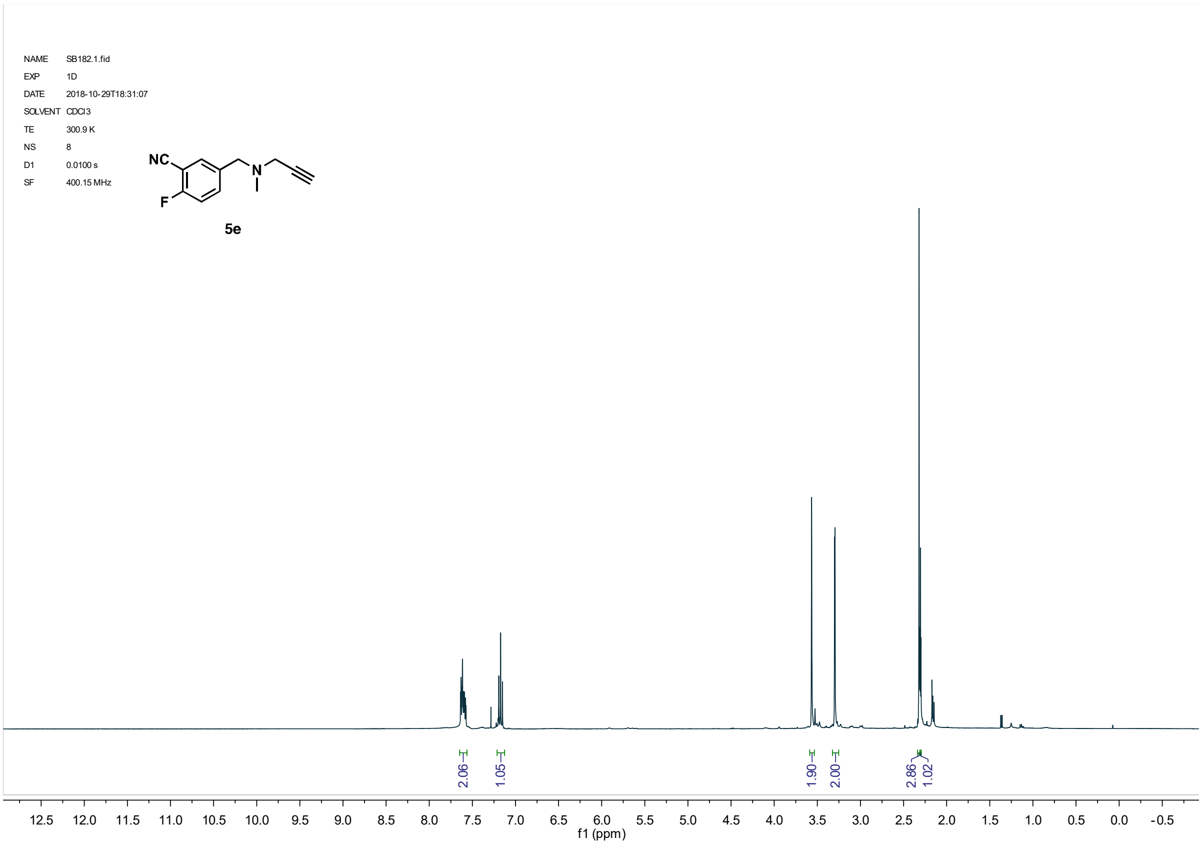

^{13}C NMR of the product **5e**:

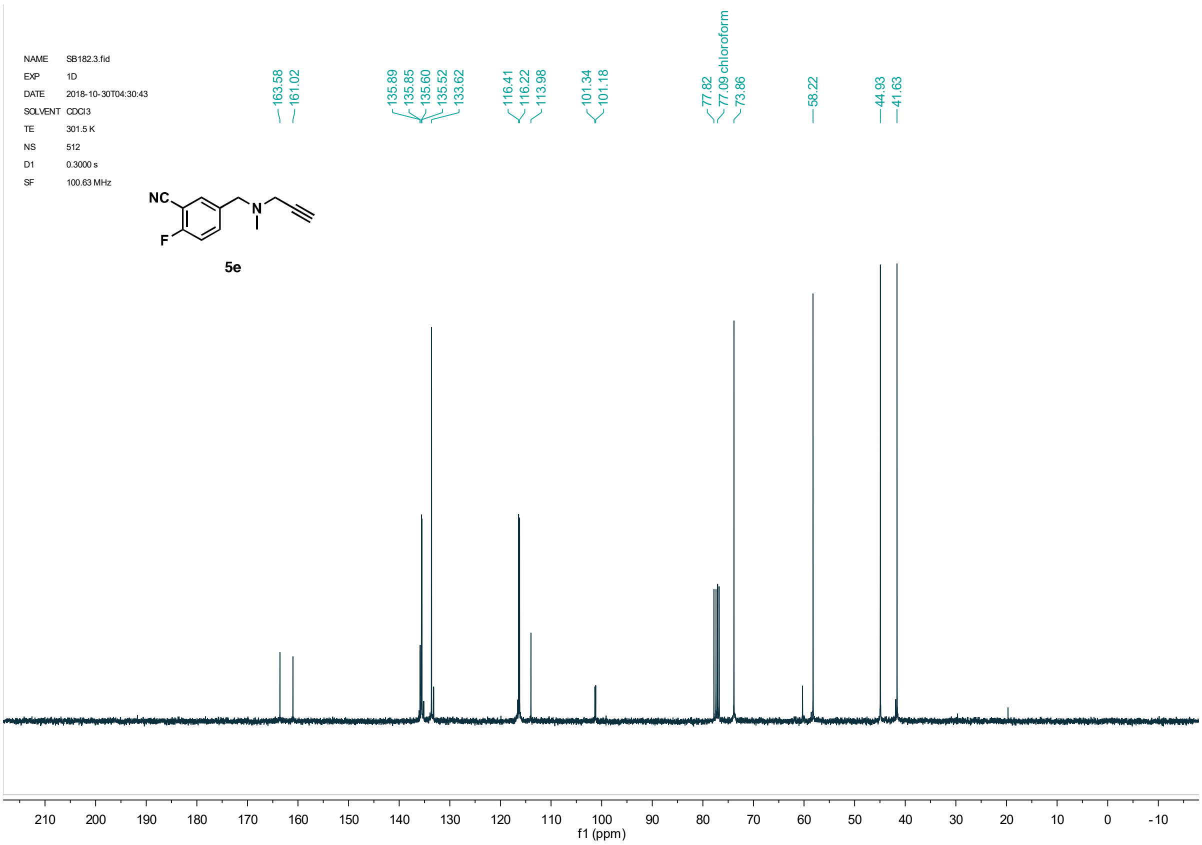

^{19}F NMR of the product **5e**:

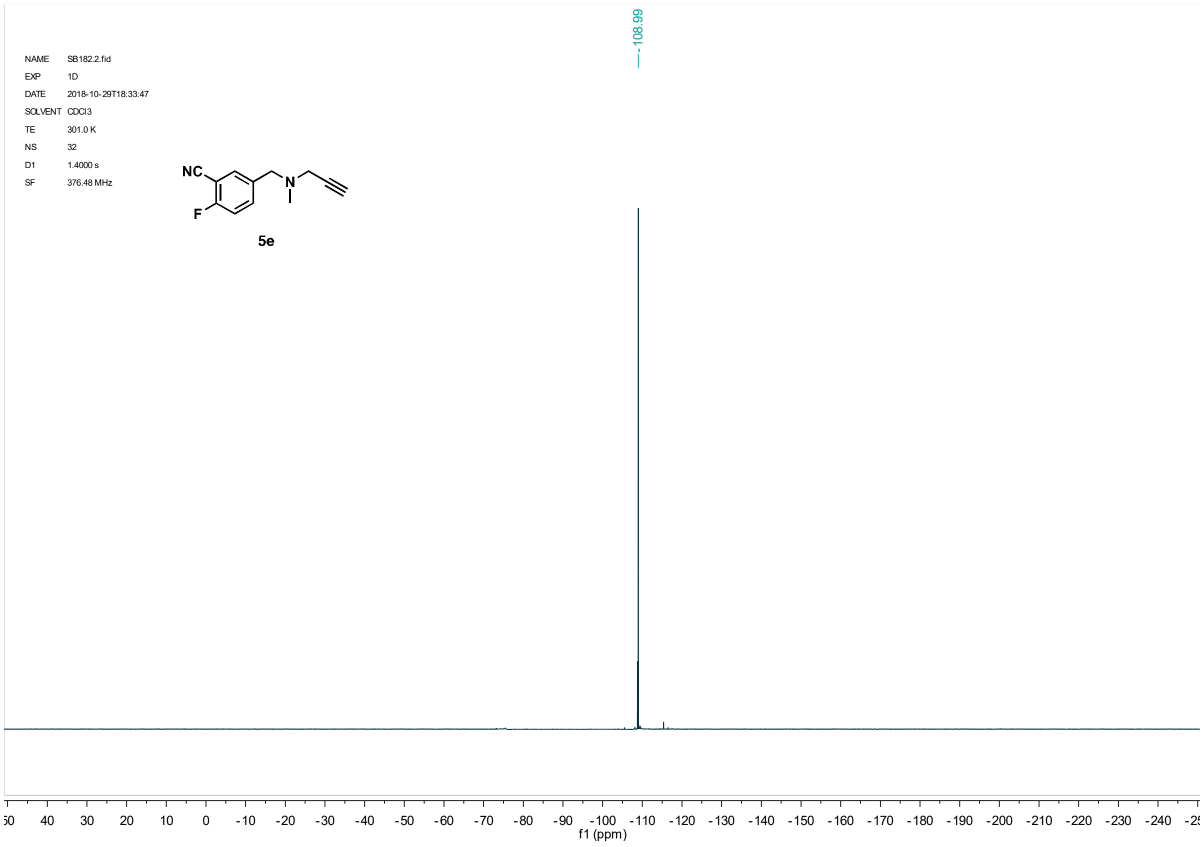

^{1}H NMR of the product **5f** obtained from Synple Chem synthesizer without purification:

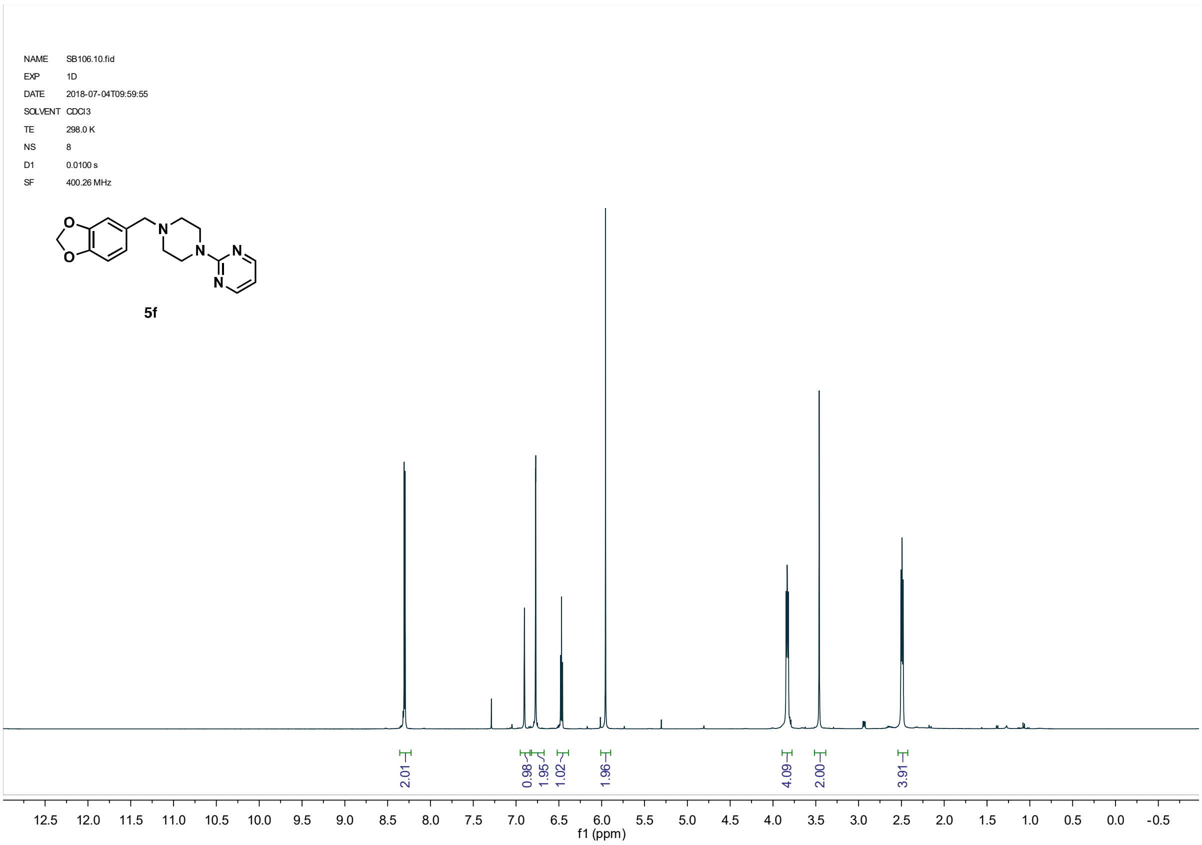

^{13}C NMR of the product **5f**:

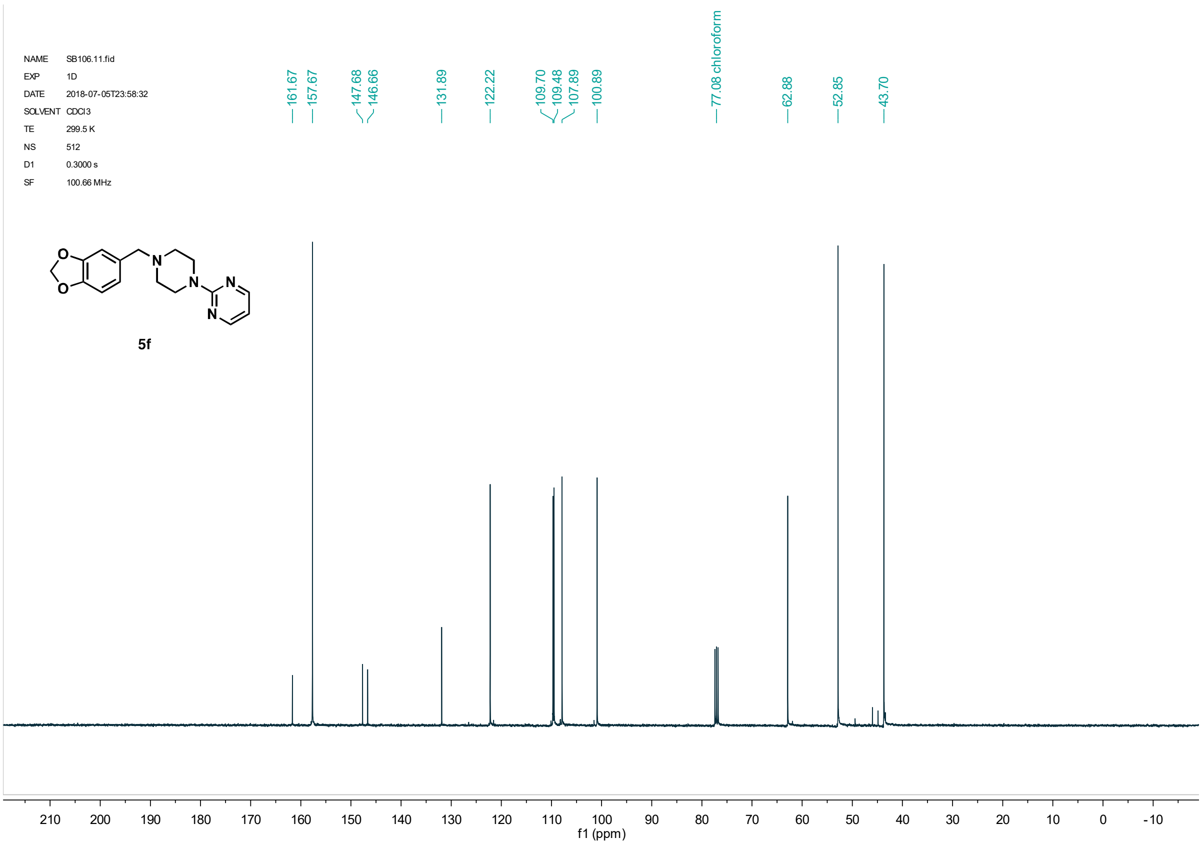

^{1}H NMR of the product **5g** obtained from Synple Chem synthesizer without purification:

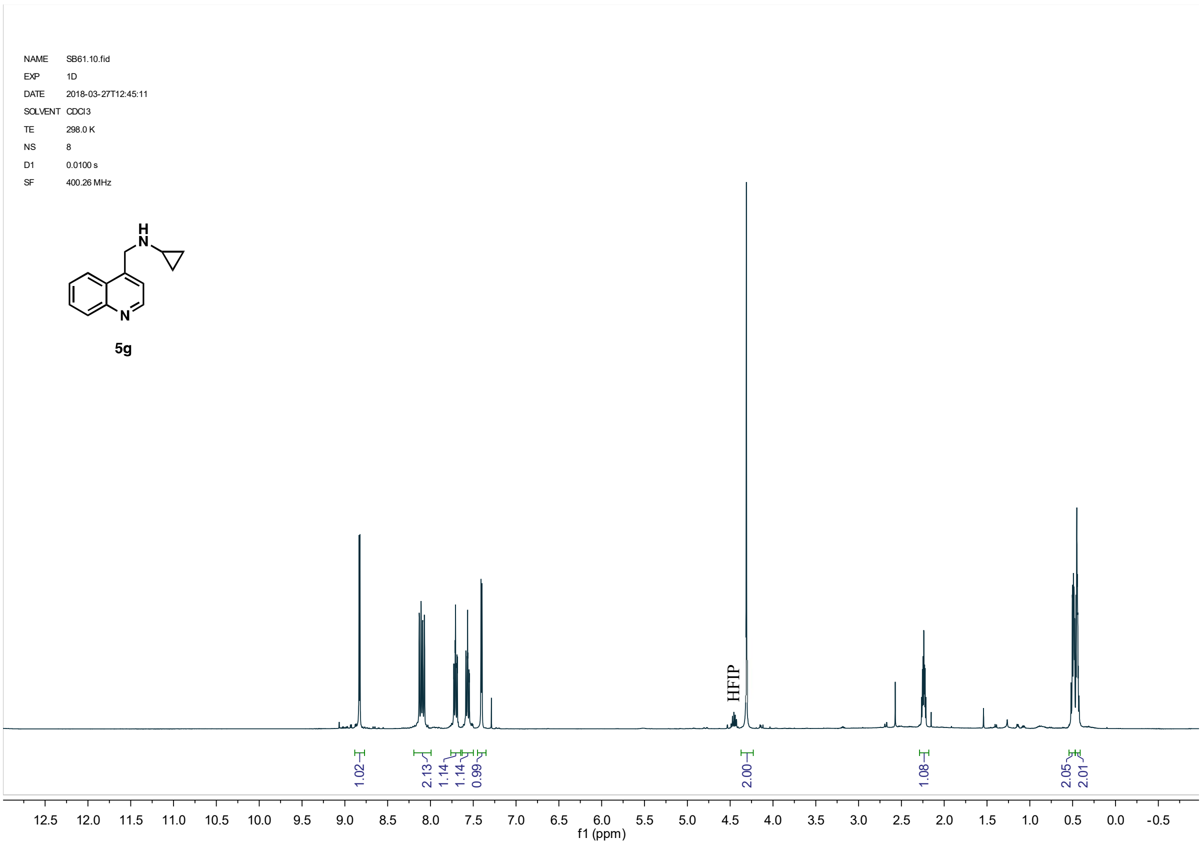

^{13}C NMR of the product **5g**:

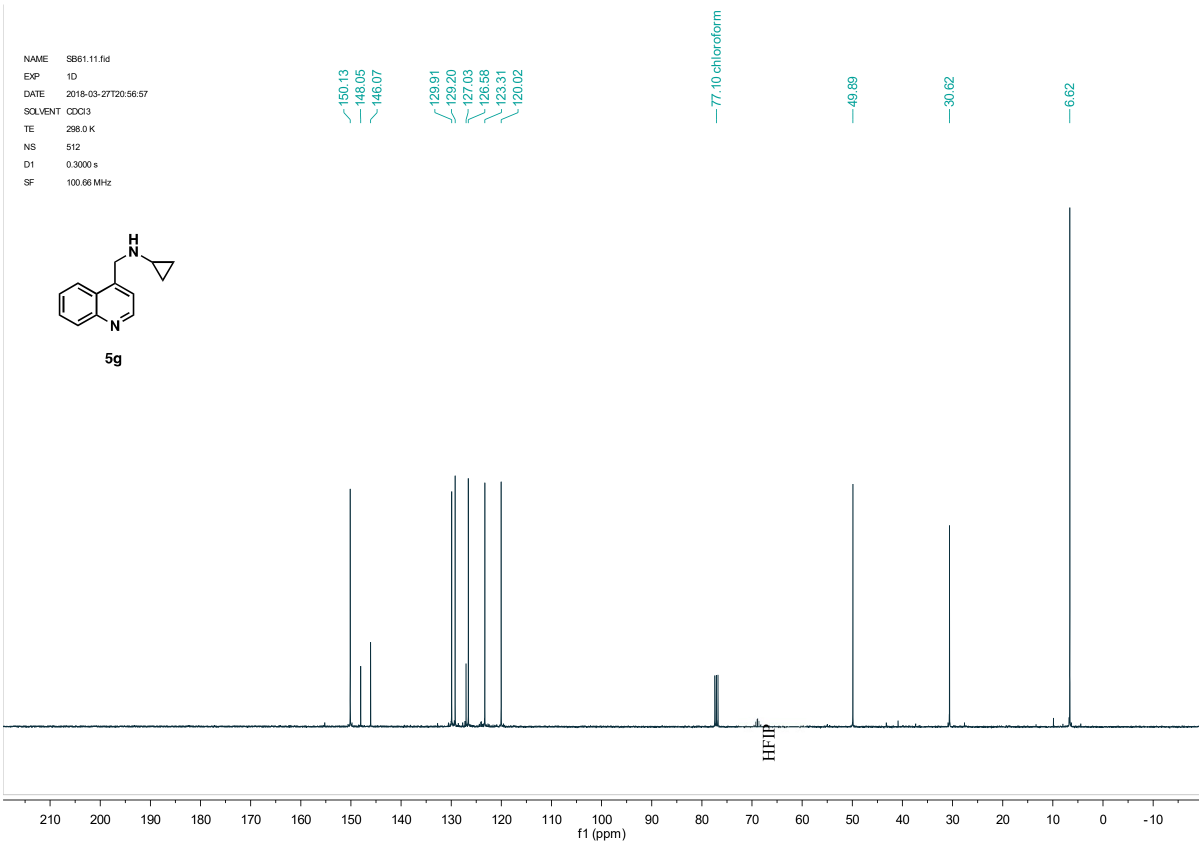

^{1}H NMR of the product **5h** obtained from Synple Chem synthesizer without purification:

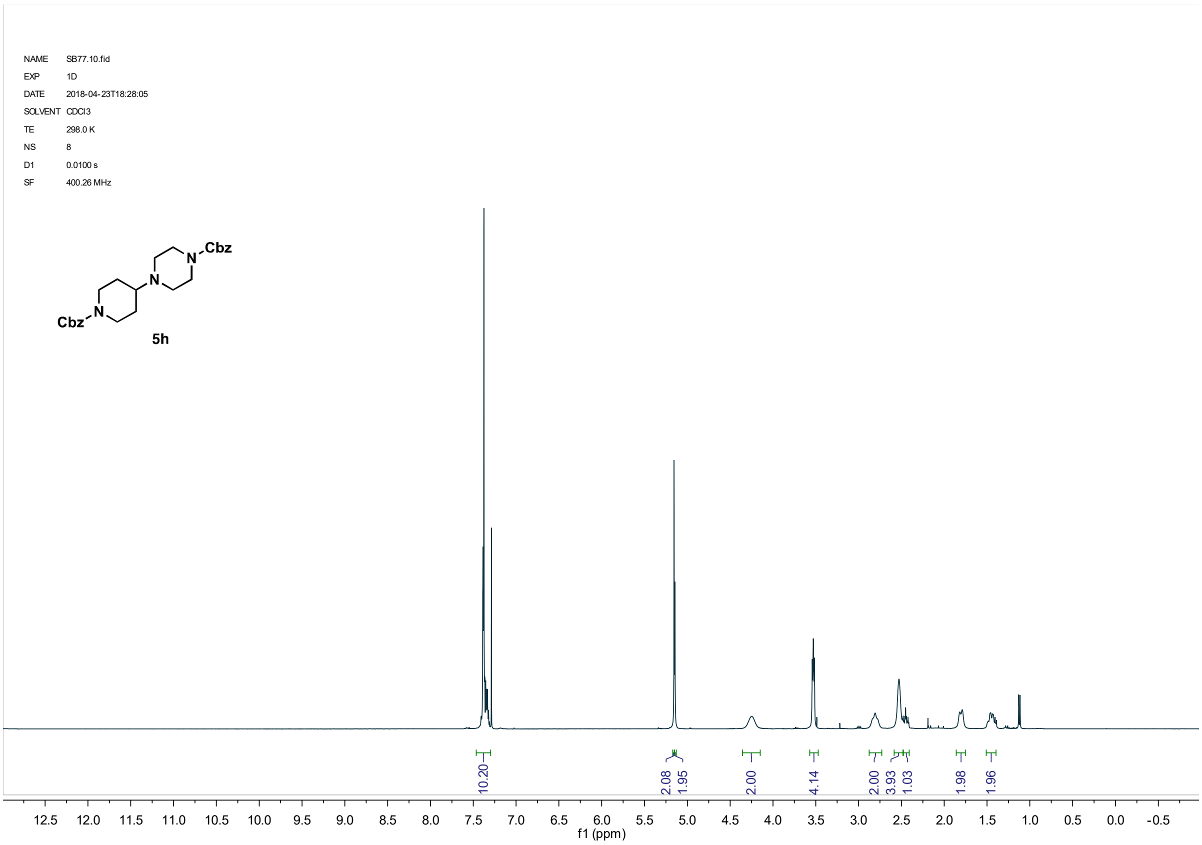

^{13}C NMR of the product **5h**:

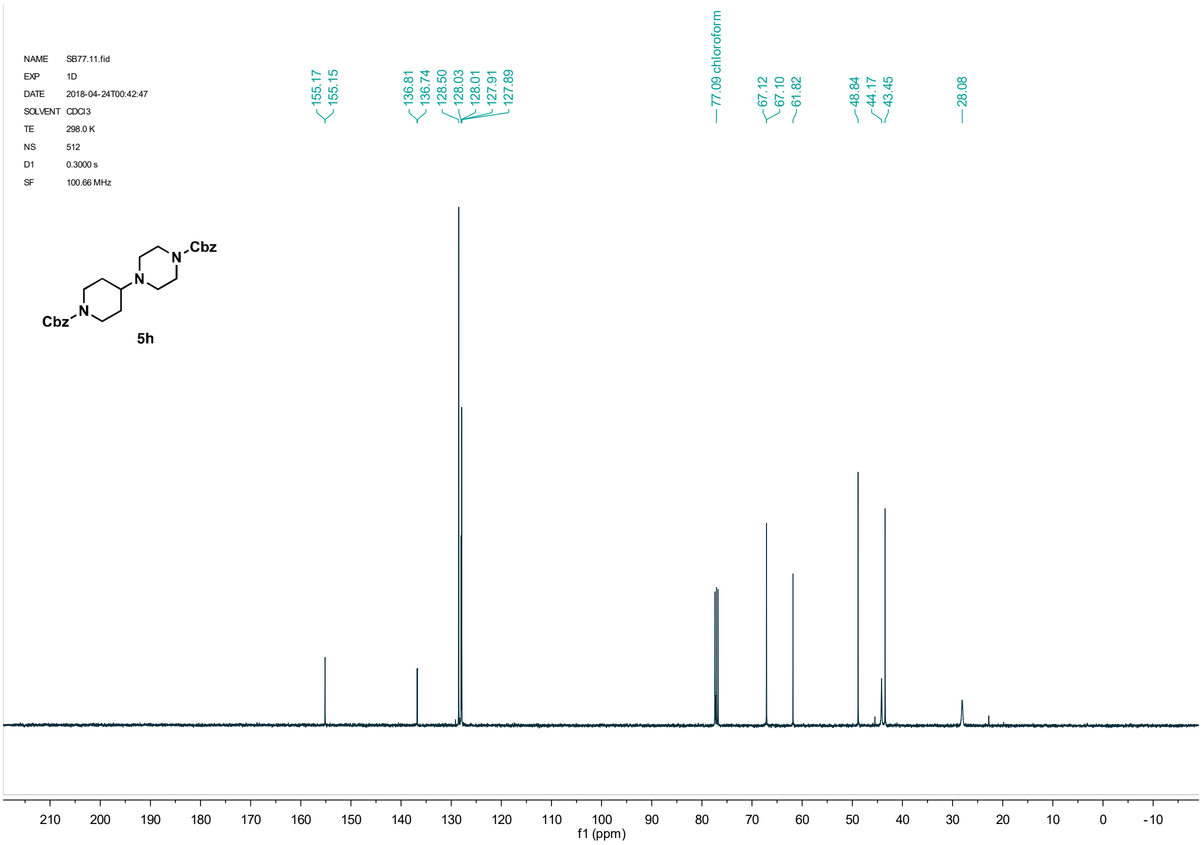

^{1}H NMR of the product **5i** obtained from Synple Chem synthesizer without purification:

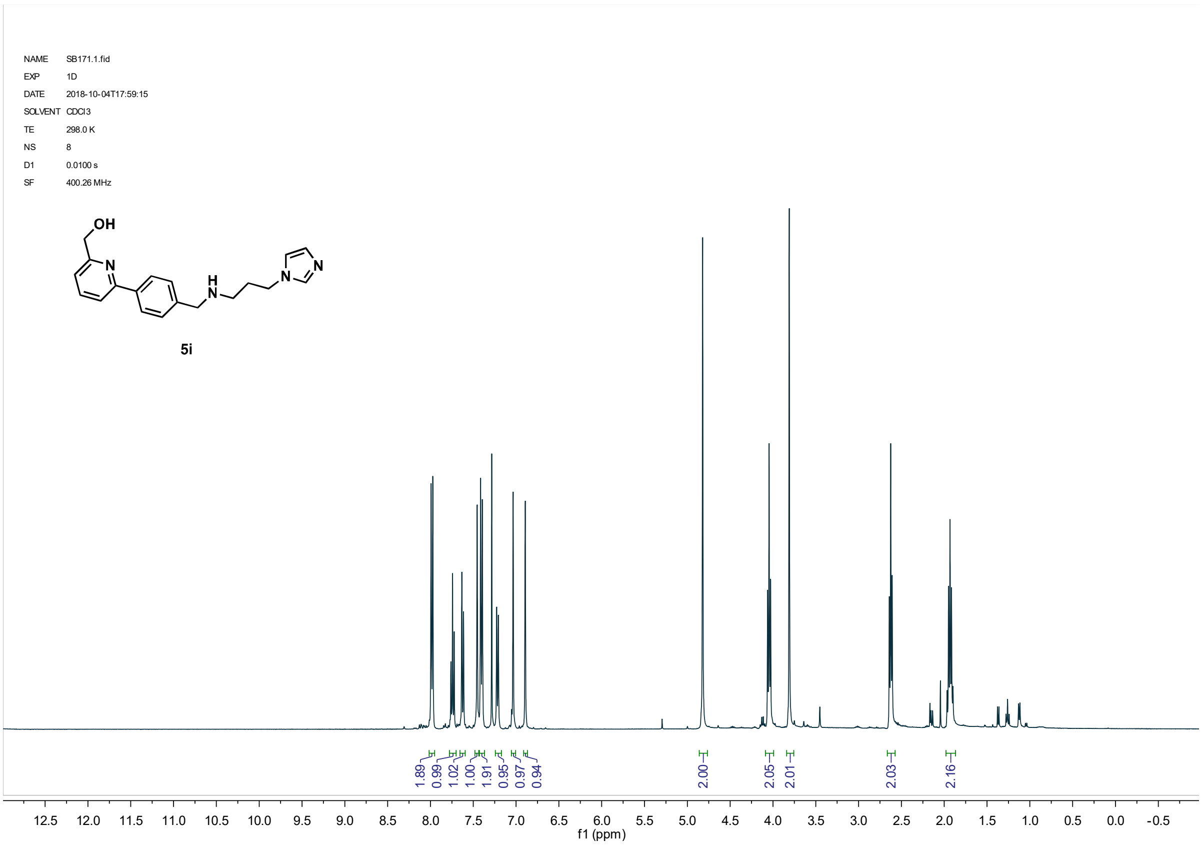

^{13}C NMR of the product **5i**:

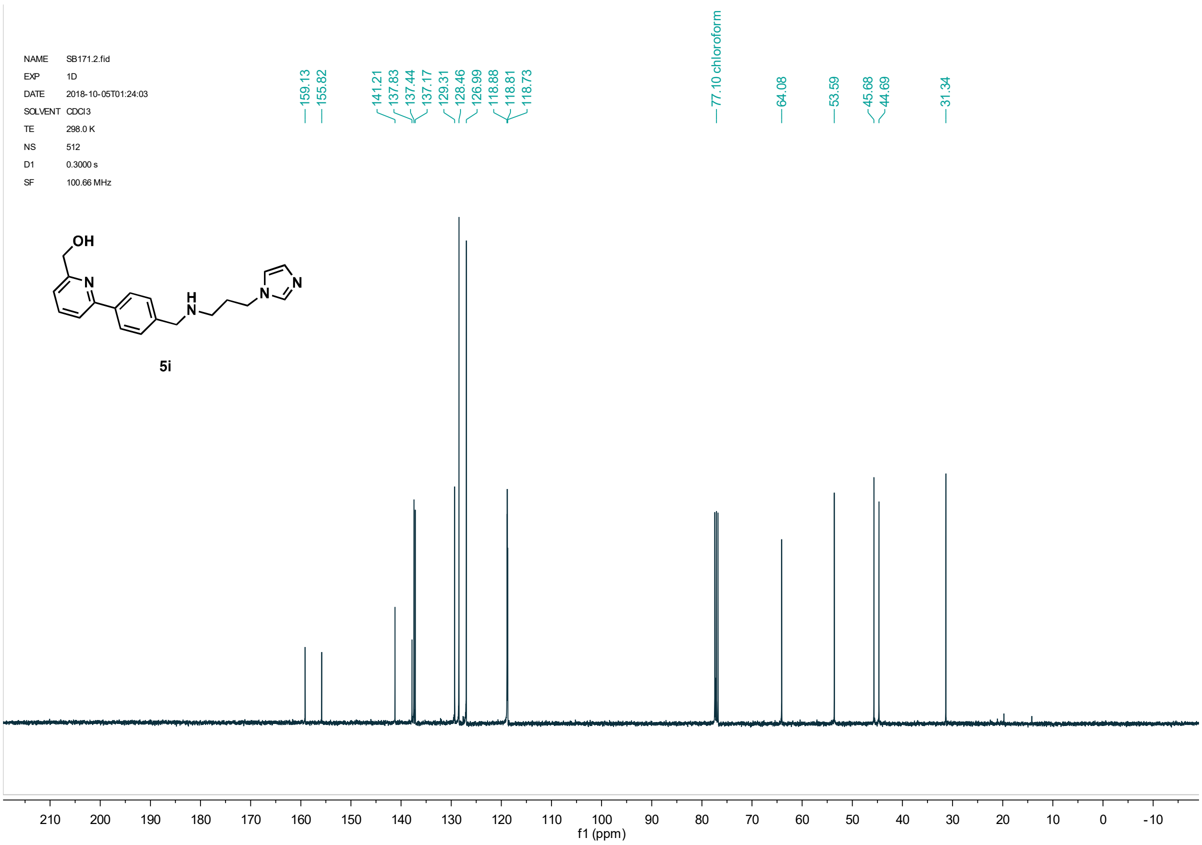

^{1}H NMR of the product **5j** obtained from Synple Chem synthesizer without purification:

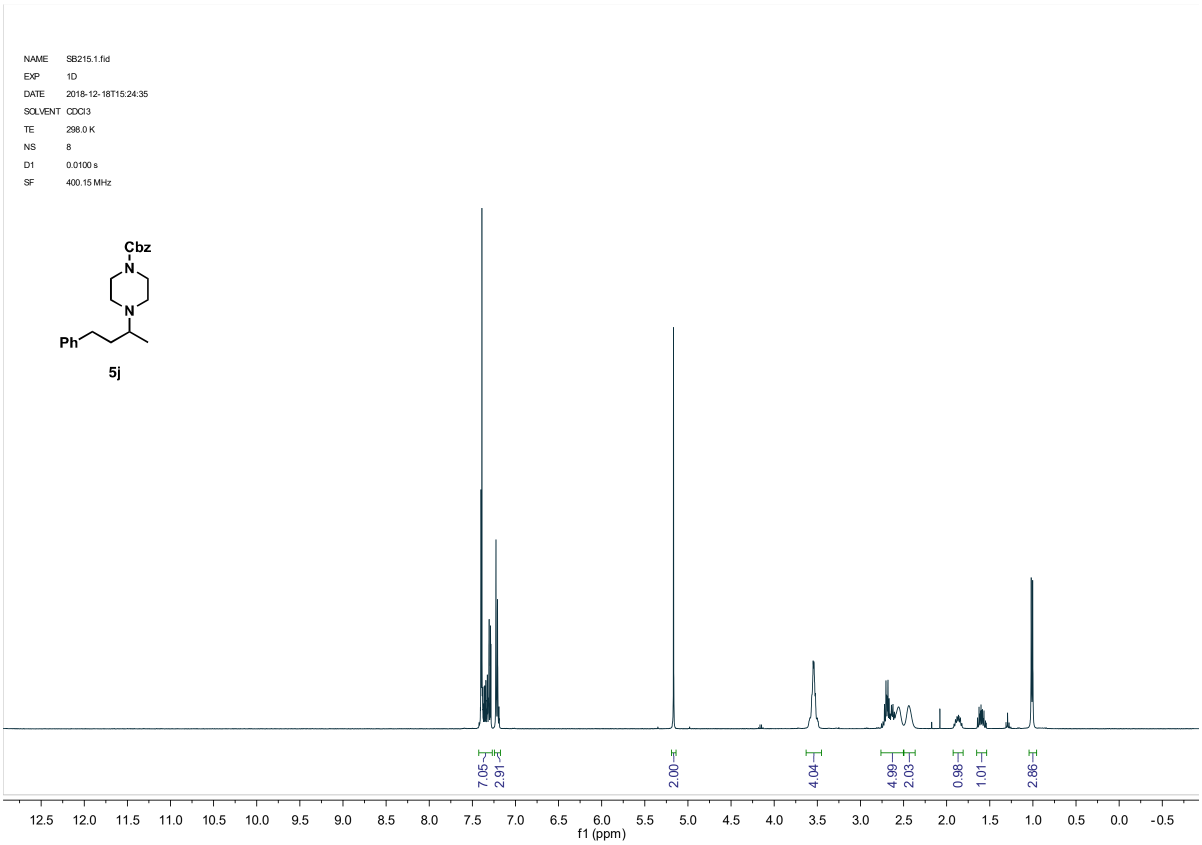

^{13}C NMR of the product **5j**:

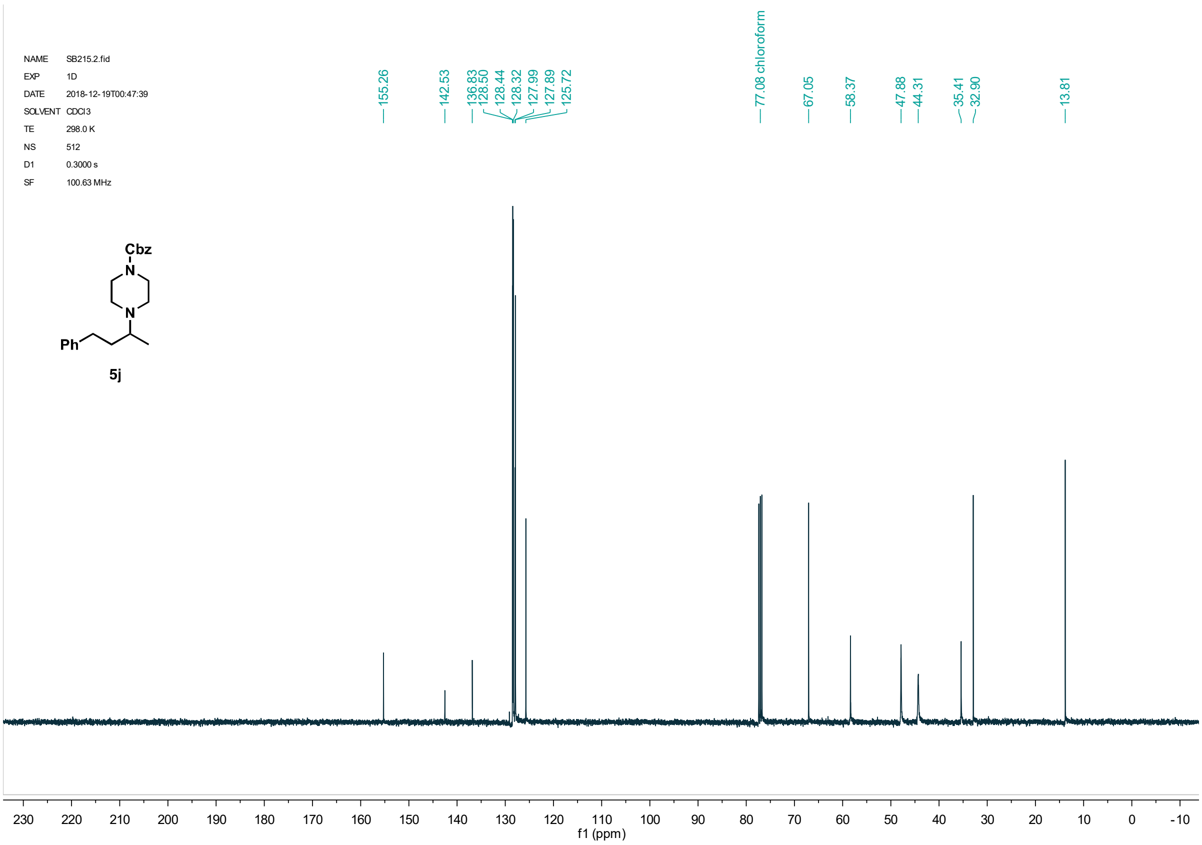

^{1}H NMR of the product **5k** obtained from Synple Chem synthesizer without purification:

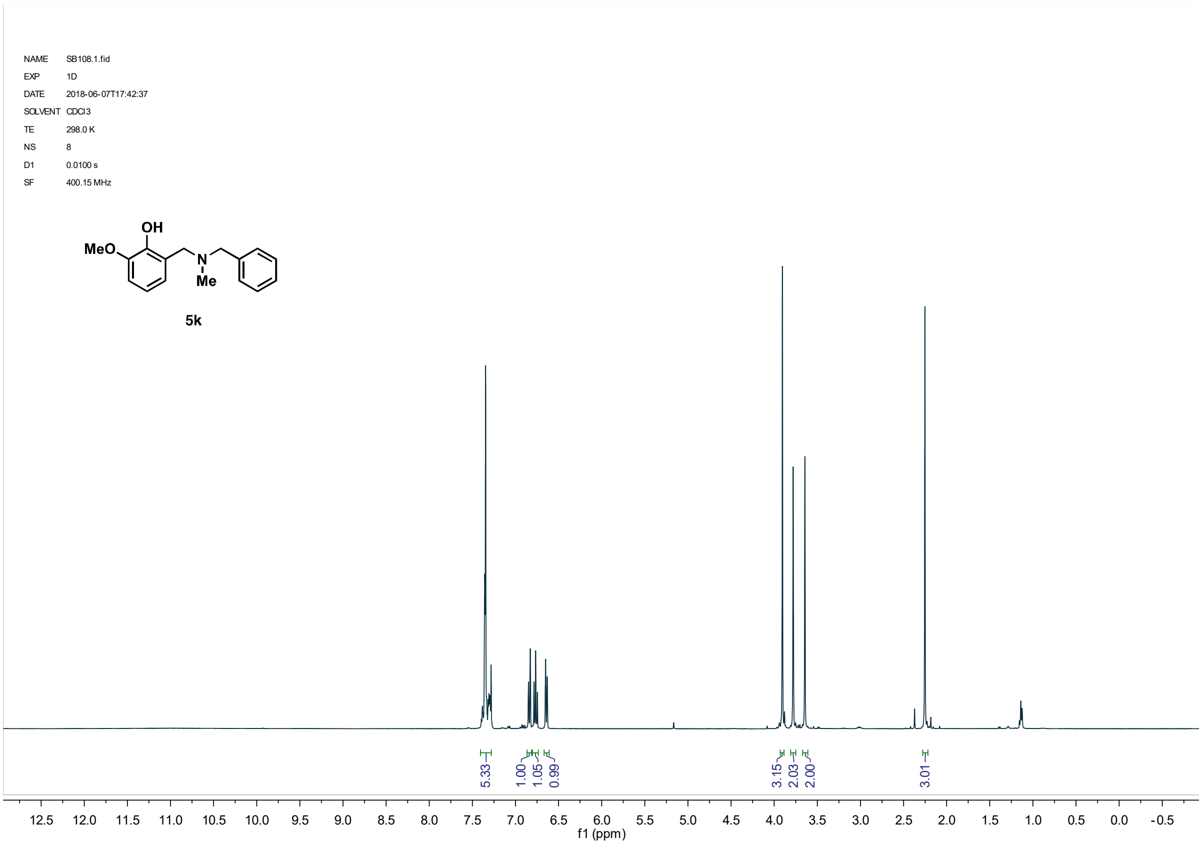

^{13}C NMR of the product **5k**:

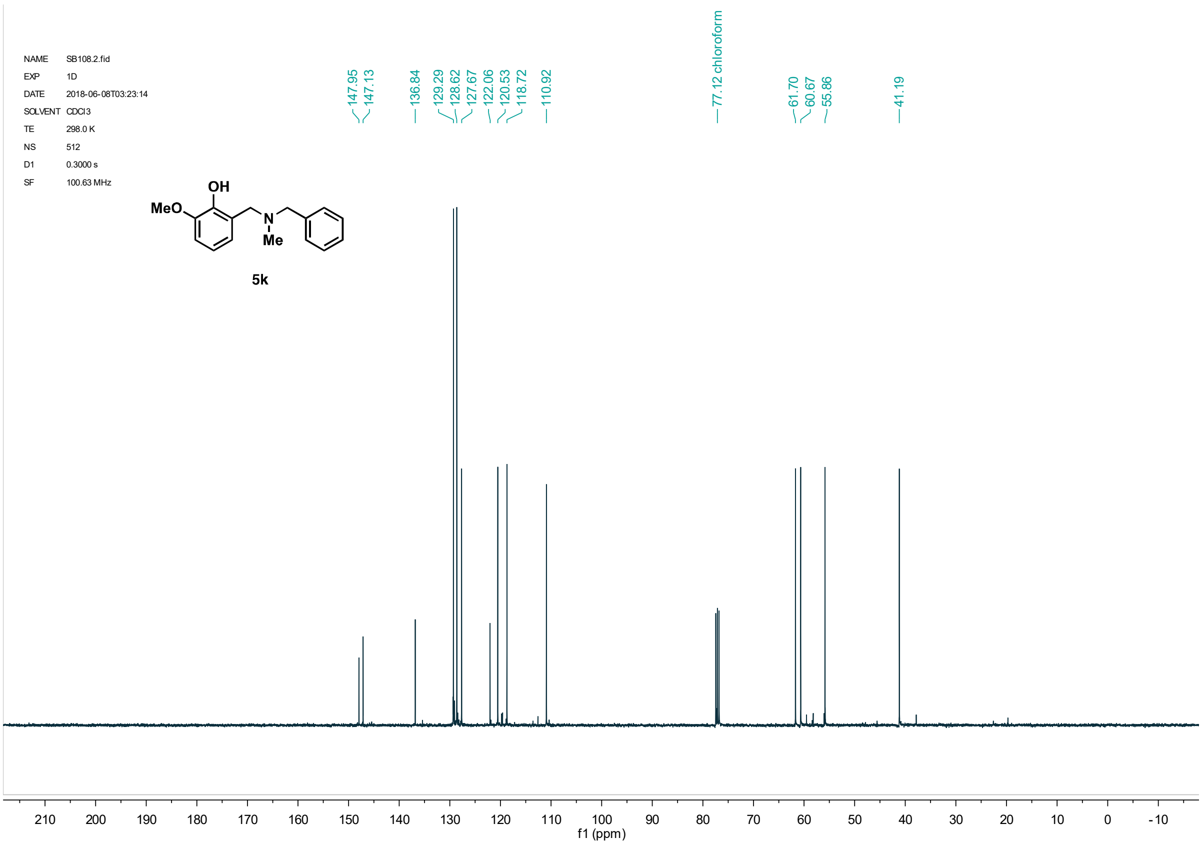

^{1}H NMR of the product **5l** obtained from Synple Chem synthesizer without purification:

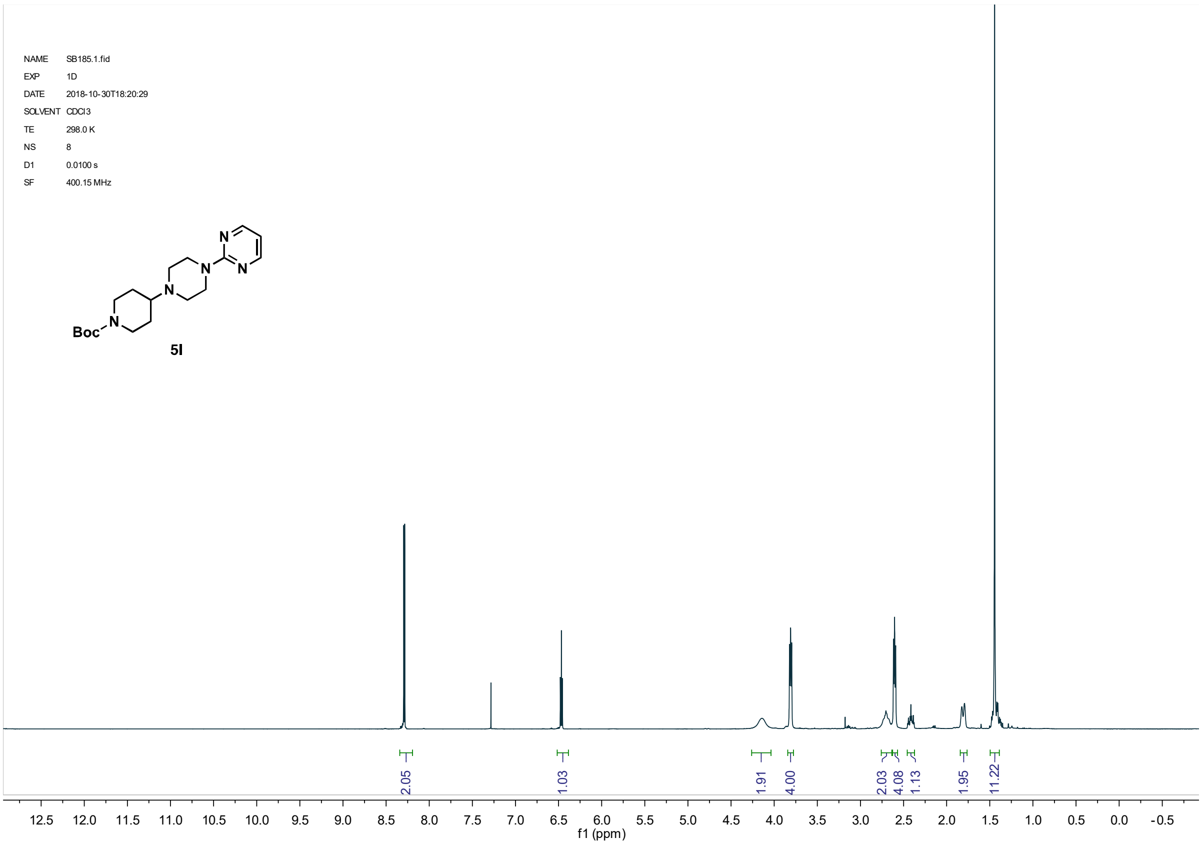

^{13}C NMR of the product **5l**:

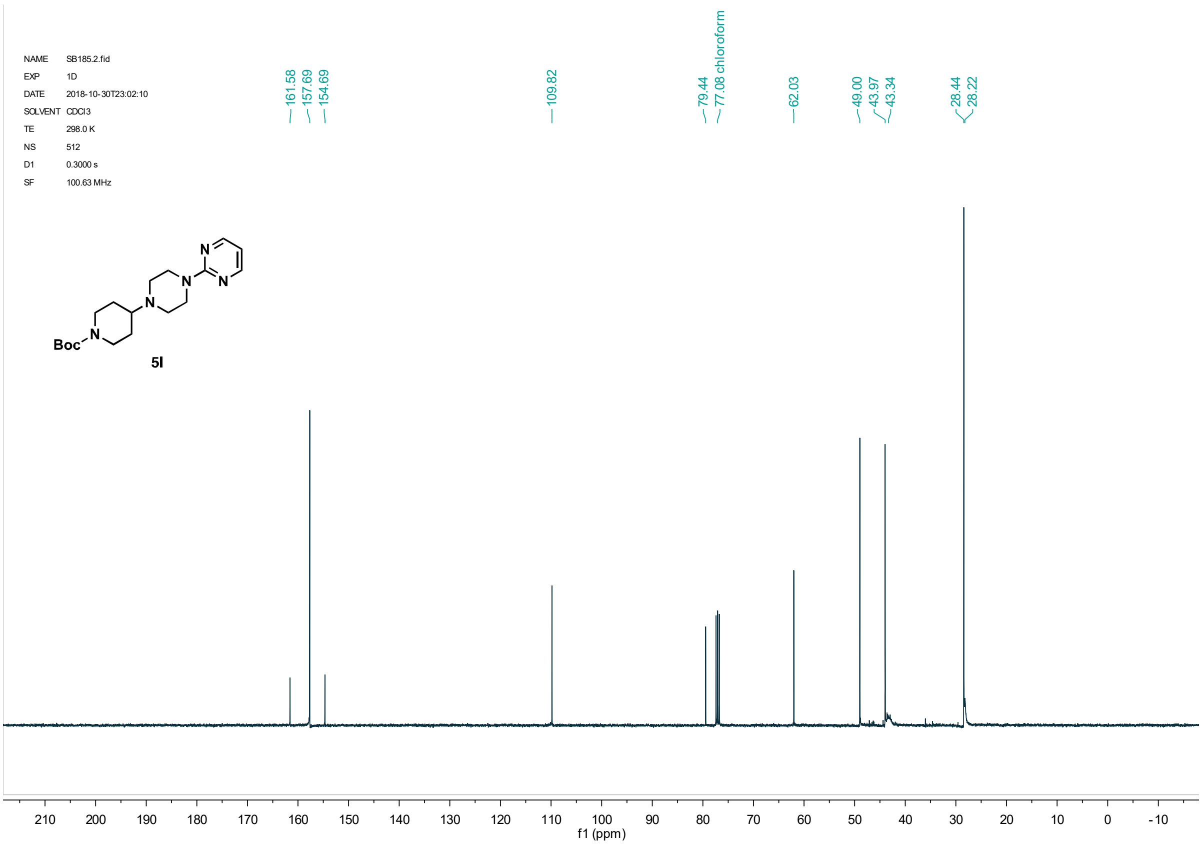

^{1}H NMR of compound **SM2**:

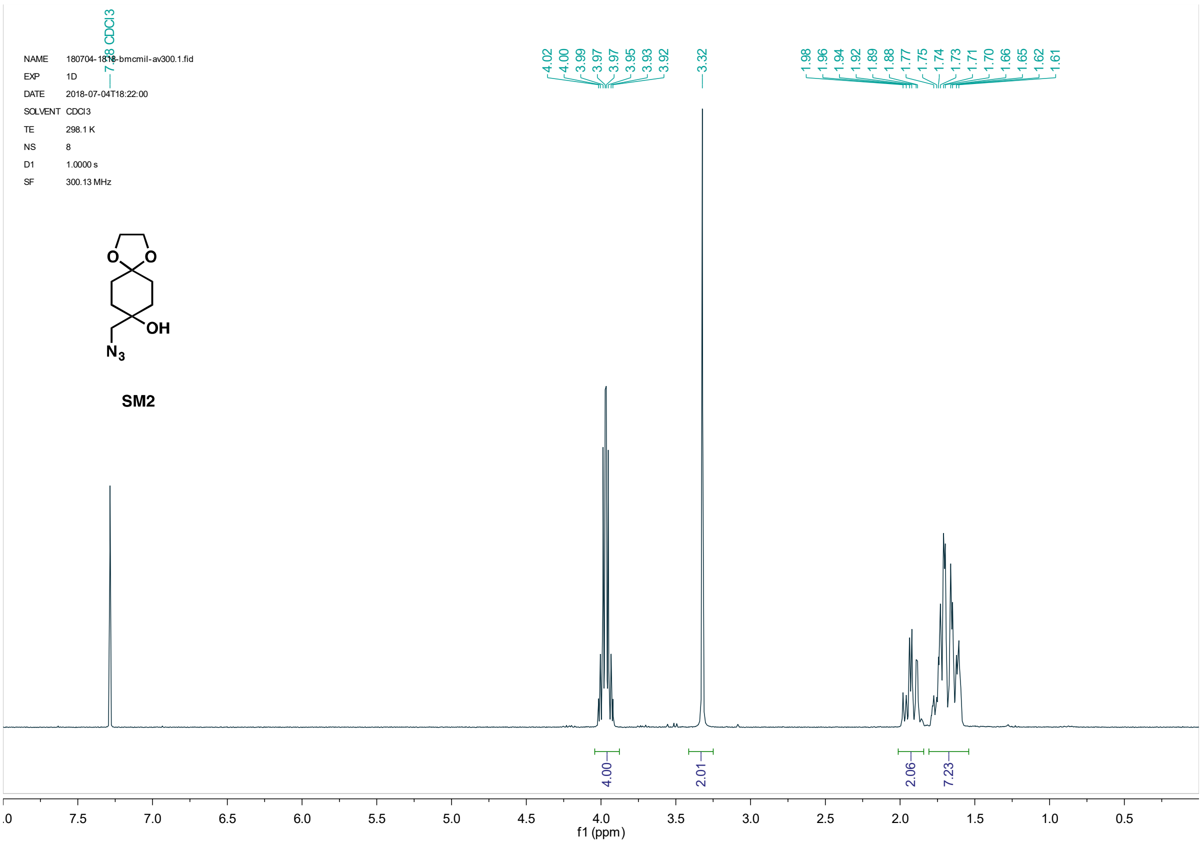

^{13}C NMR of compound **SM2**:

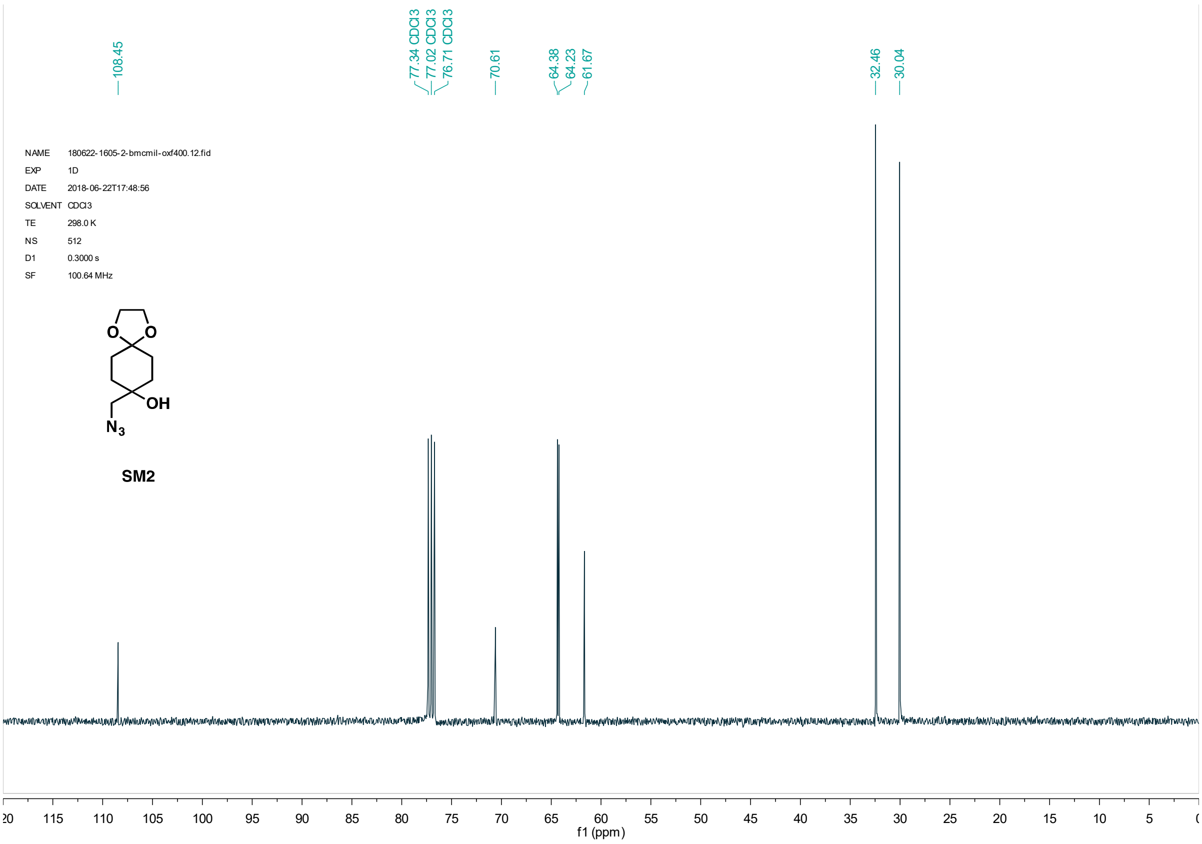

^{1}H NMR of compound **SM3**:

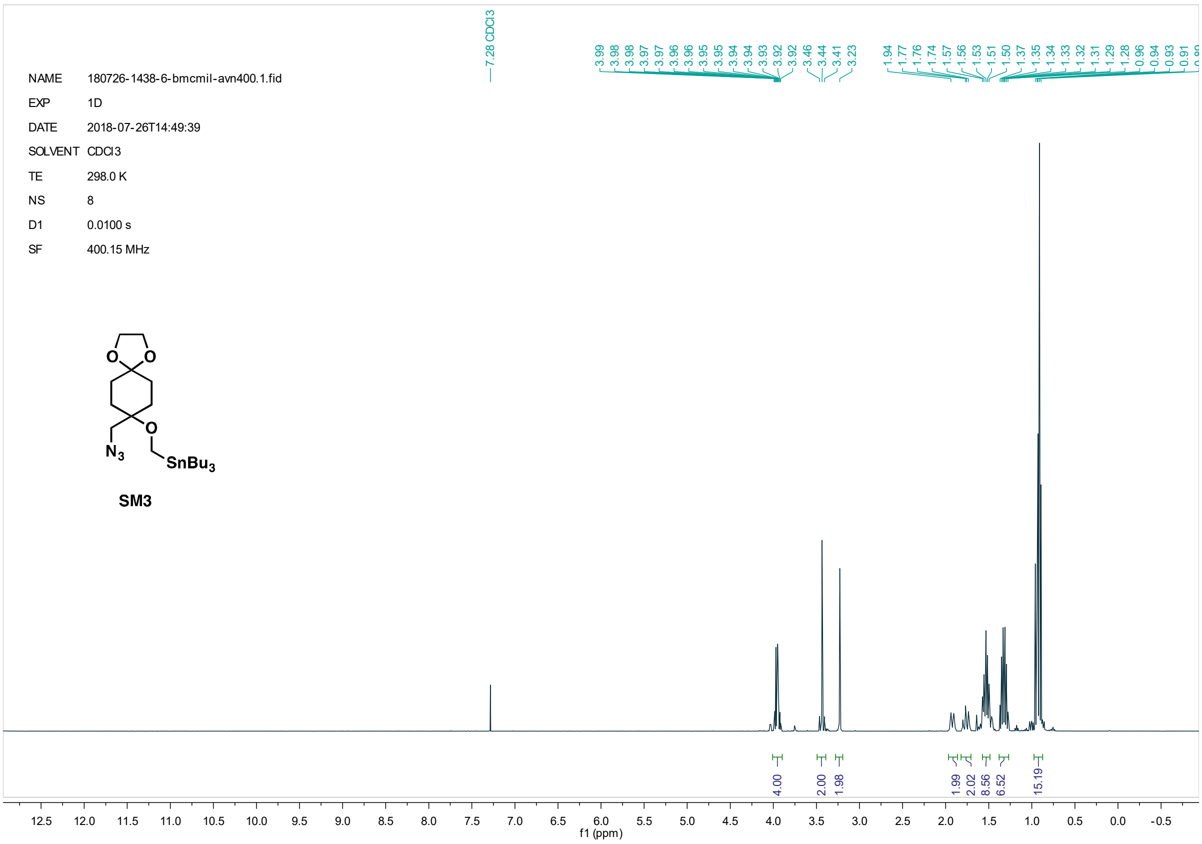

^{13}C NMR of compound **SM3**:

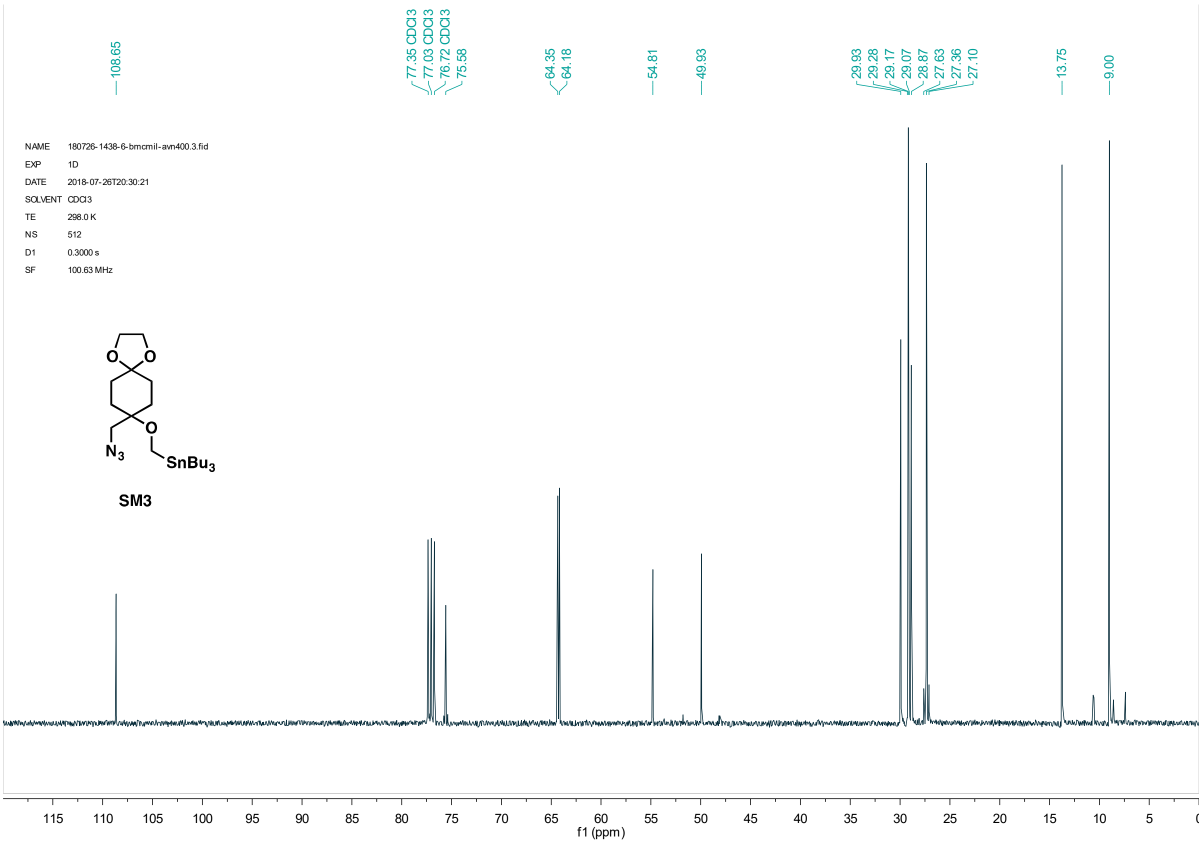

^{1}H NMR of compound **6a** major:

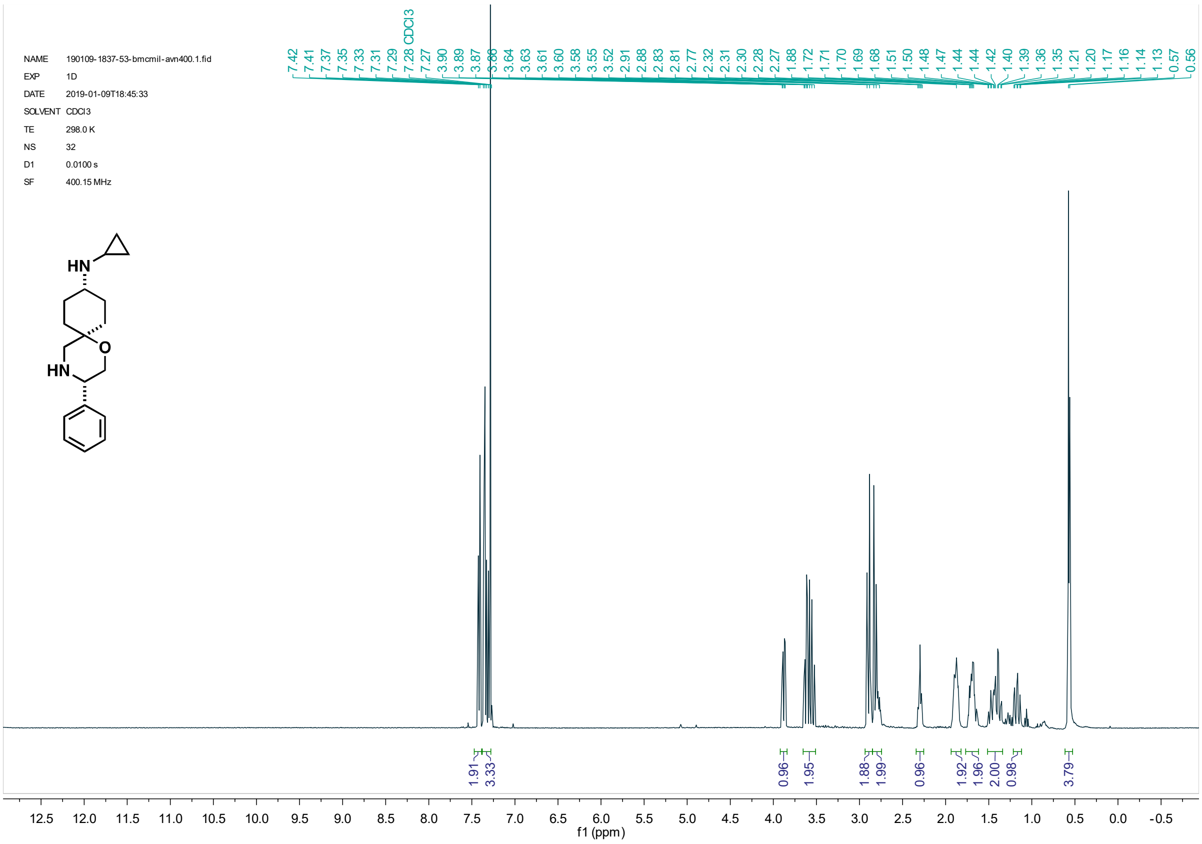

^{13}C NMR of compound **6a** major:

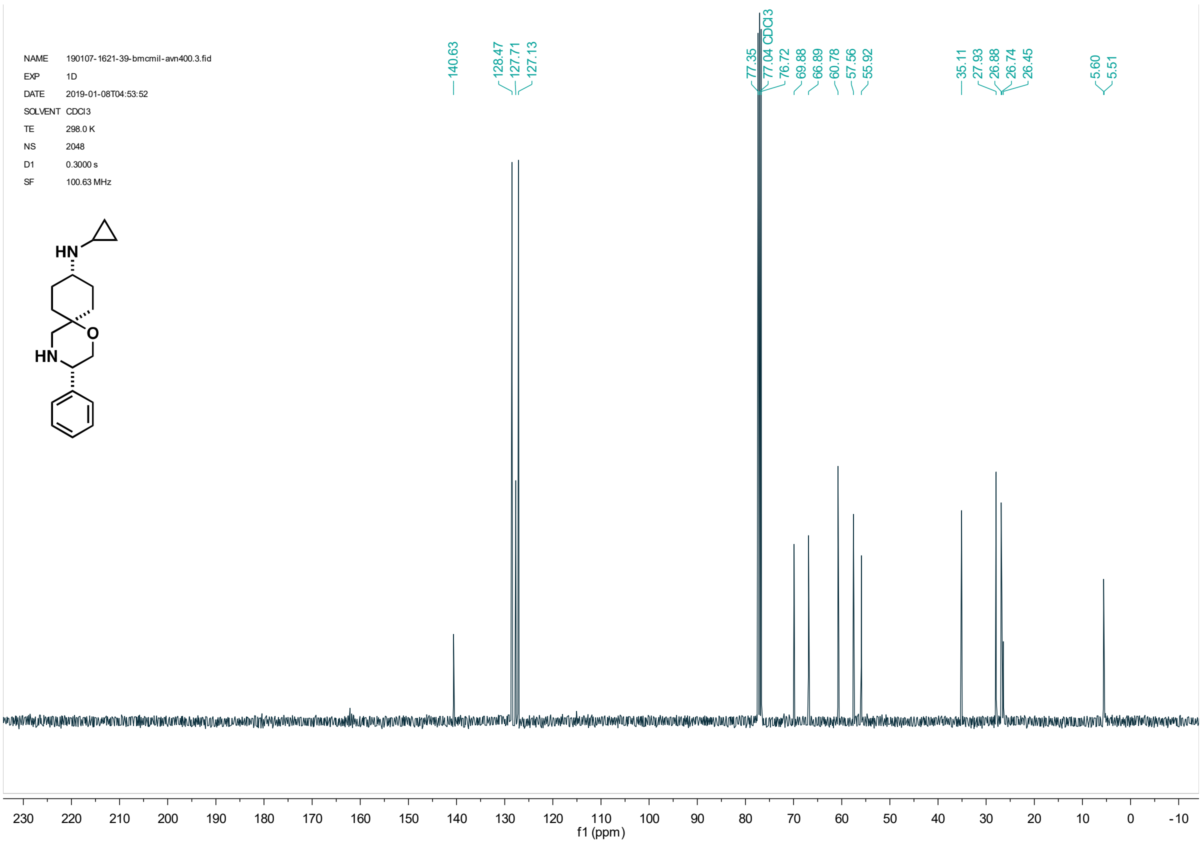

^{1}H NMR of compound **6a** minor:

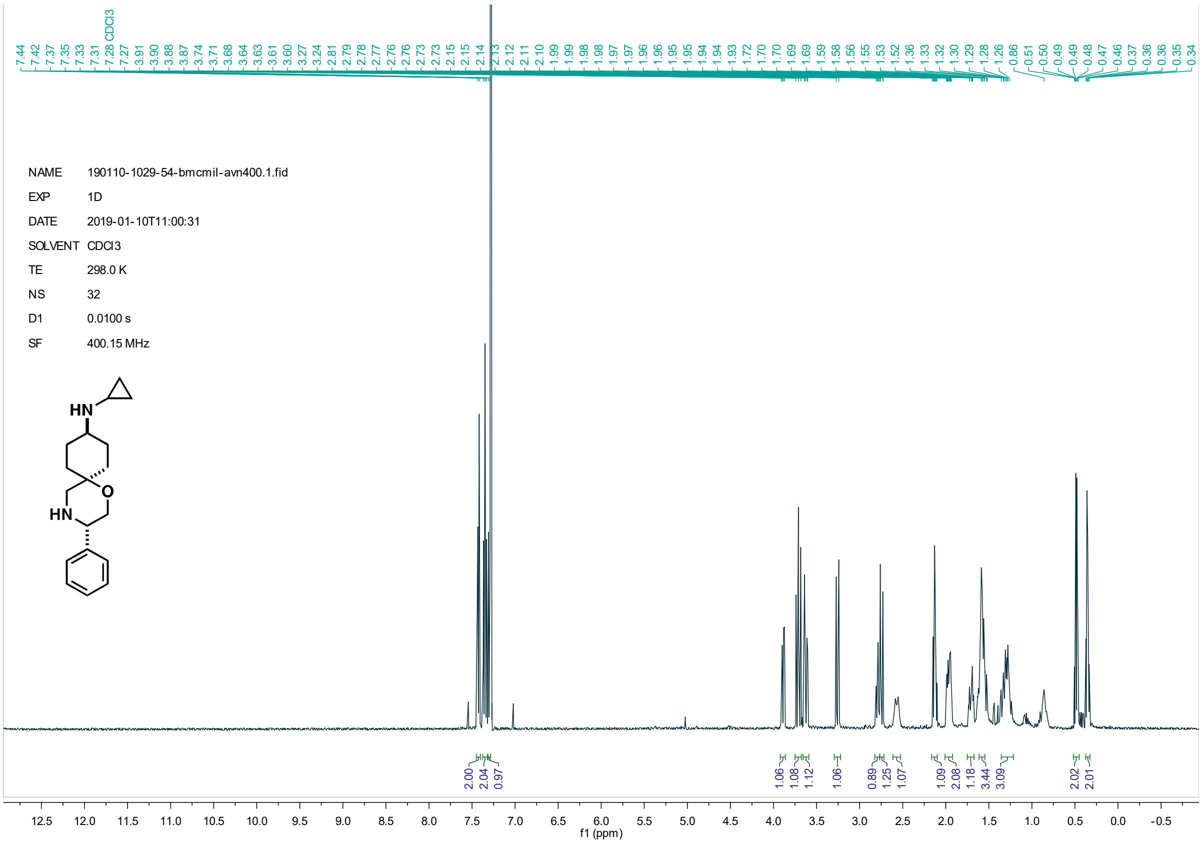

^{13}C NMR of compound **6a** minor:

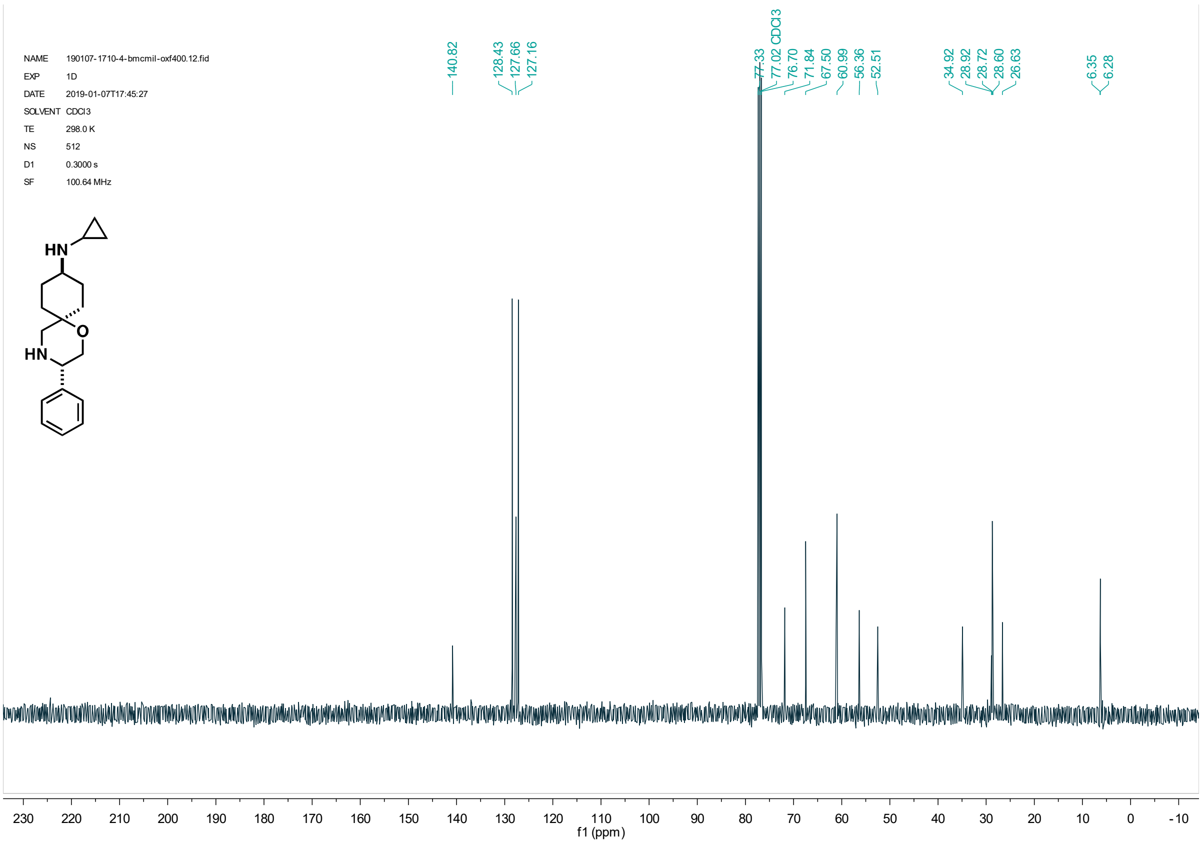

^{1}H NMR of compound **6b** major:

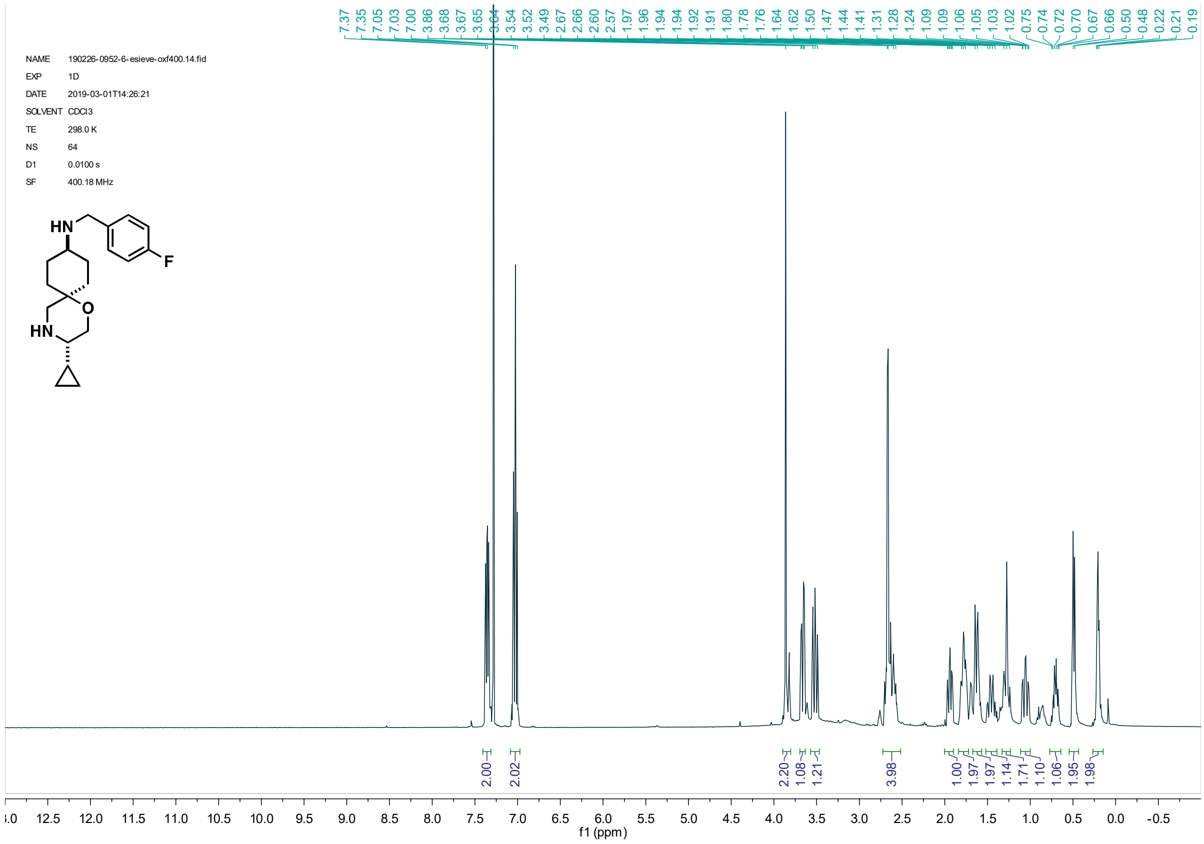

^{13}C NMR of compound **6b** major:

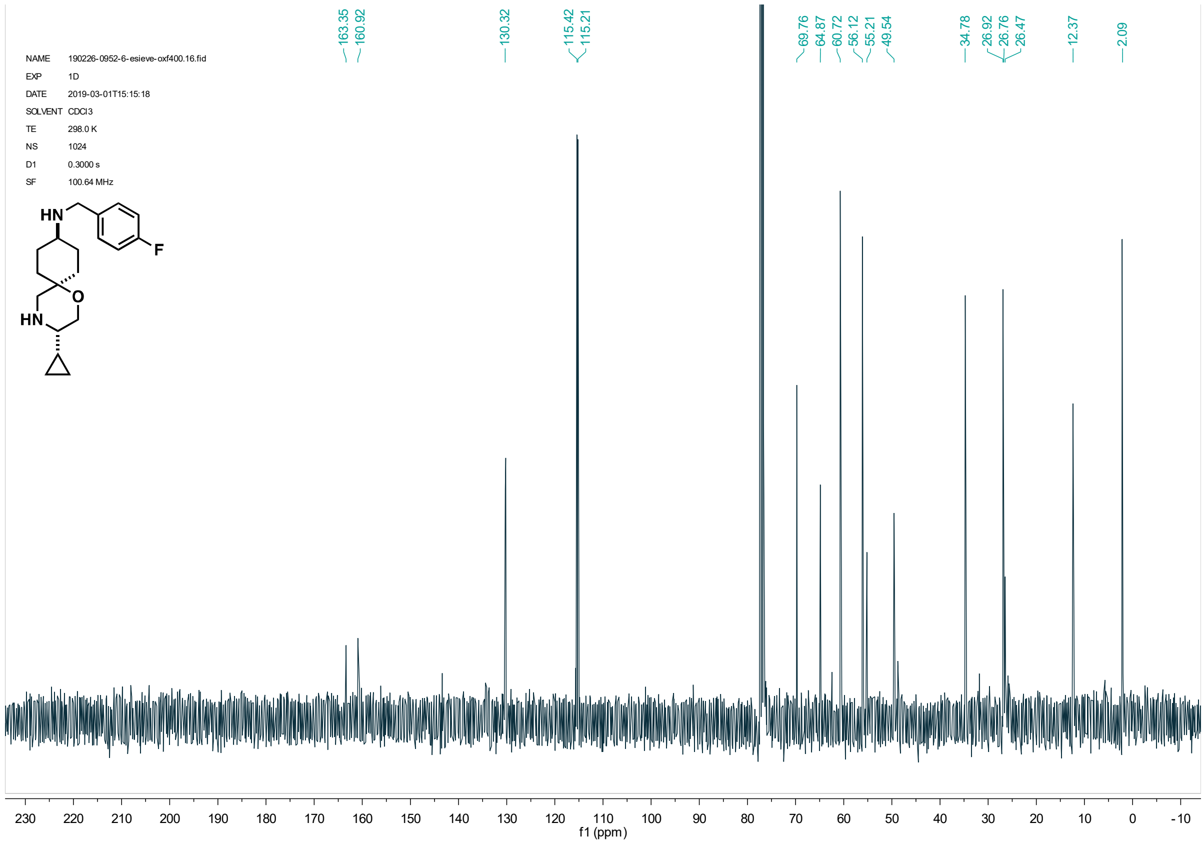

^{1}H NMR of compound **6b** minor:

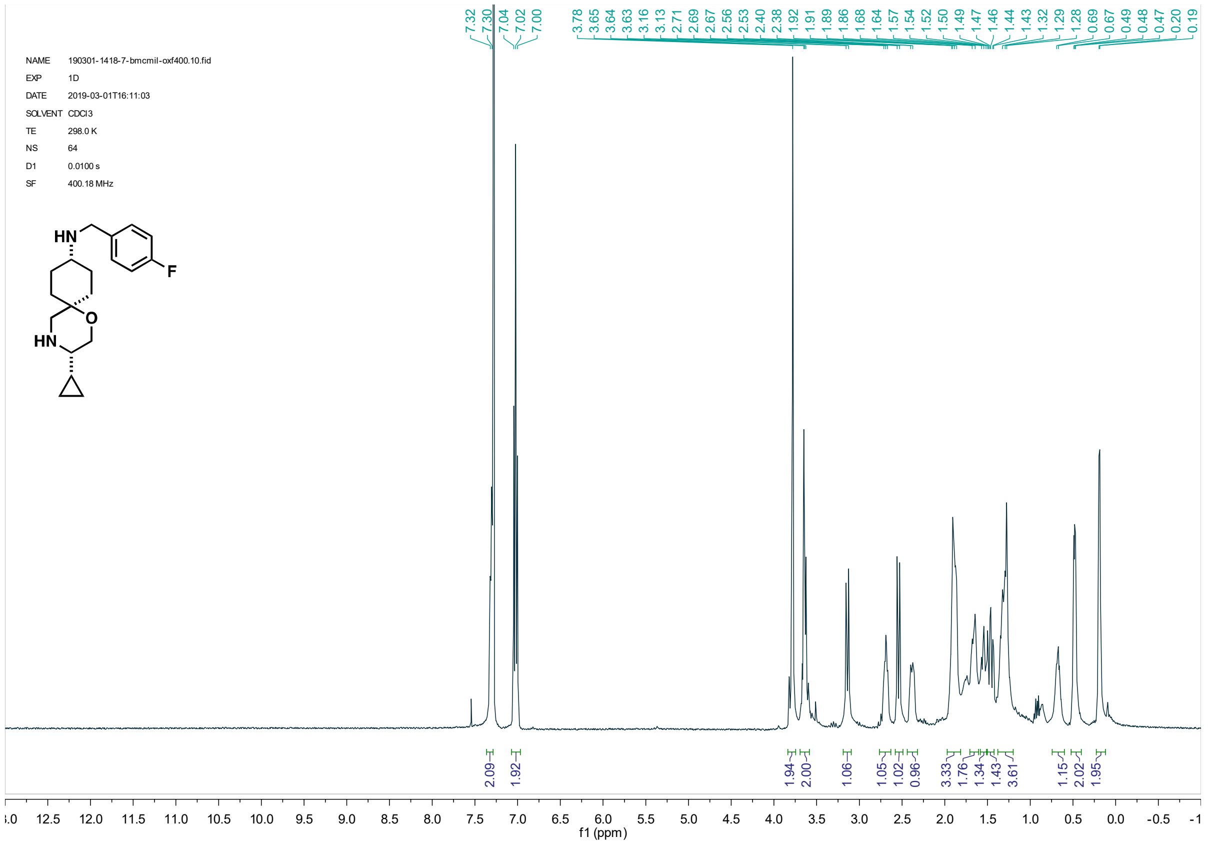

^{13}C NMR of compound **6b** minor:

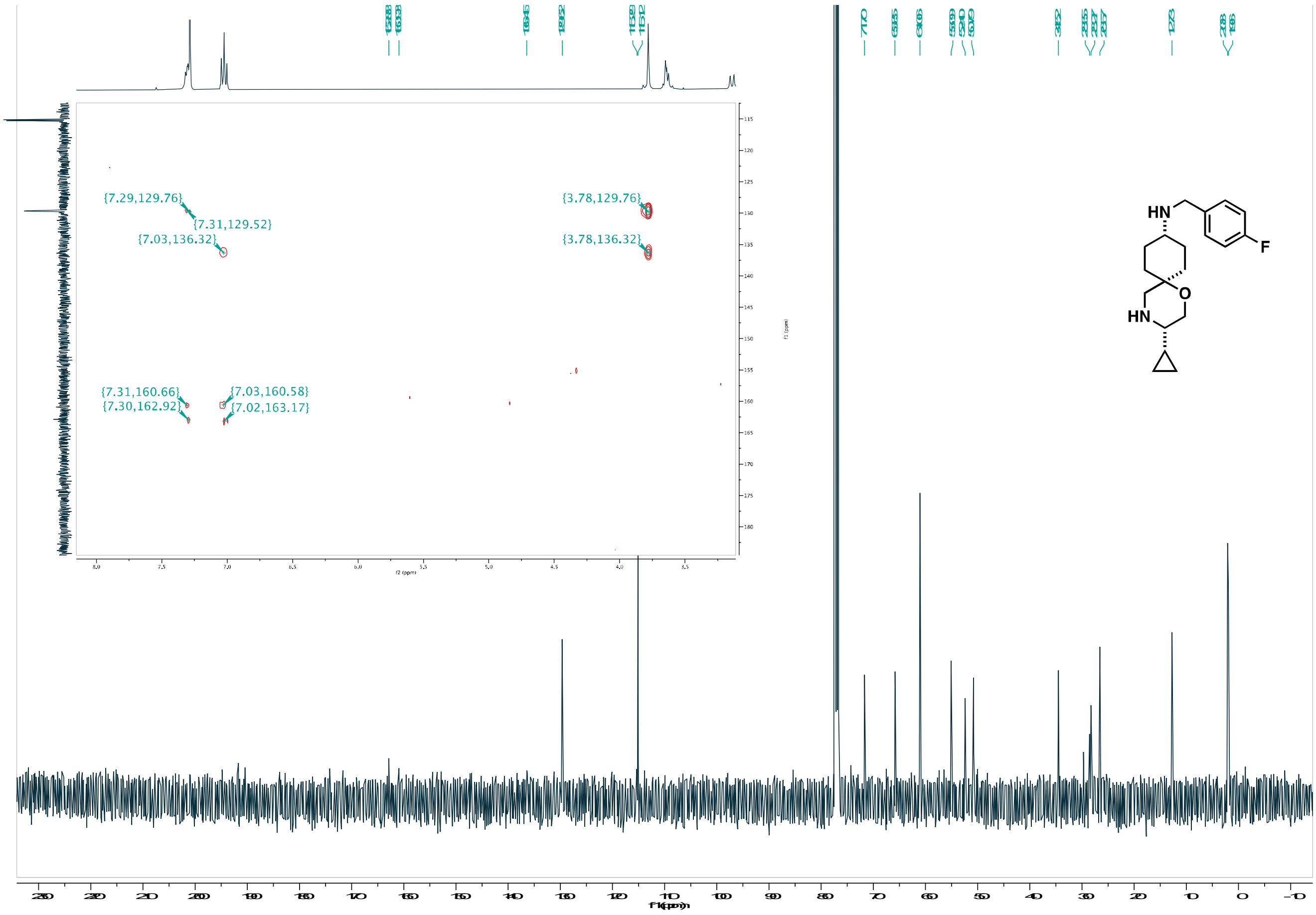

^{1}H NMR of compound **6c** major:

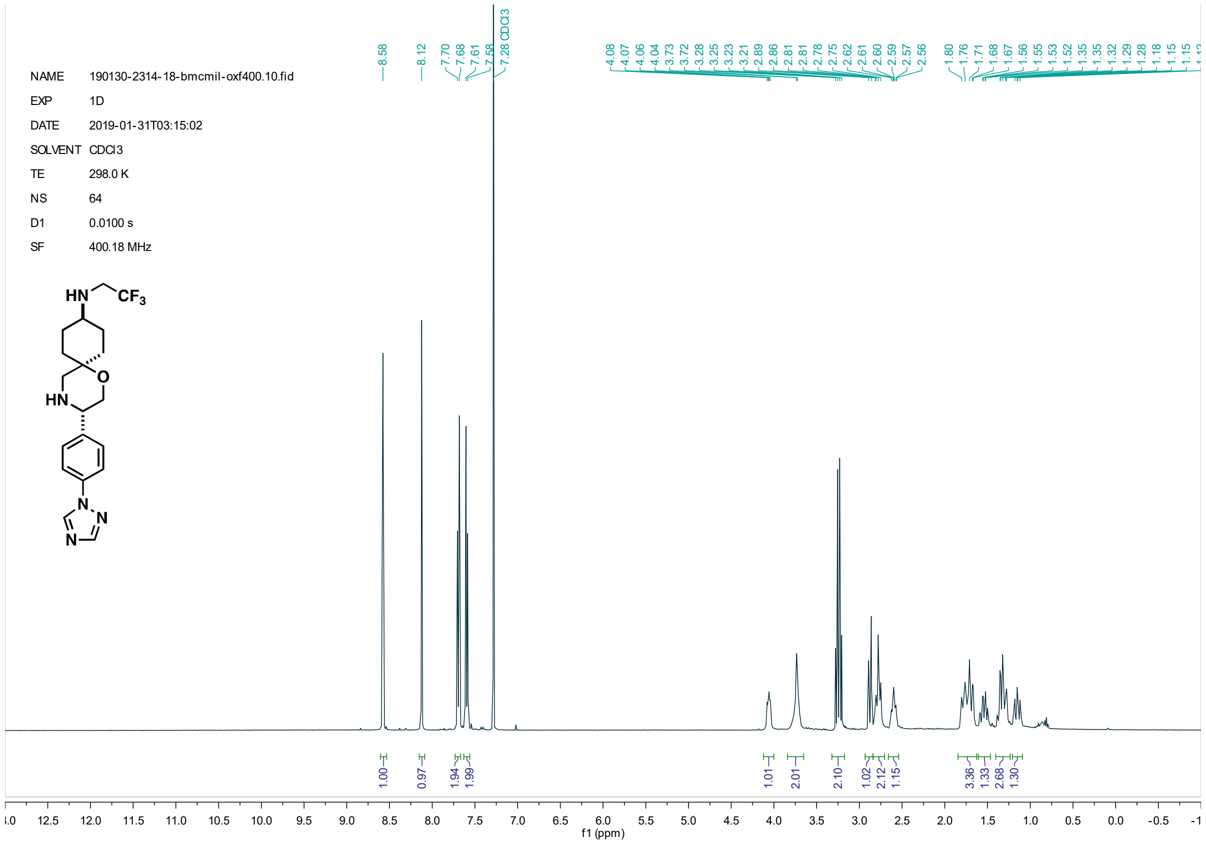

^{13}C NMR of compound **6c** major:

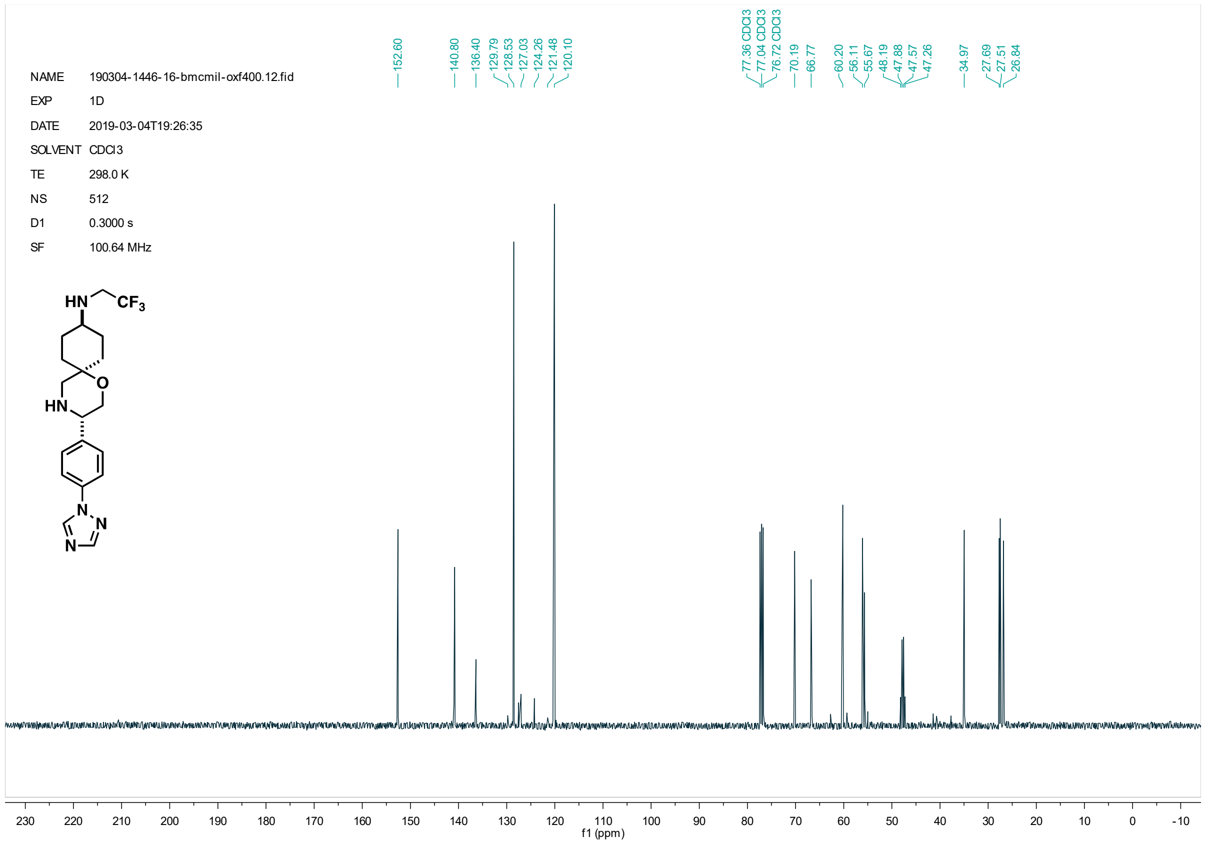

^{1}H NMR of compound **6c** minor:

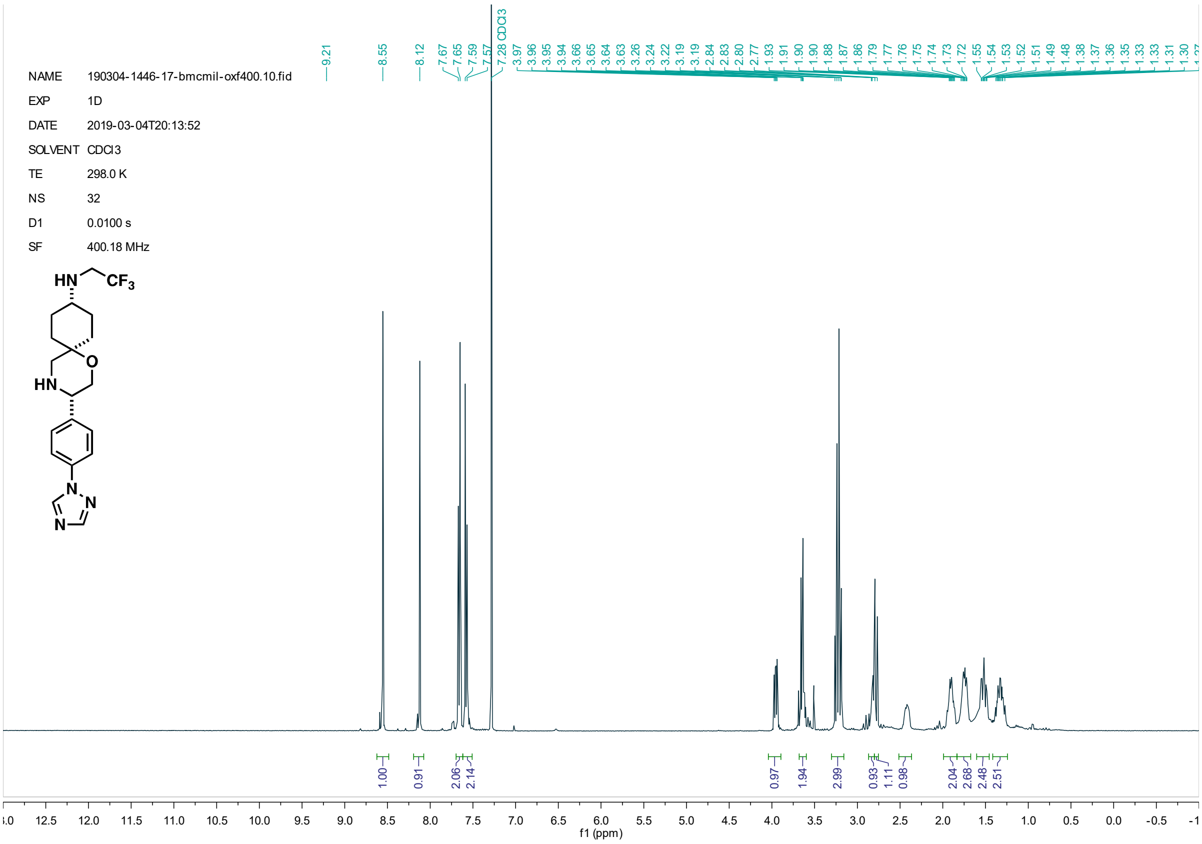

^{13}C NMR of compound **6c** minor:

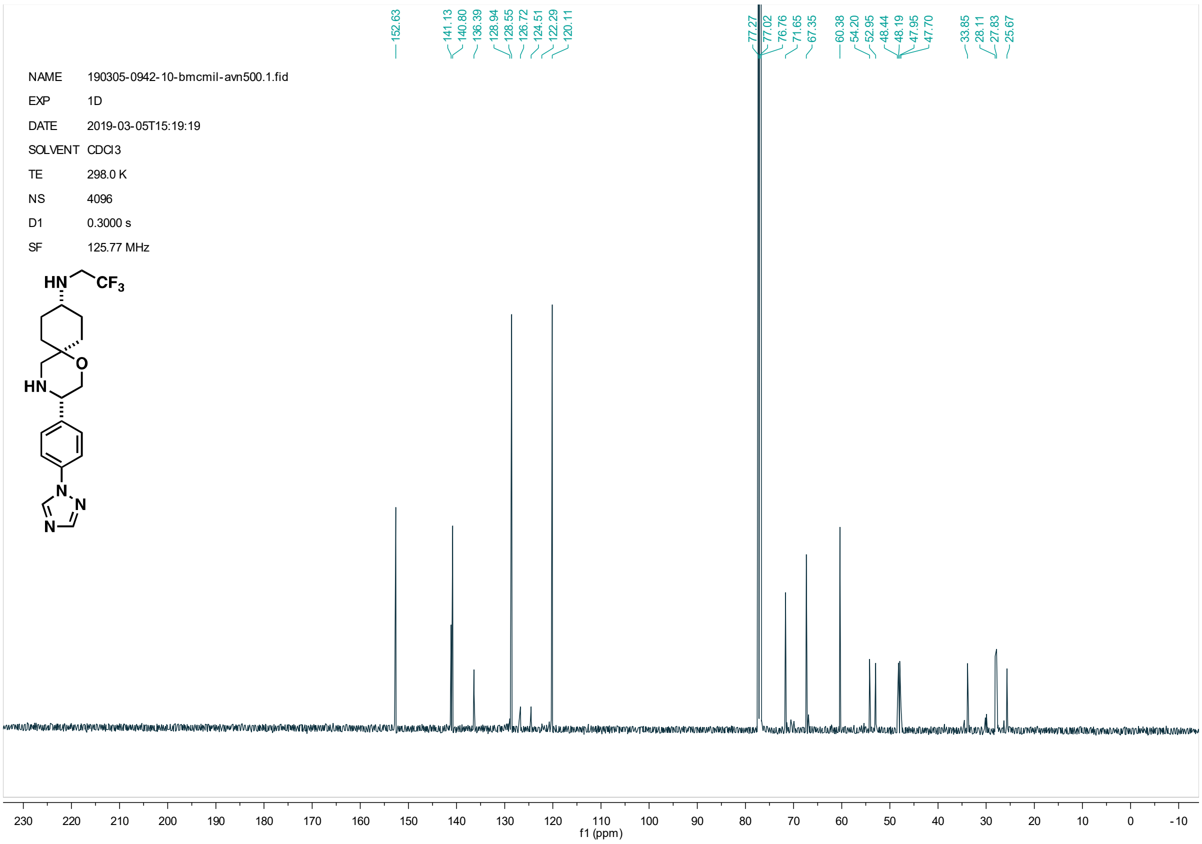

^{1}H NMR of compound **6d**:

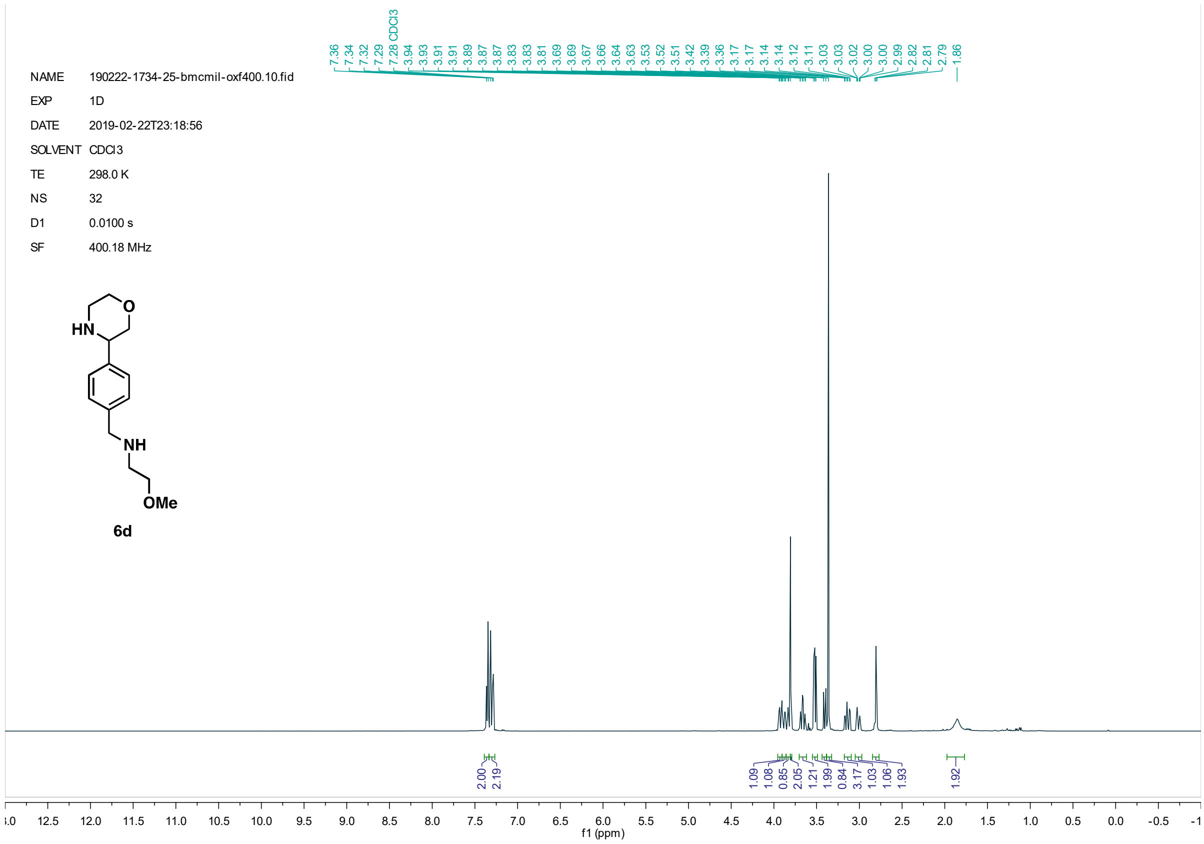

^{13}C NMR of compound **6d**:

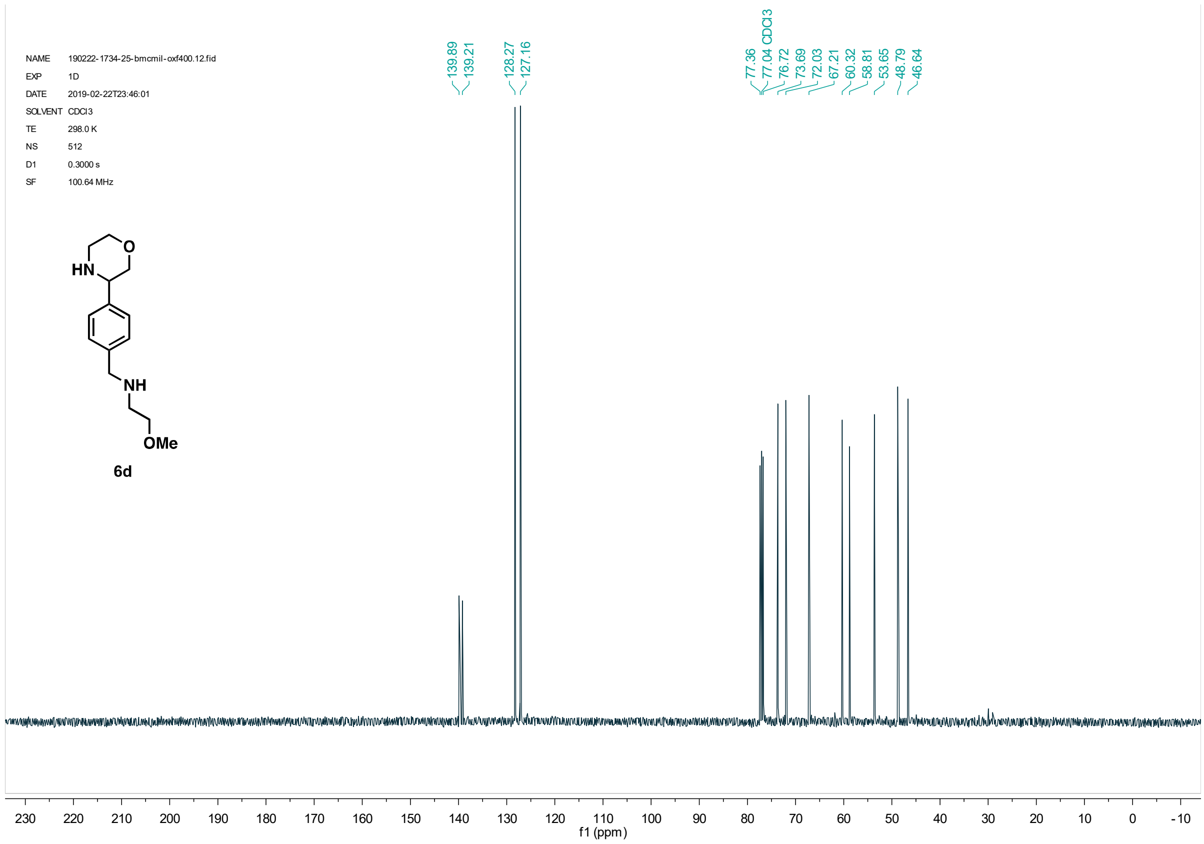

^{1}H NMR of compound **6e**:

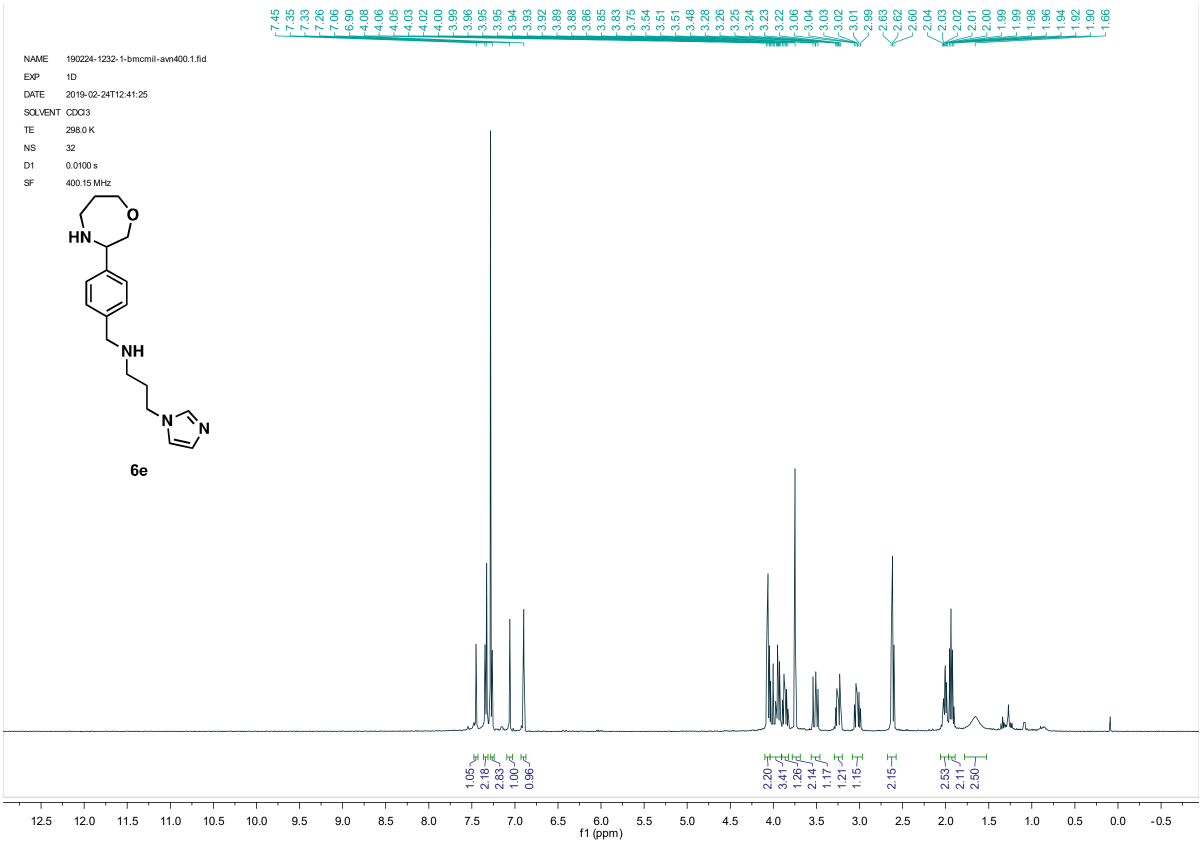

^{13}C NMR of compound **6e**:

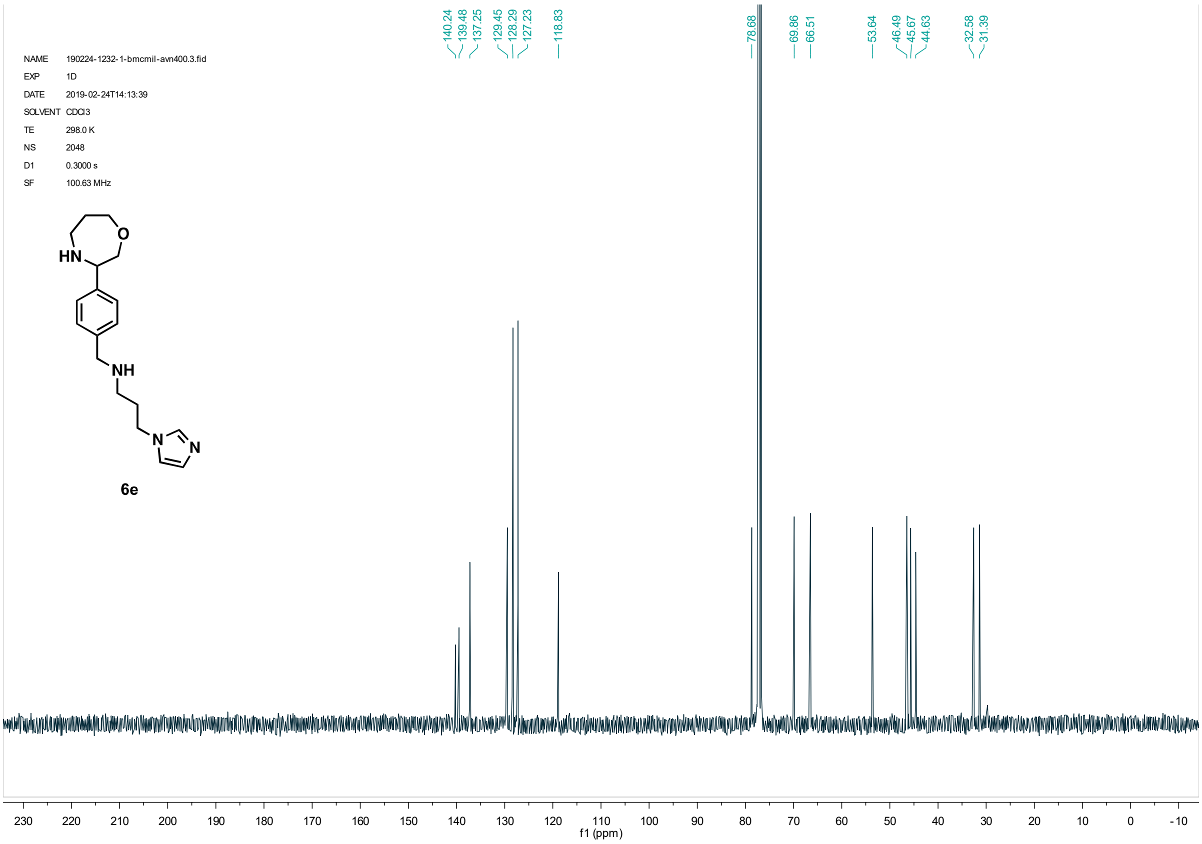

^{1}H NMR of compound **6f**:

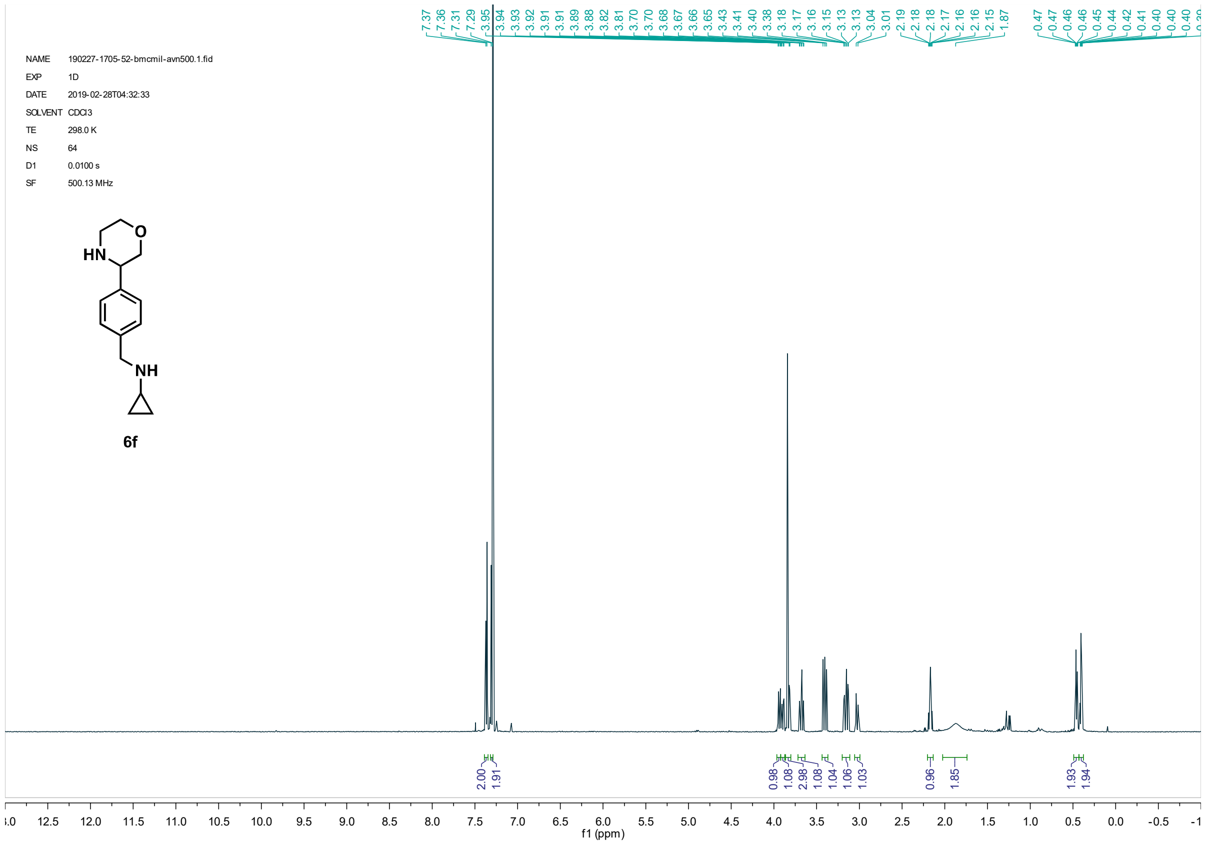

^{13}C NMR of compound **6f**:

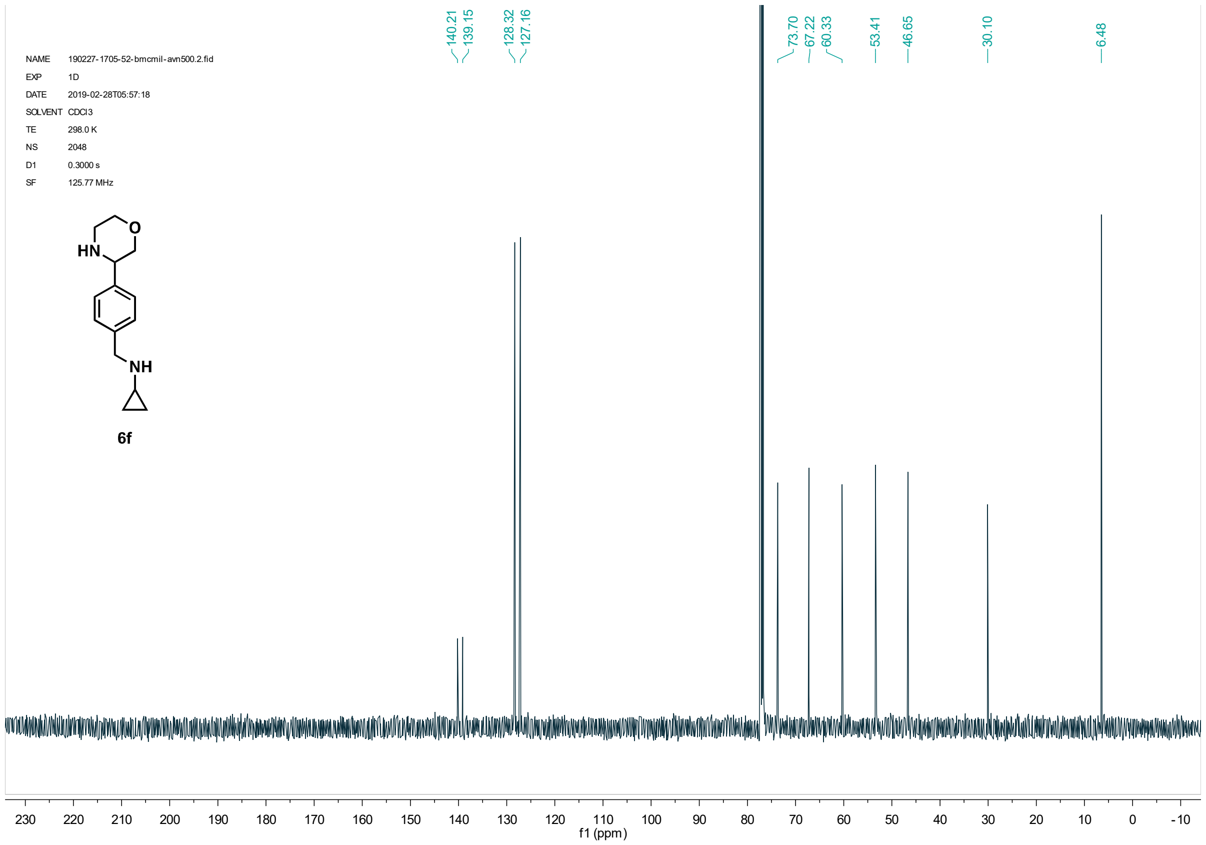

^{1}H NMR of compound **6J** major:

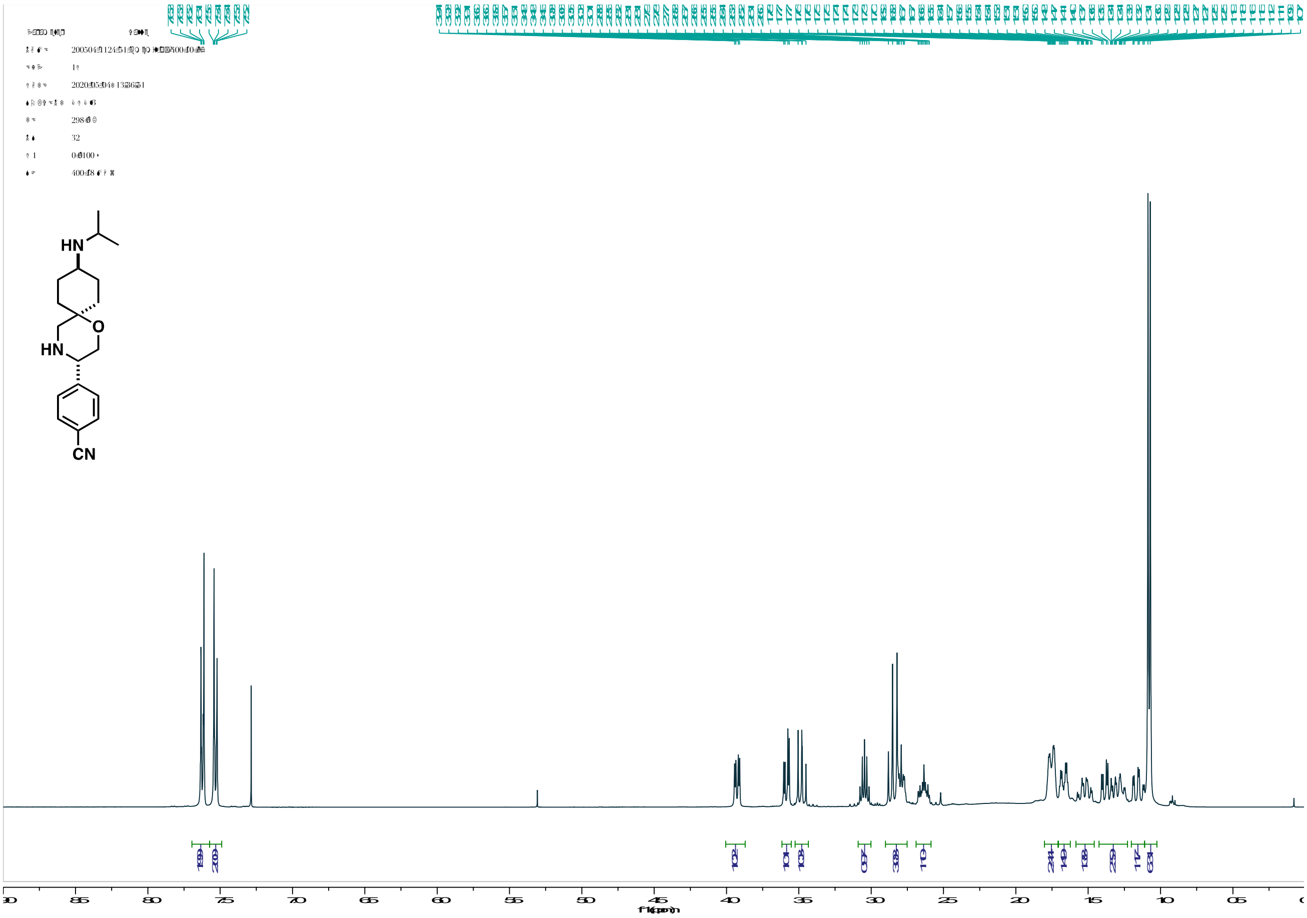

^{13}C NMR of compound **6J** major:

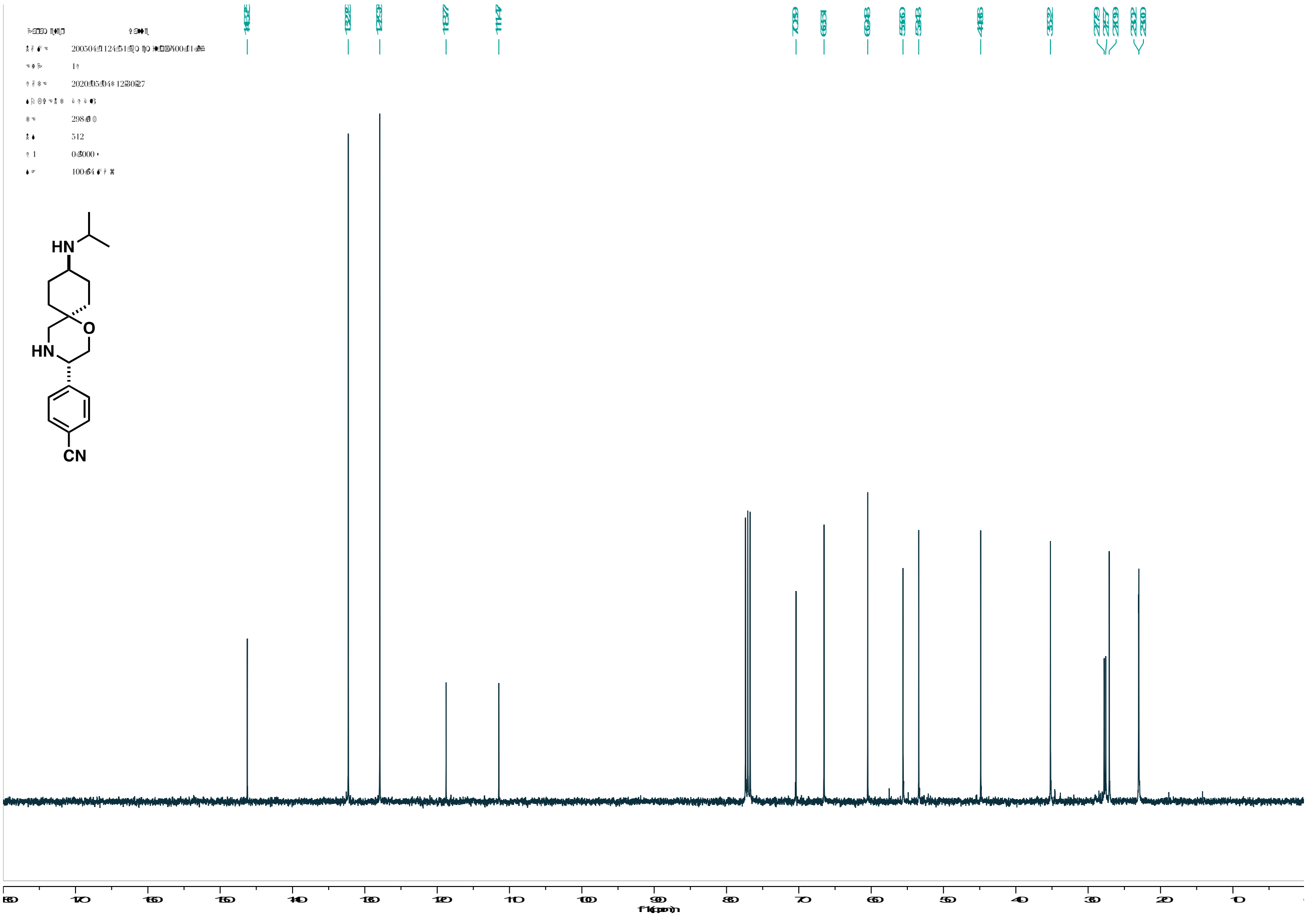

^{1}H NMR of compound **6J** minor:

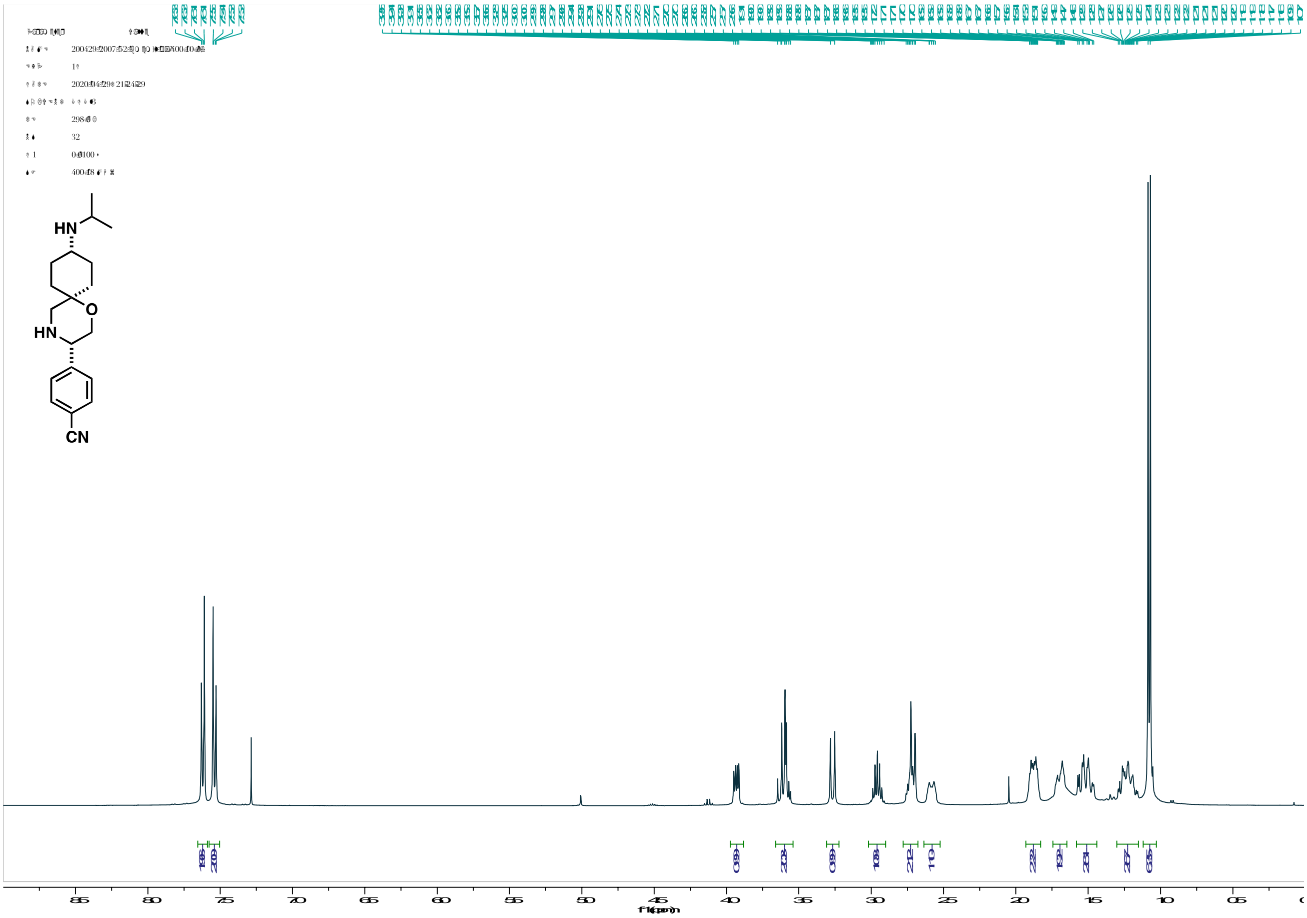

^{13}C NMR of compound **6J** minor:

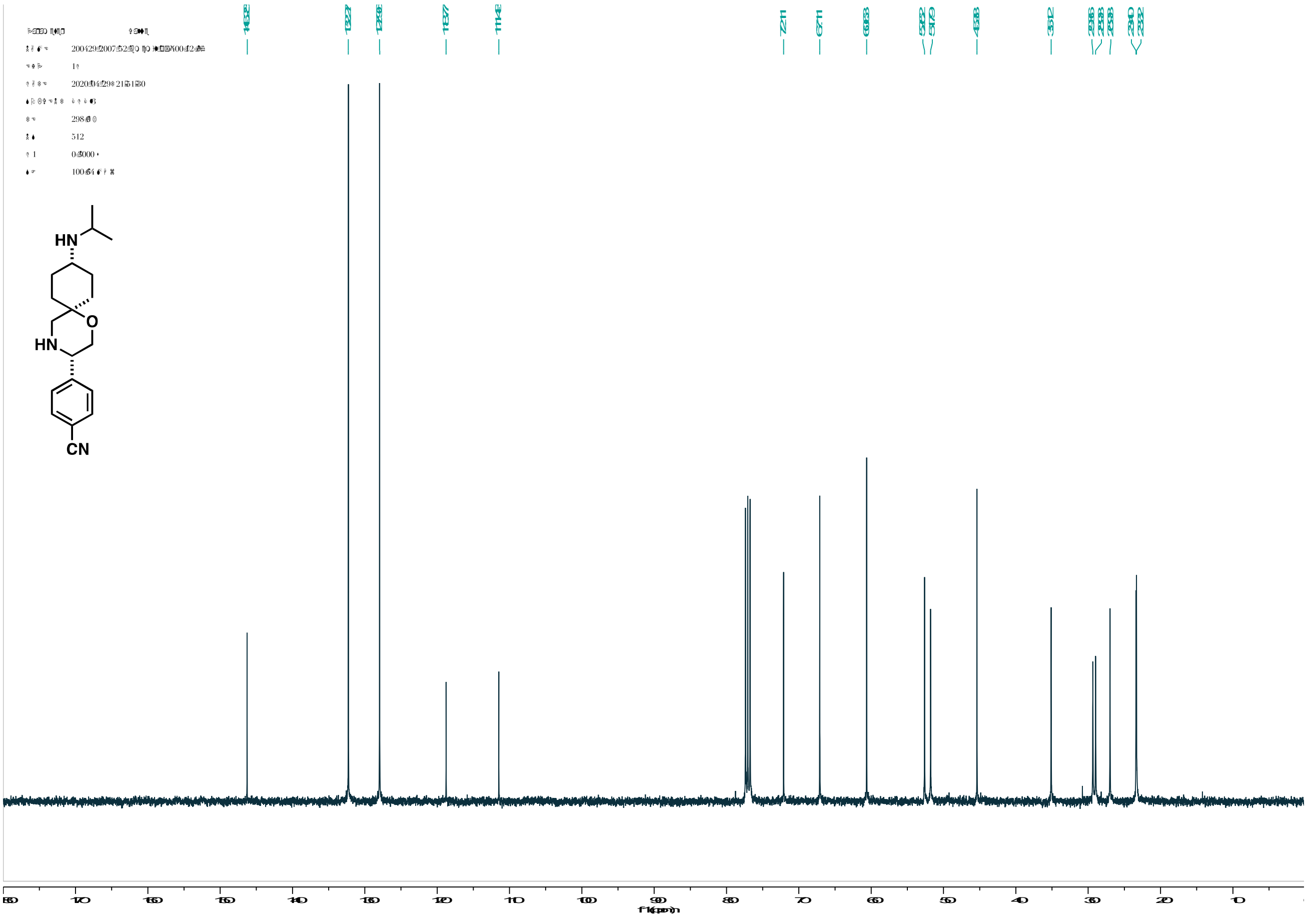

8. Additional References

1. A. Weber, E. von Roedern, H. U. Stilz, *J. Comb. Chem.*, 2005, **7**, 178.
2. T. Doi, S. Fuse, S. Miyamoto, K. Nakai, D. Sasuga, T. Takahashi, *Chem Asian J.*, 2006, **1**, 370.
3. K. Machida, Y. Hirose, S. Fuse, T. Sugawara, T. Takahashi, *Chem. Pharm. Bull.*, 2010, **58**, 87.
3. A. Buitrago Santanilla, E. L. Regalado, T. Pereira, M. Shevlin, K. Bateman, L.-C. Campeau, J. Schneeweis, S. Berritt, Z.-C. Shi, P. Nantermet, Y. Liu, R. Helmy, C. J. Welch, P. Vachal, I. W. Davies, T. Cernak, S. D. Dreher, *Science*, 2015, **347**, 49.
4. J. Li, S. G. Ballmer, E. P. Gillis, S. Fujii, M. J. Schmidt, A. M. E. Palazzolo, J. W. Lehmann, G. F. Morehouse, M. D. Burke, *Science*, 2015, **347**, 1221.
5. T. Tsubogo, H. Oyamada, S. Kobayashi, *Nature*, 2015, **520**, 329.
6. A. Adamo, R. L. Beingessner, M. Behnam, J. Chen, T. F. Jamison, K. F. Jensen, J.-C. M. Monbaliu, A. S. Myerson, E. M. Revalor, D. R. Snead, T. Stelzer, N. Weeranoppanant, S. Y. Wong, P. Zhang, *Science*, 2016, **352**, 61.
7. D. E. Fitzpatrick, S. V. Ley, *React. Chem. Eng.*, 2016, **1**, 629.
8. N. Holmes, G. R. Akien, R. J. D. Savage, C. Stanetty, I. R. Baxendale, A. J. Blacker, B. A. Taylor, R. L. Woodward, R. E. Meadows, R. A. Bourne, *React. Chem. Eng.*, 2016, **1**, 96.
9. V. Dragone, V. Sans, A. B. Henson, J. M. Granda, L. Cronin, *Nat Commun*, 2017, **8**, 15733.
10. H. Masui, S. Yosugi, S. Fuse and T. Takahashi, *Beilstein J. Org. Chem.*, 2017, **13**, 106.
11. A.-C. Bédard, A. Adamo, K. C. Aroh, M. G. Russell, A. A. Bedermann, J. Torosian, B. Yue, K. F. Jensen, T. F. Jamison, *Science*, 2018, **361**, 1220.
12. J. M. Granda, L. Donina, V. Dragone, D.-L. Long, L. Cronin, *Nature*, 2018, **559**, 377.
13. D. Perera, J. W. Tucker, S. Brahmbhatt, C. J. Helal, A. Chong, W. Farrell, P. Richardson, N. W. Sach, *Science*, 2018, **359**, 429.
14. C. W. Coley, D. A. Thomas, J. A. M. Lummiss, J. N. Jaworski, C. P. Breen, V. Schultz, T. Hart, J. S. Fishman, L. Rogers, H. Gao, R. W. Hicklin, P. P. Plehiers, J. Byington, J. S. Piotti, W. H. Green, A. J. Hart, T. F. Jamison, K. F. Jensen, *Science*, 2019, **365**, eaax1566.
15. S. Steiner, J. Wolf, S. Glatzel, A. Andreou, J. M. Granda, G. Keenan, T. Hinkley, G. Aragon-Camarasa, P. J. Kitson, D. Angelone, L. Cronin, *Science*, 2019, **363**, eaav2211.
16. N. Collins, D. Stout, J.-P. Lim, J. P. Malerich, J. D. White, P. B. Madrid, M. Latendresse, D. Krieger, J. Szeto, V.-A. Vu, K. Rucker, M. Deleo, Y. Gorfu, M. Krummenacker, L. A. Hokama, P. Karp, S. Mallya, *Org. Process Res. Dev.*, 2020, **24**, 2064.
17. A. A. Joseph, A. Pardo-Vargas, P. H. Seeberger, *J. Am. Chem. Soc.*, 2020, **142**, 8561.
18. S. H. M. Mehr, M. Craven, A. I. Leonov, G. Keenan, L. Cronin, *Science*, 2020, **370**, 101.
19. A. C. Vaucher, F. Zipoli, J. Geluykens, V. H. Nair, P. Schwaller, T. Laino, *Nat. Commun.*, 2020, **11**, 3601.
20. L. Wilbraham, S. H. M. Mehr, L. Cronin, *Acc. Chem. Res.*, 2021, **54**, 253.
21. V. Sans, L. Porwol, V. Dragone, L. Cronin, *Chem. Sci.*, 2015, **6**, 1258.
22. J. E. Hochlowski, P. A. Searle, N. P. Tu, J. Y. Pan, S. G. Spanton, S. W. Djuric, *J. Flow Chem.*, 2011, **1**, 56.
23. S. D. Pickett, D. V. S. Green, D. L. Hunt, D. A. Pardoe, I. Hughes, *ACS Med. Chem. Lett.*, 2011, **2**, 28.
24. W. Czechtizky, J. Dedio, B. Desai, K. Dixon, E. Farrant, Q. Feng, T. Morgan, D. M. Parry, M. K. Ramjee, C. N. Selway, T. Schmidt, G. J. Tarver, A. G. Wright, *ACS Med. Chem. Lett.*, 2013, **4**, 768.
25. B. Desai, K. Dixon, E. Farrant, Q. Feng, K. R. Gibson, W. P. van Hoorn, J. Mills, T. Morgan, D. M. Parry, M. K. Ramjee, C. N. Selway, G. J. Tarver, G. Whitlock, A. G. Wright, *J. Med. Chem.*, 2013, **56**, 3033.
26. L. Guetzoyan, N. Nikbin, I. R. Baxendale, S. V. Ley, *Chem. Sci.*, 2013, **4**, 764.
27. M. Werner, C. Kuratli, R. E. Martin, R. Hochstrasser, D. Wechsler, T. Enderle, A. I. Alanine, H. Vogel, *Angew. Chem. Int. Ed.*, 2014, **53**, 1704.
28. S. M. Pant, A. Mukonoweshuro, B. Desai, M. K. Ramjee, C. N. Selway, G. J. Tarver, A. G. Wright, K. Birchall, T. M. Chapman, T. A. Tervonen, J. Klefström, *J. Med. Chem.*, 2018, **61**, 4335.
29. C. A. Nicolaou, C. Humblet, H. Hu, E. M. Martin, F. C. Dorsey, T. M. Castle, K. I. Burton, H. Hu, J. Hendle, M. J. Hickey, J. Duerksen, J. Wang, J. A. Erickson, *ACS Med. Chem. Lett.*, 2019, **10**, 278.
30. D. M. Parry, *ACS Med. Chem. Lett.*, 2019, **10**, 848.
31. B. M. Wanner, P. L. Nichols, T. Jiang, *CHIMIA International Journal for Chemistry*, 2020, **74**, 808.
32. Biotage, https://www.biotage.com/initiator-robot-eight-microwave-synthesis, (accessed 21 February 2021).
33. Mettler Toledo, https://www.mt.com/ch/en/home/products/L1_AutochemProducts/Chemical-Synthesis-and-Process-Development-Lab-Reactors/Synthesis-Reactor-Systems.html, (accessed 21 February 2021).
34. Keison, http://www.keison.co.uk/electrothermal_integrity10.shtml, (accessed 21 February 2021).
35. Anton Paar, https://www.anton-paar.com/corp-en/products/details/microwave-synthesis-monowave-400200/, (accessed 21 February 2021).
36. Chemspeed, https://www.chemspeed.com/technologies/reactors-vessels-vials/, (accessed 21 February 2021).
37. Vapourtec, https://www.vapourtec.com/, (accessed 21 February 2021).
38. Syrris, https://www.syrris.com/, (accessed 21 February 2021).
39. Uniqsis, https://www.uniqsis.com/, (accessed 21 February 2021).
